# Comparative Transcriptomics in East African Cichlids Reveals Sex- and Species-Specific Expression and New Candidates for Sex Differentiation in Fishes

**DOI:** 10.1093/gbe/evu200

**Published:** 2014-09-17

**Authors:** Astrid Böhne, Thierry Sengstag, Walter Salzburger

**Affiliations:** ^1^Zoological Institute, University of Basel, Switzerland; ^2^SIB Swiss Institute of Bioinformatics and sciCORE Computing Center, University of Basel, Switzerland

**Keywords:** sex determination, speciation, cichlid fish, steroid hormones, TGF-β-superfamily, Wnt-pathway

## Abstract

Males and females of the same species differ largely in gene expression, which accounts for most of the morphological and physiological differences and sex-specific phenotypes. Here, we analyzed sex-specific gene expression in the brain and the gonads of cichlid fishes from Lake Tanganyika belonging to four different lineages, so-called tribes (Eretmodini, Ectodini, Haplochromini, and Lamprologini), using the outgroup Nile tilapia (*Oreochromis niloticus*) as reference. The comparison between male and female brains revealed few differences between the sexes, consistent in all investigated species. The gonads, on the other hand, showed a large fraction of differentially expressed transcripts with the majority of them showing the same direction of expression in all four species. All here-studied cichlids, especially the three investigated mouth-breeding species, showed a trend toward more male- than female-biased transcripts. Transcripts, which were female-biased in expression in all four species, were overrepresented on linkage group (LG)1 in the reference genome and common male-biased transcripts showed accumulation on LG23, the presumable sex chromosomes of the Nile tilapia. Sex-specific transcripts contained candidate genes for sex determination and differentiation in fishes, especially members of the transforming growth factor-β-superfamily and the Wnt-pathway and also prominent members of the sox-, dm-domain-, and high mobility group-box families. We further confirmed our previous finding on species/lineage-specific gene expression shifts in the sex steroid pathway, including synthesizing enzymes as the aromatase *cyp19a1* and estrogen and androgen receptors.

## Introduction

Fishes show a remarkable diversity of sex-determining (SD) systems, ranging from simple monogenetic determiners (Nanda et al. 2002) over systems with several sex chromosomes (Schultheis et al. 2009) to environmental sex determination (Baroiller, D’Cotta, Saillant, et al. 2009). Lately, whole-genome and reduced library/restriction site-associated DNA sequencing have helped to characterize some SD systems (e.g., Anderson et al. 2012; Palaiokostas, Bekaert, Davie, et al. 2013; Palaiokostas, Bekaert, Khan, et al. 2013; Rondeau et al. 2013); however, the actual genes and their genetic networks are still unknown in most fishes (Devlin and Nagahama 2002; Mank et al. 2006; Heule et al. 2014). Using genome-wide sequencing to study SD has the drawback of still being a costly and time-consuming endeavor, especially if several species are to be investigated and compared. In cases where the sequences of interest are coding, RNA-sequencing represents a suitable alternative, with the additional advantage of providing information about gene expression levels. This strategy has already been proven successful for the identification of the master SD gene in the rainbow trout (Yano et al. 2012).

Here, we set out to study the genetic network of sex differences in a particular fish group, the cichlid fishes of the East African Great Lakes. These fishes are textbook examples of explosive speciation and adaptive radiation (Kocher 2004) and, hence, a widely studied model system in evolutionary biology (Santos and Salzburger 2012). Because of the putative role of SD system turnovers in speciation (Qvarnstrom and Bailey 2008; Kitano and Peichel 2012), the investigation of the genetic and genomic basis of sexual development has become important also in the context of cichlid diversification. First steps in this research direction have been made with the description of several sex-chromosomal systems and possible new functions for key genes of sexual development in these fishes (Roberts et al. 2009; Ser et al. 2010; Böhne et al. 2013; Parnell and Streelman 2013).

Here, we investigated four cichlid species found in Lake Tanganyika. Based on morphological and genetic data, the cichlid assemblage of this lake can be divided into up to 16 different lineages, so-called tribes (Takahashi 2003; Koblmüller et al. 2008). We chose representatives of four tribes with different breeding behavior and with and without sexual dimorphism. More precisely, we examined *Astatotilapia burtoni* (tribe Haplochromini, maternal mouth-breeder, pronounced color, and size dimorphism), *Ophthalmotilapia ventralis* (tribe Ectodini, maternal mouth-breeder, color, and shape dimorphism), *Julidochromis ornatus* (tribe Lamprologini, substrate-breeder, and no obvious dimorphism), and *Eretmodus cyanostictus* (tribe Eretmodini, biparental mouth-breeder, and size dimorphism). The Lamprologini (∼ 80 species), Haplochromini, and Ectodini (each 30–35 species) are the species-richest tribes of Lake Tanganyika, whereas the Eretmodini tribe comprises four species only (Koblmüller et al. 2008). The SD systems of the four species are unknown, although there are indications for an XX–XY system in *A. burtoni* (Böhne A, et al., in preparation).

Here, we examined gene expression in gonads and brains, the two most important tissues in sexual development and reproduction (Vilain and McCabe 1998; Wilson and Davies 2007), to answer the questions how many and which genes differed between male and female cichlids. We were then asking how many sex-specific genes were expressed in all species and which genes showed a unique expression pattern in one species only. The two obtained sets of genes were investigated for a function in sex determination and differentiation. Additionally, we constructed a gene coexpression network using the expression data from all samples. We further tested whether sex-biased transcripts cluster together in the genome since an accumulation of sex-specific genes is a process observed in the formation of sex chromosomes (Graves 2006).

## Materials and Methods

### Tissue Samples

Research involving animals was performed under the cantonal veterinary permit no. 2317 and a research permit issued by the Lake Tanganyika Research Unit, Department of Fisheries, Mpulungu, Zambia. This study included brain and gonad RNA samples from four species endemic to Lake Tanganyika and its affluent rivers. Tissue samples for four females and four males each for *E. cyanostictus*, *J. ornatus*, and *Op. ventralis* were derived from wild-caught fish during field trips to Lake Tanganyika in 2011 and 2012 and stored directly in RNAlater (LifeTechnologies). Samples for *A. burtoni* were collected freshly prior to RNA extraction from three female and three male specimens of a laboratory strain raised at the aquarium facility at the Zoological Institute, University of Basel. Sampled individuals included only adult, mature specimen with gonad type (ovary/testis) easily distinguishable upon visual inspection suggesting active reproductive status. We chose to work on adult tissues as sexing is reliable and sexual dimorphism is most pronounced at mature stages, as is sex-biased gene expression (Mank et al. 2010; Magnusson et al. 2011; Ometto et al. 2011). RNA was extracted as previously described (Böhne et al. 2013).

### Library Preparation and Illumina Sequencing

Library construction and sequencing was conducted at the ETH Zurich Department of Biosystems Science and Engineering. The individually barcoded 60 cDNA libraries were sequenced on one flow-cell on an Illumina HiSeq2000 (randomized over seven lanes with nine to ten samples per lane, single-end sequencing with 50 cycles). Raw sequencing reads were deposited in the Sequence Read Archive under the accession number SRP042144.

### Differential Gene Expression Analysis

#### Filtering and Mapping of Reads

Sequences of each library were filtered using FASTX-toolkit version 0.0.13.2 (Gordon A, Hannon GJ, unpublished data; available from: http://hannonlab.cshl.edu/fastx_toolkit/, last accessed September 16, 2014) removing sequences with a Phred quality score below 20 in more than 4% of the nucleotide positions (settings q = 20 and p = 96%). Adaptor and rRNA sequences were removed using cutadapt version 1.2.1. (Martin 2011). Cleaned reads were mapped against the most complete cichlid cDNA set currently available, a reference cDNA from *Oreochromis niloticus* (Nile tilapia; kindly provided by BROAD; Brawand et al. 2014) consisting of 63,683 transcripts. Novoalign (Novocraft) was used for mapping with the following settings –r ALL, -t 180, -l 30, and -s 5. Alignments were reported in SAM format and sorted, indexed and transformed into count tables (number of mapped reads per transcript per sample) using SAMtools (Li et al. 2009). For details on sequencing and filtering statistics, see supplementary table S1, Supplementary Material online. In total, the data set comprised read counts for four male brain, four female brain, four ovary, and four testis samples per species except for *A. burtoni* where three biological replicates per tissue type were included.

#### Differentially Expressed Transcript Calculation

Count tables were further formatted and combined using custom Unix scripts. Count data were imported in R and differences in gene expression were analyzed using the Bioconductor edgeR package (Robinson et al. 2010). Simple pairwise comparisons between tissues were calculated using the “classic approach” (single factor design, quantile-adjusted conditional maximum likelihood, qCML, method comparing all samples of one tissue kind of one sex with all samples of the same tissue kind of the opposite sex) implemented in the edgeR package; tests with a more complex design, and including a blocking factor, were calculated using the GLM (generalized linear model) approach as implemented in edgeR (Cox–Reid, CR, profile-adjusted likelihood method to estimate tagwise dispersions for fitting negative binomial GLMs). The GLM approach was applied to the comparison between all female and all male samples (brain and gonad combined), using tissue as blocking factor, focusing on expression differences due to sex rather than tissue type. Transcripts were considered differentially expressed (DE) when showing a log_2_-fold change >1 and an FDR (false discovery rate) <0.05.

We tested whether DE transcripts expressed in one, two, three, or four species vary in their general expression level in R using custom scripts and a Kruskal–Wallis test (supplementary table S2 and fig. S1, Supplementary Material online). The R package VennDiagram (Chen and Boutros 2011) was used for visualization of transcripts with a shared expression in the investigated species. All R codes are available upon request.

### Gene Ontology Analysis

DE transcripts of interest were used in a BLASTx search (National Center for Biotechnology Information, NCBI, standalone version 2.2.27, Altschul et al. 1990) against the *nr* database run locally or online allowing for 50 output alignments per query sequence with an *e* value threshold of 0.001. Fasta files and corresponding BLAST outputs were loaded in BLAST2GO (Conesa et al. 2005). Subsequent mapping and annotation steps were run with default settings of BLAST2GO. Gene ontology (GO) graphs for the category “Biological Process” were generated with 50 sequences for the node setting and “level 2” for GO plot visualization. To identify transcripts implicated with the GO terms “sex determination” (GO:0007530) and “sex differentiation” (GO:0007548), the BLAST2GO annotation output file was searched for these two terms enabling GO ancestor search.

### Expression of Candidate Genes

We previously investigated a set of candidate genes for sex-specific expression in cichlids (Böhne et al. 2013). To test whether these genes show a similar expression pattern in the transcriptome sequencing experiment, we conducted the following analysis. Out of the 23 candidate genes with available expression data, we identified 22 in our reference cDNA set in a local BLASTn search (accession numbers for query sequences in supplementary table S3, Supplementary Material online, NCBI standalone BLAST version 2.2.28+, default settings). These genes were then checked for differences in expression between ovary and testis in each species in the output files from edgeR. The same analysis was carried out for estrogen and androgen receptors. We found six of seven described receptors (supplementary tables S3 and S12, Supplementary Material online).

### Distribution of DE Transcripts on the Nile Tilapia Linkage Groups

The Nile tilapia genome consists of 22 chromosomes. To test for colocalization of DE transcripts in the reference genome (no full genome and hence positional information is available for the here investigated species), we calculated the number of overexpressed transcripts (separately done for common, uniquely, and all overexpressed transcripts) per linkage group (LG) divided by the total number of transcripts present on that LG. Transcript location was derived from the reference transcript names that follow the scheme on.mrna.LGX.Y.Z where “on” stands for *Or. niloticus*, X for the number of the LG (the former LGs LG1–LG24 are fused in the current genome release to the actual number of 22 chromosomes with the naming LG1–LG7, LG8_24, LG9–LG15, LG16_21, LG17–LG23, information over http://www.ncbi.nlm.nih.gov/, last accessed September 16, 2014), Y for the gene number, and Z for the number of the transcript variant/isoform. For transcripts not localized on LGs, the naming follows on.mrna.UNKX.Y.Z. The transcript localization information was kindly provided by BROAD.

Values shown are in percentage of expressed transcripts per LG (fig. 5 and supplementary fig. S6 and table S4, Supplementary Material online). We tested for significant enrichment (expressed transcripts per LG compared with nonexpressed transcripts per LG) using a two-tailed Fisher’s exact test for all pairwise comparisons and corrected for multiple testing. We also tested for enrichment on LG1 and LG23, which are possible sex chromosomes in the genus *Oreochromis* (reviewed in Cnaani 2013), versus all other LGs using a two-tailed Fisher’s exact test (supplementary table S4, Supplementary Material online).

To investigate whether sequences on Nile tilapia LG1 and LG23 show a sex-specific expression pattern in the Nile tilapia itself, we downloaded the sequence identifiers for sex-enhanced transcripts from supplementary tables S6 and S7 of Tao et al. (2013). We exported the corresponding transcripts from ENSEMBL (ENSEMBL version 75) and used them as queries in a BLASTn search against our reference cDNA set with chromosomal information (NCBI standalone BLAST version 2.2.28+, *e* value <1e-5) and kept the best hit only. We then compared the hits with DE transcripts in the here-investigated cichlids.

### Characterization of Enriched Pathways

To test for enriched pathways among common DE transcripts overrepresented on LG1 and LG23, we used the web server of KOBAS2.0 (Xie et al. 2011). DE transcripts of interest (male and female transcripts DE in all four species) were blasted using a local BLASTx search (NCBI standalone BLAST version 2.2.28+, *e* value <1e-5) against the *Homo sapiens* peptide databases (most extensive database) downloaded from the KOBAS server. BLAST results in tabular format were then uploaded in KOBAS and the “annotate” step was run against the *H. sapiens* database. To test whether female overexpressed transcripts located on LG1 show a functional enrichment, the “identify” step was run using the female DE transcripts on LG1 as input and all other female DE transcripts as “background.” Similarly, to test whether male overexpressed transcripts enriched on LG23 also show a functional enrichment we tested these in the “identify” step against all other male DE transcripts as “background.” The “identify” step was run using the binomial test and Benjamini–Hochberg FDR correction. We considered only pathways with a corrected *P* value of less than 0.05 as enriched.

Pathway enrichment for GO categories in all pairwise comparisons (female vs. male and ovary vs. testis DE transcripts of the categories uniquely and commonly expressed) was run in BLAST2GO with default settings (Fisher’s exact test with correction for multiple testing) enabling for a two-tailed test and reducing the output table to the most specific terms (FDR = 0.05). We recovered all GO annotations belonging to the category Biological Process.

### Gene Expression Module Construction, Annotation, and Trait Correlation

To test for gene expression correlations, read mapping counts obtained with Novoalign for all 63,683 reference transcripts were filtered removing in a first step transcripts expressed in less than 10% of all samples and subsequently with less than 1 million reads in fewer than two samples. Filtered read counts were upper quartile normalized in edgeR and log-transformed. In case more than one transcript per gene was present in the filtered data, only the transcript with the highest variance in expression considering all samples was included for further analysis. The final data set comprised log-transformed read counts for all samples from all species for 21,120 genes. Gene expression networks were constructed using the R package WGCNA (Langfelder and Horvath 2008). Based on a topology derived from weighted correlations of gene expression across all samples (Zhang and Horvath 2005), WGCNA identifies gene modules whose members show strong coexpression over all samples of a given study. In brief, coexpression and a weighted adjacency matrix were calculated on the normalized read counts of all samples (single-block construction, soft-thresholding power set to 16 corresponding to a mean connectivity of ∼277 and a median connectivity of ∼87 genes; supplementary fig. S5, Supplementary Material online) followed by transformation of the adjacency into a topological overlap matrix and hierarchical clustering of the matrix into a dendrogram of gene coexpression. Coexpression modules were identified as clusters from the dendrogram using the cutreeDynamic function of the R package dynamicTreeCut (Langfelder et al. 2008) implemented in WGCNA with a minimum module size of 30 genes. Coexpression similarity of entire modules was quantified by calculating the module eigengenes and clustering of the correlation of eigengenes in WGCNA. Modules with very similar coexpression were subsequently merged with a height cutoff of 0.1 corresponding to a connectivity correlation of 0.9 (supplementary fig. S5, Supplementary Material online). Importance or function of a module was further assessed by relating modules to the traits “sex,” “tissue type,” and “species” using an analysis of variance (ANOVA).

Extending the analysis beyond a simple GO assignment and aiming for a more unbiased approach, genes belonging to modules were annotated using an in-house pipeline based on a staged BLASTx search against the swissprot, refseq_vertebrate, and nr databases (*e* value cutoff 1e-20, identity cutoff 50%). The GeneIDs for hits matching quality criteria were retrieved using UniProt’s up_idmapping cross-reference table (ftp://ftp.uniprot.org/pub/databases/uniprot/current_release/knowledgebase/idmapping/, last accessed September 16, 2014). Annotation for individual GeneIDs was retrieved from NCBI’s gene_info table (ftp://ftp.ncbi.nlm.nih.gov/gene/DATA/, last accessed September 16, 2014). Validation of the gene annotation is supported through use of the HomoloGene (ftp://ftp.ncbi.nih.gov/pub/HomoloGene/, last accessed September 16, 2014) and subsequent verification that matches from multiple species fall in the same HomoloGene cluster. Unannotated transcripts of interest were manually searched in BLASTx results with looser filtering criteria. The pipeline, largely based on SQL table manipulations, will be made publicly available (Sengstag T, et al., in preparation). Full annotation tables and R codes are available upon request.

## Results

### Aligning Reads to a Common Reference

For three of the investigated species (*Op. ventralis, J. ornatus*, and *E. cyanostictus*), no reference genome is available and the genome sequence of *A. burtoni* is still in a nonannotated draft version. We thus mapped reads of all sequencing libraries individually against a reference cDNA set from the Nile tilapia (Brawand et al. 2014). With over 60,000 transcripts, it represents the most comprehensive transcriptome available for any cichlid. This strategy reduced bias in sequence alignment, as the Nile tilapia is an outgroup to the East African cichlid clades and hence equidistant to all of them. From the cleaned reads, 65–71% (supplementary table S1, Supplementary Material online) aligned to the reference transcriptome, providing a solid basis for differential gene expression analysis and being comparable to alignment statistics using Nile tilapia reads against a Nile tilapia reference (Tao et al. 2013).

In a next step, we were asking which transcripts are overexpressed in the male and female sex in the two tissue types that were subject to RNA-sequencing: Brain and gonads.

### Low Degree of Differential Expression in the Brain

**Fig. 1.—:**
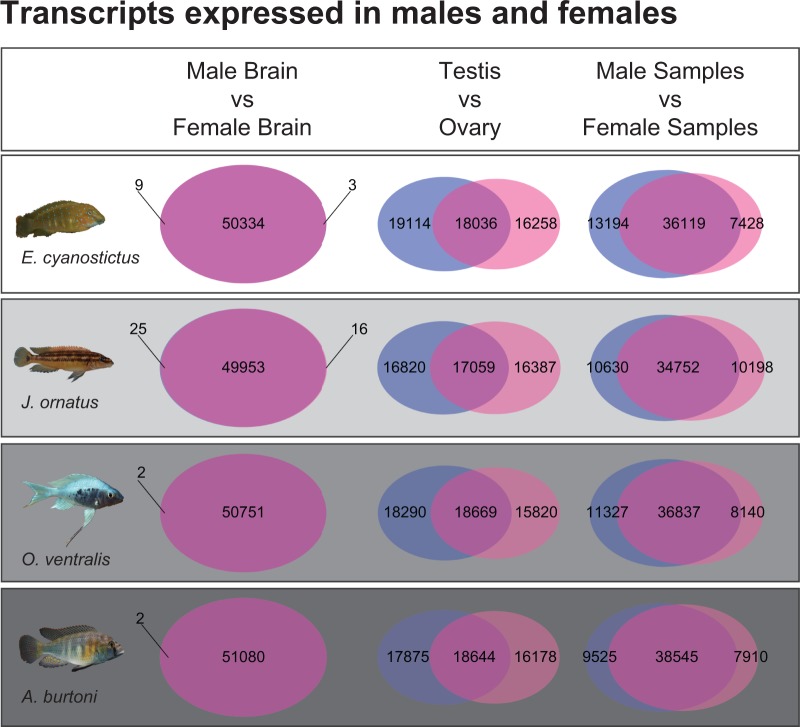
Transcripts expressed in male and female cichlids. Three comparisons are shown for the four investigated species (depicted on the left). Numbers in the overlap between circles indicate non-DE transcripts. Few differences were detected between male and female brains. The comparison between ovaries and testes of all four species shows the largest number of DE transcripts.

We detected few DE transcripts (logFC, log_2_-fold change, >1 with FDR <0.05) between male and female brain tissue, a pattern consistent for all four species (fig. 1). The rare DE transcripts (table 1) belong to genes known to be active in the brain, such as the neuropeptide isotocin, which influences social behavior. *Isotocin* was overexpressed in female brain tissue of *E. cyanostictus* and in male brain tissue of *A. burtoni*.

**Table 1:** Differentially expressed genes between Male and Female Brains

Species	Overexpressed in Females	LogFC^a^ in Expression	Overexpressed in Males	LogFC in Expression
*Eretmodus cyanostictus*	Isotocin (neuropeptide influencing social behavior, two transcripts)	1.9	FAD-dependent oxidoreductase domain-containing protein 1 (mitochondrial chaperon, one transcript)	1.2
	Polinton (transposable element, one transcript)	2.6	Hepatoma-derived growth factor-related protein 2 (three transcripts)	1.1
			Pol-poly protein (Gypsy retroelement, four transcripts)	3.1
			Uncharacterized protein (one transcript)	8.1
*Julidochromis ornatus*	Cadherin-7 (cell adhesion, six transcripts)	2.1	Myosin regulatory light chain 2 (one transcript)	5.4
	Brain aromatase (converts androgens to estrogens, six transcripts)	1.0	Myosin heavy chain (one transcript)	6.0
			Kelch like protein 41 (muscle development and differentiation, one transcript)	7.7
	Class I histocompatibility antigen, F10 alpha chain (presentation of foreign antigens to the immune system, three transcripts of one gene copy, one transcript of another gene copy)	1.04 and 1.02	Calponin homology domain-containing protein/aspartyl/asparaginyl beta-hydroxylase (12 transcripts)	1.06
			Alpha-actin (two transcripts)	6.9
			Angiopoietin-related protein 7 (vascular growth factor, two transcripts)	3.0
			Troponin 1 (one transcript)	3.8
			Troponin T (five transcripts)	4.5
*Ophthalmotilapia ventralis*	—		S100-A1-like (calcium-binding protein, two transcripts)	5.2
*Astatotilapia burtoni*	—		Isotocin (neuropeptide influencing social behavior, two transcripts)	1.9

^a^If more than one transcript per gene is differently expressed, the mean logFC is given.

Another DE neuronal factor is the calcium-binding protein *S100A1*, which binds to synapsins, and is, together with them, involved in the regulation of synaptic vesicle trafficking and neurotransmitter release (Heierhorst et al. 1999; Benfenati et al. 2004), here overexpressed in male brain tissue of *Op. ventralis*.

We also detected overexpression of *HGFRP2* in male brain tissue of *E. cyanostictus*. *HGFRP2* belongs to the hepatoma-derived growth factor family, which is expressed in the central nervous system, where different family members are specific to certain brain regions (El-Tahir et al. 2006).

In *J. ornatus*, we detected female-specific overexpression of the brain aromatase, an enzyme converting androgens into estrogens, a crucial part of the sexual development cascade.

In *E. cyanostictus* brain, each sex overexpressed a different class of transposable elements: A polinton DNA transposon in females and a gypsy retrotransposon in males. Active transposable elements in the brain have been associated with aging and neuronal decline (Coufal et al. 2009; Li et al. 2013).

### DE Transcripts in the Gonads

The comparison between the female and male gonad samples revealed a substantially larger number of DE transcripts (fig. 1; 15,820–16,387 ovary and 16,820–19,114 testis overexpressed transcripts) than in brain tissue. We also obtained high numbers of DE transcripts when comparing all female with all male samples (brain and gonad samples combined, 7,428–9,635 female and 9,525–13,194 male overexpressed transcripts). In the two comparisons, ovary vs. testis and all female vs. all male samples, we found more testis/male overexpressed transcripts than ovary/female overexpressed transcripts in all four species.

### Common and Unique Transcripts in Males and Females

**Fig. 2.—:**
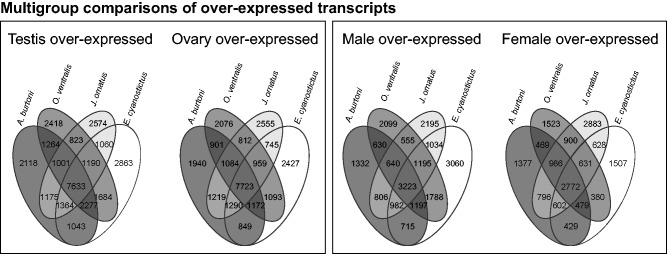
Multigroup comparisons of overexpressed transcripts. The graphic shows the numbers of shared transcripts between species within the four groups of DE transcripts (testis overexpressed, ovary overexpressed, female overexpressed, male overexpressed).

To shed more light on the gene sets overexpressed in each gonad type and in each sex in general, we tested which transcripts are shared between species and which are species-specific. To this end, we compared all overexpressed transcripts of each pairwise test (ovary vs. testis and all female samples vs. all male samples) between the four species, leading to four groups, testis, ovary, male, and female overexpressed transcripts (fig. 2). Transcripts that were commonly overexpressed in all four species in testis or ovary made up the biggest fraction of overexpressed transcripts within a species (39.93–45.38% for testis transcripts and 47.13–48.82% for ovary transcripts). Species-specific transcripts, on the other hand, made up the smallest fraction in testis and ovary (11.85–15.30% for testis and 11.99–15.59% for ovary). The same pattern was true for pooled male samples in *A. burtoni, Op. ventralis* and *J. ornatus* as well as female samples in *A. burtoni* and *Op. ventralis*. There was no clear trend in pooled female samples in *E. cyanostictus* and pooled male samples of *J. ornatus* (all values in supplementary table S5, Supplementary Material online).

Next, we compared the expression levels of transcripts shared between four, three, and two species with uniquely expressed transcripts per species (supplementary fig. S1, Supplementary Material online). In all groups of overexpression (ovary, testis, female samples, and male samples), transcripts shared between more species showed a trend toward higher/more differential expression (overall expression level: Unique < expressed in at least two species < expressed in at least three species < expressed in all four species).

In the following analyses steps we focused on two particular groups, the transcripts that were DE in all four species (common/shared transcripts) and transcripts that were only overexpressed in one species (unique transcripts). To gain more insight into these transcript groups, we annotated them according to GO terms (Conesa et al. 2005).

### GO Annotation of Shared and Unique DE Transcripts

**Fig. 3.—:**
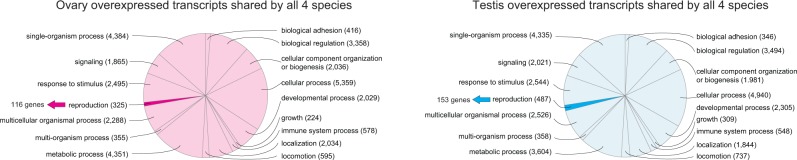
Characterization of transcripts with sex-specific expression in all species. GO annotation (Biological Process categories with at least 50 transcripts each and belonging to the highest annotation level, ontology level 2 in BLAST2GO) for ovary/testis-specific transcripts overexpressed in all species. The transcripts belonging to the GO terms sex determination and sex differentiation all belong to the ancestral GO term reproduction, depicted by colored arrows and actual gene numbers.

Using BLAST2GO we annotated the DE transcripts in the groups “unique” and “shared between all species.” Figure 3 shows the major GO categories for Biological Process (highest ontology level in BLAST2GO) for the common DE transcripts in ovary and testis (similar distributions were seen for the unique transcripts and the common and unique transcripts of the groups male samples and female samples; supplementary figs. S2–S4, Supplementary Material online). In both groups, ovary and testis, the largest fraction of transcripts was grouped in the category “cellular process,” followed by “single-organism process,” “metabolic process,” and “biological regulation” (all over 3,000 transcripts). The GO terms “response to stimulus,” “multicellular organismal process,” “developmental process,” “signaling,” and “cellular component organization or biogenesis” still all grouped around 2,000 transcripts.

With the aim to identify genes that play a role in sex determination and differentiation, we searched all data sets for these two GO categories. Note that the GO category “reproduction” (highlighted in fig. 3) is the GO ancestor to all transcripts detected in this way. In the following, we discuss the results for the tests ovaries versus testis and all male samples versus female samples together, since the obtained DE transcripts largely overlapped.

### Influencing the Female Fate—Candidate Genes for a Common Female Genetic Network in Cichlids

Among the ovary/female overexpressed transcripts common to all four species, we found 28 genes belonging to the GO terms sex determination and sex differentiation (table 2 and for details on all expression values see supplementary table S6, Supplementary Material online).

**Table 2:** Genes Belonging to the GO Categories Sex Determination (det) and Sex Differentiation (diff) Overexpressed in Ovaries/Female Tissues in All Four Investigated Species

Ovary/Female Overexpressed Genes in All Four Species	GO Term	Overexpressed In
Apoptosis regulator bax (accelerates programmed cell death by binding to, and antagonizing the apoptosis repressor bcl2)^a^	Sex diff	Ovary
Bcl-2-related ovarian killer protein (induces apoptosis)	Sex diff	Ovary/females
Beta-*n*-acetylglucosaminyltransferase lunatic fringe-like (initiates the elongation of O-linked fucose residues attached to EGF-like repeats in the extracellular domain of Notch molecules)	Sex diff	Ovary/females
Bone morphogenetic protein receptor type-1a (receptor for BMP-2 and BMP-4, regulates transcription)	Sex diff	Ovary
Bone morphogenetic protein 15 (TGF-beta family member, oocyte-specific growth/differentiation stimulating folliculogenesis and granulosa cell growth)	Sex diff	Ovary
Cbp p300-interacting transactivator 3-like (stimulates estrogen-dependent transactivation activity, positively regulates TGF-beta signaling)	Sex det/diff	Ovary/females
Doublesex- and mab-3-related transcription factor 2 (dm-family, could be implicated with gonadal dysgenesis and XY sex reversal in humans)	Sex det/diff	Ovary/females
Ensconsin (microtubule-stabilizing protein)	Sex diff	Ovary/females
Forkhead box l2 (transcriptional regulator, in mammals critical factor essential for ovary differentiation and maintenance, and repression of the genetic program for somatic testis determination)	Sex det/diff	Ovary
Forkhead box protein o3 (transcriptional activator, triggers apoptosis)	Sex diff	Ovary/females
Frizzled-10 (G protein-coupled Wnt receptor, positive regulator of the Wnt/beta-catenin pathway)	Sex diff	Ovary/females
g1 s-specific cyclin-d1 (regulation of cell-cycle during G_1_/S transition) two gene copies OE in ovary	Sex diff	Ovary/females
Hepatocyte growth factor receptor (receptor tyrosine kinase, transduces signals from the extracellular matrix into the cytoplasm, regulates many physiological processes)	Sex diff	Ovary
Inhibitor of growth protein 2 (possibly involved in apoptotic pathways)	Sex diff	Ovary/females
Mothers against decapentaplegic homolog 5 (transcriptional modulator activated by BMP type 1 receptor kinase)	Sex diff	Ovary
Mothers against decapentaplegic homolog 9 (transcriptional modulator activated by BMP type 1 receptor kinase)	Sex diff	Ovary/females
Peroxisomal multifunctional enzyme type 2 (peroxisomal beta-oxidation pathway for fatty acids)	Sex diff	Ovary
Prohibitin (inhibits DNA synthesis, regulates proliferation)	Sex diff	Ovary
Ribonucleoside-diphosphate reductase large subunit (DNA synthesis)	Sex diff	Ovary/females
Sal-like protein 1 (zinc-finger transcription factor, transcriptional repressor involved in organogenesis)	Sex diff	Ovary
Secreted frizzled-related protein 2 (modulator of Wnt signaling)	Sex diff	Ovary
Secreted frizzled-related protein 3 (modulator of Wnt signaling)	Sex diff	Ovary/females
Sox3-like (transcription factor required during the formation of the hypothalamo-pituitary axis, male sex determiner in mammals)	Sex det/diff	Ovary/females
Tcdd-inducible poly [ADP-ribose] polymerase (possibly role in the adaptive response to chemical exposure, response to DNA strand breaks)	Sex diff	Ovary
Transcription factor ap-2 gamma (DNA-binding protein interacting with inducible viral and cellular enhancer elements to regulate transcription)	Sex diff	Ovary/females
Tyrosine-protein phosphatase nonreceptor type 11 (acts downstream in signal transduction from cell surface to nucleus)	Sex diff	Ovary/females
Wnt-5a (ligand for members of the frizzled receptors, activates or inhibits canonical Wnt-signaling, depending on receptor context)	Sex diff	Ovary/females

^a^Functions taken from http://www.uniprot.org

These genes present a starting point for the definition of a common molecular network of female sex determination and differentiation in cichlids and possibly fishes in general. They include prominent examples known to be implicated in sexual development in other species, such as members of the forkhead box family (here *foxl2* and *foxo3),* the sox gene family (here *sox3*), and the dm domain gene family here (*dmrt2)*. *Foxl2* is the critical factor repressing the male developmental program and thus promoting ovary development in mammals (Ottolenghi et al. 2005); and it is crucial for maintenance and functioning of the adult ovary (Uhlenhaut et al. 2009). A similar role for *foxl2* has been suggested in the Nile tilapia (Wang et al. 2007). F*oxo3* has been implicated with the induction of apoptosis.

The function of sox3, the possible ancestor of the mammalian SD gene *sry* (Sato et al. 2010), is less clear, although it has been implicated with the induction of testis formation (Sutton et al. 2011), progression of spermatogenesis (Raverot et al. 2005), and formation of the hypothalamus–pituitary axis (Rizzoti et al. 2004). In the rice fish *Oryzias dancena, sox3* has been co-opted as the male SD gene (Takehana et al. 2014), whereas in the frog *Rana rugosa sox3* determines the female fate (Oshima et al. 2009). In addition, *sox3* has been implicated with oocyte development (Weiss et al. 2003) and promotion of oogenesis in the grouper (Zhou and Gui 2010). *Sox3* could have a similar female-promoting function in cichlids, as it showed one of the highest levels of overexpression in the ovary compared with testis, only being topped by the expression levels of *bone morphogenetic protein 1, bmp1* (supplementary table S6, Supplementary Material online). *Sox3* might hence have a female- or male-specific function, depending on the species investigated.

Also bmp proteins, bmp receptors, and their interaction partners were overexpressed in all females/ovaries.

*Dmrt2* was highly overexpressed in all female/ovary samples in our experiments, and has been implicated with sex determination before (Matsushita et al. 2007; Sheng et al. 2014).

Several of the shared overexpressed genes are members of the Wnt-signaling pathway that has been implicated with female reproductive development in mammals (Bernard and Harley 2007) and also in some fish species (Wu and Chang 2009). However, a female-specific function of the Wnt pathway does not seem to be a general pattern in fishes (Nicol et al. 2012).

Another predominant function of several detected genes is their involvement in apoptosis. In the vertebrate ovary, the apoptotic process of follicular atresia degenerates ovarian follicles that will not complete maturation but rather be reabsorbed (Morais et al. 2012, and references therein).

### Influencing the Male Fate—Candidate Genes for a Common Male Genetic Network in Cichlids

Among the commonly overexpressed male/testis transcripts, we found 21 genes belonging to the GO terms sex differentiation/determination (table 3 and for details on all expression values see supplementary table S7, Supplementary Material online). Members of the Wnt-signaling cascade were overexpressed also in testis, again including members of the frizzled-family. This pathway might thus play a role in gonad functioning in both sexes, although with different genes being implicated in each sex.

**Table 3:** Genes Belonging to the GO Categories Sex Determination (det) and Sex Differentiation Overexpressed in Testis/Male Tissues in All Four Investigated Species

Testis/Male Overexpressed Genes in All Four Species	GO Term	Overexpressed In
Follicle stimulating hormone receptor (G-protein coupled receptors, functions in gonad development)	Sex diff	Testis
Frizzled-6 (receptor for Wnt4)	Sex diff	Testis/males
Frizzled-7 (receptor for Wnt proteins)	Sex diff	Testis
High mobility group protein b2 (here two gene copies expressed in testis, DNA-binding protein that associates with chromatin and has the ability to bend DNA)	Sex diff	Testis/males
Histone (DNA packaging)	Sex diff	Testis
Protein jagged-2 (putative Notch ligand involved in the mediation of Notch signaling)	Sex diff	Testis/males
Kelch-like protein 10 (here two gene copies, substrate-specific adapter of a CUL3-based E3 ubiquitin-protein ligase complex which mediates the ubiquitination and subsequent proteasomal degradation of target proteins during spermatogenesis)	Sex diff	Testis/males
Long-chain-fatty-acid-ligase acsbg2 (mediates activation of long-chain fatty acids, may play a role in spermatogenesis)	Sex diff	Males
Mast stem cell growth factor receptor kit (tyrosine-protein kinase)	Sex diff	Testis
Meiotic recombination protein dmc1 lim15 homolog (may participate in meiotic recombination)	Sex diff	Testis/males
Mitogen-activated protein kinase kinase kinase 4 (serine/threonine kinase that may play a role in the response to environmental stress and cytokines)	Sex det	Testis
Nipped-b-like protein (probably plays a structural role in chromatin)	Sex diff	Testis
Nuclear receptor coactivator 2 (transcriptional coactivator for steroid receptors and nuclear receptors)	Sex diff	Testis/males
Polyadenylate-binding protein 2 (involved in the 3′-end formation of pre-mRNA by the addition of a poly(A) tail)	Sex diff	Testis
Retinol dehydrogenase 10 (converts all-trans-retinol to all-trans-retinal)	Sex diff	Testis
tilb homolog (may play a role in dynein arm assembly)	Sex diff	Testis/males
Transcription factor 7-like 2-like (HMG-box transcription factor that participates in the Wnt signaling pathway, two gene copies)	Sex diff	Testis/males
Transforming growth factor beta-2 (cytokine)	Sex diff	Testis

The most highly expressed testes gene common to the four species was *kelch-like protein 10* (with two different gene copies), which plays a role in spermiogenesis (Yan et al. 2004).

We also detected testis overexpression of two other prominent gene families that have already been implicated with sex determination either in mammals, namely the HMG-family of transcription factors (Koopman 2001) (here, *high mobility group protein b2*), or in fish, the TGF-β-superfamily (here, *transforming growth factor beta-2*). Especially the TGF-β-superfamily received a lot of attention recently as three new master SD genes, *amhy*, *amhr2*, and *gsdf*, identified in fish belong to this family of nontranscription factors (for a review, see Heule et al. 2014). Note that in the female/ovary common transcripts this family was also represented, with the bmp proteins, receptors, and activators. *Amhr2* was also overexpressed in testis in all four investigated species. *Amh* was overexpressed in testis of *A. burtoni* and *E. cyanostictus* but was not DE in *Op. ventralis* and *J. ornatus* (see below). *Gsdf* was overexpressed in the testis of all species but *A. burtoni,* where it was overexpressed in the ovary.

### Enriched Gene Expression Pathways in Males and Females

To confirm the importance of the above-described gene families and to detect further pathways with a possible link to sex determination and differentiation, we ran GO pathway enrichment analysis comparing all GO representations in ovary overexpressed transcripts against the testis overexpressed transcripts and the same for female against male sample overexpressed transcripts. We did so for the commonly and uniquely expressed transcripts (supplementary tables S8 and S9, Supplementary Material online) and focused again on GOs of the category Biological Process. For the commonly expressed transcripts we found, out of a total of 5,326 GO annotations, enrichment for 268 GOs in ovary and 422 in testis and out of a total of 3,428 GO annotations, 138 enriched in female tissue (73 shared with ovary) and 273 in male tissue (149 shared with testis). Although these GO terms cover a large variety of physiological processes such as lipid metabolism, cytoskeletal changes, or mitochondrial metabolism, several terms directly implicated in sex determination/differentiation were indeed enriched. These included different transcription factor groups and general transcriptional regulation as well as apoptotic processes.

The screening for the GO terms sex determination and sex differentiation described above strongly suggested an implication of the Wnt signaling pathway as well as members of the TGF-β-superfamily in sex differentiation in cichlids. We could confirm these findings with the enrichment tests. For the Wnt pathway, the GO terms “positive regulation of canonical Wnt receptor signaling pathway” and “regulation of Wnt receptor signaling pathway, planar cell polarity pathway” were enriched in ovary and female tissue, “positive regulation of noncanonical Wnt receptor signaling pathway” and “regulation of Wnt receptor signaling pathway involved in dorsal/ventral axis specification” were enriched in female tissue. We also detected enrichment related to the Wnt cascade in male tissue/testis with the GO terms “Wnt receptor signaling pathway involved in digestive tract morphogenesis” enriched in testis and male tissue, “canonical Wnt receptor signaling pathway involved in positive regulation of epithelial to mesenchymal transition” in male tissue, and “negative regulation of canonical Wnt receptor signaling pathway” in testis.

Among the uniquely expressed transcripts, GO terms belonging to the Wnt pathway were enriched; for *A. burtoni* and *Op. ventralis* only in female tissue/ovaries, in *E. cyanostictus* in testis and male tissue, and in *J. ornatus* in ovary (see pink marked lines in supplementary tables S8 and S9, Supplementary Material online).

Also the TGF-β-superfamily, already detected with the search for GOs sex determination/differentiation, figured among the enriched pathways with “negative regulation of TGF-β receptor signaling pathway” in male tissue (marked green lines in supplementary tables S8 and S9, Supplementary Material online). Further GO pathways comprising members of the TGF-β-superfamily included the above-discussed BMPs with “negative regulation of BMP signaling pathway” enriched in testis/male tissue. We could also detect enrichment of GO terms linked to apoptosis, mentioned above as a process happening frequently in the gonad (marked in red in supplementary tables S8 and S9, Supplementary Material online).

Further newly detected interesting and obvious GO groups in the context of sex determination/differentiation were the ovary-enriched pathways “female gonad development,” “negative regulation of reproductive process,” “negative regulation of androgen receptor signaling pathway” and “androgen metabolic process” as well as the testis-enriched pathways “male germ-line sex determination,” “response to progesterone stimulus” and “response to estradiol stimulus,” and the male-enriched pathway “response to steroid hormone stimulus” (for genes found in these groups, refer to supplementary tables S8 and S9, Supplementary Material online).

### Species-Specific Overexpressed Genes in Males and Females

Several members of the Wnt-signaling cascade including ligands, receptors, and regulators showed distinct species-specific patterns (supplementary tables S8–S10, Supplementary Material online, members of the Wnt-pathway are marked in pale rose). For example, several frizzled receptors and frizzled-proteins were overexpressed in the testis of *E. cyanostictus* and in the ovary of *A. burtoni*. Two different gene copies of *wnt2b* were overexpressed in the ovary and testis of *J. ornatus*, respectively.

Also several members of the TGF-β-superfamily were expressed in a species- and tissue-specific manner, again including ligands and receptors, and especially inhibins and activins and their receptors. The inhibin/activin subgroup of the TGF-β-superfamily inhibits and activates, respectively, the secretion of gonadal and pituitary hormones, thereby influencing germ cell development and the hypothalamus–pituitary–gonadal axis (e.g., Bilezikjian et al. 2004). Note that this subgroup did not appear in the analysis of common transcripts. The *A. burtoni* ovary/female tissue overexpressed transcripts were enriched for the GO term “negative regulation of activin receptor signaling pathway” and the *J. ornatus* testis overexpressed transcripts for “regulation of activin receptor signaling pathway.”

Species-specific overexpression was also found for representatives of the bmp proteins and receptors, another subgroup of the TGF-β-superfamily with GO enrichment for “regulation of BMP signaling pathway” in *E. cyanostictus* male tissue.

Enigmatic members already implicated in sex determination in vertebrates, and also recovered in our study, included the testis factor *sox9* (Kobayashi et al. 2005) (here *sox9a* in testis of *A. burtoni*, and another isoform of *sox9a* as well as *sox9b* in the testis/male tissues of *E. cyanostictus*), suggesting a male-specific function also in cichlids. Another such factor is *amh*, the anti-Mullerian hormone, responsible for the regression of Mullerian ducts in mammals (Rey et al. 2003). This gene is known to be the master SD gene in the Patagonian pejerrey (Hattori et al. 2012), as might be *amhr2*, the *amh* receptor, in pufferfishes (Kamiya et al. 2012). Different isoforms of *amh* were overexpressed in the testis of *A. burtoni* and *E. cyanostictus*. However, in *J. ornatus*, *amh* is overexpressed in the ovary and the GO category “Mullerian duct regression” is enriched in female tissue in all four species, asking for further investigation of the action of this hormone in cichlids.

In *J. ornatus*, two more genes showed opposing expression patterns compared with their published function, namely *steroidogenic factor 1* (here overexpressed in ovary, but otherwise known to be important for male development in humans; Achermann et al. 1999) and *factor in the germline alpha* (here in testis, known as oocyte-specific gene in mammals; Huntriss et al. 2002).

With *dmrt3* yet another member of the dm-domain family might play a role in sex determination in cichlids, here it was the gene with the highest level of overexpression in the testis of *Op. ventralis* (supplementary table S10, Supplementary Material online).

Other genes with a species-specific expression pattern also included further members of the steroid pathway and a variety of transcription factors (supplementary table S10, Supplementary Material online, marked in gray).

Our approach revealed that genes/transcripts, which are overexpressed in the female gonad in one species, can be overexpressed in the male gonad in another species. This was especially pronounced for several genes expressed in the testis of *Op. ventralis* including *cytosolic phospholipase a2*, *dachshund 2*, and receptor-encoding genes, which were in the other species expressed in the ovary. Also several genes expressed in the testis of *E. cyanostictus* were expressed in the ovary of *J. ornatus* (*sphingosine-1-phosphate lyase 1*, *transcription factor 21*, *bmp7*, *irx-5*). We observed similar patterns in a previous gene candidate study (Böhne et al. 2013) and, hence, aimed to compare the two data sets.

### Expression of Candidate Genes in Ovaries and Testis

In a previous study, we investigated the expression of candidate genes with a supposedly conserved role in sexual development, gonad function, and maintenance in a subset of East African cichlid fishes, including *A. burtoni* and *Op. ventralis,* by quantitative polymerase chain reaction (qPCR) (Böhne et al. 2013). This study revealed new expression patterns in adult testis and ovary in derived cichlid lineages (Haplochromini and Ectodini) for core genes of vertebrate sexual development, such as the aromatase *cyp19a1* and *sox9*. We were asking whether these shifts in expression were consistent in this study and extended the gene candidate data to *J. ornatus* and *E. cyanostictus*. To this end, we first screened the reference transcripts of *Or. niloticus* for the presence of the candidate genes described in Böhne et al. (2013) in a local BLAST search. All genes but *ctnnb1B* were present in the reference transcriptome. We then looked for expression of the corresponding transcripts in the pairwise comparison ovary versus testis in each species (supplementary table S11, Supplementary Material online). The RNA-sequencing data were largely congruent with the qPCR data from Böhne et al., especially with regard to the aromatase genes *cyp19a1A* and *cyp19a1B*. We thus confirmed our previous finding that the otherwise brain-specific aromatase *cyp19a1B* is overexpressed in the testis of *A. burtoni*, whereas the other copy, *cyp19a1A*, a gene highly expressed in the ovary in all fish species studied so far, is overexpressed in testis in *Op. ventralis*. Both, *cyp19a1A* and *cyp19a1B*, were overexpressed in the ovary of the Lamprologine *J. ornatus*, resembling the expression pattern of *Neolamprologus pulcher*, the lamprologine studied in Böhne et al. (2013). Also the expression patterns of the other candidate genes in *J. ornatus* resembled the Q-PCR patterns observed in *N. pulcher*. A representative of the Eretmodini, the tribe that *E. cyanostictus* belongs to, was not included in our previous study. This species showed the ancestral pattern for *cyp19a1A* in the ovary.

### Expression of Steroid Hormone Receptors

The shifts in expression of *cyp19a1A* and *B* raised the question if this has functional consequences. RNA-sequencing does not directly reveal functional aspects but the present data set allowed us to study other members of the steroid hormone pathway. More specifically, we were asking whether an increase of aromatase in the testis correlates with estrogen receptor expression, indicating that the enzymes indeed increase the level of estrogen in the testis of these fishes. For this, we conducted the same analysis for the estrogen and androgen receptors as we did for the candidate genes (supplementary table S12, Supplementary Material online). The Ectodini *Op. ventralis,* which overexpressed the “ovary” aromatase *cyp19a1A* in the testis, also had one estrogen receptor overexpressed in the testis, namely *esr2b.* The other three species overexpressed *esr2b* in the ovary or not at all. *Astatotilapia burtoni,* which overexpressed the “brain” aromatase *cyp19a1B* in the testis, also overexpressed one estrogen receptor in the testis, *esr1*. Again, the other species overexpressed *esr1* in the ovary or had no differential expression. These data support a species-dependent estrogen-signaling pathway usage in cichlids. The correlation between the expression of synthesizing-enzymes of hormones and their receptors could indicate a new function for estrogen in the testis of adult cichlids.

To complete the picture, we now included in our analyses, for the first time in several cichlid species, the two copies of the androgen receptor present in teleost fishes (Douard et al. 2008). *ArA* was overexpressed in the testis of *A. burtoni*, *Op. ventralis*, and *E. cyanostictus*; *arB* was overexpressed in the ovary of *Op. ventralis* and *J. ornatus*. This suggests a tissue subfunctionalization of the two copies at least in *Op. ventralis*.

### Gene Coexpression Network Analysis

**Fig. 4.—:**
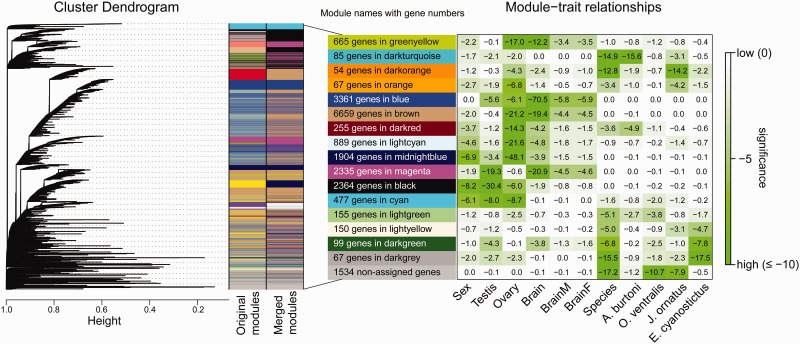
Gene expression modules constructed by WGCNA. On the left, the clustering dendrogram of genes expressed in the four cichlid species is shown. WGCNA identifies gene modules using the cutreeDynamic function, which detects clusters in a dendrogram depending on their shape (Langfelder et al. 2008). Original modules of very similar coexpression are merged. Color name-based module labels are generated automatically by WGCNA and are shown along with the number of genes belonging to each module. On the right, association between modules and traits (such as tissue type, sex, and species) based on ANOVA testing is depicted. Rows correspond to module eigengenes (shown on the left with the colored boxes), columns to a trait (indicated below each column). Each cell contains the log_10_-transformed *P* value of the trait–module association; large negative values indicate a strong association.

To analyze relationships between genes throughout the entire data set we conducted a weighted gene coexpression network analysis (WGCNA), which clusters genes based on their expression in all investigated samples into coexpression modules. The analysis resulted in 16 such modules (fig. 4) based on expression for 21,120 genes, with 7.3% of genes not assigned to a module. WGCNA automatically assigns color names to modules that we decided to keep (all 16 color names are indicated in fig. 4). Note that with the chosen minimal module size of 30 genes, unassigned genes might still belong to smaller expression pathways.

After module building, we tested for an association of the obtained modules with the traits “tissue” (ovary, testis, male brain, and female brain), “sex,” and “species,” using an ANOVA. Given the nature of the data set, several modules had indeed a significant correlation with the trait sex, especially the modules “black,” “cyan,” and “midnightblue.” The large modules (numbers of genes per module are given in fig. 4) “black,” “cyan” and “blue” were all significantly associated with the two gonad types, “magenta” with testis and brain and “greenyellow” with ovary and brain.

The majority of genes previously identified in the GO categories sex determination and sex differentiation and DE in all four species (Tables 2 and 3) belong to the modules “black,” “brown” (highly associated with ovary and brain), and “midnightblue” (highest association with ovary) (for details, see supplementary table S13, Supplementary Material online). Among the testis/male-specific genes, two genes of the Wnt-pathway were the only ones belonging to the module cyan.

The smaller modules (“darkturquoise,” “darkorange,” “darkgray,” and “darkgreen”) were significantly associated with a particular species or species-combination and will be discussed here.

The darkorange module was associated with *J. ornatus* and hence contained genes that differ between this substrate spawner and the three mouth-breeding species. This module contained mainly genes that were overexpressed in the testis of the mouth-breeding species but were not expressed in the gonads of *J. ornatus,* and three genes overexpressed in the ovary of *J. ornatus* (supplementary table S14, Supplementary Material online). The testis-overexpressed genes contained several transposable element-related sequences and genes acting in the immune response, suggesting that transposable elements as well as immune system-related factors are particularly active in males of the mouth-breeding species but not in *J. ornatus*.

The darkturquoise module showed a high association with *A. burtoni*, which is the most derived species of the investigated cichlids. Most of the genes in this group were overexpressed in the testis of *A. burtoni* and also contained several transposable element/virus-related sequences.

Both, the darkgreen module and the darkgray module are associated with *E. cyanostictus*. Darkgray contained almost exclusively genes that were overexpressed in the testis of *E. cyanostictus* but were not expressed in the other species. Darkgray might hence be a truly species-specific module with a male-specific expression pattern. The expression pattern in the darkgreen module was not as pronounced as in the darkgray module. However, it contained a large fraction of genes overexpressed in the ovary of *E. cyanostictus* and with opposing expression patterns in the other three species.

Finally, we traced the placement of the afore-mentioned candidate genes in the network modules (supplementary table S15, Supplementary Material online). Most of them also belonged to the large modules black, blue, brown, and magenta just like the genes DE in all four species. Interestingly, the different homologs *arA* and *B, cyp19a1A* and *B,* and *wnt4A* and *B* were placed in different modules, suggesting a functional difference of these duplicate genes whereas *sox9A* and *B,* for example, belonged to the same module.

### Distribution of Shared and Unique Transcripts in the Nile Tilapia Genome

Sex chromosomes are often enriched for genes with a sex-specific function reflected by sex-specific expression (e.g., Arunkumar et al. 2009; Assis et al. 2012). The master regulators of sex in Lake Tanganyika cichlids are unknown and so far no sex chromosomes have been identified in any of the species. To the best of our knowledge, no karyotype information is available for *J. ornatus*, *E. cyanostictus,* or *Op. ventralis*. However, cichlid genomes are largely syntenic and chromosome number in African species is rather conserved (predominantly 2*n* = 44; Poletto et al. 2010), suggesting that the content of large genomic regions is similar in the investigated species. The karyotype of *A. burtoni* is 2*n* = 40 with two fused chromosomes but overall large syntenic chromosomal blocks compared with *Or. niloticus* (Mazzuchelli et al. 2012).

The Nile tilapia has a male SD system (Baroiller, D’Cotta, Bezault, et al. 2009) and two LGs have repeatedly been identified as being sex-associated in this species, LG1 and LG23 (reviewed in Cnaani 2013).

**Fig. 5.—:**
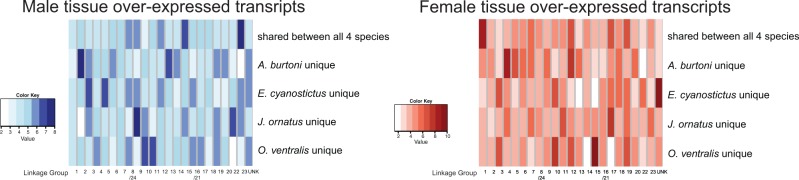
Distribution of DE transcripts in the Nile tilapia genome. Transcripts with a sex-specific expression in all species in the test male tissue versus female tissue and also with species-specific expression are grouped according to their location in the Nile tilapia genome. The darker the color on the representative heatmap, the more expressed transcripts are located on the corresponding LG.

Here, we were asking whether sex-biased genes are clustered together in the genome. We can access this information for the Nile tilapia profiting from our approach using the same reference data set for all analyses. We found that transcripts overexpressed in females of all four species were significantly overrepresented on LG1 compared with all other LGs (fig. 5, right panel, *P* value < 0.001 and supplementary table S4, Supplementary Material online), whereas transcripts overexpressed in all males were overrepresented on LG23 compared with all other LGs (fig. 5, left panel, *P* value < 0.001 and supplementary table S4, Supplementary Material online). Hence, genes with a presumably conserved function in sex determination in cichlids are clustering on the sex-linked LGs (the potential sex chromosomes) in the Nile tilapia. Species-specific overexpressed transcripts, on the other hand, grouped on different LGs and this clustering varies between species. We also tested for a genomic clustering of all overexpressed transcripts (including unique and shared transcripts) of a kind. The accumulation of female-specific/ovary overexpressed transcripts on LG1 seemed to be a general trend in the data set for all overexpressed transcripts in all mouth-brooding species. We did not see a common pattern for male sample/testis overexpressed transcripts when looking at the entire set of overexpressed genes.

Expression data for the Nile tilapia itself indicate sex-biased expression for candidate genes located on LG1 and LG23 (Ijiri et al. 2008; Eshel et al. 2012) but transcriptome-wide data for the Nile tilapia are limited. The data set of Tao et al. (2013) includes a list of “XY-enhanced” (e.g., male overexpressed) and “XX-enhanced” (i.e., female overexpressed) transcripts of the (developing) gonad. We blasted the two transcript groups against our reference set and could assign 931 of 1,358 XY-enhanced transcripts and 157 of 187 XX-enhanced transcripts to our reference. The male transcripts contained 23 transcripts on LG23 of which five were also overexpressed in the males of all four species investigated in this study. The female transcripts contained four transcripts on LG1 of which one transcript was also overexpressed in females of all four Lake Tanganyika species. However, the limited data set of Tao et al. (2013) does not allow for enrichment testing as it does not include transcript information on overall unbiased gene expression.

To gain further insight in the function of the transcripts located on LG1 and LG23, we tested whether these two LGs are enriched for specific pathways. To this aim, we compared pathway annotations for the commonly overexpressed transcripts located on LG1 and LG23 with transcripts overexpressed and located on all other LGs, respectively, using KOBAS (Xie et al. 2011). We found that the transcripts on LG1 are enriched for 16 pathways, among which were two pathways with a possible role in sex determination, “gene expression” and “progesterone-mediated oocyte maturation” (supplementary table S16, Supplementary Material online). On LG23, we found enrichment for 23 pathways including “Developmental Biology”; however, none of them seems to be specifically linked to sex determination (supplementary table S16, Supplementary Material online). We detected the pathways “Wnt ligand biogenesis and trafficking” as well as “Signaling by Wnt” on LG23; still, they are not significantly enriched.

Our constructed gene expression modules were evenly distributed over the genome (supplementary fig. S7, Supplementary Material online).

## Discussion

In this study, we investigated sex-specific gene-expression patterns in gonad and brain tissue within and between four cichlid species, representing the three species-richest tribes in Lake Tanganyika (Haplochromini, Ectodini, and Lamprologini) and a species-poor group (Eretmodini). We then focused on those genes that are DE between the sexes in all four species to identify a common network of sex determination/differentiation. In addition, we inspected uniquely expressed genes for their specific role and conducted a gene coexpression network analysis.

### Subtle Sex Differences in Overall Brain Gene Expression

Our study showed almost no sex-biased gene expression in the brain of cichlids. This has previously been observed in other vertebrates (Yang et al. 2006; Mank et al. 2008) and a similar result to ours was shown in another fish, the sharpsnout seabream (Manousaki et al. 2014). Note, however, that these approaches, including ours, examined the brain as a whole, whereas it is well established that sex-specific differences in gene expression are present in cichlids when only certain brain regions are considered (O’Connell et al. 2013).

We could show that genes known to interfere with social status/behavior can have different sex-specific patterns, depending on the investigated species. This could be linked to differences in social structure between species. The here observed sex-differences were likely based on brain-wide expression or high expression levels in certain regions and as such became detectable by our approach. In the cichlid *Oreochromis mossambicus*, *isotocin* has higher expression levels in the hindbrain of dominant males than of subordinate males (Almeida et al. 2012), whereas isotocin-treated *N. pulcher* show increased submissive behavior when challenged aggressively (Reddon et al. 2012). Here, we found *isotocin* overexpression in female brain of *E. cyanostictus* and in male brain of *A. burtoni*. These two species have different social structures: *E. cyanostictus* is a monogamous, biparental breeder with both sexes defending the breeding territorial (Morley and Balshine 2002; Taylor et al. 2003). In *A. burtoni*, males change between being dominant/territorial and subordinate/nonterritorial; females are either in gravid (egg bearing) or in nongravid status independent of social conditions (Clement et al. 2005). Hence, sex does not predict dominance per se in cichlids, which could be reflected in species-specific expression patterns of (dominance) behavior-related genes in the two sexes.

The brain expression data also revealed expression of transposable elements in the cichlid brain in *E. cyanostictus*, suggesting them to be active in this tissue. Active transposable elements, especially DNA transposons, are rather the exception in vertebrates (Böhne et al. 2008). Teleost genomes, however, contain a great variety of mobile DNA sequences including multiple families of functional and active transposable elements (e.g., Hori et al. 1998; Böhne et al. 2012). This also seems to be the case in cichlids (e.g., Shirak et al. 2010). Activity of transposable elements in the brain could be associated with aging and neuronal decline (Coufal et al. 2009; Li et al. 2013) but also occurs early in development during the formation of the central nervous system and adult neurogenesis (Singer et al. 2010). In contrast to mammals that have limited adult neurogenesis, the fish brain produces new neurons throughout its life (Kaslin et al. 2008). Sex differences in these processes remain to be investigated and data on sex-specific transposable element activity (in the soma) have to the best of our knowledge not been reported in fish although transposable elements can have a sex-specific role in the sequence evolution of sex chromosomes (e.g., Peichel et al. 2004; Böhne et al. 2012, and references therein). As indicated by the composition of coexpression modules associated with species, sex- and species-specific expression and hence maybe activity of transposable elements in the gonads seem to be yet another feature distinguishing cichlid species from each other in Lake Tanganyika.

### Do Mouth-Breeding, Dimorphic Cichlids Have More Male- Than Female-Biased Transcripts?

We observed that within sex-biased transcripts, male-biased transcripts outnumbered female-biased transcripts in all four investigated species. A similar trend has been observed in the *Drosophila* genus; however, the inverse pattern with more female-biased expression has also been described (Zhang et al. 2007). The gonad is likely the tissue that varies most between the sexes (Mank et al. 2008; Yang and Smith 2013) but at least two of our investigated species (*A. burtoni* and *Op. ventralis*) also show strong sexual dimorphism in body shape and coloration. These two species showed more testis- and male-specific transcripts compared with ovary- and female-specific ones than the mono-morphic *J. ornatus* (fig. 1, DE transcripts out of total expressed transcripts: *Op. ventralis* 34.7% in testis, 30.0% in ovary, 20.1% in male tissue, 14.5% in female tissue; *A. burtoni* 33.9% in testis, 30.7% in ovary, 17.0% in male tissue, 14.1% in female tissue; *J. ornatus* 33.5% in testis, 32.6% in ovary, 20.1% in male tissue, 14.5% in female tissue). *Eretmodus cyanostictus*, which shows a certain degree of sexual size dimorphism (Neat and Balshine-Ear 1999), had an even stronger trend toward more male-biased transcripts than female-biased ones (35.8% in testis, 30.4% in ovary, 23.3% in male tissue, 13.1% in female tissue).

Interestingly, the three sexually dimorphic species with more male-biased expression also have a similar reproductive strategy; they are all mouth-breeders, whereas *J. ornatus* is a substrate spawner. This could also be responsible for the association we found for the “darkorange,” “darkturquoise,” and “darkgray” modules with species. These modules grouped many testis-biased genes including several transposable element-related sequences and immune system factors in the mouth-breeders (darkorange), in *A. burtoni* (darkturquoise) and especially in *E. cyanostictus* (darkgray). A study on a larger set of species having either of the reproductive strategy and with and without sexual dimorphism should shed more light on this pattern.

### Transcripts Located on the Nile Tilapia Sex Chromosomes

A nonrandom accumulation of sex-biased genes on sex chromosomes compared with autosomes is a common pattern in animals, depending on the underlying sex chromosomal system: In male heterogametic (XX–XY) systems, the X chromosome is often enriched for female-biased genes, possibly caused by sex-specific selection shaping the X-chromosomal gene content (Parisi et al. 2003; Gupta et al. 2006; Sturgill et al. 2007). In species lacking dosage compensation, a female-biased overexpression of X-chromosomal genes could simply be a matter of the chromosomal constitution, as the double amount of X-chromosomal material is present in XX-females compared with XY-males. In addition, the X chromosome can also carry an excess of male-biased genes (Wang et al. 2001; Lercher et al. 2003; Yang et al. 2006), again resulting from sex-specific selection regimes. In female heterogametic ZZ–ZW systems, male-biased genes are overrepresented on the Z chromosome (Kaiser et al. 2006; Arunkumar et al. 2009).

In fishes, sex chromosomes are mostly monomorphic and possibly evolutionary young (Charlesworth et al. 2005). Hence, the sex-specific region in the genome is often small and the gene content between the two types of sex chromosomes is still very similar.

Overall, our data set suggested that a common set of sex-biased genes exists in cichlids and that this is overrepresented on the putative sex chromosomes of *Or. niloticus*. The female-biased genes on LG1 showed accumulation of genes with a female-specific function: Oocyte maturation. Cichlid genomes are highly syntenic suggesting that the sex-specific genes are also grouped in the genomes of the here-investigated cichlids.

The common, sex-specific expression pattern in the Lake Tanganyika species suggests that this is the ancestral expression pattern. It could reflect an ancestral location on a sex chromosome in the last common ancestor of the Nile tilapia and the East African lake cichlids, and hence a common regulation. On the other hand, a pre-existing cluster of sex-biased genes on an ancestral autosome could also have been a special part of the genome that then was selected in the Nile tilapia to become the sex chromosome. Expression data for the Nile tilapia itself indicate sex-biased expression for candidate genes located on the presumptive sex chromosomes LG1 and LG23 (Ijiri et al. 2008; Eshel et al. 2012). The expression data from the Lake Tanganyika species could help identify new candidate genes for sex determination in the Nile tilapia.

### Unique Transcripts versus a Common Genetic Network for Sex Determination in Fish

In this study, we investigated transcripts that were expressed in a sex-specific manner in all four species (making up almost half of all sex-specific transcripts), and on the other hand, we also inspected transcripts that showed a species-specific expression pattern. Within these two categories we focused on genes belonging to the GO classes sex determination and sex differentiation. Genes identified in the first category are the highest expressed ones and strong candidates for a common molecular network of sexual development, the process grouping together sex determination and sex differentiation. Among the common candidates for sexual development in cichlids are genes that have previously been implicated with sex determination and differentiation in vertebrates, such as *foxl2*, *sox3*, *dmrt2*, and *hmgb2*. The process of apoptosis likely also plays a role in the gonad of cichlid fishes, as already known from other vertebrates (Morais et al. 2012, and references therein).

A relatively large group of transcripts was found to be species-specifically overexpressed in one sex or the other (between 13.8 ± 1.6% and 21.2 ± 4.9% transcripts), including the inhibin/activin subgroup of the TGF-β-superfamily, the aromatase *cyp19a1*, and isoforms of the enigmatic tetrapod testis-genes *sox9* and *amh*. We hence confirmed and extended the recent observation that key genes in sexual development show expression patterns in the gonads of cichlids that are opposed to sex-specific patterns so far described in fishes. The estrogen pathway is one of these networks asking for in-depth examination in fish given its vast implication and common role in sex determination (Nakamura 2010). We confirm a new male-specific role for estrogen in the testis of *A. burtoni* and *Op. ventralis*, including one receptor in each species. In addition, the A-copy of the androgen receptor was found to be overexpressed in the ovary of *Op. ventralis* and *J. ornatus*. The action of androgens in the male gonad is well studied; their possible action in the female gonad is, however, unknown. Our results thus suggest that teleost fish or at least cichlids may use the two copies of the androgen receptor in a tissue-specific manner. This indicates that the sex-specific usage of sex steroid hormones in (cichlid) fish can depend on the species/tribe studied and that these hormones cannot per se be classified as male or female factors. This calls for a careful consideration of hormone measurements in fish, which are often correlated with a sex-specific function.

The expression patterns observed in cichlids suggest that even within groups of closely related species rapid shifts in expression can occur. It appears that genes or certain transcript variants can be associated with one sex in a species but with the opposite sex in another species. We did not find a correlation toward more shared genes for closer related species (e.g., *Op. ventralis* and *A. burtoni* compared with *Op. ventralis/J. ornatus* or *A. burtoni/J. ornatus*). Variation in and turnover of sex-biased gene expression was already shown in closely related species (e.g., inside the *Drosophila* genus; Zhang et al. 2007). This, combined with data showing accelerated rates of protein evolution for genes with sex-biased expression, led to the suggestion that sex-biased genes are more “dispensable” than unbiased genes (Mank and Ellegren 2009). The studied transcripts with a GO annotation for sex differentiation/determination are members of essential pathways for gonad development and reproduction. Information on dispensability of these genes and all other sex-biased transcripts observed here is currently not available, as functional studies for cichlids were established just recently and do not yet allow for large scale screens (Juntti et al. 2013). Protein-sequence evolution studies could help to complete the picture; however, no genome or complete transcriptome sequence data are available for *Op. ventralis*, *J. ornatus,* and *E. cyanostictus* hindering this approach for now. A de novo assembly for transcriptome data is in preparation for all species studied here, which should help answer questions concerning sequence evolution in these closely related species. Our previous study already demonstrated substantial sequence changes along the East African cichlid phylogeny for the aromatase *cypa9a1A* gene, questioning its function and hence its indispensability for sexual development in fish (Böhne et al. 2013). Also the gene coexpression modules pointed to species-specific action of gene networks in the testis.

Interestingly, both, unique and common overexpression, point out that also in East African cichlid fishes, the Wnt-pathway and several members of the TGF-β-superfamily are major players in sex determination/differentiation not only in a sex-specific manner but also in a species-specific manner. Especially inhibins and activins and their receptors were found among the species-specific transcripts and the BMP-proteins, their receptors and regulators in both groups. These factors are known to regulate the secretion of gonadal and pituitary hormones influencing germ cell development and the hypothalamus–pituitary–gonadal axis (e.g., Bilezikjian et al. 2004). This underlines, once more, the importance of the TGF-β-superfamily in sex determination in fish. This superfamily perfectly exemplifies candidates for the network of sex determination as defined by Uller and Helanterä (2011), namely genes and hormones impacting cell proliferation. Such factors can be a major control point in the decision toward the male or female fate in fish over the regulation of the number of primordial germ cells, a mechanism crucial in some fish (Kurokawa et al. 2007; Siegfried and Nüsslein-Volhard 2008).

With this study, we provide the largest body of sex-specific expression data currently available for cichlids. The largest fraction of sex-specific expressed transcripts was shared between species, albeit their differences in development, reproduction, and life style. However, we also showed that a substantial amount of transcripts (including different isoforms within the investigated species), with a potentially conserved role in sex determination/differentiation outside the fish-lineage, were species-limited in their sex-specific usage. Besides this large picture, the data set also allowed us to confirm previous data pointing to rapid evolutionary changes in sex determination in cichlids, including the sex steroid pathway. This calls for further investigation of sex determination in cichlid fishes, an ideal model system for comparative genetic studies with genetically very closely related species yet showing a tremendous variety of biological traits.

## Supplementary Material

Supplementary figures S1–S7 and tables S1–S16 are available at *Genome Biology and Evolution* online (http://www.gbe.oxfordjournals.org/).

Supplementary Data
