# Supplementary material for: Comparative Transcriptomics in East African Cichlids Reveals Sex- and Species-Specific Expression and New Candidates for Sex Differentiation in Fishes
Source: Genome Biol Evol. 2014 Sep 17;6(9):2567–85. doi: 10.1093/gbe/evu200 (PMC4202336; doi:10.1093/gbe/evu200)
Supplement: Supplementary Data [file supp_6_9_2567__index.html]

Comparative transcriptomics in East African cichlids reveals sex- and species-specific expression and new candidates for sex differentiation in fishes — Comparative Transcriptomics in East African Cichlids Reveals Sex- and Species-Specific Expression and New Candidates for Sex Differentiation in Fishes — Supplementary Data 

# Comparative Transcriptomics in East African Cichlids Reveals Sex- and Species-Specific Expression and New Candidates for Sex Differentiation in Fishes

## Supplementary Data

files

**Files in this Data Supplement:**

- Supplementary Data - pdf file
- Supplementary Data - xlsx file
