# Supplementary material for: Comparative Transcriptomics in East African Cichlids Reveals Sex- and Species-Specific Expression and New Candidates for Sex Differentiation in Fishes
Source: Genome Biol Evol. 2014 Sep 17;6(9):2567–85. doi: 10.1093/gbe/evu200 (PMC4202336; doi:10.1093/gbe/evu200)
Supplement: Supplementary Data [file supp_evu200_SupplementaryMaterial.pdf]

**Supplementary Material to**

Astrid Böhne, Thierry Sengstag & Walter Salzburger

"Comparative transcriptomics in East African cichlids reveals sex- and species-specific expression and new candidates for sex differentiation in fishes"

Including:

Supplementary Material Figure 1
Supplementary Material Figure 2
Supplementary Material Figure 3
Supplementary Material Figure 4
Supplementary Material Figure 5
Supplementary Material Figure 6
Supplementary Material Figure 7

Supplementary Material Table 1
Supplementary Material Table 2
Supplementary Material Table 3
Supplementary Material Table 5
Supplementary Material Table 6
Supplementary Material Table 7
Supplementary Material Table 8
Supplementary Material Table 9
Supplementary Material Table 10
Supplementary Material Table 11
Supplementary Material Table 12
Supplementary Material Table 13
Supplementary Material Table 14
Supplementary Material Table 15
Supplementary Material Table 16

**Supplementary Material Figure 1**
Expression levels of sex-specific transcripts
(see also Supplementary Material Table 2)
Left side: testis- and male-overexpressed transcripts, expression levels are shown as boxplots and curves, Right side: ovary- and female-overexpressed transcripts, expression levels are shown as boxplots and curves

**Supplementary Material Figure 2**
Characterization of species-specific transcripts
GO annotation (category biological process, level 2) for ovary/testis-specific transcripts only over-expressed in one species for all GO groups with more than 50 transcripts. The transcripts belonging to the GO terms sex determination and sex differentiation all belong to the ancestral GO term reproduction, depicted by colored arrows and actual gene numbers.

**Supplementary Material Figure 3**
GO categories for Biological Process for male-specific transcripts, equivalent to Main Manuscript Figure 3.

**Supplementary Material Figure 4**
GO categories for Biological Process for female-specific transcripts, equivalent to Main Manuscript Figure 3.

**Supplementary Material Figure 5**
Weighted gene co-expression network (WGCNA) analysis
**A)** Clustering dendrogram of samples based on their Euclidean distance (average linkage)
**B)** Analysis of network topology for various soft-thresholding powers. The left panel shows the scale-free fit index (y-axis) as a function of the soft-thresholding power (x-axis). The right panel displays the mean connectivity as a function of the soft-thresholding power.
**C)** Consensus clustering of consensus module eigengenes
The redline indicates the height cut for merging of modules with very similar expression, here set at 0.1 corresponding to a correlation higher than 0.9.

**Supplementary Material Figure 6**
Distribution of differentially expressed transcripts in the Nile tilapia genome
Transcripts with a sex-specific expression in all species in the test ovary versus testis and also with species-specific expression are grouped according to their location in the Nile tilapia genome. The darker the color on the representative heatmap, the more expressed transcripts are located on the corresponding linkage group (LG).

**Supplementary Material Figure 7**
Distribution of gene expression modules on the Nile tilapia chromosomes.
Genes per module are shown in percentage per linkage group.

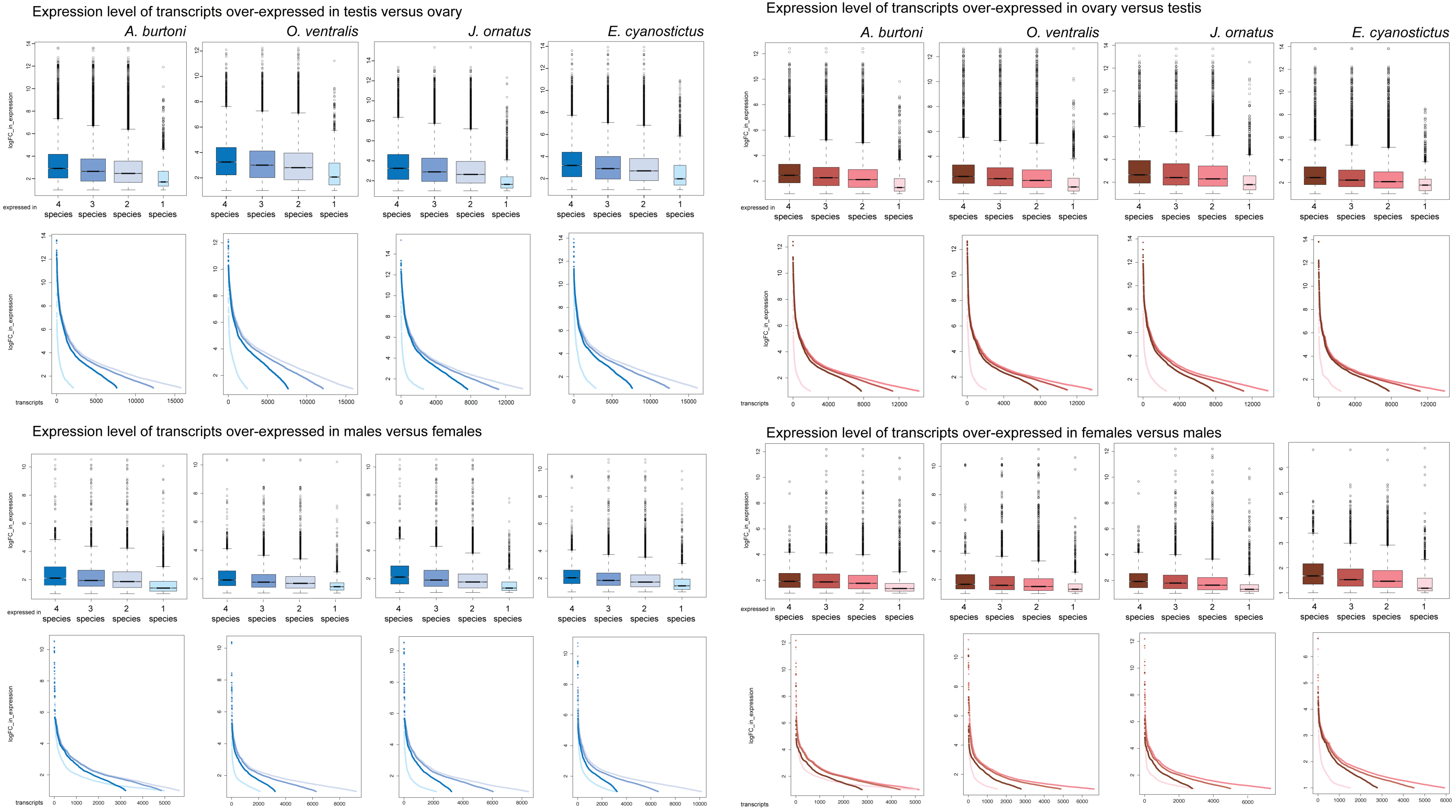


Supplementary Material Figure 1

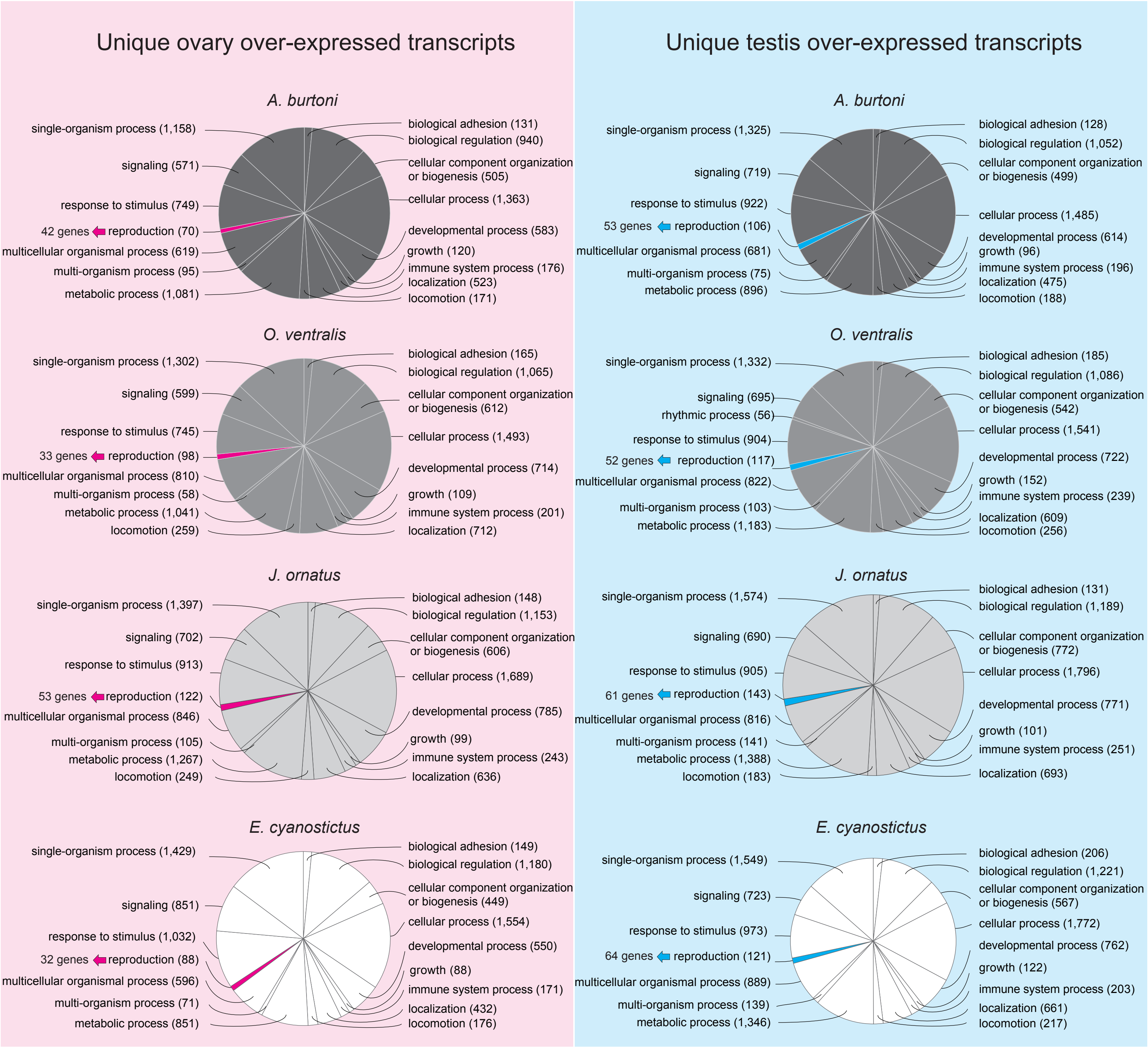


Supplementary Material Figure 2

# GO term analysis, Category Biological Process, Ontology Level 2, unique and shared male over-expressed transcripts

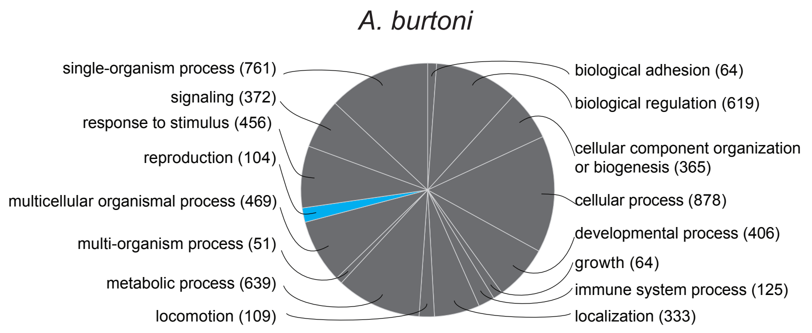


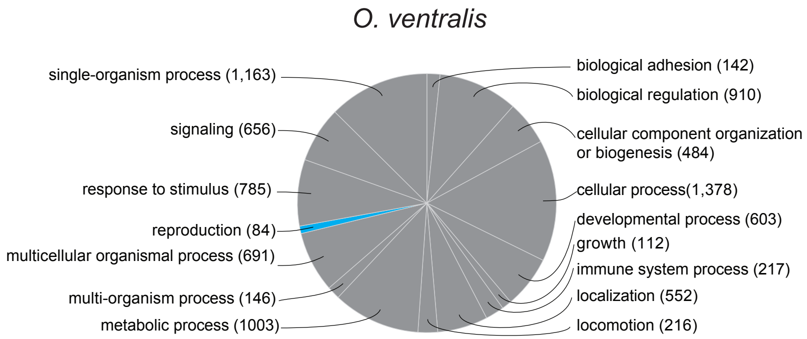


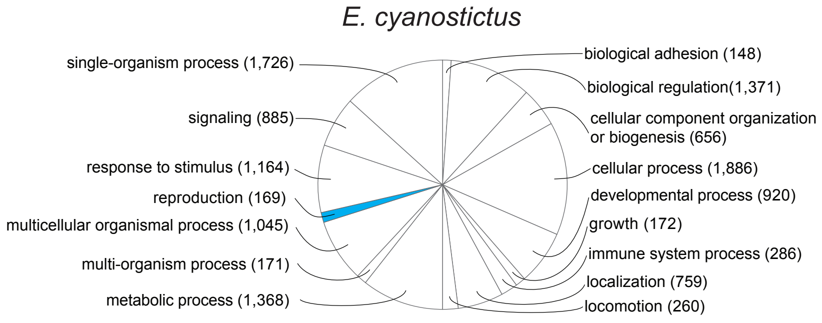


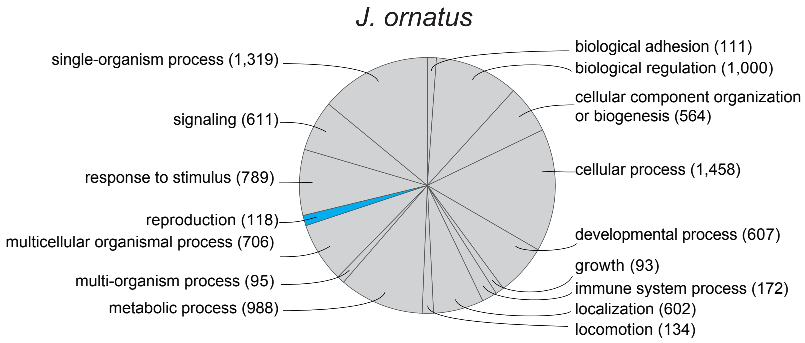


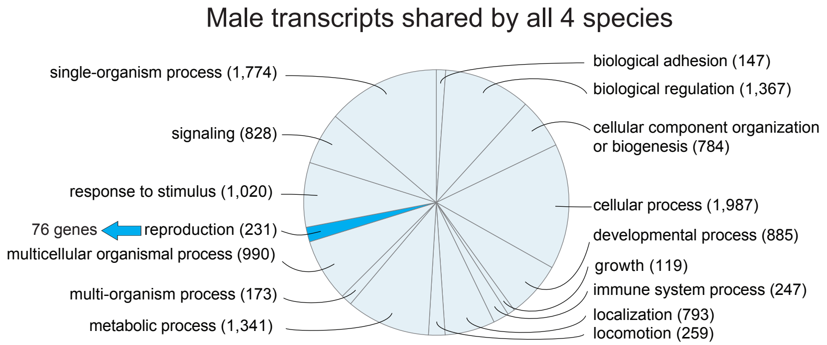


Supplementary Material Figure 3

# GO term analysis, Category Biological Process, Ontology Level 2, unique female over-expressed transcripts

*A. burtoni*

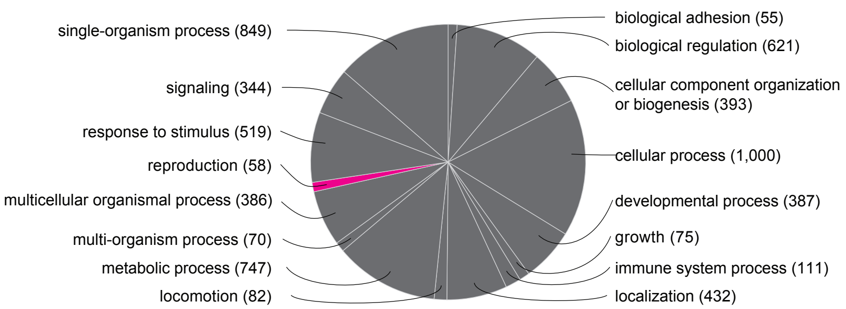


*O. ventralis*

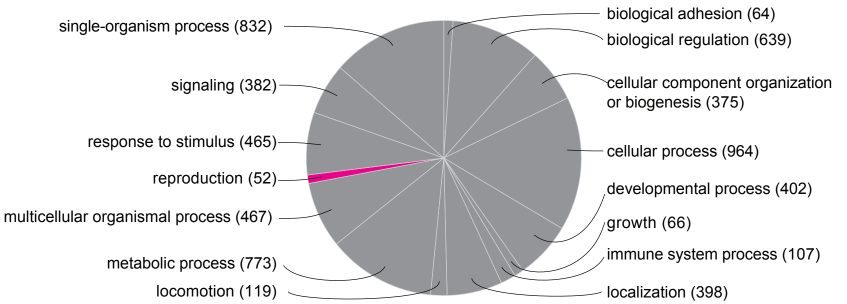


*E. cyanostictus*

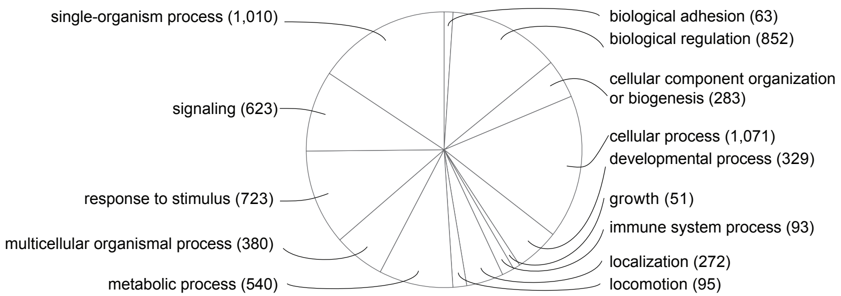


*J. ornatus*

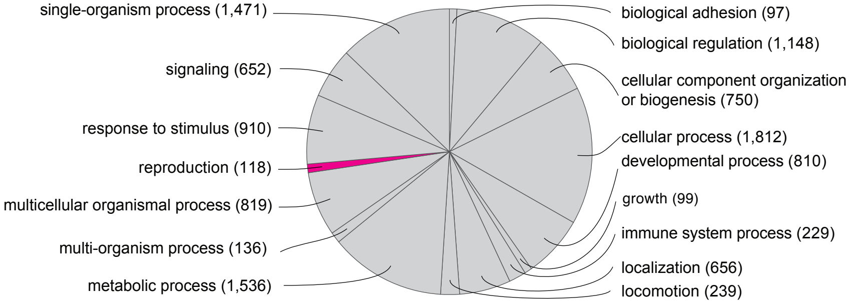


Female transcripts shared by all 4 species

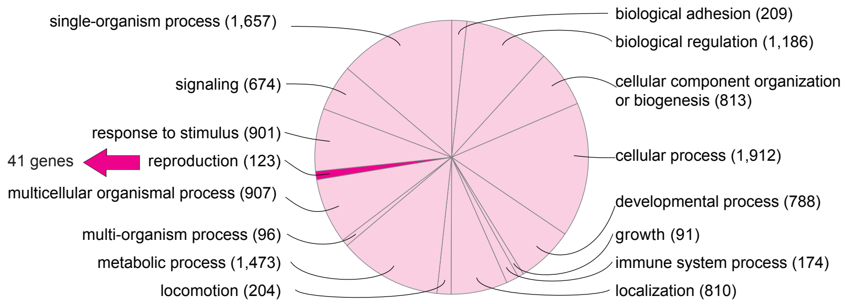


Supplementary Material Figure 4

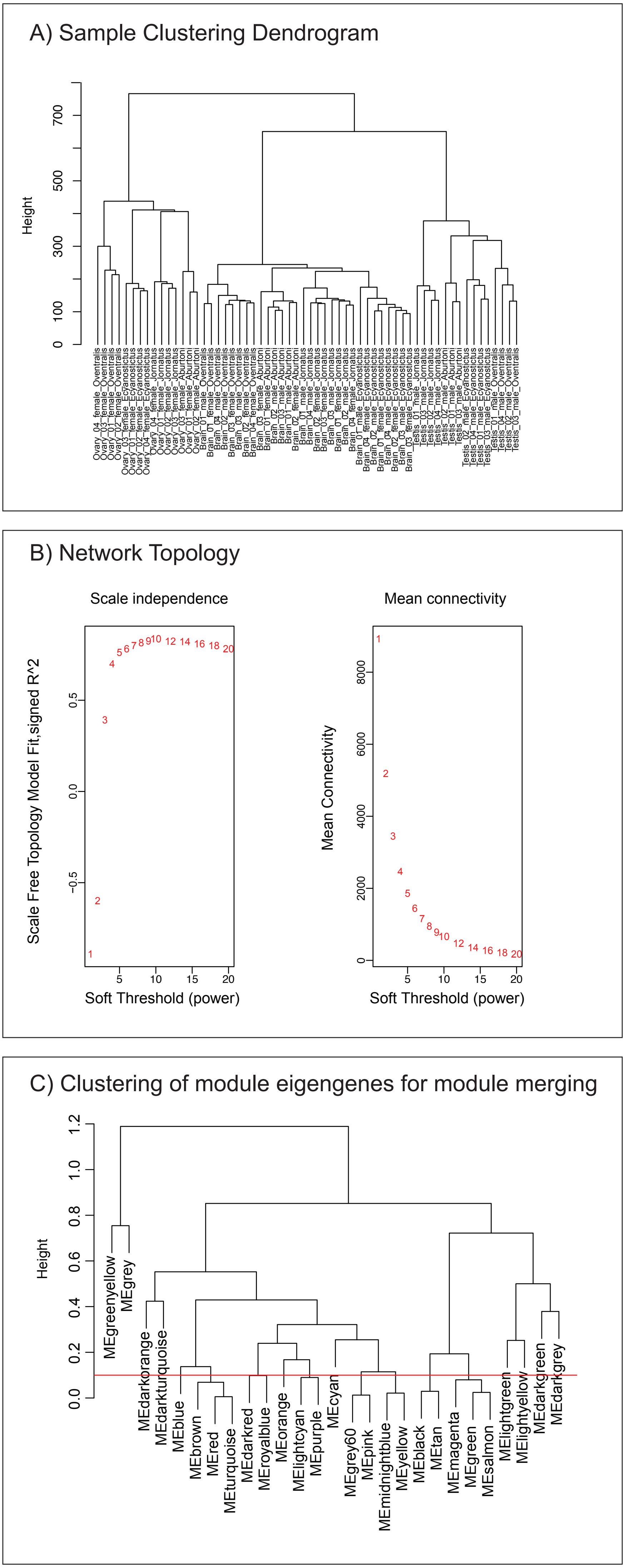


Supplementary Material Figure 5

# Distribution of differentially expressed transcripts in the Nile tilapia genome

## Testis over-expressed transcripts

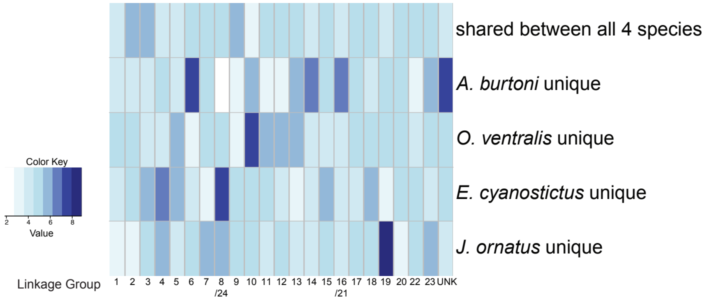


## Ovary over-expressed transcripts

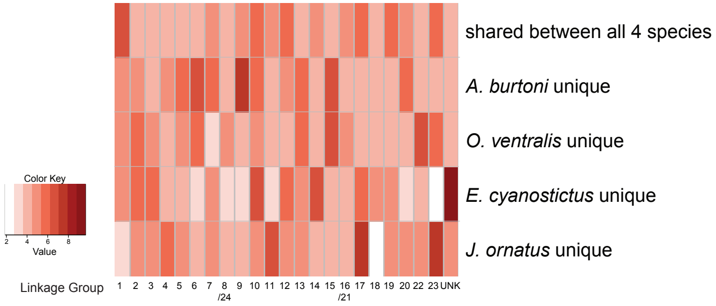


Supplementary Material Figure 6

# Distribution of gene expression modules on the Nile tilapia chromosomes

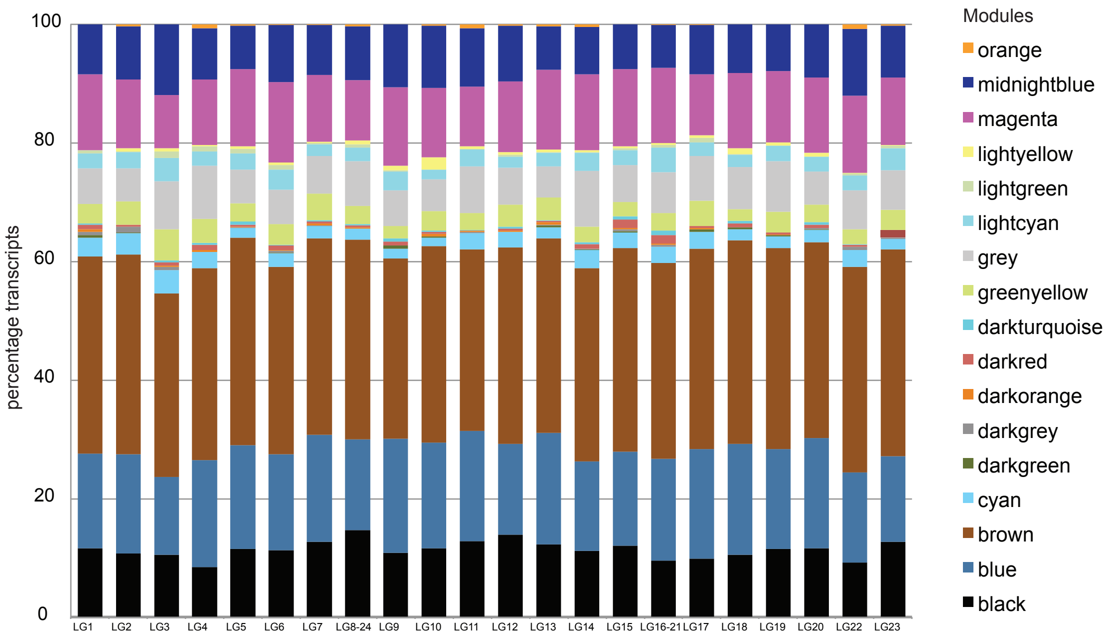


Supplementary Material Figure 7

**Supplementary Material Table 1**
**Statistics for read cleaning and aligning**

| Species | Tissue | Input Cleaned Reads | Aligned Reads | percentage Aligned Reads | Sum all Cleaned Reads | Sum all Aligned Reads | Percentage Aligned Reads |
|---|---|---|---|---|---|---|---|
| *A. burtoni* | BrainM1 | 10442056 | 6900395 | 66.08 | 176469254 | 122986068 | 69.69 |
| *A. burtoni* | BrainM2 | 15431436 | 8722656 | 56.53 | | | |
| *A. burtoni* | BrainM3 | 19176876 | 12641699 | 65.92 | | | |
| *A. burtoni* | BrainF1 | 15101039 | 10207860 | 67.60 | | | |
| *A. burtoni* | BrainF2 | 10980949 | 7205198 | 65.62 | | | |
| *A. burtoni* | BrainF3 | 20306244 | 13392877 | 65.95 | | | |
| *A. burtoni* | TestisM1 | 14663786 | 10748890 | 73.30 | | | |
| *A. burtoni* | TestisM2 | 18395237 | 13606061 | 73.97 | | | |
| *A. burtoni* | TestisM3 | 9058801 | 6514846 | 71.92 | | | |
| *A. burtoni* | OvaryF1 | 12885169 | 10097349 | 78.36 | | | |
| *A. burtoni* | OvaryF2 | 19114718 | 15012908 | 78.54 | | | |
| *A. burtoni* | OvaryF3 | 10912943 | 7935329 | 72.71 | | | |
| *O. ventralis* | BrainM1 | 11326635 | 7325157 | 64.67 | 229399817 | 149536351 | 65.19 |
| *O. ventralis* | BrainM2 | 20438695 | 12072166 | 59.07 | | | |
| *O. ventralis* | BrainM3 | 11432560 | 7185992 | 62.86 | | | |
| *O. ventralis* | BrainM4 | 10161929 | 5871532 | 57.78 | | | |
| *O. ventralis* | BrainF1 | 16020911 | 10512390 | 65.62 | | | |
| *O. ventralis* | BrainF2 | 17185135 | 10775105 | 62.70 | | | |
| *O. ventralis* | BrainF3 | 16633587 | 9962690 | 59.90 | | | |
| *O. ventralis* | BrainF4 | 13668863 | 8552114 | 62.57 | | | |
| *O. ventralis* | TestisM1 | 15204420 | 10191349 | 67.03 | | | |
| *O. ventralis* | TestisM2 | 11470322 | 7650712 | 66.70 | | | |
| *O. ventralis* | TestisM3 | 13761172 | 9366461 | 68.06 | | | |
| *O. ventralis* | TestisM4 | 18601026 | 12704728 | 68.30 | | | |
| *O. ventralis* | OvaryF1 | 12582065 | 9589391 | 76.21 | | | |
| *O. ventralis* | OvaryF2 | 14010809 | 10415732 | 74.34 | | | |
| *O. ventralis* | OvaryF3 | 19329825 | 14584358 | 75.45 | | | |
| *O. ventralis* | OvaryF4 | 7571863 | 2776474 | 36.67 | | | |
| *E. cyanostictus* | BrainM1 | 19305952 | 12400746 | 64.23 | 319979731 | 221603237 | 69.26 |
| *E. cyanostictus* | BrainM2 | 18730026 | 11585427 | 61.85 | | | |
| *E. cyanostictus* | BrainM3 | 21669914 | 14143441 | 65.27 | | | |
| *E. cyanostictus* | BrainM4 | 19718030 | 12693547 | 64.38 | | | |
| *E. cyanostictus* | BrainF1 | 14478093 | 9358495 | 64.64 | | | |
| *E. cyanostictus* | BrainF2 | 21194871 | 14069542 | 66.38 | | | |
| *E. cyanostictus* | BrainF3 | 21773183 | 14375749 | 66.03 | | | |
| *E. cyanostictus* | BrainF4 | 22435192 | 14482933 | 64.55 | | | |
| *E. cyanostictus* | TestisM1 | 17309193 | 11857989 | 68.51 | | | |
| *E. cyanostictus* | TestisM2 | 21185030 | 15153582 | 71.53 | | | |
| *E. cyanostictus* | TestisM3 | 20865514 | 15064654 | 72.20 | | | |
| *E. cyanostictus* | TestisM4 | 10788405 | 6962986 | 64.54 | | | |
| *E. cyanostictus* | OvaryF1 | 21381121 | 16302937 | 76.25 | | | |
| *E. cyanostictus* | OvaryF2 | 26718210 | 20582615 | 77.04 | | | |
| *E. cyanostictus* | OvaryF3 | 19764706 | 15191403 | 76.86 | | | |
| *E. cyanostictus* | OvaryF4 | 22662291 | 17377191 | 76.68 | | | |
| *J. ornatus* | BrainM1 | 17673313 | 11627783 | 65.79 | 298384809 | 211459903 | 70.87 |
| *J. ornatus* | BrainM2 | 21557540 | 14397658 | 66.79 | | | |
| *J. ornatus* | BrainM3 | 12052516 | 7943250 | 65.91 | | | |
| *J. ornatus* | BrainM4 | 14046895 | 9126885 | 64.97 | | | |
| *J. ornatus* | BrainF1 | 17280118 | 10949097 | 63.36 | | | |
| *J. ornatus* | BrainF2 | 18398359 | 12282488 | 66.76 | | | |
| *J. ornatus* | BrainF3 | 20833868 | 13925406 | 66.84 | | | |
| *J. ornatus* | BrainF4 | 21111040 | 13619822 | 64.52 | | | |
| *J. ornatus* | TestisM1 | 10767619 | 7885920 | 73.24 | | | |
| *J. ornatus* | TestisM2 | 23403601 | 17587366 | 75.15 | | | |
| *J. ornatus* | TestisM3 | 17803780 | 13383818 | 75.17 | | | |
| *J. ornatus* | TestisM4 | 21213176 | 15746208 | 74.23 | | | |
| *J. ornatus* | OvaryF1 | 15687474 | 11907603 | 75.91 | | | |
| *J. ornatus* | OvaryF2 | 26913881 | 20718002 | 76.98 | | | |
| *J. ornatus* | OvaryF3 | 15971181 | 12207738 | 76.44 | | | |
| *J. ornatus* | OvaryF4 | 23670448 | 18150859 | 76.68 | | | |

**Supplementary Material Table 2**

Statistics on logFC expression values in the different compared groups shown in Supplementary Material Figure 1

| Test | Group | Min value | Max value | 1st Quartile | 3rd Quartile | Median | Mean |
|---|---|---|---|---|---|---|---|
| over expressed in ovary vs. testis | *A. burtoni* 1* | 1.000 | 9.821 | 1.201 | 2.190 | 1.490 | 1.905 |
| | *A. burtoni* 2 | 1.000 | 12.410 | 1.495 | 2.905 | 2.112 | 2.482 |
| | *A. burtoni* 3 | 1.000 | 12.410 | 1.635 | 3.078 | 2.257 | 2.641 |
| | *A. burtoni* 4 | 1.001 | 12.410 | 1.856 | 3.320 | 2.450 | 2.883 |
| | *O. ventralis* 1 | 1.001 | 12.650 | 1.213 | 2.248 | 1.543 | 2.032 |
| | *O. ventralis* 2 | 1.000 | 12.650 | 1.496 | 2.921 | 2.067 | 2.493 |
| | *O. ventralis* 3 | 1.000 | 12.650 | 1.635 | 3.092 | 2.209 | 2.644 |
| | *O. ventralis* 4 | 1.001 | 12.650 | 1.836 | 3.316 | 2.391 | 2.864 |
| | *J. ornatus* 1 | 1.001 | 12.530 | 1.326 | 2.564 | 1.785 | 2.202 |
| | *J. ornatus* 2 | 1.000 | 13.700 | 1.647 | 3.423 | 2.292 | 2.790 |
| | *J. ornatus* 3 | 1.000 | 13.700 | 1.747 | 3.626 | 2.403 | 2.931 |
| | *J. ornatus* 4 | 1.001 | 13.090 | 1.919 | 3.903 | 2.645 | 3.171 |
| | *E. cyanostictus* 1 | 1.000 | 8.510 | 1.268 | 2.294 | 1.764 | 2.022 |
| | *E. cyanostictus* 2 | 1.000 | 13.830 | 1.493 | 2.939 | 2.066 | 2.470 |
| | *E. cyanostictus* 3 | 1.000 | 13.830 | 1.621 | 3.094 | 2.206 | 2.613 |
| | *E. cyanostictus* 4 | 1.001 | 13.830 | 1.804 | 3.383 | 2.426 | 2.861 |
| | | | | | | | |
| over expressed in females vs males (corrected for tissue effect) | *A. burtoni* 1 | 1.000 | 11.520 | 1.158 | 1.759 | 1.361 | 1.603 |
| | *A. burtoni* 2 | 1.001 | 12.190 | 1.334 | 2.397 | 1.767 | 2.056 |
| | *A. burtoni* 3 | 1.001 | 12.190 | 1.399 | 2.493 | 1.875 | 2.127 |
| | *A. burtoni* 4 | 1.001 | 9.671 | 1.441 | 2.543 | 1.919 | 2.112 |
| | *O. ventralis* 1 | 1.000 | 10.620 | 1.134 | 1.689 | 1.306 | 1.569 |
| | *O. ventralis* 2 | 1.000 | 11.200 | 1.213 | 2.053 | 1.499 | 1.806 |
| | *O. ventralis* 3 | 1.001 | 10.540 | 1.269 | 2.218 | 1.583 | 1.883 |
| | *O. ventralis* 4 | 1.003 | 10.130 | 1.324 | 2.347 | 1.655 | 1.909 |
| | *J. ornatus* 1 | 1.000 | 10.660 | 1.144 | 1.672 | 1.317 | 1.542 |
| | *J. ornatus* 2 | 1.000 | 12.190 | 1.274 | 2.229 | 1.636 | 1.946 |
| | *J. ornatus* 3 | 1.001 | 12.190 | 1.361 | 2.417 | 1.794 | 2.064 |
| | *J. ornatus* 4 | 1.001 | 9.671 | 1.441 | 2.543 | 1.919 | 2.112 |
| | *E. cyanostictus* 1 | 1.000 | 6.760 | 1.077 | 1.580 | 1.181 | 1.425 |
| | *E. cyanostictus* 2 | 1.000 | 6.695 | 1.209 | 1.885 | 1.459 | 1.652 |
| | *E. cyanostictus* 3 | 1.000 | 6.695 | 1.262 | 1.947 | 1.523 | 1.712 |
| | *E. cyanostictus* 4 | 1.002 | 6.695 | 1.342 | 2.157 | 1.672 | 1.834 |
| | | | | | | | |
| over expressed in testis vs. ovary | *A. burtoni* 1 | 1.001 | 11.920 | 1.332 | 2.656 | 1.709 | 2.271 |
| | *A. burtoni* 2 | 1.000 | 13.630 | 1.664 | 3.560 | 2.464 | 2.941 |
| | *A. burtoni* 3 | 1.000 | 13.630 | 1.779 | 3.757 | 2.649 | 3.125 |
| | *A. burtoni* 4 | 1.001 | 13.630 | 2.054 | 4.172 | 2.907 | 3.462 |
| | *O. ventralis* 1 | 1.001 | 11.230 | 1.444 | 3.166 | 2.066 | 2.471 |
| | *O. ventralis* 2 | 1.000 | 12.250 | 1.851 | 3.959 | 2.805 | 3.136 |
| | *O. ventralis* 3 | 1.000 | 12.250 | 2.018 | 4.121 | 3.000 | 3.320 |
| | *O. ventralis* 4 | 1.000 | 12.090 | 2.232 | 4.395 | 3.248 | 3.563 |
| | *J. ornatus* 1 | 1.001 | 12.320 | 1.263 | 2.402 | 1.639 | 2.119 |
| | *J. ornatus* 2 | 1.000 | 15.340 | 1.772 | 3.943 | 2.636 | 3.191 |
| | *J. ornatus* 3 | 1.000 | 15.340 | 1.940 | 4.266 | 2.895 | 3.422 |
| | *J. ornatus* 4 | 1.000 | 13.370 | 2.141 | 4.630 | 3.253 | 3.741 |
| | *E. cyanostictus* 1 | 1.000 | 10.890 | 1.416 | 3.205 | 1.982 | 2.615 |
| | *E. cyanostictus* 2 | 1.000 | 13.930 | 1.814 | 3.825 | 2.699 | 3.082 |
| | *E. cyanostictus* 3 | 1.000 | 13.930 | 1.958 | 4.012 | 2.910 | 3.241 |
| | *E. cyanostictus* 4 | 1.001 | 13.610 | 2.169 | 4.401 | 3.192 | 3.561 |
| | | | | | | | |
| over expressed in males vs females (corrected | *A. burtoni* 1 | 1.000 | 10.080 | 1.181 | 1.881 | 1.403 | 1.678 |
| | *A. burtoni* 2 | 1.000 | 10.540 | 1.437 | 2.559 | 1.861 | 2.154 |
| | *A. burtoni* 3 | 1.000 | 10.540 | 1.528 | 2.669 | 1.938 | 2.245 |
| | *A. burtoni* 4 | 1.000 | 10.540 | 1.607 | 2.911 | 2.104 | 2.373 |
| | *O. ventralis* 1 | 1.000 | 10.210 | 1.201 | 1.716 | 1.441 | 1.577 |

| | | | | | | | |
|---|---|---|---|---|---|---|---|
| for tissue effect) | *O. ventralis* 2 | 1.000 | 10.380 | 1.337 | 2.164 | 1.676 | 1.887 |
| | *O. ventralis* 3 | 1.000 | 10.380 | 1.406 | 2.304 | 1.761 | 1.996 |
| | *O. ventralis* 4 | 1.000 | 10.380 | 1.515 | 2.555 | 1.913 | 2.148 |
| | *J. ornatus* 1 | 1.000 | 7.746 | 1.139 | 1.756 | 1.318 | 1.579 |
| | *J. ornatus* 2 | 1.000 | 10.540 | 1.325 | 2.328 | 1.754 | 2.023 |
| | *J. ornatus* 3 | 1.000 | 10.540 | 1.436 | 2.594 | 1.891 | 2.178 |
| | *J. ornatus* 4 | 1.000 | 10.540 | 1.607 | 2.911 | 2.104 | 2.104 |
| | *E. cyanostictus* 1 | 1.000 | 9.855 | 1.171 | 1.936 | 1.444 | 1.707 |
| | *E. cyanostictus* 2 | 1.000 | 10.710 | 1.373 | 2.244 | 1.719 | 1.958 |
| | *E. cyanostictus* 3 | 1.000 | 10.710 | 1.474 | 2.383 | 1.839 | 2.083 |
| | *E. cyanostictus* 4 | 1.000 | 9.514 | 1.607 | 2.596 | 2.033 | 2.244 |

*1: unique expressed transcripts
2: transcripts also over-expressed in one other species
3: transcripts also over-expressed in two other species
4: transcripts also over-expressed in three other species

Statistics Kruskal-Wallis test:
Always: degrees of freedom = 3
And p-value < 2.2e-16

*A. burtoni* testis
chi-squared = 1154.243

*A. burtoni* ovary
chi-squared = 1410.333

*O. ventralis* testis
chi-squared = 1041.455

*O. ventralis* ovary
chi-squared = 1215.18

*J. ornatus* testis
chi-squared = 2018.759

*J. ornatus* ovary
chi-squared = 1039.301

*E. cyanostictus* testis
chi-squared = 1069.352

*E. cyanostictus* ovary
chi-squared = 1092.546

*A. burtoni* males
chi-squared = 1957.727

*A. burtoni* female
chi-squared = 599.1672

*O. ventralis* males
chi-squared = 902.1537

*O. ventralis* female
chi-squared = 444.5809

*J. ornatus* males
chi-squared = 1432.525

*J. ornatus* female
chi-squared = 1113.481

*E. cyanostictus* males
chi-squared = 1106.938

*E. cyanostictus* female
chi-squared = 739.3213

## Supplementary Material Table 3

Expression of candidate genes in the RNA-sequencing dataset

| Gene | Accession number | hits on *O.niloticus* reference | *A. burtoni* | | *O. ventralis* | | *J. ornatus* | | *E. cyanostictus* | |
|---|---|---|---|---|---|---|---|---|---|---|
| | | | **testis logFC** | **ovary logFC** | **testis logFC** | **ovary logFC** | **testis logFC** | **ovary logFC** | **testis logFC** | **ovary logFC** |
| ctnnb1A | ENSONIG00000007226 | on.mrna.LG11.350.1 | expressed but not significantly DE | expressed but not significantly DE | expressed but not significantly DE | expressed but not significantly DE | expressed but not significantly DE | expressed but not significantly DE | expressed but not significantly DE | expressed but not significantly DE |
| | | on.mrna.LG11.350.2 | expressed but not significantly DE | expressed but not significantly DE | expressed but not significantly DE | expressed but not significantly DE | expressed but not significantly DE | expressed but not significantly DE | expressed but not significantly DE | expressed but not significantly DE |
| | | on.mrna.LG11.350.3 | expressed but not significantly DE | expressed but not significantly DE | expressed but not significantly DE | expressed but not significantly DE | expressed but not significantly DE | expressed but not significantly DE | expressed but not significantly DE | expressed but not significantly DE |
| | | on.mrna.LG11.350.4 | expressed but not significantly DE | expressed but not significantly DE | expressed but not significantly DE | expressed but not significantly DE | expressed but not significantly DE | expressed but not significantly DE | expressed but not significantly DE | expressed but not significantly DE |
| | | on.mrna.LG11.350.5 | expressed but not significantly DE | expressed but not significantly DE | expressed but not significantly DE | expressed but not significantly DE | expressed but not significantly DE | expressed but not significantly DE | expressed but not significantly DE | expressed but not significantly DE |
| cyp11b2 | XM_003450906.1 | on.mrna.LG11.412.1 | 8.867895169 | | 12.09162978 | | 6.326277642 | | 10.84631637 | |
| | | on.mrna.LG11.412.2 | 5.765869734 | | 9.89066012 | | 6.463028099 | | 5.630702744 | |
| | | on.mrna.LG11.412.3 | 5.782970183 | | 9.535033658 | | 6.35279532 | | 5.644006271 | |
| | | on.mrna.LG11.412.4 | 5.818914468 | | 8.25356894 | | 3.478983613 | | 5.319991151 | |
| | | on.mrna.LG11.412.5 | 5.794636695 | | 9.927423288 | | 6.476426525 | | 5.667604852 | |
| | | Average | 6.40605725 | | 9.939663158 | | 5.81950224 | | 6.621724278 | |
| | | STDV | 1.376344364 | | 1.382041082 | | 1.310050124 | | 2.365908259 | |
| cyp19a1A | ENSONIG00000000155 | on.mrna.LG1.93.1 | | 6.045643379 | 2.664472879 | | | 8.028360714 | | 7.859832091 |
| cyp19a1B | ENSONIG00000008307 | on.mrna.LG13.740.1 | 8.34487395 | | expressed but not significantly DE | expressed but not significantly DE | | 2.731698132 | / | / |
| | | on.mrna.LG13.740.2 | 8.340054711 | | expressed but not significantly DE | expressed but not significantly DE | | 2.683849276 | / | / |
| | | on.mrna.LG13.740.3 | 8.358124881 | | expressed but not significantly DE | expressed but not significantly DE | | 2.123131829 | / | / |
| | | on.mrna.LG13.740.4 | 8.353861411 | | expressed but not significantly DE | expressed but not significantly DE | | 2.069193826 | / | / |
| | | on.mrna.LG13.740.5 | 8.359291831 | | expressed but not significantly DE | expressed but not significantly DE | | 2.135375222 | / | / |
| | | on.mrna.LG13.740.6 | 8.355021224 | | expressed but not significantly DE | expressed but not significantly DE | | 2.081596844 | / | / |
| | | Average | 8.351871335 | | | | | 2.304140855 | | |
| | | STDV | 0.007702581 | | | | | 0.31399228 | | |
| dax1A | DQ269441.1 | on.mrna.LG16-21.282.1 | expressed but not significantly DE | expressed but not significantly DE | 1.447445096 | | | 1.513113226 | 1.20304097 | |
| dmrt1 | AF203489.1 | on.mrna.LG12.915.2 | 5.182613827 | | 2.850069764 | | 3.014829884 | | 7.058250893 | |
| | | on.mrna.LG12.915.1 | 5.19934457 | | 2.885617557 | | 3.000948115 | | 7.064135728 | |
| | | Average | 5.190979199 | | 2.86784366 | | 3.007888999 | | 7.06119331 | |
| | | STDV | 0.011830422 | | 0.025136085 | | 0.009815893 | | 0.004161207 | |
| figla | ENSONIG00000015856 | on.mrna.LG12.121.1 | | 6.756465352 | | 5.987218958 | 1.191285644 | | | 3.242350595 |
| | | on.mrna.LG12.121.2 | | 6.861160995 | | 6.033148181 | 1.341153359 | | | 3.024022946 |
| | | Average | | 6.808813174 | | 6.01018357 | 1.266219501 | | | 3.133186771 |
| | | STDV | | 0.074031 | | 0.032476865 | 0.105972477 | | | 0.154380961 |
| foxl2A | AM232737.1 | on.mrna.UNK211.2.1 | | 4.740952658 | | 2.701157001 | | 5.729552087 | | 4.610267644 |
| foxl2B | XM_003444909.1 | on.mrna.LG20.823.1 | 3.79728231 | | / | / | / | / | 3.169158877 | |
| gata4 | EF577031.1 | on.mrna.LG15.456.1 | expressed but not significantly DE | expressed but not significantly DE | 2.084869823 | | / | / | 1.858304096 | |
| nanos1A | ENSONIG00000020951 | on.mrna.LG2.97.1 | / | / | / | / | / | / | expressed but not significantly DE | expressed but not significantly DE |
| nanos1B | ENSONIG00000021034 | on.mrna.LG19.703.1 | | 2.406484921 | expressed but not significantly DE | expressed but not significantly DE | / | / | expressed but not significantly DE | expressed but not significantly DE |
| rspondin | XM_003450518.1 | on.mrna.LG11.684.1 | / | / | / | / | / | / | / | / |
| | | on.mrna.LG11.685.1 | / | / | / | / | / | / | / | / |
| nr5a2 | ENSONIG00000012517 | on.mrna.UNK4.20.1 | / | / | / | / | / | / | / | / |
| sf1A(nr5a1) | ENSONIG00000020218 | on.mrna.LG12.17.1 | 3.549245214 | | 5.24076521 | | | 2.414793931 | 1.545143569 | |
| | | on.mrna.LG12.16.1 | 3.401105714 | | 4.130328243 | | | 2.022357553 | 2.00646415 | |
| | | Average | 3.475175464 | | 4.685546726 | | | 2.218575742 | 1.77580386 | |
| | | STDV | 0.104750445 | | 0.785197509 | | | 0.277494424 | 0.326202912 | |
| nr5a5 | ENSONIG00000001686 | on.mrna.LG4.556.1 | / | / | / | / | | 7.212050622 | / | / |
| | | on.mrna.LG4.556.2 | / | / | / | / | | 7.191777712 | / | / |
| | | Average | | | | | | 7.201914167 | | |
| | | STDV | | | | | | 0.014335112 | | |
| sox9A | ENSONIG00000019626 | on.mrna.LG8-24.118.1 | 1.545436931 | | / | / | / | / | / | / |
| | | on.mrna.LG8-24.117.1 | / | / | / | / | / | / | / | / |
| sox9B | ENSONIG00000005267 | on.mrna.UNK6.32.1 | expressed but not significantly DE | expressed but not significantly DE | expressed but not significantly DE | expressed but not significantly DE | expressed but not significantly DE | expressed but not significantly DE | 1.618896763 | |
| wnt4A | ENSONIG00000020236 | on.mrna.LG5.123.1 | | 3.065578382 | / | / | / | / | | 1.481038584 |
| wnt4B | ENSONIG00000012962 | on.mrna.LG11.589.1 | / | / | 8.480756692 | | 4.40881882 | | 5.963262565 | |
| | | on.mrna.LG11.589.2 | / | / | 8.446363293 | | 4.417916944 | | 6.33021966 | |
| | | on.mrna.LG11.589.3 | / | / | 8.208161656 | | 4.370177203 | | 5.916540187 | |
| | | Average | | | 8.378427214 | | 4.398970989 | | 6.07000747 | |
| | | STDV | | | 0.148453685 | | 0.025347693 | | 0.226558012 | |
| wt1A | XM_003439429.1 | on.mrna.LG7.175.1 | expressed but not significantly DE | expressed but not significantly DE | 2.021941828 | | expressed but not significantly DE | expressed but not significantly DE | 2.331452202 | expressed but not significantly DE |
| | | on.mrna.LG7.175.2 | expressed but not significantly DE | expressed but not significantly DE | 2.013310573 | | expressed but not significantly DE | expressed but not significantly DE | 2.303136761 | expressed but not significantly DE |
| | | on.mrna.LG7.175.3 | expressed but not significantly DE | expressed but not significantly DE | 2.003766291 | | expressed but not significantly DE | expressed but not significantly DE | 2.312038526 | expressed but not significantly DE |
| | | on.mrna.LG7.175.4 | expressed but not significantly DE | expressed but not significantly DE | 1.97648933 | | expressed but not significantly DE | expressed but not significantly DE | 2.297860831 | expressed but not significantly DE |
| | | on.mrna.LG7.175.5 | expressed but not significantly DE | expressed but not significantly DE | 2.012149009 | | expressed but not significantly DE | expressed but not significantly DE | 2.335044607 | expressed but not significantly DE |
| | | on.mrna.LG7.175.6 | expressed but not significantly DE | expressed but not significantly DE | 1.967046357 | | expressed but not significantly DE | expressed but not significantly DE | 2.306738954 | expressed but not significantly DE |
| | | on.mrna.LG7.175.7 | expressed but not significantly DE | expressed but not significantly DE | 2.049920198 | | expressed but not significantly DE | expressed but not significantly DE | 2.340578667 | expressed but not significantly DE |
| | | on.mrna.LG7.175.8 | expressed but not significantly DE | expressed but not significantly DE | 2.059649272 | | expressed but not significantly DE | expressed but not significantly DE | 2.331519951 | expressed but not significantly DE |
| | | on.mrna.LG7.175.9 | expressed but not significantly DE | expressed but not significantly DE | 2.021770978 | | expressed but not significantly DE | expressed but not significantly DE | 2.326010576 | expressed but not significantly DE |
| | | Average | | | 2.014004871 | | | | 2.320486786 | |
| | | STDV | | | 0.030037273 | | | | 0.015658186 | |

| | | | | | | | | | | |
|---|---|---|---|---|---|---|---|---|---|---|
| wt1B | XM_003442233.1 | on.mrna.LG1.397.1 | | 1.261714676 | expressed but not significantly DE | expressed but not significantly DE | 2.179749316 | | expressed but not significantly DE | expressed but not significantly DE |
| | | on.mrna.LG1.397.2 | | 1.283697382 | expressed but not significantly DE | expressed but not significantly DE | 2.160837999 | | expressed but not significantly DE | expressed but not significantly DE |
| | | Average | | 1.272706029 | | | 2.170293657 | | | |
| | | STDV | | 0.01554412 | | | 0.01337232 | | | |
| | | | | | | | | | | |
| | | | | | | | | | | |
| | | | | | | | | | | |
| Steroid receptors | | | | | | | | | | |
| | | | | | | | | | | |
| | | | | | | | | | | |
| arB | ENSONIG00000017538 | on.mrna.LG2.621.1 | expressed but not significantly DE | expressed but not significantly DE | | 2.578552391 | | 2.473384856 | expressed but not significantly DE | expressed but not significantly DE |
| | | on.mrna.LG2.621.2 | expressed but not significantly DE | expressed but not significantly DE | | 2.141185922 | | 1.904599058 | expressed but not significantly DE | expressed but not significantly DE |
| | | on.mrna.LG2.621.3 | expressed but not significantly DE | expressed but not significantly DE | | 2.154649123 | | 1.894032088 | expressed but not significantly DE | expressed but not significantly DE |
| | | | | | | 2.291462479 | | 2.090672001 | | |
| | | | | | | 0.24871827 | | 0.331481165 | | |
| | | | | | | | | | | |
| arA | ENSONIG00000012854 | on.mrna.UNK38.39.2 | 2.228511107 | | 4.048686017 | | expressed but not significantly DE | expressed but not significantly DE | 2.32604375 | |
| | | on.mrna.UNK38.39.1 | 2.24777927 | | 3.890075987 | | expressed but not significantly DE | expressed but not significantly DE | 2.114980354 | |
| | | on.mrna.UNK38.40.1 | 2.304338576 | | 3.057317134 | | / | / | / | / |
| | | Average | 2.260209651 | | 3.665359712 | | | | 2.220512052 | |
| | | STDV | 0.039412397 | | 0.532518656 | | | | 0.149244359 | |
| | | | | | | | | | | |
| esr1 | ENSONIG00000013354 | on.mrna.LG15.398.2 | 2.910349461 | | 3.298539018 | | expressed but not significantly DE | | 1.118966269 | |
| | | on.mrna.LG15.398.1 | 2.904670982 | | 3.29623483 | | expressed but not significantly DE | | 1.120575806 | |
| | | Average | 2.907510222 | | 3.297386924 | | | | 1.119771037 | |
| | | STDV | 0.004015291 | | 0.001629307 | | | | 0.001138115 | |
| | | | | | | | | | | |
| esr2a | ENSONIG00000005633 | on.mrna.LG15.109.2 | | expressed but not significantly DE | expressed but not significantly DE | | | expressed but not significantly DE | | expressed but not significantly DE |
| | | on.mrna.LG15.109.1 | | expressed but not significantly DE | expressed but not significantly DE | | | expressed but not significantly DE | | expressed but not significantly DE |
| | | | | | | | | | | |
| esr2b | ENSONIG00000001710 | on.mrna.LG19.754.3 | | expressed but not significantly DE | 2.368620799 | | | expressed but not significantly DE | expressed but not significantly DE | |
| | | on.mrna.LG19.754.1 | | expressed but not significantly DE | 2.36680117 | | | expressed but not significantly DE | expressed but not significantly DE | |
| | | on.mrna.LG19.754.2 | | 1.020285811 | 1.557103424 | | | 1.499666329 | | expressed but not significantly DE |
| | | on.mrna.LG19.754.4 | | expressed but not significantly DE | 1.675127303 | | | 1.402119795 | | expressed but not significantly DE |
| | | Average | | | 1.991913174 | | | 1.450893062 | | |
| | | STDV | | | 0.436601439 | | | 0.068975816 | | |
| | | | | | | | | | | |
| gperA | ENSONIG00000020935 | on.mrna.LG4.590.1 | / | / | / | / | / | / | / | / |
| | | | | | | | | | | |
| gperB | ENSONIG00000021208 | not in dataset | | | | | | | | |

## Supplementary Material Table 5

Percentage of shared and unique transcripts in each species

| | *A.burtoni* | | *O.ventralis* | | *J.ornatus* | | *E.cyanostictus* | | Mean percentage | Standard Deviation |
|---|---|---|---|---|---|---|---|---|---|---|
| **Testis-Over-expressed** | Total no transcripts | percentage transcripts | Total no transcripts | percentage transcripts | Total no transcripts | percentage transcripts | Total no transcripts | percentage transcripts | | |
| total transcripts | 17875 | | 18290 | | 16820 | | 19114 | | | |
| expressed in one species | 2118 | 11.85 | 2418 | 13.22 | 2574 | 15.30 | 2863 | 14.98 | 13.8 | 1.6 |
| shared with only one species | 3482 | 19.48 | 3771 | 20.62 | 3058 | 18.18 | 3787 | 19.81 | | |
| shared with two species | 4642 | 25.97 | 4468 | 24.43 | 3555 | 21.14 | 4831 | 25.27 | | |
| expressed in four species | 7633 | 42.70 | 7633 | 41.73 | 7633 | 45.38 | 7633 | 39.93 | 42.4 | 2.3 |
| | | | | | | | | | | |
| **Ovary-Over-expressed** | | | | | | | | | | |
| total transcripts | 16178 | | 15820 | | 16387 | | 16258 | | | |
| expressed in one species | 1940 | 11.99 | 2076 | 13.12 | 2555 | 15.59 | 2427 | 14.93 | 13.9 | 1.6 |
| shared with only one species | 2969 | 18.35 | 2806 | 17.74 | 2776 | 16.94 | 2687 | 16.53 | | |
| shared with two species | 3546 | 21.92 | 3215 | 20.32 | 3333 | 20.34 | 3421 | 21.04 | | |
| expressed in four species | 7723 | 47.74 | 7723 | 48.82 | 7723 | 47.13 | 7723 | 47.50 | 47.8 | 0.7 |
| | | | | | | | | | | |
| **Male-Over-expressed** | | | | | | | | | | |
| total transcripts | 9525 | | 11327 | | 10630 | | 13194 | | | |
| expressed in one species | 1332 | 13.98 | 2099 | 18.53 | 2195 | 20.65 | 3060 | 23.19 | 19.1 | 3.9 |
| shared with only one species | 2151 | 22.58 | 2973 | 26.25 | 2395 | 22.53 | 3537 | 26.81 | | |
| shared with two species | 2819 | 29.60 | 3032 | 26.77 | 2817 | 26.50 | 3374 | 25.57 | | |
| expressed in four species | 3223 | 33.84 | 3223 | 28.45 | 3223 | 30.32 | 3223 | 24.43 | 29.3 | 3.9 |
| | | | | | | | | | | |
| **Female-Over-expressed** | | | | | | | | | | |
| total transcripts | 7910 | | 8140 | | 10198 | | 7428 | | | |
| expressed in one species | 1377 | 17.41 | 1523 | 18.71 | 2883 | 28.27 | 1507 | 20.29 | 21.2 | 4.9 |
| shared with only one species | 1694 | 21.42 | 1749 | 21.49 | 2324 | 22.79 | 1437 | 19.35 | | |
| shared with two species | 2067 | 26.13 | 2096 | 25.75 | 2219 | 21.76 | 1712 | 23.05 | | |
| expressed in four species | 2772 | 35.04 | 2772 | 34.05 | 2772 | 27.18 | 2772 | 37.32 | 33.4 | 4.4 |

**Supplementary Material Table 6**
logFC in expression for genes overexpressed in ovary/female tissues of all four species and belonging to the GO categories sex differentiation (diff) and sex determination (det)

| **Gene** | **GO** | **logFC *A. burtoni*** | **logFC *O. ventralis*** | **logFC *E. cyanostictus*** | **logFC *J. ornatus*** |
|---|---|---|---|---|---|
| apoptosis regulator bax | sex diff | 1.81 ± 0.04 ovary | 2.87 ± 0.01 ovary | 3.58 ± 0.01 ovary | 2.99 ± 0.00 ovary |
| bcl-2-related ovarian killer protein | sex diff | 5.34 ovary 2.81 females | 5.00 ovary 2.47 females | 5.60 ovary 2.97 females | 4.03 ovary 2.05 females |
| beta-n-acetylglucosaminyltransferase lunatic fringe-like | sex diff | 2.49 ± 0.10 ovary 1.34 ± 0.08 females | 2.24 ± 0.01 ovary 1.16 ± 0.20 females | 5.18 ± 0.15 ovary 2.57 ± 0.10 females | 3.67 ± 0.27 ovary 2.09± 0.10 females |
| bone morphogenetic protein 15 | sex diff | 7.56 ovary | 7.14 ovary | 7.66 ovary | 8.44 ovary |
| bone morphogenetic protein receptor type-1a | sex diff | 1.72 ± 0.00 ovary | 1.63 ± 0.00 ovary | 2.14 ± 0.00 ovary | 1.16 ± 0.00 ovary |
| cbp p300-interacting transactivator 3-like | sex det/diff | 5.36 ovary 2.56 females | 5.60 ovary 2.83 females | 4.70 ovary 2.22 females | 6.33 ovary 3.29 females |
| doublesex- and mab-3-related transcription factor 2 | sex det/diff | 2.78 ovary 2.51 females | 2.64 ovary 2.65 females | 3.87 ovary 2.42 females | 6.54 ovary 6.21 females |
| ensconsin | sex diff | 3.54 ± 0.20 ovary 1.80 ± 0.08 females | 2.92 ± 0.13 ovary 1.42 ± 0.05 females | 3.26 ± 0.23 ovary 1.54 ± 0.10 females | 3.31 ± 0.43 ovary 1.60 ± 0.18 females |
| forkhead box l2 | sex det/diff | 4.74 ovary | 2.70 ovary | 4.61 ovary | 5.73 ovary |
| forkhead box protein o3 | sex diff | 3.18 ± 0.01 ovary 1.41 ± 0.01 females | 3.35 ± 0.05 ovary 1.60 ± 0.03 females | 3.14 ± 0.05 ovary 1.43 ± 0.02 females | 2.94 ± 0.03 ovary 1.34 ± 0.02 females |
| frizzled-10 | sex diff | 3.74 ovary 1.99 females | 3.63 ovary 1.87 females | 3.80 ovary 1.76 females | 4.45 ovary 2.23 females |
| g1 s-specific cyclin-d1 | sex diff | 5.50 ± 0.02 ovary 2.88 ± 0.00 females | 4.86 ± 0.00 ovary 2.55 ± 0.00 females | 6.90 ± 0.00 ovary 3.50 ± 0.01 females | 6.61 ± 0.01 ovary 3.23 ± 0.01 females |
| g1 s-specific cyclin-d1 (other gene copy) | sex diff | 1.17 ovary | 2.41 ovary | 2.32 ovary | 1.28 ovary |
| hepatocyte growth factor receptor | sex diff | 2.64 ovary | 3.23 ovary | 1.21 ovary | 2.85 ovary |
| inhibitor of growth protein 2 | sex diff | 3.70 ovary 1.68 females | 4.47 ovary 2.26 females | 4.50 ovary 2.32 females | 5.39 ovary 2.77 females |
| mothers against decapentaplegic homolog 5 | sex diff | 3.16 ± 0.00 ovary | 2.95 ± 0.01 ovary | 2.75 ± 0.02 ovary | 2.83 ± 0.01 ovary |
| mothers against decapentaplegic homolog 9 | sex diff | 4.67 ± 0.74 ovary 2.56 ± 0.07 females | 3.05 ± 0.67 ovary 1.67 ± 0.07 females | 4.92 ± 1.46 ovary 2.70 ± 0.10 females | 4.62 ± 1.90 ovary 2.78 ± 0.05 females |
| peroxisomal multifunctional enzyme type 2 | sex diff | 2.90 ± 0.01 ovary | 1.81 ± 0.02 ovary | 3.20 ± 0.02 ovary | 4.31 ± 0.02 ovary |
| prohibitin | sex diff | 1.48 ± 0.01 ovary | 1.46 ± 0.01 ovary | 1.32 ± 0.01 ovary | 2.43 ± 0.01 ovary |
| ribonucleoside-diphosphate reductase large subunit | sex diff | 2.12 ± 0.01 ovary 1.14 ± 0.01 females | 2.62 ± 0.01 ovary 1.34 ± 0.01 females | 2.13 ± 0.00 ovary 1.05 ± 0.01 females | 1.81 ± 0.01 ovary 1.02 ± 0.00 females |
| sal-like protein 1 | sex diff | 4.46 ± 0.04 | 2.16 ± 0.01 | 1.33 ± 0.02 | 3.09 ± 0.02 |

| | | | | | |
|---|---|---|---|---|---|
| | | ovary | ovary | ovary | ovary |
| secreted frizzled-related protein 2 | sex diff | 6.47 ± 0.61 ovary | 6.48 ± 0.09 ovary | 7.04 ± 0.33 ovary | 5.21 ± 0.08 ovary |
| secreted frizzled-related protein 3 | sex diff | 3.40 ± 0.12 ovary<br>1.97 ± 0.09 females | 2.24 ± 0.05 ovary<br>1.34 ± 0.06 females | 3.91 ± 0.06 ovary<br>1.99 ± 0.07 females | 5.60 ± 0.10 ovary<br>2.99 ± 0.10 females |
| sox3-like | sex det/diff | 6.73 ovary<br>3.30 females | 5.60 ovary<br>2.64 females | 6.18 ovary<br>2.94 females | 8.31 ovary<br>4.16 females |
| tcdd-inducible polymerase | sex diff | 1.71 ± 0.10 ovary | 1.40 ± 0.05 ovary | 2.53 ± 0.27 ovary | 2.26 ± 0.09 ovary |
| transcription factor ap-2 gamma | sex diff | 6.06 ± 0.02 ovary<br>3.54 ± 0.04 females | 6.54 ± 0.04 ovary<br>3.81 ± 0.02 females | 6.35 ± 0.10 ovary<br>2.82 ± 0.02 females | 7.53 ± 0.05 ovary<br>3.64 ± 0.04 females |
| tyrosine-protein phosphatase non-receptor type 11 | sex diff | 2.12 ± 0.00 ovary<br>1.24 ± 0.00 females | 2.07 ± 0.02 ovary<br>1.11 ± 0.01 females | 3.57 ± 0.01 ovary<br>1.93 ± 0.01 females | 2.76 ± 0.00 ovary<br>1.27 ± 0.00 females |
| wnt-5a | sex diff | 4.02 ± 0.03 ovary<br>2.31 ± 0.00 females | 4.94 ± 0.09 ovary<br>2.35 ± 0.00 females | 4.48 ± 0.28 ovary<br>2.35 ± 0.02 females | 5.70 ± 0.17 ovary<br>2.87 ± 0.02 females |

**Supplementary Material Table 7**

logFC in expression for genes overexpressed in testis/male tissues of all four species and belonging to the GO categories sex differentiation (diff) and sex determination (det)

| **Gene** | **GO** | **logFC** ***A. burtoni*** | **logFC** ***O. ventralis*** | **logFC** ***E. cyanostictus*** | **logFC** ***J. ornatus*** |
|---|---|---|---|---|---|
| follicle stimulating hormone receptor | sex diff. | 2.01 testis | 3.56 testis | 2.43 testis | 1.87 testis |
| frizzled-6 | sex diff. | 1.71 ± 0.32 testis<br>1.02 males | 3.41 ± 0.07 testis<br>1.55 males | 3.0 ± 0.18 testis<br>1.39 males | 2.50 ± 0.02 testis<br>1.15 males |
| frizzled-7 | sex diff. | 1.65 testis | 1.32 testis | 2.14 testis | 2.33 testis |
| high mobility group protein b2 | sex diff. | 2.47 ± 0.02 testis<br>1.24 ± 0.01 males | 2.25 ± 0.002 testis<br>1.12 ± 0.005 males | 2.63 ± 0.004 testis<br>1.29 ± 0.001 males | 2.89 ± 0.01 testis<br>1.26 ± 0.01 males |
| high mobility group protein b2 (other gene copy) | sex diff. | 2.29 testis | 2.37 testis | 2.03 testis | 1.66 testis |
| histone | sex diff. | 1.07 ± 0.006 testis | 1.99 ± 0.002 testis | 1.45 ± 0.003 testis | 1.39 ± 0.00 testis |
| protein jagged-2 | sex diff. | 3.76 ± 0.01 testis<br>1.98 ± 0.003 males | 4.38 ± 0.18 testis<br>1.74 ± 0.07 males | 4.62 ± 0.01 testis<br>2.15 ± 0.01 males | 3.09 ± 0.003 testis<br>1.42 ± 0.01 males |
| kelch-like protein 10 | sex diff. | 11.54 testis<br>9.55 males | 7.49 testis<br>3.56 males | 6.21 testis<br>5.78 males | 10.32 testis<br>5.18 males |
| kelch-like protein 10 (other gene copy) | sex diff. | 10.95 testis<br>5.55 males | 7.14 testis<br>3.82 males | 5.36 testis<br>3.51 males | 10.02 testis<br>4.93 males |
| long-chain-fatty-acid-- ligase acsbg2 | sex diff. | 2.11 males | 1.87 males | 1.23 males | 2.31 males |
| mast stem cell growth factor receptor kit | sex diff. | 5.01 ± 0.07 testis | 3.08 ± 0.00 testis | 4.49 ± 0.00 testis | 1.74 ± 0.004 testis |
| meiotic recombination protein dmc1 lim15 homolog | sex diff. | 8.67 testis<br>8.03 males | 6.82 testis<br>4.12 males | 3.96 testis<br>2.74 males | 4.38 testis<br>2.39 males |
| mitogen-activated protein kinase kinase kinase 4 | sex det. | 1.76 ± 0.08 testis | 1.93 ± 0.07 testis | 3.24 ± 0.03 testis | 2.67 ± 0.08 testis |
| nipped-b-like protein | sex diff. | 3.59 ± 0.04 testis | 2.31 ± 0.01 testis | 1.75 ± 0.004 testis | 2.44 ± 0.02 testis |
| nuclear receptor coactivator 2 | sex diff. | 3.1 testis<br>1.59 males | 2.96 testis<br>1.5 males | 3.84 testis<br>1.8 males | 3.09 testis<br>1.39 males |
| polyadenylate-binding protein 2 | sex diff. | 1.35 ± 0.19 testis | 1.48 ± 0.14 testis | 1.67 ± 0.18 testis | 1.72 ± 0.14 testis |
| retinol dehydrogenase 10 | sex diff. | 2.41 ± 0.02 testis | 2.05 ± 0.02 testis | 1.4 ± 0.02 testis | 2.18 ± 0.015 testis |
| tilb homolog | sex diff. | 3.39 ± 0.55 testis<br>1.74 ± 0.3 males | 3.2 ± 0.03 testis<br>2.15 ± 0.16 males | 2.37 ± 0.34 testis<br>1.26 ± 0.15 males | 9.27 ± 0.19 testis<br>4.58 ± 0.07 males |
| transcription factor 7-like 2-like | sex diff. | 3.34 ± 0.43 testis<br>1.57 ± 0.2 males | 4.83 ± 0.17 testis<br>2.29 ± 0.05 males | 4.59 ± 0.15 testis<br>2.19 ± 0.02 males | 4.53 ± 0.2 testis<br>2.16 ± 0.11 males |
| transcription factor 7-like 2-like (other gene copy) | sex diff. | 2.88 ± 0.1 testis | 2.06 ± 0.09 testis | 2.60 ± 0.06 testis | 1.09 ± 0.04 testis |
| transforming growth factor beta-2 | sex diff. | 1.64 testis | 3.34 testis | 3.55 testis | 3.30 testis |

## Supplementary Material Table 8

**Enriched in Females in all four species**

| GO-ID | Term | FDR | P-Value | #FTranscripts | #Mtranscripts | Enriched in |
|---|---|---|---|---|---|---|
| GO:0031532 | actin cytoskeleton reorganization | 3.20E-16 | 1.87E-19 | 86 | 6 | FemaleAllSpecies |
| GO:0042632 | cholesterol homeostasis | 2.89E-14 | 4.40E-17 | 55 | 0 | FemaleAllSpecies |
| GO:0009395 | phospholipid catabolic process | 1.65E-12 | 5.96E-15 | 48 | 0 | FemaleAllSpecies |
| GO:0034375 | high-density lipoprotein particle remodeling | 1.65E-12 | 5.96E-15 | 48 | 0 | FemaleAllSpecies |
| GO:0043691 | reverse cholesterol transport | 1.65E-12 | 5.96E-15 | 48 | 0 | FemaleAllSpecies |
| GO:0045218 | zonula adherens maintenance | 1.55E-11 | 9.81E-14 | 44 | 0 | FemaleAllSpecies |
| GO:0090136 | epithelial cell-cell adhesion | 1.55E-11 | 9.81E-14 | 44 | 0 | FemaleAllSpecies |
| GO:0006828 | manganese ion transport | 2.68E-11 | 1.98E-13 | 43 | 0 | FemaleAllSpecies |
| GO:0032472 | Golgi calcium ion transport | 2.68E-11 | 1.98E-13 | 43 | 0 | FemaleAllSpecies |
| GO:0032468 | Golgi calcium ion homeostasis | 2.68E-11 | 1.98E-13 | 43 | 0 | FemaleAllSpecies |
| GO:0030026 | cellular manganese ion homeostasis | 2.68E-11 | 1.98E-13 | 43 | 0 | FemaleAllSpecies |
| GO:0010983 | positive regulation of high-density lipoprotein particle clearance | 8.45E-11 | 8.00E-13 | 41 | 0 | FemaleAllSpecies |
| GO:0032376 | positive regulation of cholesterol transport | 8.45E-11 | 8.00E-13 | 41 | 0 | FemaleAllSpecies |
| GO:0051764 | actin crosslink formation | 1.51E-10 | 1.61E-12 | 40 | 0 | FemaleAllSpecies |
| GO:0016339 | calcium-dependent cell-cell adhesion | 3.86E-10 | 4.47E-12 | 43 | 1 | FemaleAllSpecies |
| GO:0051017 | actin filament bundle assembly | 8.46E-08 | 2.19E-09 | 60 | 11 | FemaleAllSpecies |
| GO:0055091 | phospholipid homeostasis | 2.61E-07 | 8.41E-09 | 41 | 4 | FemaleAllSpecies |
| GO:0036336 | dendritic cell migration | 7.32E-07 | 2.83E-08 | 26 | 0 | FemaleAllSpecies |
| GO:0051016 | barbed-end actin filament capping | 7.32E-07 | 2.83E-08 | 26 | 0 | FemaleAllSpecies |
| GO:0043123 | positive regulation of I-kappaB kinase/NF-kappaB cascade | 7.41E-07 | 2.88E-08 | 67 | 17 | FemaleAllSpecies |
| GO:0070588 | calcium ion transmembrane transport | 2.18E-06 | 9.35E-08 | 70 | 20 | FemaleAllSpecies |
| GO:0050746 | regulation of lipoprotein metabolic process | 2.76E-06 | 1.23E-07 | 31 | 2 | FemaleAllSpecies |
| GO:0048149 | behavioral response to ethanol | 8.23E-06 | 3.98E-07 | 26 | 1 | FemaleAllSpecies |
| GO:0010977 | negative regulation of neuron projection development | 8.92E-06 | 4.38E-07 | 29 | 2 | FemaleAllSpecies |
| GO:0035046 | pronuclear migration | 9.30E-06 | 4.60E-07 | 22 | 0 | FemaleAllSpecies |
| GO:0001522 | pseudouridine synthesis | 3.24E-05 | 1.85E-06 | 20 | 0 | FemaleAllSpecies |
| GO:0070358 | actin polymerization-dependent cell motility | 4.83E-05 | 2.90E-06 | 26 | 2 | FemaleAllSpecies |
| GO:0007004 | telomere maintenance via telomerase | 1.10E-04 | 7.45E-06 | 18 | 0 | FemaleAllSpecies |
| GO:0006636 | unsaturated fatty acid biosynthetic process | 3.68E-04 | 3.00E-05 | 16 | 0 | FemaleAllSpecies |
| GO:2000114 | regulation of establishment of cell polarity | 3.68E-04 | 3.00E-05 | 16 | 0 | FemaleAllSpecies |
| GO:0009262 | deoxyribonucleotide metabolic process | 6.71E-04 | 6.01E-05 | 15 | 0 | FemaleAllSpecies |
| GO:0071803 | positive regulation of podosome assembly | 1.23E-03 | 1.20E-04 | 14 | 0 | FemaleAllSpecies |
| GO:0051382 | kinetochore assembly | 1.23E-03 | 1.20E-04 | 14 | 0 | FemaleAllSpecies |
| GO:0043486 | histone exchange | 1.23E-03 | 1.20E-04 | 14 | 0 | FemaleAllSpecies |
| GO:0010458 | exit from mitosis | 1.85E-03 | 1.88E-04 | 28 | 6 | FemaleAllSpecies |
| GO:0060216 | definitive hemopoiesis | 2.09E-03 | 2.17E-04 | 19 | 2 | FemaleAllSpecies |
| GO:0090303 | positive regulation of wound healing | 2.09E-03 | 2.17E-04 | 19 | 2 | FemaleAllSpecies |
| GO:0001947 | heart looping | 2.24E-03 | 2.41E-04 | 75 | 36 | FemaleAllSpecies |
| GO:0035413 | positive regulation of catenin import into nucleus | 2.24E-03 | 2.41E-04 | 13 | 0 | FemaleAllSpecies |
| GO:0016266 | O-glycan processing | 2.24E-03 | 2.41E-04 | 13 | 0 | FemaleAllSpecies |
| GO:0001942 | hair follicle development | 2.46E-03 | 2.70E-04 | 16 | 1 | FemaleAllSpecies |
| GO:0060444 | branching involved in mammary gland duct morphogenesis | 3.47E-03 | 3.95E-04 | 18 | 2 | FemaleAllSpecies |
| GO:2001235 | positive regulation of apoptotic signaling pathway | 3.98E-03 | 4.83E-04 | 12 | 0 | FemaleAllSpecies |
| GO:0006081 | cellular aldehyde metabolic process | 3.98E-03 | 4.83E-04 | 12 | 0 | FemaleAllSpecies |
| GO:0007077 | mitotic nuclear envelope disassembly | 4.17E-03 | 5.11E-04 | 15 | 1 | FemaleAllSpecies |
| GO:0072655 | establishment of protein localization to mitochondrion | 4.17E-03 | 5.11E-04 | 15 | 1 | FemaleAllSpecies |
| GO:0030216 | keratinocyte differentiation | 4.24E-03 | 5.21E-04 | 22 | 4 | FemaleAllSpecies |
| GO:0016525 | negative regulation of angiogenesis | 6.20E-03 | 7.97E-04 | 27 | 7 | FemaleAllSpecies |
| GO:0048284 | organelle fusion | 6.20E-03 | 7.97E-04 | 27 | 7 | FemaleAllSpecies |
| GO:0045604 | regulation of epidermal cell differentiation | 6.41E-03 | 8.39E-04 | 19 | 3 | FemaleAllSpecies |
| GO:0009409 | response to cold | 6.41E-03 | 8.91E-04 | 21 | 4 | FemaleAllSpecies |
| GO:0006766 | vitamin metabolic process | 6.41E-03 | 8.91E-04 | 21 | 4 | FemaleAllSpecies |
| GO:0060391 | positive regulation of SMAD protein import into nucleus | 6.41E-03 | 8.91E-04 | 21 | 4 | FemaleAllSpecies |
| GO:0007064 | mitotic sister chromatid cohesion | 6.66E-03 | 9.65E-04 | 14 | 1 | FemaleAllSpecies |
| GO:0007039 | vacuolar protein catabolic process | 6.66E-03 | 9.68E-04 | 11 | 0 | FemaleAllSpecies |
| GO:0010825 | positive regulation of centrosome duplication | 6.66E-03 | 9.68E-04 | 11 | 0 | FemaleAllSpecies |
| GO:0060775 | planar cell polarity pathway involved in gastrula mediolateral intercalation | 6.66E-03 | 9.68E-04 | 11 | 0 | FemaleAllSpecies |
| GO:0034354 | 'de novo' NAD biosynthetic process from tryptophan | 6.66E-03 | 9.68E-04 | 11 | 0 | FemaleAllSpecies |
| GO:0043654 | recognition of apoptotic cell | 6.66E-03 | 9.68E-04 | 11 | 0 | FemaleAllSpecies |
| GO:0019441 | tryptophan catabolic process to kynurenine | 6.66E-03 | 9.68E-04 | 11 | 0 | FemaleAllSpecies |
| GO:0007498 | mesoderm development | 8.21E-03 | 1.21E-03 | 33 | 11 | FemaleAllSpecies |
| GO:0009112 | nucleobase metabolic process | 9.26E-03 | 1.40E-03 | 24 | 6 | FemaleAllSpecies |
| GO:0010862 | positive regulation of pathway-restricted SMAD protein phosphorylation | 9.71E-03 | 1.48E-03 | 22 | 5 | FemaleAllSpecies |
| GO:0001569 | patterning of blood vessels | 1.12E-02 | 1.81E-03 | 13 | 1 | FemaleAllSpecies |
| GO:0009311 | oligosaccharide metabolic process | 1.17E-02 | 1.94E-03 | 10 | 0 | FemaleAllSpecies |
| GO:0033344 | cholesterol efflux | 1.17E-02 | 1.94E-03 | 10 | 0 | FemaleAllSpecies |
| GO:0043097 | pyrimidine nucleoside salvage | 1.17E-02 | 1.94E-03 | 10 | 0 | FemaleAllSpecies |
| GO:0032786 | positive regulation of DNA-dependent transcription, elongation | 1.17E-02 | 1.94E-03 | 10 | 0 | FemaleAllSpecies |
| GO:0071379 | cellular response to prostaglandin stimulus | 1.17E-02 | 1.94E-03 | 10 | 0 | FemaleAllSpecies |
| GO:0032415 | regulation of sodium:hydrogen antiporter activity | 1.17E-02 | 1.94E-03 | 10 | 0 | FemaleAllSpecies |
| GO:0046503 | glycerolipid catabolic process | 1.17E-02 | 1.94E-03 | 10 | 0 | FemaleAllSpecies |
| GO:2000370 | positive regulation of clathrin-mediated endocytosis | 1.17E-02 | 1.94E-03 | 10 | 0 | FemaleAllSpecies |
| GO:0045909 | positive regulation of vasodilation | 1.17E-02 | 1.94E-03 | 10 | 0 | FemaleAllSpecies |
| GO:0048490 | anterograde synaptic vesicle transport | 1.17E-02 | 1.94E-03 | 10 | 0 | FemaleAllSpecies |
| GO:0003151 | outflow tract morphogenesis | 1.17E-02 | 1.94E-03 | 10 | 0 | FemaleAllSpecies |
| GO:2000052 | positive regulation of non-canonical Wnt receptor signaling pathway | 1.17E-02 | 1.94E-03 | 10 | 0 | FemaleAllSpecies |
| GO:0014033 | neural crest cell differentiation | 1.44E-02 | 2.45E-03 | 21 | 5 | FemaleAllSpecies |
| GO:0001937 | negative regulation of endothelial cell proliferation | 1.91E-02 | 3.39E-03 | 12 | 1 | FemaleAllSpecies |
| GO:0006654 | phosphatidic acid biosynthetic process | 1.91E-02 | 3.39E-03 | 12 | 1 | FemaleAllSpecies |
| GO:0032456 | endocytic recycling | 1.91E-02 | 3.39E-03 | 12 | 1 | FemaleAllSpecies |
| GO:0090263 | positive regulation of canonical Wnt receptor signaling pathway | 1.91E-02 | 3.39E-03 | 12 | 1 | FemaleAllSpecies |
| GO:0006691 | leukotriene metabolic process | 2.07E-02 | 3.88E-03 | 9 | 0 | FemaleAllSpecies |
| GO:0060029 | convergent extension involved in organogenesis | 2.07E-02 | 3.88E-03 | 9 | 0 | FemaleAllSpecies |
| GO:0006516 | glycoprotein catabolic process | 2.07E-02 | 3.88E-03 | 9 | 0 | FemaleAllSpecies |
| GO:0009191 | ribonucleoside diphosphate catabolic process | 2.07E-02 | 3.88E-03 | 9 | 0 | FemaleAllSpecies |
| GO:0038092 | nodal signaling pathway | 2.07E-02 | 3.88E-03 | 9 | 0 | FemaleAllSpecies |
| GO:0006435 | threonyl-tRNA aminoacylation | 2.07E-02 | 3.88E-03 | 9 | 0 | FemaleAllSpecies |
| GO:0005978 | glycogen biosynthetic process | 2.07E-02 | 3.88E-03 | 9 | 0 | FemaleAllSpecies |
| GO:0035024 | negative regulation of Rho protein signal transduction | 2.07E-02 | 3.88E-03 | 9 | 0 | FemaleAllSpecies |
| GO:2000393 | negative regulation of lamellipodium morphogenesis | 2.07E-02 | 3.88E-03 | 9 | 0 | FemaleAllSpecies |
| GO:0090174 | organelle membrane fusion | 2.07E-02 | 3.88E-03 | 9 | 0 | FemaleAllSpecies |
| GO:0006044 | N-acetylglucosamine metabolic process | 2.07E-02 | 3.88E-03 | 9 | 0 | FemaleAllSpecies |
| GO:1900028 | negative regulation of ruffle assembly | 2.07E-02 | 3.88E-03 | 9 | 0 | FemaleAllSpecies |
| GO:1900025 | negative regulation of substrate adhesion-dependent cell spreading | 2.07E-02 | 3.88E-03 | 9 | 0 | FemaleAllSpecies |
| GO:0046485 | ether lipid metabolic process | 2.07E-02 | 3.88E-03 | 9 | 0 | FemaleAllSpecies |
| GO:0046386 | deoxyribose phosphate catabolic process | 2.07E-02 | 3.88E-03 | 9 | 0 | FemaleAllSpecies |

| | | | | | | |
|---|---|---|---|---|---|---|
| GO:0003190 | atrioventricular valve formation | 2.07E-02 | 3.88E-03 | 9 | 0 | FemaleAllSpecies |
| GO:2000095 | regulation of Wnt receptor signaling pathway, planar cell polarity pathway | 2.07E-02 | 3.88E-03 | 9 | 0 | FemaleAllSpecies |
| GO:0032060 | bleb assembly | 2.07E-02 | 3.88E-03 | 9 | 0 | FemaleAllSpecies |
| GO:2000053 | regulation of Wnt receptor signaling pathway involved in dorsal/ventral axis specification | 2.07E-02 | 3.88E-03 | 9 | 0 | FemaleAllSpecies |
| GO:0006907 | pinocytosis | 2.07E-02 | 3.88E-03 | 9 | 0 | FemaleAllSpecies |
| GO:0042993 | positive regulation of transcription factor import into nucleus | 2.07E-02 | 3.88E-03 | 9 | 0 | FemaleAllSpecies |
| GO:0051220 | cytoplasmic sequestering of protein | 2.18E-02 | 4.14E-03 | 14 | 2 | FemaleAllSpecies |
| GO:0045665 | negative regulation of neuron differentiation | 2.18E-02 | 4.14E-03 | 14 | 2 | FemaleAllSpecies |
| GO:0045732 | positive regulation of protein catabolic process | 3.01E-02 | 5.83E-03 | 21 | 6 | FemaleAllSpecies |
| GO:0008344 | adult locomotory behavior | 3.02E-02 | 5.86E-03 | 26 | 9 | FemaleAllSpecies |
| GO:0045454 | cell redox homeostasis | 3.20E-02 | 6.30E-03 | 11 | 1 | FemaleAllSpecies |
| GO:0060122 | inner ear receptor stereocilium organization | 3.47E-02 | 7.32E-03 | 13 | 2 | FemaleAllSpecies |
| GO:0090162 | establishment of epithelial cell polarity | 3.47E-02 | 7.32E-03 | 13 | 2 | FemaleAllSpecies |
| GO:0021846 | cell proliferation in forebrain | 3.47E-02 | 7.32E-03 | 13 | 2 | FemaleAllSpecies |
| GO:0001952 | regulation of cell-matrix adhesion | 3.50E-02 | 7.46E-03 | 15 | 3 | FemaleAllSpecies |
| GO:0000154 | rRNA modification | 3.56E-02 | 7.78E-03 | 8 | 0 | FemaleAllSpecies |
| GO:0001880 | Mullerian duct regression | 3.56E-02 | 7.78E-03 | 8 | 0 | FemaleAllSpecies |
| GO:0000083 | regulation of transcription involved in G1/S transition of mitotic cell cycle | 3.56E-02 | 7.78E-03 | 8 | 0 | FemaleAllSpecies |
| GO:0060065 | uterus development | 3.56E-02 | 7.78E-03 | 8 | 0 | FemaleAllSpecies |
| GO:0043088 | regulation of Cdc42 GTPase activity | 3.56E-02 | 7.78E-03 | 8 | 0 | FemaleAllSpecies |
| GO:0002138 | retinoic acid biosynthetic process | 3.56E-02 | 7.78E-03 | 8 | 0 | FemaleAllSpecies |
| GO:0042572 | retinol metabolic process | 3.56E-02 | 7.78E-03 | 8 | 0 | FemaleAllSpecies |
| GO:0071476 | cellular hypotonic response | 3.56E-02 | 7.78E-03 | 8 | 0 | FemaleAllSpecies |
| GO:0014029 | neural crest formation | 3.56E-02 | 7.78E-03 | 8 | 0 | FemaleAllSpecies |
| GO:0005980 | glycogen catabolic process | 3.56E-02 | 7.78E-03 | 8 | 0 | FemaleAllSpecies |
| GO:2000641 | regulation of early endosome to late endosome transport | 3.56E-02 | 7.78E-03 | 8 | 0 | FemaleAllSpecies |
| GO:0010878 | cholesterol storage | 3.56E-02 | 7.78E-03 | 8 | 0 | FemaleAllSpecies |
| GO:0000959 | mitochondrial RNA metabolic process | 3.56E-02 | 7.78E-03 | 8 | 0 | FemaleAllSpecies |
| GO:0006108 | malate metabolic process | 3.56E-02 | 7.78E-03 | 8 | 0 | FemaleAllSpecies |
| GO:0090222 | centrosome-templated microtubule nucleation | 3.56E-02 | 7.78E-03 | 8 | 0 | FemaleAllSpecies |
| GO:0002943 | tRNA dihydrouridine synthesis | 3.56E-02 | 7.78E-03 | 8 | 0 | FemaleAllSpecies |
| GO:0003303 | BMP signaling pathway involved in heart jogging | 3.56E-02 | 7.78E-03 | 8 | 0 | FemaleAllSpecies |
| GO:0046326 | positive regulation of glucose import | 3.56E-02 | 7.78E-03 | 8 | 0 | FemaleAllSpecies |
| GO:0070328 | triglyceride homeostasis | 3.56E-02 | 7.78E-03 | 8 | 0 | FemaleAllSpecies |
| GO:0051084 | 'de novo' posttranslational protein folding | 3.56E-02 | 7.78E-03 | 8 | 0 | FemaleAllSpecies |
| GO:0043467 | regulation of generation of precursor metabolites and energy | 3.56E-02 | 7.78E-03 | 8 | 0 | FemaleAllSpecies |
| GO:0048263 | determination of dorsal identity | 3.56E-02 | 7.78E-03 | 8 | 0 | FemaleAllSpecies |
| GO:0071453 | cellular response to oxygen levels | 4.01E-02 | 8.87E-03 | 25 | 9 | FemaleAllSpecies |
| GO:1901214 | regulation of neuron death | 4.54E-02 | 1.01E-02 | 38 | 18 | FemaleAllSpecies |
| GO:1901264 | carbohydrate derivative transport | 4.69E-02 | 1.05E-02 | 18 | 5 | FemaleAllSpecies |
| GO:0007006 | mitochondrial membrane organization | 4.69E-02 | 1.05E-02 | 18 | 5 | FemaleAllSpecies |
| GO:0051402 | neuron apoptotic process | 4.76E-02 | 1.07E-02 | 35 | 16 | FemaleAllSpecies |

**Enriched in Male Tissue in all four species**

| GO-ID | Term | FDR | P-Value | #FTranscripts | #Mtranscripts | Enriched in |
|---|---|---|---|---|---|---|
| GO:0044333 | Wnt receptor signaling pathway involved in digestive tract morphogenesis | 3.94E-10 | 4.84E-12 | 0 | 37 | MaleAllSpecies |
| GO:0060729 | intestinal epithelial structure maintenance | 3.94E-10 | 4.84E-12 | 0 | 37 | MaleAllSpecies |
| GO:0045716 | positive regulation of low-density lipoprotein particle receptor biosynthetic process | 1.72E-08 | 3.37E-10 | 0 | 31 | MaleAllSpecies |
| GO:0072369 | regulation of lipid transport by positive regulation of transcription from RNA polymerase II promoter | 1.72E-08 | 3.37E-10 | 0 | 31 | MaleAllSpecies |
| GO:0031641 | regulation of myelination | 3.07E-08 | 6.84E-10 | 0 | 30 | MaleAllSpecies |
| GO:0006977 | DNA damage response, signal transduction by p53 class mediator resulting in cell cycle arrest | 3.07E-08 | 6.84E-10 | 0 | 30 | MaleAllSpecies |
| GO:0019886 | antigen processing and presentation of exogenous peptide antigen via MHC class II | 4.67E-08 | 1.12E-09 | 3 | 40 | MaleAllSpecies |
| GO:0048660 | regulation of smooth muscle cell proliferation | 7.98E-08 | 2.03E-09 | 2 | 36 | MaleAllSpecies |
| GO:0009791 | post-embryonic development | 8.32E-08 | 2.13E-09 | 3 | 39 | MaleAllSpecies |
| GO:0030538 | embryonic genitalia morphogenesis | 1.84E-07 | 5.69E-09 | 0 | 27 | MaleAllSpecies |
| GO:0010909 | positive regulation of heparan sulfate proteoglycan biosynthetic process | 1.84E-07 | 5.69E-09 | 0 | 27 | MaleAllSpecies |
| GO:0046827 | positive regulation of protein export from nucleus | 1.84E-07 | 5.69E-09 | 0 | 27 | MaleAllSpecies |
| GO:2000675 | negative regulation of type B pancreatic cell apoptotic process | 1.84E-07 | 5.69E-09 | 0 | 27 | MaleAllSpecies |
| GO:0046621 | negative regulation of organ growth | 1.84E-07 | 5.69E-09 | 0 | 27 | MaleAllSpecies |
| GO:0044334 | canonical Wnt receptor signaling pathway involved in positive regulation of epithelial to mesenchymal transition | 1.84E-07 | 5.69E-09 | 0 | 27 | MaleAllSpecies |
| GO:0048625 | myoblast fate commitment | 1.84E-07 | 5.69E-09 | 0 | 27 | MaleAllSpecies |
| GO:0032252 | secretory granule localization | 1.84E-07 | 5.69E-09 | 0 | 27 | MaleAllSpecies |
| GO:0043570 | maintenance of DNA repeat elements | 1.84E-07 | 5.69E-09 | 0 | 27 | MaleAllSpecies |
| GO:0021549 | cerebellum development | 1.88E-07 | 5.90E-09 | 1 | 31 | MaleAllSpecies |
| GO:1902165 | regulation of intrinsic apoptotic signaling pathway in response to DNA damage by p53 class mediator | 1.88E-07 | 5.90E-09 | 1 | 31 | MaleAllSpecies |
| GO:0032481 | positive regulation of type I interferon production | 3.41E-07 | 1.15E-08 | 0 | 26 | MaleAllSpecies |
| GO:0030282 | bone mineralization | 7.24E-07 | 2.78E-08 | 2 | 32 | MaleAllSpecies |
| GO:0010988 | regulation of low-density lipoprotein particle clearance | 1.32E-06 | 5.31E-08 | 2 | 31 | MaleAllSpecies |
| GO:0031016 | pancreas development | 4.16E-06 | 1.90E-07 | 5 | 37 | MaleAllSpecies |
| GO:0000122 | negative regulation of transcription from RNA polymerase II promoter | 5.49E-06 | 2.55E-07 | 40 | 99 | MaleAllSpecies |
| GO:0042475 | odontogenesis of dentin-containing tooth | 7.21E-06 | 3.41E-07 | 2 | 36 | MaleAllSpecies |
| GO:0048713 | regulation of oligodendrocyte differentiation | 7.71E-06 | 3.68E-07 | 2 | 28 | MaleAllSpecies |
| GO:0006386 | termination of RNA polymerase III transcription | 8.12E-06 | 3.92E-07 | 0 | 21 | MaleAllSpecies |
| GO:0006385 | transcription elongation from RNA polymerase III promoter | 8.12E-06 | 3.92E-07 | 0 | 21 | MaleAllSpecies |
| GO:0050728 | negative regulation of inflammatory response | 8.12E-06 | 3.92E-07 | 0 | 21 | MaleAllSpecies |
| GO:0060291 | long-term synaptic potentiation | 1.30E-05 | 6.55E-07 | 1 | 24 | MaleAllSpecies |
| GO:0045599 | negative regulation of fat cell differentiation | 1.37E-05 | 6.98E-07 | 2 | 27 | MaleAllSpecies |
| GO:0048619 | embryonic hindgut morphogenesis | 1.37E-05 | 6.98E-07 | 2 | 27 | MaleAllSpecies |
| GO:0048557 | embryonic digestive tract morphogenesis | 1.37E-05 | 6.98E-07 | 2 | 27 | MaleAllSpecies |
| GO:0048208 | COPII vesicle coating | 1.51E-05 | 7.93E-07 | 0 | 20 | MaleAllSpecies |
| GO:0045494 | photoreceptor cell maintenance | 1.51E-05 | 7.93E-07 | 0 | 20 | MaleAllSpecies |
| GO:0035083 | cilium axoneme assembly | 2.43E-05 | 1.32E-06 | 2 | 26 | MaleAllSpecies |
| GO:0030514 | negative regulation of BMP signaling pathway | 2.78E-05 | 1.56E-06 | 4 | 31 | MaleAllSpecies |
| GO:0021983 | pituitary gland development | 2.78E-05 | 1.56E-06 | 4 | 31 | MaleAllSpecies |
| GO:0000381 | regulation of alternative mRNA splicing, via spliceosome | 2.85E-05 | 1.60E-06 | 0 | 19 | MaleAllSpecies |
| GO:0046777 | protein autophosphorylation | 3.41E-05 | 1.96E-06 | 15 | 53 | MaleAllSpecies |
| GO:0002474 | antigen processing and presentation of peptide antigen via MHC class I | 4.22E-05 | 2.48E-06 | 1 | 22 | MaleAllSpecies |
| GO:0040018 | positive regulation of multicellular organism growth | 5.34E-05 | 3.24E-06 | 0 | 18 | MaleAllSpecies |
| GO:0006114 | glycerol biosynthetic process | 5.34E-05 | 3.24E-06 | 0 | 18 | MaleAllSpecies |
| GO:0000184 | nuclear-transcribed mRNA catabolic process, nonsense-mediated decay | 7.52E-05 | 4.71E-06 | 2 | 24 | MaleAllSpecies |
| GO:0030198 | extracellular matrix organization | 7.62E-05 | 4.82E-06 | 1 | 21 | MaleAllSpecies |
| GO:0007612 | learning | 1.29E-04 | 8.84E-06 | 2 | 23 | MaleAllSpecies |
| GO:0007224 | smoothened signaling pathway | 1.76E-04 | 1.24E-05 | 7 | 34 | MaleAllSpecies |
| GO:2001237 | negative regulation of extrinsic apoptotic signaling pathway | 1.78E-04 | 1.25E-05 | 8 | 36 | MaleAllSpecies |
| GO:0000289 | nuclear-transcribed mRNA poly(A) tail shortening | 1.82E-04 | 1.33E-05 | 0 | 16 | MaleAllSpecies |
| GO:0000413 | protein peptidyl-prolyl isomerization | 1.82E-04 | 1.33E-05 | 0 | 16 | MaleAllSpecies |
| GO:0032350 | regulation of hormone metabolic process | 2.17E-04 | 1.62E-05 | 4 | 27 | MaleAllSpecies |
| GO:0040037 | negative regulation of fibroblast growth factor receptor signaling pathway | 2.45E-04 | 1.87E-05 | 5 | 29 | MaleAllSpecies |
| GO:0061178 | regulation of insulin secretion involved in cellular response to glucose stimulus | 2.71E-04 | 2.10E-05 | 8 | 35 | MaleAllSpecies |
| GO:0030866 | cortical actin cytoskeleton organization | 3.33E-04 | 2.68E-05 | 0 | 15 | MaleAllSpecies |

| | | | | | | |
|---|---|---|---|---|---|---|
| GO:2000300 | regulation of synaptic vesicle exocytosis | 3.33E-04 | 2.68E-05 | 0 | 15 | MaleAllSpecies |
| GO:0006002 | fructose 6-phosphate metabolic process | 3.33E-04 | 2.68E-05 | 0 | 15 | MaleAllSpecies |
| GO:0051149 | positive regulation of muscle cell differentiation | 3.33E-04 | 2.68E-05 | 0 | 15 | MaleAllSpecies |
| GO:0021766 | hippocampus development | 3.78E-04 | 3.09E-05 | 2 | 21 | MaleAllSpecies |
| GO:0014003 | oligodendrocyte development | 4.19E-04 | 3.48E-05 | 6 | 30 | MaleAllSpecies |
| GO:0045055 | regulated secretory pathway | 4.98E-04 | 4.23E-05 | 3 | 23 | MaleAllSpecies |
| GO:0048011 | neurotrophin TRK receptor signaling pathway | 5.53E-04 | 4.74E-05 | 23 | 59 | MaleAllSpecies |
| GO:0051877 | pigment granule aggregation in cell center | 6.19E-04 | 5.41E-05 | 0 | 14 | MaleAllSpecies |
| GO:0048791 | calcium ion-dependent exocytosis of neurotransmitter | 6.19E-04 | 5.41E-05 | 0 | 14 | MaleAllSpecies |
| GO:0045931 | positive regulation of mitotic cell cycle | 6.19E-04 | 5.41E-05 | 0 | 14 | MaleAllSpecies |
| GO:0032000 | positive regulation of fatty acid beta-oxidation | 6.19E-04 | 5.41E-05 | 0 | 14 | MaleAllSpecies |
| GO:0032024 | positive regulation of insulin secretion | 6.69E-04 | 5.93E-05 | 6 | 29 | MaleAllSpecies |
| GO:0051592 | response to calcium ion | 6.70E-04 | 5.96E-05 | 7 | 31 | MaleAllSpecies |
| GO:0048015 | phosphatidylinositol-mediated signaling | 6.75E-04 | 6.07E-05 | 13 | 42 | MaleAllSpecies |
| GO:0016458 | gene silencing | 7.39E-04 | 6.71E-05 | 1 | 17 | MaleAllSpecies |
| GO:0017156 | calcium ion-dependent exocytosis | 8.34E-04 | 7.64E-05 | 3 | 22 | MaleAllSpecies |
| GO:0043433 | negative regulation of sequence-specific DNA binding transcription factor activity | 1.03E-03 | 9.50E-05 | 13 | 41 | MaleAllSpecies |
| GO:0000086 | G2/M transition of mitotic cell cycle | 1.03E-03 | 9.57E-05 | 8 | 32 | MaleAllSpecies |
| GO:0006533 | aspartate catabolic process | 1.13E-03 | 1.09E-04 | 0 | 13 | MaleAllSpecies |
| GO:0016188 | synaptic vesicle maturation | 1.13E-03 | 1.09E-04 | 0 | 13 | MaleAllSpecies |
| GO:0010881 | regulation of cardiac muscle contraction by regulation of the release of sequestered calcium ion | 1.13E-03 | 1.09E-04 | 0 | 13 | MaleAllSpecies |
| GO:0042073 | intraflagellar transport | 1.13E-03 | 1.09E-04 | 0 | 13 | MaleAllSpecies |
| GO:0050893 | sensory processing | 1.13E-03 | 1.09E-04 | 0 | 13 | MaleAllSpecies |
| GO:0035845 | photoreceptor cell outer segment organization | 1.13E-03 | 1.09E-04 | 0 | 13 | MaleAllSpecies |
| GO:0016485 | protein processing | 1.35E-03 | 1.33E-04 | 10 | 35 | MaleAllSpecies |
| GO:0001938 | positive regulation of endothelial cell proliferation | 1.38E-03 | 1.37E-04 | 3 | 21 | MaleAllSpecies |
| GO:0006333 | chromatin assembly or disassembly | 1.38E-03 | 1.37E-04 | 3 | 21 | MaleAllSpecies |
| GO:0048169 | regulation of long-term neuronal synaptic plasticity | 1.56E-03 | 1.58E-04 | 4 | 23 | MaleAllSpecies |
| GO:0070373 | negative regulation of ERK1 and ERK2 cascade | 1.63E-03 | 1.65E-04 | 7 | 29 | MaleAllSpecies |
| GO:0019233 | sensory perception of pain | 1.91E-03 | 1.97E-04 | 2 | 18 | MaleAllSpecies |
| GO:0061001 | regulation of dendritic spine morphogenesis | 1.91E-03 | 1.97E-04 | 2 | 18 | MaleAllSpecies |
| GO:0030512 | negative regulation of transforming growth factor beta receptor signaling pathway | 2.09E-03 | 2.21E-04 | 0 | 12 | MaleAllSpecies |
| GO:0046686 | response to cadmium ion | 2.09E-03 | 2.21E-04 | 0 | 12 | MaleAllSpecies |
| GO:0061014 | positive regulation of mRNA catabolic process | 2.09E-03 | 2.21E-04 | 0 | 12 | MaleAllSpecies |
| GO:0007032 | endosome organization | 2.28E-03 | 2.46E-04 | 1 | 15 | MaleAllSpecies |
| GO:0007528 | neuromuscular junction development | 2.28E-03 | 2.46E-04 | 1 | 15 | MaleAllSpecies |
| GO:0032869 | cellular response to insulin stimulus | 2.30E-03 | 2.49E-04 | 30 | 65 | MaleAllSpecies |
| GO:0007608 | sensory perception of smell | 3.20E-03 | 3.61E-04 | 2 | 17 | MaleAllSpecies |
| GO:0009636 | response to toxic substance | 3.20E-03 | 3.61E-04 | 2 | 17 | MaleAllSpecies |
| GO:0032092 | positive regulation of protein binding | 3.61E-03 | 4.15E-04 | 8 | 29 | MaleAllSpecies |
| GO:0002437 | inflammatory response to antigenic stimulus | 3.76E-03 | 4.46E-04 | 0 | 11 | MaleAllSpecies |
| GO:0021591 | ventricular system development | 3.76E-03 | 4.46E-04 | 0 | 11 | MaleAllSpecies |
| GO:0070098 | chemokine-mediated signaling pathway | 3.76E-03 | 4.46E-04 | 0 | 11 | MaleAllSpecies |
| GO:0060059 | embryonic retina morphogenesis in camera-type eye | 3.76E-03 | 4.46E-04 | 0 | 11 | MaleAllSpecies |
| GO:0070842 | aggresome assembly | 3.76E-03 | 4.46E-04 | 0 | 11 | MaleAllSpecies |
| GO:0035176 | social behavior | 3.76E-03 | 4.46E-04 | 0 | 11 | MaleAllSpecies |
| GO:0035020 | regulation of Rac protein signal transduction | 3.76E-03 | 4.46E-04 | 0 | 11 | MaleAllSpecies |
| GO:0018094 | protein polyglycylation | 3.76E-03 | 4.46E-04 | 0 | 11 | MaleAllSpecies |
| GO:0050918 | positive chemotaxis | 3.76E-03 | 4.46E-04 | 0 | 11 | MaleAllSpecies |
| GO:0085032 | modulation by symbiont of host I-kappaB kinase/NF-kappaB cascade | 3.76E-03 | 4.46E-04 | 0 | 11 | MaleAllSpecies |
| GO:0050690 | regulation of defense response to virus by virus | 3.76E-03 | 4.46E-04 | 0 | 11 | MaleAllSpecies |
| GO:0035019 | somatic stem cell maintenance | 3.78E-03 | 4.49E-04 | 7 | 27 | MaleAllSpecies |
| GO:0035249 | synaptic transmission, glutamatergic | 3.92E-03 | 4.70E-04 | 1 | 14 | MaleAllSpecies |
| GO:0032402 | melanosome transport | 3.92E-03 | 4.70E-04 | 1 | 14 | MaleAllSpecies |
| GO:0045727 | positive regulation of translation | 3.92E-03 | 4.70E-04 | 1 | 14 | MaleAllSpecies |
| GO:0031623 | receptor internalization | 3.96E-03 | 4.76E-04 | 4 | 21 | MaleAllSpecies |
| GO:0050852 | T cell receptor signaling pathway | 4.32E-03 | 5.32E-04 | 10 | 32 | MaleAllSpecies |
| GO:0043124 | negative regulation of I-kappaB kinase/NF-kappaB cascade | 5.24E-03 | 6.60E-04 | 2 | 16 | MaleAllSpecies |
| GO:0007029 | endoplasmic reticulum organization | 5.24E-03 | 6.60E-04 | 2 | 16 | MaleAllSpecies |
| GO:0071260 | cellular response to mechanical stimulus | 6.14E-03 | 7.86E-04 | 6 | 24 | MaleAllSpecies |
| GO:0014047 | glutamate secretion | 6.35E-03 | 8.20E-04 | 5 | 22 | MaleAllSpecies |
| GO:0061326 | renal tubule development | 6.41E-03 | 8.93E-04 | 1 | 13 | MaleAllSpecies |
| GO:0048703 | embryonic viscerocranium morphogenesis | 6.41E-03 | 8.93E-04 | 1 | 13 | MaleAllSpecies |
| GO:0043536 | positive regulation of blood vessel endothelial cell migration | 6.41E-03 | 8.93E-04 | 1 | 13 | MaleAllSpecies |
| GO:0006853 | carnitine shuttle | 6.41E-03 | 9.00E-04 | 0 | 10 | MaleAllSpecies |
| GO:0060296 | regulation of cilium beat frequency involved in ciliary motility | 6.41E-03 | 9.00E-04 | 0 | 10 | MaleAllSpecies |
| GO:0009437 | carnitine metabolic process | 6.41E-03 | 9.00E-04 | 0 | 10 | MaleAllSpecies |
| GO:0002318 | myeloid progenitor cell differentiation | 6.41E-03 | 9.00E-04 | 0 | 10 | MaleAllSpecies |
| GO:0042755 | eating behavior | 6.41E-03 | 9.00E-04 | 0 | 10 | MaleAllSpecies |
| GO:0009225 | nucleotide-sugar metabolic process | 6.41E-03 | 9.00E-04 | 0 | 10 | MaleAllSpecies |
| GO:0001709 | cell fate determination | 6.41E-03 | 9.00E-04 | 0 | 10 | MaleAllSpecies |
| GO:0006532 | aspartate biosynthetic process | 6.41E-03 | 9.00E-04 | 0 | 10 | MaleAllSpecies |
| GO:0055013 | cardiac muscle cell development | 6.41E-03 | 9.00E-04 | 0 | 10 | MaleAllSpecies |
| GO:0045071 | negative regulation of viral genome replication | 6.41E-03 | 9.00E-04 | 0 | 10 | MaleAllSpecies |
| GO:2001014 | regulation of skeletal muscle cell differentiation | 6.41E-03 | 9.00E-04 | 0 | 10 | MaleAllSpecies |
| GO:0010762 | regulation of fibroblast migration | 6.41E-03 | 9.00E-04 | 0 | 10 | MaleAllSpecies |
| GO:0090292 | nuclear matrix anchoring at nuclear membrane | 6.41E-03 | 9.00E-04 | 0 | 10 | MaleAllSpecies |
| GO:0090286 | cytoskeletal anchoring at nuclear membrane | 6.41E-03 | 9.00E-04 | 0 | 10 | MaleAllSpecies |
| GO:0048752 | semicircular canal morphogenesis | 6.41E-03 | 9.00E-04 | 0 | 10 | MaleAllSpecies |
| GO:0046548 | retinal rod cell development | 6.41E-03 | 9.00E-04 | 0 | 10 | MaleAllSpecies |
| GO:0000722 | telomere maintenance via recombination | 6.41E-03 | 9.00E-04 | 0 | 10 | MaleAllSpecies |
| GO:0019551 | glutamate catabolic process to 2-oxoglutarate | 6.41E-03 | 9.00E-04 | 0 | 10 | MaleAllSpecies |
| GO:0019550 | glutamate catabolic process to aspartate | 6.41E-03 | 9.00E-04 | 0 | 10 | MaleAllSpecies |
| GO:0050798 | activated T cell proliferation | 6.41E-03 | 9.00E-04 | 0 | 10 | MaleAllSpecies |
| GO:0002698 | negative regulation of immune effector process | 6.41E-03 | 9.00E-04 | 0 | 10 | MaleAllSpecies |
| GO:0003085 | negative regulation of systemic arterial blood pressure | 6.41E-03 | 9.00E-04 | 0 | 10 | MaleAllSpecies |
| GO:0007286 | spermatid development | 6.66E-03 | 9.48E-04 | 9 | 29 | MaleAllSpecies |
| GO:0018279 | protein N-linked glycosylation via asparagine | 7.29E-03 | 1.07E-03 | 8 | 27 | MaleAllSpecies |
| GO:0042542 | response to hydrogen peroxide | 8.12E-03 | 1.20E-03 | 2 | 15 | MaleAllSpecies |
| GO:2000177 | regulation of neural precursor cell proliferation | 8.12E-03 | 1.20E-03 | 2 | 15 | MaleAllSpecies |
| GO:0019048 | modulation by virus of host morphology or physiology | 9.10E-03 | 1.36E-03 | 33 | 64 | MaleAllSpecies |
| GO:0045766 | positive regulation of angiogenesis | 9.28E-03 | 1.40E-03 | 4 | 19 | MaleAllSpecies |
| GO:0048793 | pronephros development | 9.64E-03 | 1.46E-03 | 12 | 33 | MaleAllSpecies |
| GO:0001964 | startle response | 1.12E-02 | 1.82E-03 | 0 | 9 | MaleAllSpecies |
| GO:0048172 | regulation of short-term neuronal synaptic plasticity | 1.12E-02 | 1.82E-03 | 0 | 9 | MaleAllSpecies |
| GO:0035641 | locomotory exploration behavior | 1.12E-02 | 1.82E-03 | 0 | 9 | MaleAllSpecies |
| GO:0007158 | neuron cell-cell adhesion | 1.12E-02 | 1.82E-03 | 0 | 9 | MaleAllSpecies |
| GO:0043162 | ubiquitin-dependent protein catabolic process via the multivesicular body sorting pathway | 1.12E-02 | 1.82E-03 | 0 | 9 | MaleAllSpecies |
| GO:1901021 | positive regulation of calcium ion transmembrane transporter activity | 1.12E-02 | 1.82E-03 | 0 | 9 | MaleAllSpecies |
| GO:0038108 | negative regulation of appetite by leptin-mediated signaling pathway | 1.12E-02 | 1.82E-03 | 0 | 9 | MaleAllSpecies |

| | | | | | | |
|---|---|---|---|---|---|---|
| GO:0051965 | positive regulation of synapse assembly | 1.12E-02 | 1.82E-03 | 0 | 9 | MaleAllSpecies |
| GO:2001259 | positive regulation of cation channel activity | 1.12E-02 | 1.82E-03 | 0 | 9 | MaleAllSpecies |
| GO:0031060 | regulation of histone methylation | 1.12E-02 | 1.82E-03 | 0 | 9 | MaleAllSpecies |
| GO:0042531 | positive regulation of tyrosine phosphorylation of STAT protein | 1.12E-02 | 1.82E-03 | 0 | 9 | MaleAllSpecies |
| GO:0022904 | respiratory electron transport chain | 1.12E-02 | 1.82E-03 | 0 | 9 | MaleAllSpecies |
| GO:0046856 | phosphatidylinositol dephosphorylation | 1.12E-02 | 1.82E-03 | 0 | 9 | MaleAllSpecies |
| GO:0070846 | Hsp90 deacetylation | 1.12E-02 | 1.82E-03 | 0 | 9 | MaleAllSpecies |
| GO:0071218 | cellular response to misfolded protein | 1.12E-02 | 1.82E-03 | 0 | 9 | MaleAllSpecies |
| GO:2000463 | positive regulation of excitatory postsynaptic membrane potential | 1.12E-02 | 1.82E-03 | 0 | 9 | MaleAllSpecies |
| GO:0090245 | axis elongation involved in somitogenesis | 1.12E-02 | 1.82E-03 | 0 | 9 | MaleAllSpecies |
| GO:0090074 | negative regulation of protein homodimerization activity | 1.12E-02 | 1.82E-03 | 0 | 9 | MaleAllSpecies |
| GO:0090042 | tubulin deacetylation | 1.12E-02 | 1.82E-03 | 0 | 9 | MaleAllSpecies |
| GO:0045724 | positive regulation of cilium assembly | 1.12E-02 | 1.82E-03 | 0 | 9 | MaleAllSpecies |
| GO:0046154 | rhodopsin metabolic process | 1.12E-02 | 1.82E-03 | 0 | 9 | MaleAllSpecies |
| GO:0003016 | respiratory system process | 1.12E-02 | 1.82E-03 | 0 | 9 | MaleAllSpecies |
| GO:0072383 | plus-end-directed vesicle transport along microtubule | 1.12E-02 | 1.82E-03 | 0 | 9 | MaleAllSpecies |
| GO:0048286 | lung alveolus development | 1.12E-02 | 1.82E-03 | 0 | 9 | MaleAllSpecies |
| GO:0031109 | microtubule polymerization or depolymerization | 1.27E-02 | 2.11E-03 | 6 | 22 | MaleAllSpecies |
| GO:0032465 | regulation of cytokinesis | 1.30E-02 | 2.17E-03 | 2 | 14 | MaleAllSpecies |
| GO:0045646 | regulation of erythrocyte differentiation | 1.41E-02 | 2.39E-03 | 4 | 18 | MaleAllSpecies |
| GO:0007411 | axon guidance | 1.70E-02 | 2.95E-03 | 44 | 76 | MaleAllSpecies |
| GO:0007159 | leukocyte cell-cell adhesion | 1.81E-02 | 3.18E-03 | 1 | 11 | MaleAllSpecies |
| GO:0051568 | histone H3-K4 methylation | 1.81E-02 | 3.18E-03 | 1 | 11 | MaleAllSpecies |
| GO:0000724 | double-strand break repair via homologous recombination | 1.81E-02 | 3.18E-03 | 1 | 11 | MaleAllSpecies |
| GO:0060314 | regulation of ryanodine-sensitive calcium-release channel activity | 1.81E-02 | 3.18E-03 | 1 | 11 | MaleAllSpecies |
| GO:0022029 | telencephalon cell migration | 2.02E-02 | 3.66E-03 | 0 | 8 | MaleAllSpecies |
| GO:0036159 | inner dynein arm assembly | 2.02E-02 | 3.66E-03 | 0 | 8 | MaleAllSpecies |
| GO:0036158 | outer dynein arm assembly | 2.02E-02 | 3.66E-03 | 0 | 8 | MaleAllSpecies |
| GO:0015871 | choline transport | 2.02E-02 | 3.66E-03 | 0 | 8 | MaleAllSpecies |
| GO:0086064 | cell communication by electrical coupling involved in cardiac conduction | 2.02E-02 | 3.66E-03 | 0 | 8 | MaleAllSpecies |
| GO:0002042 | cell migration involved in sprouting angiogenesis | 2.02E-02 | 3.66E-03 | 0 | 8 | MaleAllSpecies |
| GO:0035235 | ionotropic glutamate receptor signaling pathway | 2.02E-02 | 3.66E-03 | 0 | 8 | MaleAllSpecies |
| GO:0006270 | DNA replication initiation | 2.02E-02 | 3.66E-03 | 0 | 8 | MaleAllSpecies |
| GO:0044458 | motile cilium assembly | 2.02E-02 | 3.66E-03 | 0 | 8 | MaleAllSpecies |
| GO:1902202 | regulation of hepatocyte growth factor receptor signaling pathway | 2.02E-02 | 3.66E-03 | 0 | 8 | MaleAllSpecies |
| GO:0097113 | alpha-amino-3-hydroxy-5-methyl-4-isoxazole propionate receptor clustering | 2.02E-02 | 3.66E-03 | 0 | 8 | MaleAllSpecies |
| GO:0050777 | negative regulation of immune response | 2.02E-02 | 3.66E-03 | 0 | 8 | MaleAllSpecies |
| GO:0002679 | respiratory burst involved in defense response | 2.02E-02 | 3.66E-03 | 0 | 8 | MaleAllSpecies |
| GO:0031585 | regulation of inositol 1,4,5-trisphosphate-sensitive calcium-release channel activity | 2.02E-02 | 3.66E-03 | 0 | 8 | MaleAllSpecies |
| GO:0043537 | negative regulation of blood vessel endothelial cell migration | 2.02E-02 | 3.66E-03 | 0 | 8 | MaleAllSpecies |
| GO:0021522 | spinal cord motor neuron differentiation | 2.07E-02 | 3.89E-03 | 2 | 13 | MaleAllSpecies |
| GO:0045930 | negative regulation of mitotic cell cycle | 2.07E-02 | 3.89E-03 | 2 | 13 | MaleAllSpecies |
| GO:0007565 | female pregnancy | 2.07E-02 | 3.89E-03 | 2 | 13 | MaleAllSpecies |
| GO:0002366 | leukocyte activation involved in immune response | 2.17E-02 | 4.11E-03 | 3 | 15 | MaleAllSpecies |
| GO:0072073 | kidney epithelium development | 2.17E-02 | 4.11E-03 | 3 | 15 | MaleAllSpecies |
| GO:0032855 | positive regulation of Rac GTPase activity | 2.17E-02 | 4.11E-03 | 3 | 15 | MaleAllSpecies |
| GO:0043281 | regulation of cysteine-type endopeptidase activity involved in apoptotic process | 2.17E-02 | 4.12E-03 | 13 | 32 | MaleAllSpecies |
| GO:0006813 | potassium ion transport | 2.52E-02 | 4.82E-03 | 7 | 22 | MaleAllSpecies |
| GO:0048545 | response to steroid hormone stimulus | 2.52E-02 | 4.84E-03 | 28 | 53 | MaleAllSpecies |
| GO:0006107 | oxaloacetate metabolic process | 3.05E-02 | 5.96E-03 | 1 | 10 | MaleAllSpecies |
| GO:0030148 | sphingolipid biosynthetic process | 3.05E-02 | 5.96E-03 | 1 | 10 | MaleAllSpecies |
| GO:0050685 | positive regulation of mRNA processing | 3.05E-02 | 5.96E-03 | 1 | 10 | MaleAllSpecies |
| GO:0045639 | positive regulation of myeloid cell differentiation | 3.14E-02 | 6.15E-03 | 5 | 18 | MaleAllSpecies |
| GO:0034142 | toll-like receptor 4 signaling pathway | 3.29E-02 | 6.48E-03 | 8 | 23 | MaleAllSpecies |
| GO:0042491 | auditory receptor cell differentiation | 3.47E-02 | 6.93E-03 | 2 | 12 | MaleAllSpecies |
| GO:0010863 | positive regulation of phospholipase C activity | 3.47E-02 | 6.93E-03 | 2 | 12 | MaleAllSpecies |
| GO:0050775 | positive regulation of dendrite morphogenesis | 3.47E-02 | 6.93E-03 | 2 | 12 | MaleAllSpecies |
| GO:0097194 | execution phase of apoptosis | 3.47E-02 | 7.05E-03 | 10 | 26 | MaleAllSpecies |
| GO:0002062 | chondrocyte differentiation | 3.47E-02 | 7.06E-03 | 3 | 14 | MaleAllSpecies |
| GO:0006323 | DNA packaging | 3.47E-02 | 7.06E-03 | 3 | 14 | MaleAllSpecies |
| GO:0050435 | beta-amyloid metabolic process | 3.47E-02 | 7.39E-03 | 0 | 7 | MaleAllSpecies |
| GO:0030817 | regulation of cAMP biosynthetic process | 3.47E-02 | 7.39E-03 | 0 | 7 | MaleAllSpecies |
| GO:0007157 | heterophilic cell-cell adhesion | 3.47E-02 | 7.39E-03 | 0 | 7 | MaleAllSpecies |
| GO:0032964 | collagen biosynthetic process | 3.47E-02 | 7.39E-03 | 0 | 7 | MaleAllSpecies |
| GO:0071625 | vocalization behavior | 3.47E-02 | 7.39E-03 | 0 | 7 | MaleAllSpecies |
| GO:0002260 | lymphocyte homeostasis | 3.47E-02 | 7.39E-03 | 0 | 7 | MaleAllSpecies |
| GO:0000050 | urea cycle | 3.47E-02 | 7.39E-03 | 0 | 7 | MaleAllSpecies |
| GO:0032845 | negative regulation of homeostatic process | 3.47E-02 | 7.39E-03 | 0 | 7 | MaleAllSpecies |
| GO:2000821 | regulation of grooming behavior | 3.47E-02 | 7.39E-03 | 0 | 7 | MaleAllSpecies |
| GO:0006527 | arginine catabolic process | 3.47E-02 | 7.39E-03 | 0 | 7 | MaleAllSpecies |
| GO:0042527 | negative regulation of tyrosine phosphorylation of Stat6 protein | 3.47E-02 | 7.39E-03 | 0 | 7 | MaleAllSpecies |
| GO:0042524 | negative regulation of tyrosine phosphorylation of Stat5 protein | 3.47E-02 | 7.39E-03 | 0 | 7 | MaleAllSpecies |
| GO:0042518 | negative regulation of tyrosine phosphorylation of Stat3 protein | 3.47E-02 | 7.39E-03 | 0 | 7 | MaleAllSpecies |
| GO:0033119 | negative regulation of RNA splicing | 3.47E-02 | 7.39E-03 | 0 | 7 | MaleAllSpecies |
| GO:0046855 | inositol phosphate dephosphorylation | 3.47E-02 | 7.39E-03 | 0 | 7 | MaleAllSpecies |
| GO:0006376 | mRNA splice site selection | 3.47E-02 | 7.39E-03 | 0 | 7 | MaleAllSpecies |
| GO:0010804 | negative regulation of tumor necrosis factor-mediated signaling pathway | 3.47E-02 | 7.39E-03 | 0 | 7 | MaleAllSpecies |
| GO:0090400 | stress-induced premature senescence | 3.47E-02 | 7.39E-03 | 0 | 7 | MaleAllSpecies |
| GO:2000587 | negative regulation of platelet-derived growth factor receptor-beta signaling pathway | 3.47E-02 | 7.39E-03 | 0 | 7 | MaleAllSpecies |
| GO:0034721 | histone H3-K4 demethylation, trimethyl-H3-K4-specific | 3.47E-02 | 7.39E-03 | 0 | 7 | MaleAllSpecies |
| GO:0006282 | regulation of DNA repair | 3.47E-02 | 7.39E-03 | 0 | 7 | MaleAllSpecies |
| GO:0018200 | peptidyl-glutamic acid modification | 3.47E-02 | 7.39E-03 | 0 | 7 | MaleAllSpecies |
| GO:0090329 | regulation of DNA-dependent DNA replication | 3.47E-02 | 7.39E-03 | 0 | 7 | MaleAllSpecies |
| GO:0030206 | chondroitin sulfate biosynthetic process | 3.47E-02 | 7.39E-03 | 0 | 7 | MaleAllSpecies |
| GO:0048742 | regulation of skeletal muscle fiber development | 3.47E-02 | 7.39E-03 | 0 | 7 | MaleAllSpecies |
| GO:2000310 | regulation of N-methyl-D-aspartate selective glutamate receptor activity | 3.47E-02 | 7.39E-03 | 0 | 7 | MaleAllSpecies |
| GO:0051444 | negative regulation of ubiquitin-protein ligase activity | 3.47E-02 | 7.39E-03 | 0 | 7 | MaleAllSpecies |
| GO:1902233 | negative regulation of positive thymic T cell selection | 3.47E-02 | 7.39E-03 | 0 | 7 | MaleAllSpecies |
| GO:0051439 | regulation of ubiquitin-protein ligase activity involved in mitotic cell cycle | 3.47E-02 | 7.39E-03 | 0 | 7 | MaleAllSpecies |
| GO:1902227 | negative regulation of macrophage colony-stimulating factor signaling pathway | 3.47E-02 | 7.39E-03 | 0 | 7 | MaleAllSpecies |
| GO:1902215 | negative regulation of interleukin-4-mediated signaling pathway | 3.47E-02 | 7.39E-03 | 0 | 7 | MaleAllSpecies |
| GO:1902212 | negative regulation of prolactin signaling pathway | 3.47E-02 | 7.39E-03 | 0 | 7 | MaleAllSpecies |
| GO:1902206 | negative regulation of interleukin-2-mediated signaling pathway | 3.47E-02 | 7.39E-03 | 0 | 7 | MaleAllSpecies |
| GO:0003300 | cardiac muscle hypertrophy | 3.47E-02 | 7.39E-03 | 0 | 7 | MaleAllSpecies |
| GO:0072661 | protein targeting to plasma membrane | 3.47E-02 | 7.39E-03 | 0 | 7 | MaleAllSpecies |
| GO:0010324 | membrane invagination | 3.47E-02 | 7.39E-03 | 0 | 7 | MaleAllSpecies |
| GO:0072599 | establishment of protein localization to endoplasmic reticulum | 3.47E-02 | 7.39E-03 | 0 | 7 | MaleAllSpecies |
| GO:0045722 | positive regulation of gluconeogenesis | 3.47E-02 | 7.39E-03 | 0 | 7 | MaleAllSpecies |
| GO:0003009 | skeletal muscle contraction | 3.47E-02 | 7.39E-03 | 0 | 7 | MaleAllSpecies |
| GO:0071910 | determination of liver left/right asymmetry | 3.47E-02 | 7.39E-03 | 0 | 7 | MaleAllSpecies |

| | | | | | | |
|---|---|---|---|---|---|---|
| GO:0045670 | regulation of osteoclast differentiation | 3.47E-02 | 7.39E-03 | 0 | 7 | MaleAllSpecies |
| GO:0045650 | negative regulation of macrophage differentiation | 3.47E-02 | 7.39E-03 | 0 | 7 | MaleAllSpecies |
| GO:0002548 | monocyte chemotaxis | 3.47E-02 | 7.39E-03 | 0 | 7 | MaleAllSpecies |
| GO:0045621 | positive regulation of lymphocyte differentiation | 3.47E-02 | 7.39E-03 | 0 | 7 | MaleAllSpecies |
| GO:0048261 | negative regulation of receptor-mediated endocytosis | 3.47E-02 | 7.39E-03 | 0 | 7 | MaleAllSpecies |
| GO:0060339 | negative regulation of type I interferon-mediated signaling pathway | 3.47E-02 | 7.39E-03 | 0 | 7 | MaleAllSpecies |
| GO:0060336 | negative regulation of interferon-gamma-mediated signaling pathway | 3.47E-02 | 7.39E-03 | 0 | 7 | MaleAllSpecies |
| GO:0070104 | negative regulation of interleukin-6-mediated signaling pathway | 3.47E-02 | 7.39E-03 | 0 | 7 | MaleAllSpecies |
| GO:0014074 | response to purine-containing compound | 3.82E-02 | 8.40E-03 | 9 | 24 | MaleAllSpecies |
| GO:0051260 | protein homooligomerization | 3.93E-02 | 8.66E-03 | 25 | 47 | MaleAllSpecies |
| GO:0042787 | protein ubiquitination involved in ubiquitin-dependent protein catabolic process | 3.96E-02 | 8.74E-03 | 6 | 19 | MaleAllSpecies |
| GO:0035108 | limb morphogenesis | 3.98E-02 | 8.78E-03 | 11 | 27 | MaleAllSpecies |
| GO:0030917 | midbrain-hindbrain boundary development | 4.90E-02 | 1.11E-02 | 1 | 9 | MaleAllSpecies |
| GO:0001818 | negative regulation of cytokine production | 4.90E-02 | 1.11E-02 | 1 | 9 | MaleAllSpecies |
| GO:0048884 | neuromast development | 4.90E-02 | 1.11E-02 | 1 | 9 | MaleAllSpecies |
| GO:0042059 | negative regulation of epidermal growth factor receptor signaling pathway | 4.90E-02 | 1.11E-02 | 1 | 9 | MaleAllSpecies |
| GO:0046835 | carbohydrate phosphorylation | 4.90E-02 | 1.11E-02 | 4 | 15 | MaleAllSpecies |
| GO:0050870 | positive regulation of T cell activation | 4.90E-02 | 1.11E-02 | 4 | 15 | MaleAllSpecies |

**Enriched in Female Tissue of *A. burtoni***

| GO-ID | Term | FDR | P-Value | #FTranscripts | #Mtranscripts | Enriched in |
|---|---|---|---|---|---|---|
| GO:0015991 | ATP hydrolysis coupled proton transport | 5.15E-15 | 7.59E-18 | 61 | 0 | FemaleAburtoni |
| GO:0000046 | autophagic vacuole fusion | 5.15E-15 | 7.59E-18 | 61 | 0 | FemaleAburtoni |
| GO:0001845 | phagolysosome assembly | 1.50E-13 | 3.43E-16 | 61 | 1 | FemaleAburtoni |
| GO:0071466 | cellular response to xenobiotic stimulus | 3.37E-10 | 1.35E-12 | 68 | 7 | FemaleAburtoni |
| GO:0046513 | ceramide biosynthetic process | 4.98E-04 | 1.14E-05 | 18 | 0 | FemaleAburtoni |
| GO:0015813 | L-glutamate transport | 1.37E-03 | 4.23E-05 | 16 | 0 | FemaleAburtoni |
| GO:0042307 | positive regulation of protein import into nucleus | 1.37E-03 | 4.23E-05 | 16 | 0 | FemaleAburtoni |
| GO:0051707 | response to other organism | 1.65E-03 | 5.65E-05 | 37 | 8 | FemaleAburtoni |
| GO:0034470 | ncRNA processing | 1.93E-03 | 6.82E-05 | 25 | 3 | FemaleAburtoni |
| GO:0050434 | positive regulation of viral transcription | 2.22E-03 | 8.16E-05 | 15 | 0 | FemaleAburtoni |
| GO:0051149 | positive regulation of muscle cell differentiation | 2.22E-03 | 8.16E-05 | 15 | 0 | FemaleAburtoni |
| GO:0042254 | ribosome biogenesis | 2.50E-03 | 9.45E-05 | 22 | 2 | FemaleAburtoni |
| GO:2000379 | positive regulation of reactive oxygen species metabolic process | 2.65E-03 | 1.10E-04 | 18 | 1 | FemaleAburtoni |
| GO:0071600 | otic vesicle morphogenesis | 3.55E-03 | 1.58E-04 | 14 | 0 | FemaleAburtoni |
| GO:0010662 | regulation of striated muscle cell apoptotic process | 3.55E-03 | 1.58E-04 | 14 | 0 | FemaleAburtoni |
| GO:0055114 | oxidation-reduction process | 4.23E-03 | 1.92E-04 | 86 | 38 | FemaleAburtoni |
| GO:0043085 | positive regulation of catalytic activity | 4.77E-03 | 2.27E-04 | 91 | 42 | FemaleAburtoni |
| GO:0032926 | negative regulation of activin receptor signaling pathway | 5.76E-03 | 3.05E-04 | 13 | 0 | FemaleAburtoni |
| GO:0090400 | stress-induced premature senescence | 5.76E-03 | 3.05E-04 | 13 | 0 | FemaleAburtoni |
| GO:0042157 | lipoprotein metabolic process | 5.76E-03 | 3.05E-04 | 13 | 0 | FemaleAburtoni |
| GO:0033059 | cellular pigmentation | 6.67E-03 | 3.68E-04 | 16 | 1 | FemaleAburtoni |
| GO:0010212 | response to ionizing radiation | 7.22E-03 | 4.02E-04 | 24 | 4 | FemaleAburtoni |
| GO:0006545 | glycine biosynthetic process | 9.02E-03 | 5.91E-04 | 12 | 0 | FemaleAburtoni |
| GO:0001502 | cartilage condensation | 9.02E-03 | 5.91E-04 | 12 | 0 | FemaleAburtoni |
| GO:0003279 | cardiac septum development | 9.02E-03 | 5.91E-04 | 12 | 0 | FemaleAburtoni |
| GO:0035999 | tetrahydrofolate interconversion | 9.02E-03 | 5.91E-04 | 12 | 0 | FemaleAburtoni |
| GO:0006767 | water-soluble vitamin metabolic process | 9.98E-03 | 6.71E-04 | 15 | 1 | FemaleAburtoni |
| GO:0050931 | pigment cell differentiation | 1.40E-02 | 1.10E-03 | 20 | 3 | FemaleAburtoni |
| GO:0009855 | determination of bilateral symmetry | 1.42E-02 | 1.14E-03 | 24 | 5 | FemaleAburtoni |
| GO:0031101 | fin regeneration | 1.42E-02 | 1.15E-03 | 11 | 0 | FemaleAburtoni |
| GO:0001570 | vasculogenesis | 1.42E-02 | 1.15E-03 | 11 | 0 | FemaleAburtoni |
| GO:0006213 | pyrimidine nucleoside metabolic process | 1.42E-02 | 1.15E-03 | 11 | 0 | FemaleAburtoni |
| GO:2000136 | regulation of cell proliferation involved in heart morphogenesis | 1.42E-02 | 1.15E-03 | 11 | 0 | FemaleAburtoni |
| GO:0030316 | osteoclast differentiation | 1.50E-02 | 1.22E-03 | 14 | 1 | FemaleAburtoni |
| GO:0016072 | rRNA metabolic process | 1.99E-02 | 1.70E-03 | 16 | 2 | FemaleAburtoni |
| GO:0006695 | cholesterol biosynthetic process | 2.18E-02 | 1.90E-03 | 21 | 4 | FemaleAburtoni |
| GO:0001822 | kidney development | 2.28E-02 | 2.01E-03 | 31 | 9 | FemaleAburtoni |
| GO:0023014 | signal transduction by phosphorylation | 2.41E-02 | 2.22E-03 | 13 | 1 | FemaleAburtoni |
| GO:0015936 | coenzyme A metabolic process | 2.41E-02 | 2.23E-03 | 10 | 0 | FemaleAburtoni |
| GO:0070935 | 3'-UTR-mediated mRNA stabilization | 2.41E-02 | 2.23E-03 | 10 | 0 | FemaleAburtoni |
| GO:0016188 | synaptic vesicle maturation | 2.41E-02 | 2.23E-03 | 10 | 0 | FemaleAburtoni |
| GO:0032495 | response to muramyl dipeptide | 2.41E-02 | 2.23E-03 | 10 | 0 | FemaleAburtoni |
| GO:0021984 | adenohypophysis development | 2.41E-02 | 2.23E-03 | 10 | 0 | FemaleAburtoni |
| GO:0045648 | positive regulation of erythrocyte differentiation | 2.41E-02 | 2.23E-03 | 10 | 0 | FemaleAburtoni |
| GO:0032787 | monocarboxylic acid metabolic process | 3.11E-02 | 3.09E-03 | 56 | 25 | FemaleAburtoni |
| GO:0051289 | protein homotetramerization | 3.21E-02 | 3.21E-03 | 18 | 3 | FemaleAburtoni |
| GO:0048010 | vascular endothelial growth factor receptor signaling pathway | 3.94E-02 | 4.02E-03 | 12 | 1 | FemaleAburtoni |
| GO:0050810 | regulation of steroid biosynthetic process | 3.94E-02 | 4.02E-03 | 12 | 1 | FemaleAburtoni |
| GO:0000041 | transition metal ion transport | 4.05E-02 | 4.35E-03 | 9 | 0 | FemaleAburtoni |
| GO:0030656 | regulation of vitamin metabolic process | 4.05E-02 | 4.35E-03 | 9 | 0 | FemaleAburtoni |
| GO:0008593 | regulation of Notch signaling pathway | 4.05E-02 | 4.35E-03 | 9 | 0 | FemaleAburtoni |
| GO:0006283 | transcription-coupled nucleotide-excision repair | 4.05E-02 | 4.35E-03 | 9 | 0 | FemaleAburtoni |
| GO:0006012 | galactose metabolic process | 4.05E-02 | 4.35E-03 | 9 | 0 | FemaleAburtoni |
| GO:0019751 | polyol metabolic process | 4.05E-02 | 4.35E-03 | 9 | 0 | FemaleAburtoni |
| GO:0019747 | regulation of isoprenoid metabolic process | 4.05E-02 | 4.35E-03 | 9 | 0 | FemaleAburtoni |
| GO:0048665 | neuron fate specification | 4.05E-02 | 4.35E-03 | 9 | 0 | FemaleAburtoni |
| GO:0003156 | regulation of organ formation | 4.05E-02 | 4.35E-03 | 9 | 0 | FemaleAburtoni |
| GO:0043589 | skin morphogenesis | 4.05E-02 | 4.35E-03 | 9 | 0 | FemaleAburtoni |
| GO:0035909 | aorta morphogenesis | 4.05E-02 | 4.35E-03 | 9 | 0 | FemaleAburtoni |
| GO:0060487 | lung epithelial cell differentiation | 4.05E-02 | 4.35E-03 | 9 | 0 | FemaleAburtoni |
| GO:0008299 | isoprenoid biosynthetic process | 4.46E-02 | 5.19E-03 | 19 | 4 | FemaleAburtoni |
| GO:0048706 | embryonic skeletal system development | 4.62E-02 | 5.44E-03 | 17 | 3 | FemaleAburtoni |
| GO:0061041 | regulation of wound healing | 4.62E-02 | 5.44E-03 | 17 | 3 | FemaleAburtoni |
| GO:0044070 | regulation of anion transport | 4.62E-02 | 5.44E-03 | 17 | 3 | FemaleAburtoni |

**Enriched in Male Tissue of *A. burtoni***

| GO-ID | Term | FDR | P-Value | #FTranscripts | #Mtranscripts | Enriched in |
|---|---|---|---|---|---|---|
| GO:0007156 | homophilic cell adhesion | 4.06E-05 | 6.20E-07 | 2 | 25 | MaleAburtoni |
| GO:0042384 | cilium assembly | 2.23E-04 | 4.44E-06 | 1 | 20 | MaleAburtoni |
| GO:0043966 | histone H3 acetylation | 7.00E-04 | 1.77E-05 | 1 | 18 | MaleAburtoni |
| GO:0006198 | cAMP catabolic process | 1.56E-03 | 5.26E-05 | 0 | 13 | MaleAburtoni |
| GO:0072331 | signal transduction by p53 class mediator | 2.32E-03 | 8.63E-05 | 3 | 20 | MaleAburtoni |
| GO:0007141 | male meiosis I | 2.65E-03 | 1.13E-04 | 0 | 12 | MaleAburtoni |
| GO:0032968 | positive regulation of transcription elongation from RNA polymerase II promoter | 2.65E-03 | 1.13E-04 | 0 | 12 | MaleAburtoni |
| GO:0016446 | somatic hypermutation of immunoglobulin genes | 2.65E-03 | 1.13E-04 | 0 | 12 | MaleAburtoni |
| GO:0043060 | meiotic metaphase I plate congression | 2.65E-03 | 1.13E-04 | 0 | 12 | MaleAburtoni |
| GO:0050885 | neuromuscular process controlling balance | 2.65E-03 | 1.13E-04 | 0 | 12 | MaleAburtoni |
| GO:0051026 | chiasma assembly | 2.65E-03 | 1.13E-04 | 0 | 12 | MaleAburtoni |
| GO:0008344 | adult locomotory behavior | 2.88E-03 | 1.23E-04 | 2 | 18 | MaleAburtoni |

| | | | | | | |
|---|---|---|---|---|---|---|
| GO:0000122 | negative regulation of transcription from RNA polymerase II promoter | 3.00E-03 | 1.29E-04 | 23 | 51 | MaleAburtoni |
| GO:0010842 | retina layer formation | 4.77E-03 | 2.41E-04 | 0 | 11 | MaleAburtoni |
| GO:0006303 | double-strand break repair via nonhomologous end joining | 4.77E-03 | 2.41E-04 | 0 | 11 | MaleAburtoni |
| GO:0035025 | positive regulation of Rho protein signal transduction | 4.77E-03 | 2.41E-04 | 0 | 11 | MaleAburtoni |
| GO:0051491 | positive regulation of filopodium assembly | 4.77E-03 | 2.41E-04 | 0 | 11 | MaleAburtoni |
| GO:0048384 | retinoic acid receptor signaling pathway | 4.77E-03 | 2.41E-04 | 0 | 11 | MaleAburtoni |
| GO:0051092 | positive regulation of NF-kappaB transcription factor activity | 4.77E-03 | 2.41E-04 | 0 | 11 | MaleAburtoni |
| GO:0001893 | maternal placenta development | 5.38E-03 | 2.78E-04 | 1 | 14 | MaleAburtoni |
| GO:0045727 | positive regulation of translation | 5.38E-03 | 2.78E-04 | 1 | 14 | MaleAburtoni |
| GO:0045944 | positive regulation of transcription from RNA polymerase II promoter | 5.52E-03 | 2.87E-04 | 43 | 75 | MaleAburtoni |
| GO:0034097 | response to cytokine stimulus | 6.44E-03 | 3.52E-04 | 10 | 30 | MaleAburtoni |
| GO:0019048 | modulation by virus of host morphology or physiology | 8.30E-03 | 4.86E-04 | 7 | 25 | MaleAburtoni |
| GO:0035774 | positive regulation of insulin secretion involved in cellular response to glucose stimulus | 8.30E-03 | 5.15E-04 | 0 | 10 | MaleAburtoni |
| GO:0000184 | nuclear-transcribed mRNA catabolic process, nonsense-mediated decay | 8.30E-03 | 5.15E-04 | 0 | 10 | MaleAburtoni |
| GO:0060038 | cardiac muscle cell proliferation | 8.30E-03 | 5.15E-04 | 0 | 10 | MaleAburtoni |
| GO:0051965 | positive regulation of synapse assembly | 8.30E-03 | 5.15E-04 | 0 | 10 | MaleAburtoni |
| GO:0032717 | negative regulation of interleukin-8 production | 8.30E-03 | 5.15E-04 | 0 | 10 | MaleAburtoni |
| GO:0032715 | negative regulation of interleukin-6 production | 8.30E-03 | 5.15E-04 | 0 | 10 | MaleAburtoni |
| GO:0006449 | regulation of translational termination | 8.30E-03 | 5.15E-04 | 0 | 10 | MaleAburtoni |
| GO:0035357 | peroxisome proliferator activated receptor signaling pathway | 8.30E-03 | 5.15E-04 | 0 | 10 | MaleAburtoni |
| GO:0090240 | positive regulation of histone H4 acetylation | 8.30E-03 | 5.15E-04 | 0 | 10 | MaleAburtoni |
| GO:0060687 | regulation of branching involved in prostate gland morphogenesis | 8.30E-03 | 5.15E-04 | 0 | 10 | MaleAburtoni |
| GO:0080182 | histone H3-K4 trimethylation | 8.30E-03 | 5.15E-04 | 0 | 10 | MaleAburtoni |
| GO:0060528 | secretory columnal luminar epithelial cell differentiation involved in prostate glandular acinus development | 8.30E-03 | 5.15E-04 | 0 | 10 | MaleAburtoni |
| GO:0031290 | retinal ganglion cell axon guidance | 8.74E-03 | 5.50E-04 | 1 | 13 | MaleAburtoni |
| GO:0001738 | morphogenesis of a polarized epithelium | 8.74E-03 | 5.50E-04 | 1 | 13 | MaleAburtoni |
| GO:0014032 | neural crest cell development | 8.80E-03 | 5.61E-04 | 4 | 19 | MaleAburtoni |
| GO:0071695 | anatomical structure maturation | 1.18E-02 | 8.11E-04 | 2 | 15 | MaleAburtoni |
| GO:0031623 | receptor internalization | 1.18E-02 | 8.11E-04 | 2 | 15 | MaleAburtoni |
| GO:0016567 | protein ubiquitination | 1.27E-02 | 8.82E-04 | 33 | 59 | MaleAburtoni |
| GO:0019233 | sensory perception of pain | 1.27E-02 | 8.82E-04 | 6 | 22 | MaleAburtoni |
| GO:0043401 | steroid hormone mediated signaling pathway | 1.36E-02 | 9.48E-04 | 5 | 20 | MaleAburtoni |
| GO:0007131 | reciprocal meiotic recombination | 1.40E-02 | 1.08E-03 | 1 | 12 | MaleAburtoni |
| GO:0031124 | mRNA 3'-end processing | 1.40E-02 | 1.08E-03 | 1 | 12 | MaleAburtoni |
| GO:0006298 | mismatch repair | 1.40E-02 | 1.08E-03 | 1 | 12 | MaleAburtoni |
| GO:0009636 | response to toxic substance | 1.40E-02 | 1.08E-03 | 1 | 12 | MaleAburtoni |
| GO:0021548 | pons development | 1.40E-02 | 1.10E-03 | 0 | 9 | MaleAburtoni |
| GO:0065004 | protein-DNA complex assembly | 1.40E-02 | 1.10E-03 | 0 | 9 | MaleAburtoni |
| GO:0007060 | male meiosis chromosome segregation | 1.40E-02 | 1.10E-03 | 0 | 9 | MaleAburtoni |
| GO:0001711 | endodermal cell fate commitment | 1.40E-02 | 1.10E-03 | 0 | 9 | MaleAburtoni |
| GO:0006586 | indolalkylamine metabolic process | 1.40E-02 | 1.10E-03 | 0 | 9 | MaleAburtoni |
| GO:0006333 | chromatin assembly or disassembly | 1.40E-02 | 1.10E-03 | 0 | 9 | MaleAburtoni |
| GO:0051571 | positive regulation of histone H3-K4 methylation | 1.40E-02 | 1.10E-03 | 0 | 9 | MaleAburtoni |
| GO:0010613 | positive regulation of cardiac muscle hypertrophy | 1.40E-02 | 1.10E-03 | 0 | 9 | MaleAburtoni |
| GO:0030100 | regulation of endocytosis | 1.40E-02 | 1.10E-03 | 0 | 9 | MaleAburtoni |
| GO:0070562 | regulation of vitamin D receptor signaling pathway | 1.40E-02 | 1.10E-03 | 0 | 9 | MaleAburtoni |
| GO:0045950 | negative regulation of mitotic recombination | 1.40E-02 | 1.10E-03 | 0 | 9 | MaleAburtoni |
| GO:0051257 | spindle midzone assembly involved in meiosis | 1.40E-02 | 1.10E-03 | 0 | 9 | MaleAburtoni |
| GO:0022412 | cellular process involved in reproduction in multicellular organism | 1.47E-02 | 1.20E-03 | 8 | 25 | MaleAburtoni |
| GO:0001964 | startle response | 1.80E-02 | 1.51E-03 | 2 | 14 | MaleAburtoni |
| GO:0032526 | response to retinoic acid | 1.80E-02 | 1.51E-03 | 2 | 14 | MaleAburtoni |
| GO:0000086 | G2/M transition of mitotic cell cycle | 2.18E-02 | 1.90E-03 | 11 | 28 | MaleAburtoni |
| GO:0046777 | protein autophosphorylation | 2.33E-02 | 2.06E-03 | 15 | 34 | MaleAburtoni |
| GO:0007252 | I-kappaB phosphorylation | 2.36E-02 | 2.13E-03 | 1 | 11 | MaleAburtoni |
| GO:0001937 | negative regulation of endothelial cell proliferation | 2.36E-02 | 2.13E-03 | 1 | 11 | MaleAburtoni |
| GO:0042147 | retrograde transport, endosome to Golgi | 2.36E-02 | 2.13E-03 | 1 | 11 | MaleAburtoni |
| GO:0061099 | negative regulation of protein tyrosine kinase activity | 2.36E-02 | 2.13E-03 | 1 | 11 | MaleAburtoni |
| GO:0048002 | antigen processing and presentation of peptide antigen | 2.46E-02 | 2.35E-03 | 0 | 8 | MaleAburtoni |
| GO:0016199 | axon midline choice point recognition | 2.46E-02 | 2.35E-03 | 0 | 8 | MaleAburtoni |
| GO:0035067 | negative regulation of histone acetylation | 2.46E-02 | 2.35E-03 | 0 | 8 | MaleAburtoni |
| GO:0097120 | receptor localization to synapse | 2.46E-02 | 2.35E-03 | 0 | 8 | MaleAburtoni |
| GO:0097090 | presynaptic membrane organization | 2.46E-02 | 2.35E-03 | 0 | 8 | MaleAburtoni |
| GO:0060445 | branching involved in salivary gland morphogenesis | 2.46E-02 | 2.35E-03 | 0 | 8 | MaleAburtoni |
| GO:0051235 | maintenance of location | 2.58E-02 | 2.49E-03 | 6 | 20 | MaleAburtoni |
| GO:0008630 | intrinsic apoptotic signaling pathway in response to DNA damage | 2.84E-02 | 2.79E-03 | 5 | 18 | MaleAburtoni |
| GO:0048477 | oogenesis | 2.84E-02 | 2.79E-03 | 5 | 18 | MaleAburtoni |
| GO:0090002 | establishment of protein localization to plasma membrane | 2.84E-02 | 2.79E-03 | 2 | 13 | MaleAburtoni |
| GO:0045744 | negative regulation of G-protein coupled receptor protein signaling pathway | 2.84E-02 | 2.79E-03 | 2 | 13 | MaleAburtoni |
| GO:0046488 | phosphatidylinositol metabolic process | 3.98E-02 | 4.12E-03 | 6 | 19 | MaleAburtoni |
| GO:0055012 | ventricular cardiac muscle cell differentiation | 3.98E-02 | 4.16E-03 | 1 | 10 | MaleAburtoni |
| GO:0030516 | regulation of axon extension | 3.98E-02 | 4.16E-03 | 1 | 10 | MaleAburtoni |
| GO:0042446 | hormone biosynthetic process | 3.98E-02 | 4.16E-03 | 1 | 10 | MaleAburtoni |
| GO:0010596 | negative regulation of endothelial cell migration | 3.98E-02 | 4.16E-03 | 1 | 10 | MaleAburtoni |
| GO:0007617 | mating behavior | 3.98E-02 | 4.16E-03 | 1 | 10 | MaleAburtoni |
| GO:0048368 | lateral mesoderm development | 3.98E-02 | 4.16E-03 | 1 | 10 | MaleAburtoni |
| GO:0033564 | anterior/posterior axon guidance | 4.36E-02 | 5.02E-03 | 0 | 7 | MaleAburtoni |
| GO:0043278 | response to morphine | 4.36E-02 | 5.02E-03 | 0 | 7 | MaleAburtoni |
| GO:0031293 | membrane protein intracellular domain proteolysis | 4.36E-02 | 5.02E-03 | 0 | 7 | MaleAburtoni |
| GO:0035616 | histone H2B conserved C-terminal lysine deubiquitination | 4.36E-02 | 5.02E-03 | 0 | 7 | MaleAburtoni |
| GO:0043117 | positive regulation of vascular permeability | 4.36E-02 | 5.02E-03 | 0 | 7 | MaleAburtoni |
| GO:0006509 | membrane protein ectodomain proteolysis | 4.36E-02 | 5.02E-03 | 0 | 7 | MaleAburtoni |
| GO:0038096 | Fc-gamma receptor signaling pathway involved in phagocytosis | 4.36E-02 | 5.02E-03 | 0 | 7 | MaleAburtoni |
| GO:0042537 | benzene-containing compound metabolic process | 4.36E-02 | 5.02E-03 | 0 | 7 | MaleAburtoni |
| GO:0045162 | clustering of voltage-gated sodium channels | 4.36E-02 | 5.02E-03 | 0 | 7 | MaleAburtoni |
| GO:0001554 | luteolysis | 4.36E-02 | 5.02E-03 | 0 | 7 | MaleAburtoni |
| GO:0071321 | cellular response to cGMP | 4.36E-02 | 5.02E-03 | 0 | 7 | MaleAburtoni |
| GO:0034728 | nucleosome organization | 4.36E-02 | 5.02E-03 | 0 | 7 | MaleAburtoni |
| GO:0035176 | social behavior | 4.36E-02 | 5.02E-03 | 0 | 7 | MaleAburtoni |
| GO:0035138 | pectoral fin morphogenesis | 4.36E-02 | 5.02E-03 | 0 | 7 | MaleAburtoni |
| GO:2000463 | positive regulation of excitatory postsynaptic membrane potential | 4.36E-02 | 5.02E-03 | 0 | 7 | MaleAburtoni |
| GO:0070509 | calcium ion import | 4.36E-02 | 5.02E-03 | 0 | 7 | MaleAburtoni |
| GO:0007603 | phototransduction, visible light | 4.36E-02 | 5.02E-03 | 0 | 7 | MaleAburtoni |
| GO:0032091 | negative regulation of protein binding | 4.36E-02 | 5.02E-03 | 0 | 7 | MaleAburtoni |
| GO:0034260 | negative regulation of GTPase activity | 4.36E-02 | 5.02E-03 | 0 | 7 | MaleAburtoni |
| GO:0007413 | axonal fasciculation | 4.36E-02 | 5.02E-03 | 0 | 7 | MaleAburtoni |
| GO:0002204 | somatic recombination of immunoglobulin genes involved in immune response | 4.41E-02 | 5.13E-03 | 2 | 12 | MaleAburtoni |
| GO:0033138 | positive regulation of peptidyl-serine phosphorylation | 4.41E-02 | 5.13E-03 | 2 | 12 | MaleAburtoni |
| GO:0051592 | response to calcium ion | 4.57E-02 | 5.35E-03 | 4 | 15 | MaleAburtoni |
| GO:0043524 | negative regulation of neuron apoptotic process | 4.62E-02 | 5.47E-03 | 3 | 14 | MaleAburtoni |
| GO:0007270 | neuron-neuron synaptic transmission | 4.84E-02 | 5.77E-03 | 7 | 20 | MaleAburtoni |

**Enriched in Female Tissue in *E. cyanostictus***

| GO-ID | Term | FDR | P-Value | #FTranscripts | #Mtranscripts | Enriched in |
|---|---|---|---|---|---|---|
| GO:0033540 | fatty acid beta-oxidation using acyl-CoA oxidase | 2.11E-05 | 2.40E-07 | 14 | 0 | FemaleEcyanostictus |
| GO:0038180 | nerve growth factor signaling pathway | 1.20E-04 | 2.13E-06 | 12 | 0 | FemaleEcyanostictus |
| GO:0051898 | negative regulation of protein kinase B signaling cascade | 1.20E-04 | 2.13E-06 | 12 | 0 | FemaleEcyanostictus |
| GO:0002092 | positive regulation of receptor internalization | 1.20E-04 | 2.13E-06 | 12 | 0 | FemaleEcyanostictus |
| GO:0003402 | planar cell polarity pathway involved in axis elongation | 1.20E-04 | 2.13E-06 | 12 | 0 | FemaleEcyanostictus |
| GO:0051291 | protein heterooligomerization | 1.60E-04 | 3.05E-06 | 25 | 9 | FemaleEcyanostictus |
| GO:0036109 | alpha-linolenic acid metabolic process | 2.48E-04 | 6.35E-06 | 11 | 0 | FemaleEcyanostictus |
| GO:0032516 | positive regulation of phosphoprotein phosphatase activity | 6.04E-04 | 1.91E-05 | 12 | 1 | FemaleEcyanostictus |
| GO:0016559 | peroxisome fission | 6.68E-04 | 2.12E-05 | 15 | 3 | FemaleEcyanostictus |
| GO:0006418 | tRNA aminoacylation for protein translation | 3.04E-03 | 1.44E-04 | 10 | 1 | FemaleEcyanostictus |
| GO:0017148 | negative regulation of translation | 3.04E-03 | 1.44E-04 | 10 | 1 | FemaleEcyanostictus |
| GO:0045200 | establishment of neuroblast polarity | 3.40E-03 | 1.67E-04 | 8 | 0 | FemaleEcyanostictus |
| GO:0071850 | mitotic cell cycle arrest | 5.80E-03 | 3.20E-04 | 12 | 3 | FemaleEcyanostictus |
| GO:0072015 | glomerular visceral epithelial cell development | 5.80E-03 | 3.20E-04 | 12 | 3 | FemaleEcyanostictus |
| GO:0006098 | pentose-phosphate shunt | 6.89E-03 | 3.92E-04 | 9 | 1 | FemaleEcyanostictus |
| GO:0000723 | telomere maintenance | 7.49E-03 | 4.31E-04 | 14 | 5 | FemaleEcyanostictus |
| GO:0071577 | zinc ion transmembrane transport | 8.14E-03 | 4.96E-04 | 7 | 0 | FemaleEcyanostictus |
| GO:0010976 | positive regulation of neuron projection development | 8.31E-03 | 5.07E-04 | 17 | 8 | FemaleEcyanostictus |
| GO:0021761 | limbic system development | 8.87E-03 | 5.50E-04 | 19 | 10 | FemaleEcyanostictus |
| GO:0048671 | negative regulation of collateral sprouting | 1.49E-02 | 1.05E-03 | 8 | 1 | FemaleEcyanostictus |
| GO:0043044 | ATP-dependent chromatin remodeling | 1.89E-02 | 1.47E-03 | 6 | 0 | FemaleEcyanostictus |
| GO:0051875 | pigment granule localization | 1.89E-02 | 1.47E-03 | 6 | 0 | FemaleEcyanostictus |
| GO:0070981 | L-asparagine biosynthetic process | 1.89E-02 | 1.47E-03 | 6 | 0 | FemaleEcyanostictus |
| GO:0051764 | actin crosslink formation | 1.89E-02 | 1.47E-03 | 6 | 0 | FemaleEcyanostictus |
| GO:0090336 | positive regulation of brown fat cell differentiation | 1.89E-02 | 1.47E-03 | 6 | 0 | FemaleEcyanostictus |
| GO:0070376 | regulation of ERK5 cascade | 1.89E-02 | 1.47E-03 | 6 | 0 | FemaleEcyanostictus |
| GO:0031427 | response to methotrexate | 1.89E-02 | 1.47E-03 | 6 | 0 | FemaleEcyanostictus |
| GO:0007026 | negative regulation of microtubule depolymerization | 1.91E-02 | 1.51E-03 | 9 | 2 | FemaleEcyanostictus |
| GO:0055072 | iron ion homeostasis | 1.91E-02 | 1.51E-03 | 9 | 2 | FemaleEcyanostictus |
| GO:0043113 | receptor clustering | 1.95E-02 | 1.54E-03 | 13 | 5 | FemaleEcyanostictus |
| GO:0000038 | very long-chain fatty acid metabolic process | 2.43E-02 | 1.96E-03 | 17 | 10 | FemaleEcyanostictus |
| GO:0019682 | glyceraldehyde-3-phosphate metabolic process | 3.17E-02 | 2.80E-03 | 7 | 1 | FemaleEcyanostictus |
| GO:0006839 | mitochondrial transport | 3.29E-02 | 2.92E-03 | 15 | 8 | FemaleEcyanostictus |
| GO:2000189 | positive regulation of cholesterol homeostasis | 3.32E-02 | 2.98E-03 | 12 | 5 | FemaleEcyanostictus |
| GO:0032481 | positive regulation of type I interferon production | 3.96E-02 | 3.70E-03 | 8 | 2 | FemaleEcyanostictus |
| GO:0031330 | negative regulation of cellular catabolic process | 4.07E-02 | 4.22E-03 | 9 | 3 | FemaleEcyanostictus |
| GO:0030336 | negative regulation of cell migration | 4.07E-02 | 4.29E-03 | 15 | 9 | FemaleEcyanostictus |
| GO:0006882 | cellular zinc ion homeostasis | 4.07E-02 | 4.38E-03 | 5 | 0 | FemaleEcyanostictus |
| GO:0001921 | positive regulation of receptor recycling | 4.07E-02 | 4.38E-03 | 5 | 0 | FemaleEcyanostictus |
| GO:0001880 | Mullerian duct regression | 4.07E-02 | 4.38E-03 | 5 | 0 | FemaleEcyanostictus |
| GO:0002268 | follicular dendritic cell differentiation | 4.07E-02 | 4.38E-03 | 5 | 0 | FemaleEcyanostictus |
| GO:0001783 | B cell apoptotic process | 4.07E-02 | 4.38E-03 | 5 | 0 | FemaleEcyanostictus |
| GO:0045218 | zonula adherens maintenance | 4.07E-02 | 4.38E-03 | 5 | 0 | FemaleEcyanostictus |
| GO:0035435 | phosphate ion transmembrane transport | 4.07E-02 | 4.38E-03 | 5 | 0 | FemaleEcyanostictus |
| GO:0016239 | positive regulation of macroautophagy | 4.07E-02 | 4.38E-03 | 5 | 0 | FemaleEcyanostictus |
| GO:0006335 | DNA replication-dependent nucleosome assembly | 4.07E-02 | 4.38E-03 | 5 | 0 | FemaleEcyanostictus |
| GO:0048933 | afferent axon development in posterior lateral line nerve | 4.07E-02 | 4.38E-03 | 5 | 0 | FemaleEcyanostictus |
| GO:0048929 | efferent axon development in posterior lateral line nerve | 4.07E-02 | 4.38E-03 | 5 | 0 | FemaleEcyanostictus |
| GO:0060971 | embryonic heart tube left/right pattern formation | 4.07E-02 | 4.38E-03 | 5 | 0 | FemaleEcyanostictus |
| GO:0090136 | epithelial cell-cell adhesion | 4.07E-02 | 4.38E-03 | 5 | 0 | FemaleEcyanostictus |
| GO:0021998 | neural plate mediolateral regionalization | 4.07E-02 | 4.38E-03 | 5 | 0 | FemaleEcyanostictus |
| GO:0060600 | dichotomous subdivision of an epithelial terminal unit | 4.07E-02 | 4.38E-03 | 5 | 0 | FemaleEcyanostictus |
| GO:0032108 | negative regulation of response to nutrient levels | 4.07E-02 | 4.38E-03 | 5 | 0 | FemaleEcyanostictus |
| GO:0048378 | regulation of lateral mesodermal cell fate specification | 4.07E-02 | 4.38E-03 | 5 | 0 | FemaleEcyanostictus |
| GO:0048352 | paraxial mesoderm structural organization | 4.07E-02 | 4.38E-03 | 5 | 0 | FemaleEcyanostictus |
| GO:0048305 | immunoglobulin secretion | 4.07E-02 | 4.38E-03 | 5 | 0 | FemaleEcyanostictus |
| GO:0019322 | pentose biosynthetic process | 4.07E-02 | 4.38E-03 | 5 | 0 | FemaleEcyanostictus |
| GO:0071896 | protein localization to adherens junction | 4.07E-02 | 4.38E-03 | 5 | 0 | FemaleEcyanostictus |
| GO:0071894 | histone H2B conserved C-terminal lysine ubiquitination | 4.07E-02 | 4.38E-03 | 5 | 0 | FemaleEcyanostictus |
| GO:0006091 | generation of precursor metabolites and energy | 4.79E-02 | 5.22E-03 | 36 | 36 | FemaleEcyanostictus |

**Enriched in Male Tissue in *E. cyanostictus***

| GO-ID | Term | FDR | P-Value | #FTranscripts | #Mtranscripts | Enriched in |
|---|---|---|---|---|---|---|
| GO:0008284 | positive regulation of cell proliferation | 1.04E-06 | 6.18E-09 | 14 | 111 | MaleEcyanostictus |
| GO:0051592 | response to calcium ion | 1.27E-05 | 1.31E-07 | 0 | 39 | MaleEcyanostictus |
| GO:0001570 | vasculogenesis | 3.24E-05 | 4.14E-07 | 0 | 37 | MaleEcyanostictus |
| GO:0048011 | neurotrophin TRK receptor signaling pathway | 3.69E-05 | 4.91E-07 | 2 | 48 | MaleEcyanostictus |
| GO:0032496 | response to lipopolysaccharide | 1.20E-04 | 2.11E-06 | 3 | 49 | MaleEcyanostictus |
| GO:0006633 | fatty acid biosynthetic process | 1.58E-04 | 2.98E-06 | 4 | 53 | MaleEcyanostictus |
| GO:0019083 | viral transcription | 1.69E-04 | 3.39E-06 | 0 | 31 | MaleEcyanostictus |
| GO:0048546 | digestive tract morphogenesis | 2.38E-04 | 5.74E-06 | 0 | 30 | MaleEcyanostictus |
| GO:0006614 | SRP-dependent cotranslational protein targeting to membrane | 3.56E-04 | 1.01E-05 | 0 | 29 | MaleEcyanostictus |
| GO:0006415 | translational termination | 3.56E-04 | 1.01E-05 | 0 | 29 | MaleEcyanostictus |
| GO:0097306 | cellular response to alcohol | 5.66E-04 | 1.73E-05 | 0 | 27 | MaleEcyanostictus |
| GO:0032355 | response to estradiol stimulus | 7.31E-04 | 2.38E-05 | 3 | 43 | MaleEcyanostictus |
| GO:0007173 | epidermal growth factor receptor signaling pathway | 7.75E-04 | 2.58E-05 | 1 | 33 | MaleEcyanostictus |
| GO:0032570 | response to progesterone stimulus | 1.35E-03 | 5.03E-05 | 0 | 25 | MaleEcyanostictus |
| GO:0010564 | regulation of cell cycle process | 1.43E-03 | 5.42E-05 | 5 | 48 | MaleEcyanostictus |
| GO:0006414 | translational elongation | 1.72E-03 | 6.91E-05 | 1 | 30 | MaleEcyanostictus |
| GO:0008585 | female gonad development | 2.14E-03 | 8.88E-05 | 0 | 23 | MaleEcyanostictus |
| GO:0022602 | ovulation cycle process | 2.14E-03 | 9.02E-05 | 0 | 24 | MaleEcyanostictus |
| GO:0018105 | peptidyl-serine phosphorylation | 2.14E-03 | 9.02E-05 | 0 | 24 | MaleEcyanostictus |
| GO:0071383 | cellular response to steroid hormone stimulus | 2.18E-03 | 9.49E-05 | 3 | 38 | MaleEcyanostictus |
| GO:0072593 | reactive oxygen species metabolic process | 2.60E-03 | 1.18E-04 | 1 | 29 | MaleEcyanostictus |
| GO:0045944 | positive regulation of transcription from RNA polymerase II promoter | 2.69E-03 | 1.23E-04 | 37 | 141 | MaleEcyanostictus |
| GO:0006469 | negative regulation of protein kinase activity | 3.00E-03 | 1.39E-04 | 6 | 49 | MaleEcyanostictus |
| GO:0060047 | heart contraction | 3.07E-03 | 1.48E-04 | 7 | 53 | MaleEcyanostictus |
| GO:0007229 | integrin-mediated signaling pathway | 3.61E-03 | 1.80E-04 | 2 | 32 | MaleEcyanostictus |
| GO:0007224 | smoothened signaling pathway | 3.61E-03 | 1.80E-04 | 2 | 32 | MaleEcyanostictus |
| GO:0008544 | epidermis development | 3.61E-03 | 1.81E-04 | 4 | 41 | MaleEcyanostictus |
| GO:0046883 | regulation of hormone secretion | 3.81E-03 | 1.92E-04 | 1 | 27 | MaleEcyanostictus |
| GO:0055002 | striated muscle cell development | 4.18E-03 | 2.14E-04 | 6 | 48 | MaleEcyanostictus |
| GO:0043065 | positive regulation of apoptotic process | 4.26E-03 | 2.19E-04 | 8 | 54 | MaleEcyanostictus |
| GO:0019221 | cytokine-mediated signaling pathway | 4.58E-03 | 2.37E-04 | 3 | 36 | MaleEcyanostictus |
| GO:0051346 | negative regulation of hydrolase activity | 4.58E-03 | 2.37E-04 | 3 | 36 | MaleEcyanostictus |
| GO:2000736 | regulation of stem cell differentiation | 4.79E-03 | 2.52E-04 | 0 | 21 | MaleEcyanostictus |
| GO:0046890 | regulation of lipid biosynthetic process | 4.79E-03 | 2.52E-04 | 0 | 21 | MaleEcyanostictus |

| | | | | | | |
|---|---|---|---|---|---|---|
| GO:0050819 | negative regulation of coagulation | 4.79E-03 | 2.52E-04 | 0 | 21 | MaleEcyanostictus |
| GO:0042493 | response to drug | 5.28E-03 | 2.81E-04 | 11 | 63 | MaleEcyanostictus |
| GO:0010927 | cellular component assembly involved in morphogenesis | 5.69E-03 | 3.06E-04 | 5 | 43 | MaleEcyanostictus |
| GO:0051384 | response to glucocorticoid stimulus | 5.80E-03 | 3.15E-04 | 1 | 26 | MaleEcyanostictus |
| GO:0006936 | muscle contraction | 5.80E-03 | 3.21E-04 | 8 | 53 | MaleEcyanostictus |
| GO:0000281 | mitotic cytokinesis | 7.58E-03 | 4.47E-04 | 0 | 20 | MaleEcyanostictus |
| GO:0010634 | positive regulation of epithelial cell migration | 7.58E-03 | 4.47E-04 | 0 | 20 | MaleEcyanostictus |
| GO:0009615 | response to virus | 7.58E-03 | 4.47E-04 | 0 | 20 | MaleEcyanostictus |
| GO:0001666 | response to hypoxia | 7.98E-03 | 4.79E-04 | 2 | 30 | MaleEcyanostictus |
| GO:0043281 | regulation of cysteine-type endopeptidase activity involved in apoptotic process | 9.38E-03 | 5.85E-04 | 3 | 33 | MaleEcyanostictus |
| GO:0043405 | regulation of MAP kinase activity | 1.12E-02 | 7.18E-04 | 7 | 47 | MaleEcyanostictus |
| GO:0000077 | DNA damage checkpoint | 1.14E-02 | 7.51E-04 | 0 | 18 | MaleEcyanostictus |
| GO:0010799 | regulation of peptidyl-threonine phosphorylation | 1.14E-02 | 7.51E-04 | 0 | 18 | MaleEcyanostictus |
| GO:0050851 | antigen receptor-mediated signaling pathway | 1.14E-02 | 7.51E-04 | 0 | 18 | MaleEcyanostictus |
| GO:0000184 | nuclear-transcribed mRNA catabolic process, nonsense-mediated decay | 1.21E-02 | 8.06E-04 | 2 | 29 | MaleEcyanostictus |
| GO:0045785 | positive regulation of cell adhesion | 1.21E-02 | 8.06E-04 | 2 | 29 | MaleEcyanostictus |
| GO:0032855 | positive regulation of Rac GTPase activity | 1.21E-02 | 8.18E-04 | 0 | 19 | MaleEcyanostictus |
| GO:0048641 | regulation of skeletal muscle tissue development | 1.21E-02 | 8.18E-04 | 0 | 19 | MaleEcyanostictus |
| GO:0045834 | positive regulation of lipid metabolic process | 1.21E-02 | 8.18E-04 | 0 | 19 | MaleEcyanostictus |
| GO:0006970 | response to osmotic stress | 1.21E-02 | 8.18E-04 | 0 | 19 | MaleEcyanostictus |
| GO:0033002 | muscle cell proliferation | 1.27E-02 | 8.71E-04 | 1 | 23 | MaleEcyanostictus |
| GO:0006979 | response to oxidative stress | 1.29E-02 | 8.95E-04 | 10 | 56 | MaleEcyanostictus |
| GO:0008543 | fibroblast growth factor receptor signaling pathway | 1.32E-02 | 9.25E-04 | 3 | 32 | MaleEcyanostictus |
| GO:0070925 | organelle assembly | 1.44E-02 | 1.02E-03 | 8 | 49 | MaleEcyanostictus |
| GO:0030099 | myeloid cell differentiation | 1.50E-02 | 1.07E-03 | 7 | 46 | MaleEcyanostictus |
| GO:0043410 | positive regulation of MAPK cascade | 1.50E-02 | 1.08E-03 | 4 | 36 | MaleEcyanostictus |
| GO:0035264 | multicellular organism growth | 1.50E-02 | 1.08E-03 | 5 | 39 | MaleEcyanostictus |
| GO:0051153 | regulation of striated muscle cell differentiation | 1.73E-02 | 1.27E-03 | 0 | 17 | MaleEcyanostictus |
| GO:0007281 | germ cell development | 1.89E-02 | 1.42E-03 | 1 | 22 | MaleEcyanostictus |
| GO:0007044 | cell-substrate junction assembly | 1.89E-02 | 1.42E-03 | 1 | 22 | MaleEcyanostictus |
| GO:0007605 | sensory perception of sound | 1.89E-02 | 1.46E-03 | 3 | 30 | MaleEcyanostictus |
| GO:0043009 | chordate embryonic development | 2.07E-02 | 1.65E-03 | 42 | 140 | MaleEcyanostictus |
| GO:0007200 | phospholipase C-activating G-protein coupled receptor signaling pathway | 2.63E-02 | 2.23E-03 | 0 | 16 | MaleEcyanostictus |
| GO:0016525 | negative regulation of angiogenesis | 2.63E-02 | 2.23E-03 | 0 | 16 | MaleEcyanostictus |
| GO:0006703 | estrogen biosynthetic process | 2.63E-02 | 2.23E-03 | 0 | 16 | MaleEcyanostictus |
| GO:0036089 | cleavage furrow formation | 2.63E-02 | 2.23E-03 | 0 | 16 | MaleEcyanostictus |
| GO:0031101 | fin regeneration | 2.63E-02 | 2.23E-03 | 0 | 16 | MaleEcyanostictus |
| GO:0016101 | diterpenoid metabolic process | 2.63E-02 | 2.23E-03 | 0 | 16 | MaleEcyanostictus |
| GO:0015669 | gas transport | 2.63E-02 | 2.23E-03 | 0 | 16 | MaleEcyanostictus |
| GO:0060988 | lipid tube assembly | 2.63E-02 | 2.23E-03 | 0 | 16 | MaleEcyanostictus |
| GO:0046620 | regulation of organ growth | 2.63E-02 | 2.23E-03 | 0 | 16 | MaleEcyanostictus |
| GO:0032461 | positive regulation of protein oligomerization | 2.63E-02 | 2.23E-03 | 0 | 16 | MaleEcyanostictus |
| GO:0050729 | positive regulation of inflammatory response | 2.63E-02 | 2.23E-03 | 0 | 16 | MaleEcyanostictus |
| GO:0045685 | regulation of glial cell differentiation | 2.63E-02 | 2.23E-03 | 0 | 16 | MaleEcyanostictus |
| GO:0051028 | mRNA transport | 2.63E-02 | 2.23E-03 | 0 | 16 | MaleEcyanostictus |
| GO:0072332 | intrinsic apoptotic signaling pathway by p53 class mediator | 2.63E-02 | 2.23E-03 | 0 | 16 | MaleEcyanostictus |
| GO:0060325 | face morphogenesis | 2.63E-02 | 2.23E-03 | 0 | 16 | MaleEcyanostictus |
| GO:0044706 | multi-multicellular organism process | 2.63E-02 | 2.24E-03 | 3 | 29 | MaleEcyanostictus |
| GO:0044703 | multi-organism reproductive process | 2.63E-02 | 2.24E-03 | 3 | 29 | MaleEcyanostictus |
| GO:0050673 | epithelial cell proliferation | 2.68E-02 | 2.29E-03 | 7 | 43 | MaleEcyanostictus |
| GO:0060113 | inner ear receptor cell differentiation | 2.73E-02 | 2.37E-03 | 1 | 21 | MaleEcyanostictus |
| GO:0006665 | sphingolipid metabolic process | 2.73E-02 | 2.37E-03 | 1 | 21 | MaleEcyanostictus |
| GO:0022612 | gland morphogenesis | 2.73E-02 | 2.37E-03 | 1 | 21 | MaleEcyanostictus |
| GO:0007584 | response to nutrient | 2.81E-02 | 2.44E-03 | 4 | 33 | MaleEcyanostictus |
| GO:0043066 | negative regulation of apoptotic process | 3.31E-02 | 2.96E-03 | 23 | 88 | MaleEcyanostictus |
| GO:0007411 | axon guidance | 3.33E-02 | 2.99E-03 | 21 | 82 | MaleEcyanostictus |
| GO:0010629 | negative regulation of gene expression | 3.39E-02 | 3.08E-03 | 41 | 134 | MaleEcyanostictus |
| GO:0010638 | positive regulation of organelle organization | 3.70E-02 | 3.38E-03 | 7 | 41 | MaleEcyanostictus |
| GO:0008154 | actin polymerization or depolymerization | 3.81E-02 | 3.51E-03 | 3 | 28 | MaleEcyanostictus |
| GO:0019932 | second-messenger-mediated signaling | 3.94E-02 | 3.66E-03 | 4 | 31 | MaleEcyanostictus |
| GO:0042110 | T cell activation | 3.94E-02 | 3.66E-03 | 4 | 31 | MaleEcyanostictus |
| GO:0032271 | regulation of protein polymerization | 3.94E-02 | 3.66E-03 | 4 | 31 | MaleEcyanostictus |
| GO:0071902 | positive regulation of protein serine/threonine kinase activity | 3.94E-02 | 3.66E-03 | 4 | 31 | MaleEcyanostictus |
| GO:0035601 | protein deacylation | 3.96E-02 | 3.85E-03 | 0 | 14 | MaleEcyanostictus |
| GO:0007159 | leukocyte cell-cell adhesion | 3.96E-02 | 3.85E-03 | 0 | 14 | MaleEcyanostictus |
| GO:0007109 | cytokinesis, completion of separation | 3.96E-02 | 3.85E-03 | 0 | 14 | MaleEcyanostictus |
| GO:0051568 | histone H3-K4 methylation | 3.96E-02 | 3.85E-03 | 0 | 14 | MaleEcyanostictus |
| GO:0044253 | positive regulation of multicellular organismal metabolic process | 3.96E-02 | 3.85E-03 | 0 | 14 | MaleEcyanostictus |
| GO:0090090 | negative regulation of canonical Wnt receptor signaling pathway | 3.96E-02 | 3.85E-03 | 0 | 14 | MaleEcyanostictus |
| GO:0045930 | negative regulation of mitotic cell cycle | 3.96E-02 | 3.85E-03 | 0 | 14 | MaleEcyanostictus |
| GO:0048477 | oogenesis | 3.96E-02 | 3.85E-03 | 0 | 14 | MaleEcyanostictus |
| GO:0046164 | alcohol catabolic process | 3.96E-02 | 3.85E-03 | 0 | 14 | MaleEcyanostictus |
| GO:0030510 | regulation of BMP signaling pathway | 3.96E-02 | 3.88E-03 | 1 | 19 | MaleEcyanostictus |
| GO:0001578 | microtubule bundle formation | 3.96E-02 | 3.88E-03 | 1 | 19 | MaleEcyanostictus |
| GO:0071216 | cellular response to biotic stimulus | 3.96E-02 | 3.88E-03 | 1 | 19 | MaleEcyanostictus |
| GO:0048708 | astrocyte differentiation | 3.96E-02 | 3.88E-03 | 1 | 19 | MaleEcyanostictus |
| GO:1902275 | regulation of chromatin organization | 3.96E-02 | 3.88E-03 | 1 | 19 | MaleEcyanostictus |
| GO:0030073 | insulin secretion | 3.96E-02 | 3.88E-03 | 1 | 19 | MaleEcyanostictus |
| GO:0002685 | regulation of leukocyte migration | 3.96E-02 | 3.88E-03 | 1 | 19 | MaleEcyanostictus |
| GO:0019217 | regulation of fatty acid metabolic process | 4.05E-02 | 4.04E-03 | 0 | 15 | MaleEcyanostictus |
| GO:0007259 | JAK-STAT cascade | 4.05E-02 | 4.04E-03 | 0 | 15 | MaleEcyanostictus |
| GO:0001569 | patterning of blood vessels | 4.05E-02 | 4.04E-03 | 0 | 15 | MaleEcyanostictus |
| GO:0050670 | regulation of lymphocyte proliferation | 4.05E-02 | 4.04E-03 | 0 | 15 | MaleEcyanostictus |
| GO:0010517 | regulation of phospholipase activity | 4.05E-02 | 4.05E-03 | 1 | 20 | MaleEcyanostictus |
| GO:0070371 | ERK1 and ERK2 cascade | 4.05E-02 | 4.05E-03 | 1 | 20 | MaleEcyanostictus |
| GO:0009416 | response to light stimulus | 4.14E-02 | 4.46E-03 | 14 | 62 | MaleEcyanostictus |
| GO:0060541 | respiratory system development | 4.34E-02 | 4.68E-03 | 11 | 53 | MaleEcyanostictus |
| GO:0038095 | Fc-epsilon receptor signaling pathway | 4.49E-02 | 4.87E-03 | 2 | 23 | MaleEcyanostictus |
| GO:0010876 | lipid localization | 4.94E-02 | 5.42E-03 | 3 | 26 | MaleEcyanostictus |
| GO:0044070 | regulation of anion transport | 4.94E-02 | 5.42E-03 | 3 | 26 | MaleEcyanostictus |
| GO:0034101 | erythrocyte homeostasis | 4.94E-02 | 5.42E-03 | 3 | 26 | MaleEcyanostictus |

**Enriched in Female Tissue of J. ornatus**

| GO-ID | Term | FDR | P-Value | #FTranscripts | #Mtranscripts | Enriched in |
|---|---|---|---|---|---|---|
| GO:0006307 | DNA dealkylation involved in DNA repair | 1.81E-05 | 5.59E-07 | 26 | 0 | FemaleJornatus |
| GO:2001045 | negative regulation of integrin-mediated signaling pathway | 1.81E-05 | 5.59E-07 | 26 | 0 | FemaleJornatus |
| GO:0045654 | positive regulation of megakaryocyte differentiation | 3.24E-05 | 1.07E-06 | 25 | 0 | FemaleJornatus |
| GO:0070933 | histone H4 deacetylation | 5.48E-05 | 2.05E-06 | 24 | 0 | FemaleJornatus |
| GO:0061386 | closure of optic fissure | 7.75E-05 | 3.09E-06 | 27 | 1 | FemaleJornatus |
| GO:0048484 | enteric nervous system development | 7.75E-05 | 3.09E-06 | 27 | 1 | FemaleJornatus |

| | | | | | | |
|---|---|---|---|---|---|---|
| GO:0021535 | cell migration in hindbrain | 9.31E-05 | 3.97E-06 | 23 | 0 | FemaleJornatus |
| GO:0045007 | depurination | 9.31E-05 | 3.97E-06 | 23 | 0 | FemaleJornatus |
| GO:1900052 | regulation of retinoic acid biosynthetic process | 9.31E-05 | 3.97E-06 | 23 | 0 | FemaleJornatus |
| GO:0006364 | rRNA processing | 1.50E-04 | 6.91E-06 | 21 | 0 | FemaleJornatus |
| GO:0051491 | positive regulation of filopodium assembly | 1.50E-04 | 6.91E-06 | 21 | 0 | FemaleJornatus |
| GO:0008360 | regulation of cell shape | 3.51E-04 | 1.85E-05 | 61 | 15 | FemaleJornatus |
| GO:0030218 | erythrocyte differentiation | 3.58E-04 | 1.90E-05 | 34 | 4 | FemaleJornatus |
| GO:0001921 | positive regulation of receptor recycling | 6.98E-04 | 4.18E-05 | 18 | 0 | FemaleJornatus |
| GO:0015991 | ATP hydrolysis coupled proton transport | 6.98E-04 | 4.18E-05 | 18 | 0 | FemaleJornatus |
| GO:0045197 | establishment or maintenance of epithelial cell apical/basal polarity | 6.98E-04 | 4.18E-05 | 18 | 0 | FemaleJornatus |
| GO:0032874 | positive regulation of stress-activated MAPK cascade | 8.66E-04 | 5.55E-05 | 25 | 2 | FemaleJornatus |
| GO:0035249 | synaptic transmission, glutamatergic | 8.66E-04 | 5.55E-05 | 25 | 2 | FemaleJornatus |
| GO:0032776 | DNA methylation on cytosine | 1.14E-03 | 7.70E-05 | 17 | 0 | FemaleJornatus |
| GO:0051497 | negative regulation of stress fiber assembly | 1.14E-03 | 7.70E-05 | 17 | 0 | FemaleJornatus |
| GO:0030037 | actin filament reorganization involved in cell cycle | 1.14E-03 | 7.70E-05 | 17 | 0 | FemaleJornatus |
| GO:0050873 | brown fat cell differentiation | 1.14E-03 | 7.70E-05 | 17 | 0 | FemaleJornatus |
| GO:0006637 | acyl-CoA metabolic process | 1.91E-03 | 1.39E-04 | 47 | 11 | FemaleJornatus |
| GO:0051660 | establishment of centrosome localization | 1.94E-03 | 1.43E-04 | 16 | 0 | FemaleJornatus |
| GO:0006749 | glutathione metabolic process | 2.40E-03 | 1.86E-04 | 20 | 1 | FemaleJornatus |
| GO:0019048 | modulation by virus of host morphology or physiology | 3.04E-03 | 2.47E-04 | 41 | 9 | FemaleJornatus |
| GO:0001755 | neural crest cell migration | 3.12E-03 | 2.55E-04 | 38 | 8 | FemaleJornatus |
| GO:0032846 | positive regulation of homeostatic process | 3.24E-03 | 2.66E-04 | 15 | 0 | FemaleJornatus |
| GO:0006516 | glycoprotein catabolic process | 3.24E-03 | 2.66E-04 | 15 | 0 | FemaleJornatus |
| GO:0006635 | fatty acid beta-oxidation | 3.32E-03 | 2.75E-04 | 22 | 2 | FemaleJornatus |
| GO:0050885 | neuromuscular process controlling balance | 3.99E-03 | 3.38E-04 | 19 | 1 | FemaleJornatus |
| GO:0043244 | regulation of protein complex disassembly | 4.56E-03 | 3.94E-04 | 37 | 8 | FemaleJornatus |
| GO:0006754 | ATP biosynthetic process | 5.65E-03 | 5.00E-04 | 14 | 0 | FemaleJornatus |
| GO:0051568 | histone H3-K4 methylation | 6.46E-03 | 6.00E-04 | 17 | 1 | FemaleJornatus |
| GO:0043089 | positive regulation of Cdc42 GTPase activity | 6.57E-03 | 6.16E-04 | 18 | 1 | FemaleJornatus |
| GO:0030837 | negative regulation of actin filament polymerization | 8.35E-03 | 8.04E-04 | 20 | 2 | FemaleJornatus |
| GO:0007603 | phototransduction, visible light | 8.35E-03 | 8.04E-04 | 20 | 2 | FemaleJornatus |
| GO:0019218 | regulation of steroid metabolic process | 9.53E-03 | 9.46E-04 | 13 | 0 | FemaleJornatus |
| GO:0022904 | respiratory electron transport chain | 9.53E-03 | 9.46E-04 | 13 | 0 | FemaleJornatus |
| GO:0001569 | patterning of blood vessels | 9.53E-03 | 9.46E-04 | 13 | 0 | FemaleJornatus |
| GO:0090303 | positive regulation of wound healing | 9.53E-03 | 9.46E-04 | 13 | 0 | FemaleJornatus |
| GO:2000171 | negative regulation of dendrite development | 9.53E-03 | 9.46E-04 | 13 | 0 | FemaleJornatus |
| GO:0048264 | determination of ventral identity | 9.53E-03 | 9.46E-04 | 13 | 0 | FemaleJornatus |
| GO:0016573 | histone acetylation | 1.01E-02 | 1.02E-03 | 23 | 3 | FemaleJornatus |
| GO:0006987 | activation of signaling protein activity involved in unfolded protein response | 1.01E-02 | 1.02E-03 | 23 | 3 | FemaleJornatus |
| GO:0006144 | purine nucleobase metabolic process | 1.02E-02 | 1.04E-03 | 16 | 1 | FemaleJornatus |
| GO:0019674 | NAD metabolic process | 1.24E-02 | 1.38E-03 | 19 | 2 | FemaleJornatus |
| GO:0009313 | oligosaccharide catabolic process | 1.51E-02 | 1.80E-03 | 12 | 0 | FemaleJornatus |
| GO:0006689 | ganglioside catabolic process | 1.51E-02 | 1.80E-03 | 12 | 0 | FemaleJornatus |
| GO:0000045 | autophagic vacuole assembly | 1.51E-02 | 1.80E-03 | 12 | 0 | FemaleJornatus |
| GO:2000737 | negative regulation of stem cell differentiation | 1.51E-02 | 1.80E-03 | 12 | 0 | FemaleJornatus |
| GO:0001522 | pseudouridine synthesis | 1.51E-02 | 1.80E-03 | 12 | 0 | FemaleJornatus |
| GO:0001510 | RNA methylation | 1.51E-02 | 1.80E-03 | 12 | 0 | FemaleJornatus |
| GO:0061443 | endocardial cushion cell differentiation | 1.51E-02 | 1.80E-03 | 12 | 0 | FemaleJornatus |
| GO:0008049 | male courtship behavior | 1.51E-02 | 1.80E-03 | 12 | 0 | FemaleJornatus |
| GO:0043615 | astrocyte cell migration | 1.51E-02 | 1.80E-03 | 12 | 0 | FemaleJornatus |
| GO:0007341 | penetration of zona pellucida | 1.51E-02 | 1.80E-03 | 12 | 0 | FemaleJornatus |
| GO:0090382 | phagosome maturation | 1.51E-02 | 1.80E-03 | 15 | 1 | FemaleJornatus |
| GO:0046348 | amino sugar catabolic process | 1.51E-02 | 1.80E-03 | 15 | 1 | FemaleJornatus |
| GO:0007626 | locomotory behavior | 2.03E-02 | 2.51E-03 | 43 | 13 | FemaleJornatus |
| GO:0060325 | face morphogenesis | 2.13E-02 | 2.66E-03 | 20 | 3 | FemaleJornatus |
| GO:0032313 | regulation of Rab GTPase activity | 2.31E-02 | 3.14E-03 | 14 | 1 | FemaleJornatus |
| GO:0019321 | pentose metabolic process | 2.31E-02 | 3.14E-03 | 14 | 1 | FemaleJornatus |
| GO:0050808 | synapse organization | 2.34E-02 | 3.19E-03 | 51 | 18 | FemaleJornatus |
| GO:0048172 | regulation of short-term neuronal synaptic plasticity | 2.43E-02 | 3.45E-03 | 11 | 0 | FemaleJornatus |
| GO:0060117 | auditory receptor cell development | 2.43E-02 | 3.45E-03 | 11 | 0 | FemaleJornatus |
| GO:0042340 | keratan sulfate catabolic process | 2.43E-02 | 3.45E-03 | 11 | 0 | FemaleJornatus |
| GO:0016081 | synaptic vesicle docking involved in exocytosis | 2.43E-02 | 3.45E-03 | 11 | 0 | FemaleJornatus |
| GO:0071230 | cellular response to amino acid stimulus | 2.43E-02 | 3.45E-03 | 11 | 0 | FemaleJornatus |
| GO:0097345 | mitochondrial outer membrane permeabilization | 2.43E-02 | 3.45E-03 | 11 | 0 | FemaleJornatus |
| GO:0030214 | hyaluronan catabolic process | 2.43E-02 | 3.45E-03 | 11 | 0 | FemaleJornatus |
| GO:0006177 | GMP biosynthetic process | 2.43E-02 | 3.45E-03 | 11 | 0 | FemaleJornatus |
| GO:0051560 | mitochondrial calcium ion homeostasis | 2.43E-02 | 3.45E-03 | 11 | 0 | FemaleJornatus |
| GO:0097006 | regulation of plasma lipoprotein particle levels | 2.43E-02 | 3.45E-03 | 11 | 0 | FemaleJornatus |
| GO:0050729 | positive regulation of inflammatory response | 2.43E-02 | 3.45E-03 | 11 | 0 | FemaleJornatus |
| GO:0045662 | negative regulation of myoblast differentiation | 2.43E-02 | 3.45E-03 | 11 | 0 | FemaleJornatus |
| GO:0007411 | axon guidance | 2.76E-02 | 4.01E-03 | 62 | 24 | FemaleJornatus |
| GO:0006739 | NADP metabolic process | 2.76E-02 | 4.03E-03 | 17 | 2 | FemaleJornatus |
| GO:0061136 | regulation of proteasomal protein catabolic process | 2.76E-02 | 4.03E-03 | 17 | 2 | FemaleJornatus |
| GO:0043900 | regulation of multi-organism process | 2.76E-02 | 4.04E-03 | 27 | 6 | FemaleJornatus |
| GO:0007405 | neuroblast proliferation | 2.86E-02 | 4.21E-03 | 24 | 5 | FemaleJornatus |
| GO:0010811 | positive regulation of cell-substrate adhesion | 2.94E-02 | 4.33E-03 | 19 | 3 | FemaleJornatus |
| GO:0048145 | regulation of fibroblast proliferation | 3.61E-02 | 5.47E-03 | 13 | 1 | FemaleJornatus |
| GO:0006283 | transcription-coupled nucleotide-excision repair | 3.61E-02 | 5.47E-03 | 13 | 1 | FemaleJornatus |
| GO:0050482 | arachidonic acid secretion | 3.97E-02 | 6.48E-03 | 9 | 0 | FemaleJornatus |
| GO:0052646 | alditol phosphate metabolic process | 3.97E-02 | 6.48E-03 | 9 | 0 | FemaleJornatus |
| GO:0006783 | heme biosynthetic process | 3.97E-02 | 6.48E-03 | 9 | 0 | FemaleJornatus |
| GO:0042761 | very long-chain fatty acid biosynthetic process | 3.97E-02 | 6.48E-03 | 9 | 0 | FemaleJornatus |
| GO:0033336 | caudal fin development | 3.97E-02 | 6.48E-03 | 9 | 0 | FemaleJornatus |
| GO:0031101 | fin regeneration | 3.97E-02 | 6.48E-03 | 9 | 0 | FemaleJornatus |
| GO:0051890 | regulation of cardioblast differentiation | 3.97E-02 | 6.48E-03 | 9 | 0 | FemaleJornatus |
| GO:0046889 | positive regulation of lipid biosynthetic process | 3.97E-02 | 6.48E-03 | 9 | 0 | FemaleJornatus |
| GO:0071371 | cellular response to gonadotropin stimulus | 3.97E-02 | 6.48E-03 | 9 | 0 | FemaleJornatus |
| GO:0010823 | negative regulation of mitochondrion organization | 3.97E-02 | 6.48E-03 | 9 | 0 | FemaleJornatus |
| GO:0051639 | actin filament network formation | 3.97E-02 | 6.48E-03 | 9 | 0 | FemaleJornatus |
| GO:0046456 | icosanoid biosynthetic process | 3.97E-02 | 6.48E-03 | 9 | 0 | FemaleJornatus |
| GO:0051156 | glucose 6-phosphate metabolic process | 3.97E-02 | 6.48E-03 | 9 | 0 | FemaleJornatus |
| GO:0043542 | endothelial cell migration | 3.97E-02 | 6.48E-03 | 9 | 0 | FemaleJornatus |
| GO:0071901 | negative regulation of protein serine/threonine kinase activity | 3.97E-02 | 6.48E-03 | 9 | 0 | FemaleJornatus |
| GO:0051036 | regulation of endosome size | 3.97E-02 | 6.48E-03 | 9 | 0 | FemaleJornatus |
| GO:0048260 | positive regulation of receptor-mediated endocytosis | 3.97E-02 | 6.48E-03 | 9 | 0 | FemaleJornatus |
| GO:0031331 | positive regulation of cellular catabolic process | 3.97E-02 | 6.56E-03 | 23 | 5 | FemaleJornatus |
| GO:0006636 | unsaturated fatty acid biosynthetic process | 3.97E-02 | 6.64E-03 | 10 | 0 | FemaleJornatus |
| GO:0007095 | mitotic G2 DNA damage checkpoint | 3.97E-02 | 6.64E-03 | 10 | 0 | FemaleJornatus |
| GO:0006400 | tRNA modification | 3.97E-02 | 6.64E-03 | 10 | 0 | FemaleJornatus |
| GO:0030497 | fatty acid elongation | 3.97E-02 | 6.64E-03 | 10 | 0 | FemaleJornatus |

| | | | | | | |
|---|---|---|---|---|---|---|
| GO:0006297 | nucleotide-excision repair, DNA gap filling | 3.97E-02 | 6.64E-03 | 10 | 0 | FemaleJornatus |
| GO:0035022 | positive regulation of Rac protein signal transduction | 3.97E-02 | 6.64E-03 | 10 | 0 | FemaleJornatus |
| GO:0046459 | short-chain fatty acid metabolic process | 3.97E-02 | 6.64E-03 | 10 | 0 | FemaleJornatus |
| GO:0097194 | execution phase of apoptosis | 4.07E-02 | 6.88E-03 | 16 | 2 | FemaleJornatus |
| GO:0019915 | lipid storage | 4.15E-02 | 7.03E-03 | 18 | 3 | FemaleJornatus |
| GO:0044264 | cellular polysaccharide metabolic process | 4.15E-02 | 7.03E-03 | 18 | 3 | FemaleJornatus |
| GO:0030879 | mammary gland development | 4.22E-02 | 7.17E-03 | 21 | 4 | FemaleJornatus |
| GO:0001889 | liver development | 4.25E-02 | 7.23E-03 | 43 | 15 | FemaleJornatus |

**Enriched in Male Tissue of J. ornatus**

| GO-ID | Term | FDR | P-Value | #FTranscripts | #Mtranscripts | Enriched in |
|---|---|---|---|---|---|---|
| GO:2001235 | positive regulation of apoptotic signaling pathway | 1.51E-11 | 3.08E-14 | 0 | 37 | MaleJornatus |
| GO:0019886 | antigen processing and presentation of exogenous peptide antigen via MHC class II | 1.51E-11 | 3.08E-14 | 0 | 37 | MaleJornatus |
| GO:0010259 | multicellular organismal aging | 5.04E-10 | 2.11E-12 | 0 | 32 | MaleJornatus |
| GO:0042771 | intrinsic apoptotic signaling pathway in response to DNA damage by p53 class mediator | 9.86E-10 | 4.92E-12 | 0 | 31 | MaleJornatus |
| GO:0010332 | response to gamma radiation | 5.47E-09 | 3.79E-11 | 2 | 35 | MaleJornatus |
| GO:0048485 | sympathetic nervous system development | 5.70E-09 | 4.07E-11 | 1 | 32 | MaleJornatus |
| GO:0030859 | polarized epithelial cell differentiation | 6.00E-09 | 6.19E-11 | 0 | 28 | MaleJornatus |
| GO:0060197 | cloacal septation | 6.00E-09 | 6.19E-11 | 0 | 28 | MaleJornatus |
| GO:0060157 | urinary bladder development | 6.00E-09 | 6.19E-11 | 0 | 28 | MaleJornatus |
| GO:0048807 | female genitalia morphogenesis | 6.00E-09 | 6.19E-11 | 0 | 28 | MaleJornatus |
| GO:0001302 | replicative cell aging | 6.00E-09 | 6.19E-11 | 0 | 28 | MaleJornatus |
| GO:2000381 | negative regulation of mesoderm development | 6.00E-09 | 6.19E-11 | 0 | 28 | MaleJornatus |
| GO:2000271 | positive regulation of fibroblast apoptotic process | 6.00E-09 | 6.19E-11 | 0 | 28 | MaleJornatus |
| GO:0010481 | epidermal cell division | 6.00E-09 | 6.19E-11 | 0 | 28 | MaleJornatus |
| GO:0043616 | keratinocyte proliferation | 6.00E-09 | 6.19E-11 | 0 | 28 | MaleJornatus |
| GO:0060529 | squamous basal epithelial stem cell differentiation involved in prostate gland acinus development | 6.00E-09 | 6.19E-11 | 0 | 28 | MaleJornatus |
| GO:0060513 | prostatic bud formation | 6.00E-09 | 6.19E-11 | 0 | 28 | MaleJornatus |
| GO:0007499 | ectoderm and mesoderm interaction | 6.00E-09 | 6.19E-11 | 0 | 28 | MaleJornatus |
| GO:0006978 | DNA damage response, signal transduction by p53 class mediator resulting in transcription of p21 class mediator | 6.00E-09 | 6.19E-11 | 0 | 28 | MaleJornatus |
| GO:0045617 | negative regulation of keratinocyte differentiation | 6.00E-09 | 6.19E-11 | 0 | 28 | MaleJornatus |
| GO:0010165 | response to X-ray | 8.27E-08 | 1.05E-09 | 1 | 28 | MaleJornatus |
| GO:0047496 | vesicle transport along microtubule | 1.28E-07 | 1.81E-09 | 0 | 24 | MaleJornatus |
| GO:0036342 | post-anal tail morphogenesis | 1.37E-07 | 1.94E-09 | 2 | 30 | MaleJornatus |
| GO:0006338 | chromatin remodeling | 2.56E-07 | 4.05E-09 | 6 | 38 | MaleJornatus |
| GO:0031069 | hair follicle morphogenesis | 2.63E-07 | 4.23E-09 | 2 | 29 | MaleJornatus |
| GO:0000122 | negative regulation of transcription from RNA polymerase II promoter | 3.24E-07 | 5.29E-09 | 45 | 94 | MaleJornatus |
| GO:0034332 | adherens junction organization | 4.37E-07 | 7.34E-09 | 10 | 45 | MaleJornatus |
| GO:0034644 | cellular response to UV | 5.42E-07 | 9.22E-09 | 2 | 28 | MaleJornatus |
| GO:1901660 | calcium ion export | 5.61E-07 | 9.74E-09 | 0 | 22 | MaleJornatus |
| GO:0031571 | mitotic G1 DNA damage checkpoint | 1.60E-06 | 3.20E-08 | 4 | 31 | MaleJornatus |
| GO:0030183 | B cell differentiation | 2.41E-06 | 5.24E-08 | 0 | 20 | MaleJornatus |
| GO:0001736 | establishment of planar polarity | 3.37E-06 | 7.60E-08 | 3 | 28 | MaleJornatus |
| GO:0001892 | embryonic placenta development | 1.05E-05 | 2.73E-07 | 4 | 28 | MaleJornatus |
| GO:0009954 | proximal/distal pattern formation | 1.05E-05 | 2.73E-07 | 4 | 28 | MaleJornatus |
| GO:0002053 | positive regulation of mesenchymal cell proliferation | 1.56E-05 | 4.49E-07 | 7 | 33 | MaleJornatus |
| GO:0010763 | positive regulation of fibroblast migration | 2.09E-05 | 6.53E-07 | 0 | 17 | MaleJornatus |
| GO:0035050 | embryonic heart tube development | 2.28E-05 | 7.26E-07 | 13 | 42 | MaleJornatus |
| GO:0034329 | cell junction assembly | 3.98E-05 | 1.34E-06 | 23 | 55 | MaleJornatus |
| GO:0033198 | response to ATP | 4.20E-05 | 1.44E-06 | 1 | 19 | MaleJornatus |
| GO:0014829 | vascular smooth muscle contraction | 4.20E-05 | 1.44E-06 | 1 | 19 | MaleJornatus |
| GO:0071436 | sodium ion export | 4.32E-05 | 1.51E-06 | 0 | 16 | MaleJornatus |
| GO:0044557 | relaxation of smooth muscle | 4.32E-05 | 1.51E-06 | 0 | 16 | MaleJornatus |
| GO:0097369 | sodium ion import | 4.32E-05 | 1.51E-06 | 0 | 16 | MaleJornatus |
| GO:0051481 | reduction of cytosolic calcium ion concentration | 4.32E-05 | 1.51E-06 | 0 | 16 | MaleJornatus |
| GO:0045747 | positive regulation of Notch signaling pathway | 4.35E-05 | 1.53E-06 | 8 | 33 | MaleJornatus |
| GO:0045669 | positive regulation of osteoblast differentiation | 4.39E-05 | 1.58E-06 | 9 | 35 | MaleJornatus |
| GO:0002028 | regulation of sodium ion transport | 6.74E-05 | 2.57E-06 | 3 | 23 | MaleJornatus |
| GO:0010172 | embryonic body morphogenesis | 8.68E-05 | 3.50E-06 | 0 | 15 | MaleJornatus |
| GO:0071333 | cellular response to glucose stimulus | 1.50E-04 | 6.96E-06 | 1 | 17 | MaleJornatus |
| GO:0071320 | cellular response to cAMP | 2.61E-04 | 1.32E-05 | 4 | 23 | MaleJornatus |
| GO:0030308 | negative regulation of cell growth | 2.61E-04 | 1.32E-05 | 4 | 23 | MaleJornatus |
| GO:0071313 | cellular response to caffeine | 2.97E-04 | 1.53E-05 | 1 | 16 | MaleJornatus |
| GO:0070509 | calcium ion import | 3.92E-04 | 2.11E-05 | 3 | 20 | MaleJornatus |
| GO:0006959 | humoral immune response | 3.92E-04 | 2.11E-05 | 3 | 20 | MaleJornatus |
| GO:0007613 | memory | 4.46E-04 | 2.50E-05 | 4 | 22 | MaleJornatus |
| GO:0048745 | smooth muscle tissue development | 4.70E-04 | 2.66E-05 | 9 | 30 | MaleJornatus |
| GO:0006334 | nucleosome assembly | 5.72E-04 | 3.34E-05 | 1 | 15 | MaleJornatus |
| GO:0035721 | intraflagellar retrograde transport | 7.00E-04 | 4.35E-05 | 0 | 12 | MaleJornatus |
| GO:0072160 | nephron tubule epithelial cell differentiation | 7.00E-04 | 4.35E-05 | 0 | 12 | MaleJornatus |
| GO:0072102 | glomerulus morphogenesis | 7.00E-04 | 4.35E-05 | 0 | 12 | MaleJornatus |
| GO:0060831 | smoothened signaling pathway involved in dorsal/ventral neural tube patterning | 7.00E-04 | 4.35E-05 | 0 | 12 | MaleJornatus |
| GO:0060830 | ciliary receptor clustering involved in smoothened signaling pathway | 7.00E-04 | 4.35E-05 | 0 | 12 | MaleJornatus |
| GO:0045879 | negative regulation of smoothened signaling pathway | 7.00E-04 | 4.35E-05 | 0 | 12 | MaleJornatus |
| GO:0039021 | pronephric glomerulus development | 7.00E-04 | 4.35E-05 | 0 | 12 | MaleJornatus |
| GO:0003009 | skeletal muscle contraction | 7.00E-04 | 4.35E-05 | 0 | 12 | MaleJornatus |
| GO:0007050 | cell cycle arrest | 8.57E-04 | 5.45E-05 | 6 | 24 | MaleJornatus |
| GO:0007283 | spermatogenesis | 1.14E-03 | 7.58E-05 | 27 | 52 | MaleJornatus |
| GO:0045176 | apical protein localization | 1.44E-03 | 1.01E-04 | 0 | 11 | MaleJornatus |
| GO:0048388 | endosomal lumen acidification | 1.44E-03 | 1.01E-04 | 0 | 11 | MaleJornatus |
| GO:0042733 | embryonic digit morphogenesis | 1.62E-03 | 1.17E-04 | 2 | 16 | MaleJornatus |
| GO:0010881 | regulation of cardiac muscle contraction by regulation of the release of sequestered calcium ion | 1.62E-03 | 1.17E-04 | 2 | 16 | MaleJornatus |
| GO:0039022 | pronephric duct development | 1.62E-03 | 1.17E-04 | 2 | 16 | MaleJornatus |
| GO:0051289 | protein homotetramerization | 2.06E-03 | 1.52E-04 | 15 | 35 | MaleJornatus |
| GO:0010842 | retina layer formation | 2.91E-03 | 2.31E-04 | 2 | 15 | MaleJornatus |
| GO:2001242 | regulation of intrinsic apoptotic signaling pathway | 2.91E-03 | 2.33E-04 | 0 | 10 | MaleJornatus |
| GO:0060832 | oocyte animal/vegetal axis specification | 2.91E-03 | 2.33E-04 | 0 | 10 | MaleJornatus |
| GO:0048505 | regulation of timing of cell differentiation | 2.91E-03 | 2.33E-04 | 0 | 10 | MaleJornatus |
| GO:0046327 | glycerol biosynthetic process from pyruvate | 2.91E-03 | 2.33E-04 | 0 | 10 | MaleJornatus |
| GO:0050852 | T cell receptor signaling pathway | 3.90E-03 | 3.28E-04 | 3 | 16 | MaleJornatus |
| GO:0001974 | blood vessel remodeling | 3.99E-03 | 3.40E-04 | 1 | 12 | MaleJornatus |
| GO:0030049 | muscle filament sliding | 3.99E-03 | 3.40E-04 | 1 | 12 | MaleJornatus |
| GO:0048311 | mitochondrion distribution | 5.23E-03 | 4.57E-04 | 2 | 14 | MaleJornatus |
| GO:0044070 | regulation of anion transport | 5.65E-03 | 5.01E-04 | 7 | 22 | MaleJornatus |
| GO:0007274 | neuromuscular synaptic transmission | 5.87E-03 | 5.38E-04 | 0 | 9 | MaleJornatus |
| GO:0035690 | cellular response to drug | 5.87E-03 | 5.38E-04 | 0 | 9 | MaleJornatus |
| GO:0035666 | TRIF-dependent toll-like receptor signaling pathway | 5.87E-03 | 5.38E-04 | 0 | 9 | MaleJornatus |
| GO:0006607 | NLS-bearing substrate import into nucleus | 5.87E-03 | 5.38E-04 | 0 | 9 | MaleJornatus |
| GO:0045329 | carnitine biosynthetic process | 5.87E-03 | 5.38E-04 | 0 | 9 | MaleJornatus |
| GO:0038124 | toll-like receptor TLR6:TLR2 signaling pathway | 5.87E-03 | 5.38E-04 | 0 | 9 | MaleJornatus |

| | | | | | | |
|---|---|---|---|---|---|---|
| GO:0038123 | toll-like receptor TLR1:TLR2 signaling pathway | 5.87E-03 | 5.38E-04 | 0 | 9 | MaleJornatus |
| GO:0008594 | photoreceptor cell morphogenesis | 5.87E-03 | 5.38E-04 | 0 | 9 | MaleJornatus |
| GO:0018095 | protein polyglutamylation | 5.87E-03 | 5.38E-04 | 0 | 9 | MaleJornatus |
| GO:0006003 | fructose 2,6-bisphosphate metabolic process | 5.87E-03 | 5.38E-04 | 0 | 9 | MaleJornatus |
| GO:0050771 | negative regulation of axonogenesis | 5.87E-03 | 5.38E-04 | 0 | 9 | MaleJornatus |
| GO:0034166 | toll-like receptor 10 signaling pathway | 5.87E-03 | 5.38E-04 | 0 | 9 | MaleJornatus |
| GO:0034162 | toll-like receptor 9 signaling pathway | 5.87E-03 | 5.38E-04 | 0 | 9 | MaleJornatus |
| GO:0034138 | toll-like receptor 3 signaling pathway | 5.87E-03 | 5.38E-04 | 0 | 9 | MaleJornatus |
| GO:0060219 | camera-type eye photoreceptor cell differentiation | 6.32E-03 | 5.82E-04 | 5 | 19 | MaleJornatus |
| GO:0035335 | peptidyl-tyrosine dephosphorylation | 6.43E-03 | 5.93E-04 | 4 | 17 | MaleJornatus |
| GO:0017144 | drug metabolic process | 7.63E-03 | 7.28E-04 | 1 | 11 | MaleJornatus |
| GO:0002062 | chondrocyte differentiation | 9.04E-03 | 8.76E-04 | 13 | 29 | MaleJornatus |
| GO:0006857 | oligopeptide transport | 9.23E-03 | 9.02E-04 | 2 | 13 | MaleJornatus |
| GO:0000381 | regulation of alternative mRNA splicing, via spliceosome | 1.08E-02 | 1.10E-03 | 4 | 16 | MaleJornatus |
| GO:0002467 | germinal center formation | 1.12E-02 | 1.24E-03 | 0 | 8 | MaleJornatus |
| GO:0002446 | neutrophil mediated immunity | 1.12E-02 | 1.24E-03 | 0 | 8 | MaleJornatus |
| GO:0007221 | positive regulation of transcription of Notch receptor target | 1.12E-02 | 1.24E-03 | 0 | 8 | MaleJornatus |
| GO:0072149 | glomerular visceral epithelial cell fate commitment | 1.12E-02 | 1.24E-03 | 0 | 8 | MaleJornatus |
| GO:1901189 | positive regulation of ephrin receptor signaling pathway | 1.12E-02 | 1.24E-03 | 0 | 8 | MaleJornatus |
| GO:0048026 | positive regulation of mRNA splicing, via spliceosome | 1.12E-02 | 1.24E-03 | 0 | 8 | MaleJornatus |
| GO:0071498 | cellular response to fluid shear stress | 1.12E-02 | 1.24E-03 | 0 | 8 | MaleJornatus |
| GO:0010907 | positive regulation of glucose metabolic process | 1.12E-02 | 1.24E-03 | 0 | 8 | MaleJornatus |
| GO:0045022 | early endosome to late endosome transport | 1.12E-02 | 1.24E-03 | 0 | 8 | MaleJornatus |
| GO:2001013 | epithelial cell proliferation involved in renal tubule morphogenesis | 1.12E-02 | 1.24E-03 | 0 | 8 | MaleJornatus |
| GO:0030279 | negative regulation of ossification | 1.12E-02 | 1.24E-03 | 0 | 8 | MaleJornatus |
| GO:0060844 | arterial endothelial cell fate commitment | 1.12E-02 | 1.24E-03 | 0 | 8 | MaleJornatus |
| GO:1901844 | regulation of cell communication by electrical coupling involved in cardiac conduction | 1.12E-02 | 1.24E-03 | 0 | 8 | MaleJornatus |
| GO:0008292 | acetylcholine biosynthetic process | 1.12E-02 | 1.24E-03 | 0 | 8 | MaleJornatus |
| GO:0009912 | auditory receptor cell fate commitment | 1.12E-02 | 1.24E-03 | 0 | 8 | MaleJornatus |
| GO:0097101 | blood vessel endothelial cell fate specification | 1.12E-02 | 1.24E-03 | 0 | 8 | MaleJornatus |
| GO:2000212 | negative regulation of glutamate metabolic process | 1.12E-02 | 1.24E-03 | 0 | 8 | MaleJornatus |
| GO:0072602 | interleukin-4 secretion | 1.12E-02 | 1.24E-03 | 0 | 8 | MaleJornatus |
| GO:0003256 | regulation of transcription from RNA polymerase II promoter involved in myocardial precursor cell differentiation | 1.12E-02 | 1.24E-03 | 0 | 8 | MaleJornatus |
| GO:0061028 | establishment of endothelial barrier | 1.12E-02 | 1.24E-03 | 0 | 8 | MaleJornatus |
| GO:0072554 | blood vessel lumenization | 1.12E-02 | 1.24E-03 | 0 | 8 | MaleJornatus |
| GO:0070375 | ERK5 cascade | 1.12E-02 | 1.24E-03 | 0 | 8 | MaleJornatus |
| GO:0035912 | dorsal aorta morphogenesis | 1.12E-02 | 1.24E-03 | 0 | 8 | MaleJornatus |
| GO:0035845 | photoreceptor cell outer segment organization | 1.12E-02 | 1.24E-03 | 0 | 8 | MaleJornatus |
| GO:0010469 | regulation of receptor activity | 1.12E-02 | 1.25E-03 | 11 | 26 | MaleJornatus |
| GO:0007612 | learning | 1.12E-02 | 1.25E-03 | 11 | 26 | MaleJornatus |
| GO:0043524 | negative regulation of neuron apoptotic process | 1.14E-02 | 1.27E-03 | 8 | 22 | MaleJornatus |
| GO:0014823 | response to activity | 1.33E-02 | 1.50E-03 | 7 | 20 | MaleJornatus |
| GO:0007271 | synaptic transmission, cholinergic | 1.34E-02 | 1.55E-03 | 1 | 10 | MaleJornatus |
| GO:0048733 | sebaceous gland development | 1.34E-02 | 1.55E-03 | 1 | 10 | MaleJornatus |
| GO:2000138 | positive regulation of cell proliferation involved in heart morphogenesis | 1.34E-02 | 1.55E-03 | 1 | 10 | MaleJornatus |
| GO:0003198 | epithelial to mesenchymal transition involved in endocardial cushion formation | 1.34E-02 | 1.55E-03 | 1 | 10 | MaleJornatus |
| GO:0033762 | response to glucagon stimulus | 1.34E-02 | 1.55E-03 | 1 | 10 | MaleJornatus |
| GO:0036302 | atrioventricular canal development | 1.34E-02 | 1.55E-03 | 1 | 10 | MaleJornatus |
| GO:0007205 | protein kinase C-activating G-protein coupled receptor signaling pathway | 1.51E-02 | 1.78E-03 | 2 | 12 | MaleJornatus |
| GO:0072178 | nephric duct morphogenesis | 1.51E-02 | 1.78E-03 | 2 | 12 | MaleJornatus |
| GO:0072114 | pronephros morphogenesis | 1.51E-02 | 1.78E-03 | 2 | 12 | MaleJornatus |
| GO:0060271 | cilium morphogenesis | 1.51E-02 | 1.81E-03 | 28 | 45 | MaleJornatus |
| GO:0002026 | regulation of the force of heart contraction | 1.52E-02 | 1.82E-03 | 5 | 17 | MaleJornatus |
| GO:0006821 | chloride transport | 1.76E-02 | 2.14E-03 | 15 | 30 | MaleJornatus |
| GO:0042475 | odontogenesis of dentin-containing tooth | 1.76E-02 | 2.14E-03 | 15 | 30 | MaleJornatus |
| GO:0008285 | negative regulation of cell proliferation | 2.07E-02 | 2.57E-03 | 28 | 44 | MaleJornatus |
| GO:0071872 | cellular response to epinephrine stimulus | 2.15E-02 | 2.87E-03 | 0 | 7 | MaleJornatus |
| GO:0006853 | carnitine shuttle | 2.15E-02 | 2.87E-03 | 0 | 7 | MaleJornatus |
| GO:0033590 | response to cobalamin | 2.15E-02 | 2.87E-03 | 0 | 7 | MaleJornatus |
| GO:0009414 | response to water deprivation | 2.15E-02 | 2.87E-03 | 0 | 7 | MaleJornatus |
| GO:0050432 | catecholamine secretion | 2.15E-02 | 2.87E-03 | 0 | 7 | MaleJornatus |
| GO:0035611 | protein branching point deglutamylation | 2.15E-02 | 2.87E-03 | 0 | 7 | MaleJornatus |
| GO:0007193 | adenylate cyclase-inhibiting G-protein coupled receptor signaling pathway | 2.15E-02 | 2.87E-03 | 0 | 7 | MaleJornatus |
| GO:0033344 | cholesterol efflux | 2.15E-02 | 2.87E-03 | 0 | 7 | MaleJornatus |
| GO:0047484 | regulation of response to osmotic stress | 2.15E-02 | 2.87E-03 | 0 | 7 | MaleJornatus |
| GO:0032754 | positive regulation of interleukin-5 production | 2.15E-02 | 2.87E-03 | 0 | 7 | MaleJornatus |
| GO:0032743 | positive regulation of interleukin-2 production | 2.15E-02 | 2.87E-03 | 0 | 7 | MaleJornatus |
| GO:0071477 | cellular hypotonic salinity response | 2.15E-02 | 2.87E-03 | 0 | 7 | MaleJornatus |
| GO:0071420 | cellular response to histamine | 2.15E-02 | 2.87E-03 | 0 | 7 | MaleJornatus |
| GO:0086024 | adrenergic receptor signaling pathway involved in positive regulation of heart rate | 2.15E-02 | 2.87E-03 | 0 | 7 | MaleJornatus |
| GO:0033137 | negative regulation of peptidyl-serine phosphorylation | 2.15E-02 | 2.87E-03 | 0 | 7 | MaleJornatus |
| GO:0033133 | positive regulation of glucokinase activity | 2.15E-02 | 2.87E-03 | 0 | 7 | MaleJornatus |
| GO:0015721 | bile acid and bile salt transport | 2.15E-02 | 2.87E-03 | 0 | 7 | MaleJornatus |
| GO:0033032 | regulation of myeloid cell apoptotic process | 2.15E-02 | 2.87E-03 | 0 | 7 | MaleJornatus |
| GO:0032413 | negative regulation of ion transmembrane transporter activity | 2.15E-02 | 2.87E-03 | 0 | 7 | MaleJornatus |
| GO:1901898 | negative regulation of relaxation of cardiac muscle | 2.15E-02 | 2.87E-03 | 0 | 7 | MaleJornatus |
| GO:2000378 | negative regulation of reactive oxygen species metabolic process | 2.15E-02 | 2.87E-03 | 0 | 7 | MaleJornatus |
| GO:0006097 | glyoxylate cycle | 2.15E-02 | 2.87E-03 | 0 | 7 | MaleJornatus |
| GO:0032237 | activation of store-operated calcium channel activity | 2.15E-02 | 2.87E-03 | 0 | 7 | MaleJornatus |
| GO:0030002 | cellular anion homeostasis | 2.15E-02 | 2.87E-03 | 0 | 7 | MaleJornatus |
| GO:0045932 | negative regulation of muscle contraction | 2.15E-02 | 2.87E-03 | 0 | 7 | MaleJornatus |
| GO:0097009 | energy homeostasis | 2.15E-02 | 2.87E-03 | 0 | 7 | MaleJornatus |
| GO:0019518 | L-threonine catabolic process to glycine | 2.15E-02 | 2.87E-03 | 0 | 7 | MaleJornatus |
| GO:0060487 | lung epithelial cell differentiation | 2.15E-02 | 2.87E-03 | 0 | 7 | MaleJornatus |
| GO:0021603 | cranial nerve formation | 2.15E-02 | 2.87E-03 | 0 | 7 | MaleJornatus |
| GO:0050795 | regulation of behavior | 2.17E-02 | 2.91E-03 | 6 | 18 | MaleJornatus |
| GO:0021537 | telencephalon development | 2.31E-02 | 3.12E-03 | 24 | 39 | MaleJornatus |
| GO:0071363 | cellular response to growth factor stimulus | 2.33E-02 | 3.18E-03 | 50 | 66 | MaleJornatus |
| GO:0071711 | basement membrane organization | 2.37E-02 | 3.28E-03 | 1 | 9 | MaleJornatus |
| GO:0000002 | mitochondrial genome maintenance | 2.37E-02 | 3.28E-03 | 1 | 9 | MaleJornatus |
| GO:0055078 | sodium ion homeostasis | 2.37E-02 | 3.28E-03 | 1 | 9 | MaleJornatus |
| GO:0050853 | B cell receptor signaling pathway | 2.37E-02 | 3.28E-03 | 1 | 9 | MaleJornatus |
| GO:0048536 | spleen development | 2.37E-02 | 3.28E-03 | 1 | 9 | MaleJornatus |
| GO:0050732 | negative regulation of peptidyl-tyrosine phosphorylation | 2.37E-02 | 3.28E-03 | 1 | 9 | MaleJornatus |
| GO:0034146 | toll-like receptor 5 signaling pathway | 2.37E-02 | 3.28E-03 | 1 | 9 | MaleJornatus |
| GO:0034142 | toll-like receptor 4 signaling pathway | 2.37E-02 | 3.28E-03 | 1 | 9 | MaleJornatus |
| GO:0034134 | toll-like receptor 2 signaling pathway | 2.37E-02 | 3.28E-03 | 1 | 9 | MaleJornatus |
| GO:0051291 | protein heterooligomerization | 2.59E-02 | 3.73E-03 | 3 | 13 | MaleJornatus |
| GO:0045214 | sarcomere organization | 2.75E-02 | 3.97E-03 | 16 | 30 | MaleJornatus |
| GO:0055010 | ventricular cardiac muscle tissue morphogenesis | 3.26E-02 | 4.88E-03 | 6 | 17 | MaleJornatus |

| | | | | | | |
|---|---|---|---|---|---|---|
| GO:0001958 | endochondral ossification | 3.97E-02 | 6.63E-03 | 0 | 6 | MaleJornatus |
| GO:0009225 | nucleotide-sugar metabolic process | 3.97E-02 | 6.63E-03 | 0 | 6 | MaleJornatus |
| GO:0006570 | tyrosine metabolic process | 3.97E-02 | 6.63E-03 | 0 | 6 | MaleJornatus |
| GO:0051952 | regulation of amine transport | 3.97E-02 | 6.63E-03 | 0 | 6 | MaleJornatus |
| GO:0055005 | ventricular cardiac myofibril assembly | 3.97E-02 | 6.63E-03 | 0 | 6 | MaleJornatus |
| GO:0031061 | negative regulation of histone methylation | 3.97E-02 | 6.63E-03 | 0 | 6 | MaleJornatus |
| GO:2000727 | positive regulation of cardiac muscle cell differentiation | 3.97E-02 | 6.63E-03 | 0 | 6 | MaleJornatus |
| GO:0002029 | desensitization of G-protein coupled receptor protein signaling pathway | 3.97E-02 | 6.63E-03 | 0 | 6 | MaleJornatus |
| GO:0071386 | cellular response to corticosterone stimulus | 3.97E-02 | 6.63E-03 | 0 | 6 | MaleJornatus |
| GO:0042416 | dopamine biosynthetic process | 3.97E-02 | 6.63E-03 | 0 | 6 | MaleJornatus |
| GO:0071305 | cellular response to vitamin D | 3.97E-02 | 6.63E-03 | 0 | 6 | MaleJornatus |
| GO:0034656 | nucleobase-containing small molecule catabolic process | 3.97E-02 | 6.63E-03 | 0 | 6 | MaleJornatus |
| GO:0030241 | skeletal muscle myosin thick filament assembly | 3.97E-02 | 6.63E-03 | 0 | 6 | MaleJornatus |
| GO:0051574 | positive regulation of histone H3-K9 methylation | 3.97E-02 | 6.63E-03 | 0 | 6 | MaleJornatus |
| GO:0030206 | chondroitin sulfate biosynthetic process | 3.97E-02 | 6.63E-03 | 0 | 6 | MaleJornatus |
| GO:0061303 | cornea development in camera-type eye | 3.97E-02 | 6.63E-03 | 0 | 6 | MaleJornatus |
| GO:0048739 | cardiac muscle fiber development | 3.97E-02 | 6.63E-03 | 0 | 6 | MaleJornatus |
| GO:0046503 | glycerolipid catabolic process | 3.97E-02 | 6.63E-03 | 0 | 6 | MaleJornatus |
| GO:0010575 | positive regulation vascular endothelial growth factor production | 3.97E-02 | 6.63E-03 | 0 | 6 | MaleJornatus |
| GO:0090162 | establishment of epithelial cell polarity | 3.97E-02 | 6.63E-03 | 0 | 6 | MaleJornatus |
| GO:0003383 | apical constriction | 3.97E-02 | 6.63E-03 | 0 | 6 | MaleJornatus |
| GO:0048640 | negative regulation of developmental growth | 3.97E-02 | 6.63E-03 | 0 | 6 | MaleJornatus |
| GO:0014898 | cardiac muscle hypertrophy in response to stress | 3.97E-02 | 6.63E-03 | 0 | 6 | MaleJornatus |
| GO:0030033 | microvillus assembly | 3.97E-02 | 6.63E-03 | 0 | 6 | MaleJornatus |
| GO:0050830 | defense response to Gram-positive bacterium | 3.97E-02 | 6.63E-03 | 0 | 6 | MaleJornatus |
| GO:0031641 | regulation of myelination | 3.97E-02 | 6.63E-03 | 0 | 6 | MaleJornatus |
| GO:0060425 | lung morphogenesis | 3.97E-02 | 6.63E-03 | 0 | 6 | MaleJornatus |
| GO:0045685 | regulation of glial cell differentiation | 3.97E-02 | 6.63E-03 | 0 | 6 | MaleJornatus |
| GO:0002576 | platelet degranulation | 3.97E-02 | 6.63E-03 | 0 | 6 | MaleJornatus |
| GO:0035019 | somatic stem cell maintenance | 3.99E-02 | 6.70E-03 | 4 | 13 | MaleJornatus |
| GO:1900449 | regulation of glutamate receptor signaling pathway | 4.01E-02 | 6.74E-03 | 2 | 10 | MaleJornatus |
| GO:0031063 | regulation of histone deacetylation | 4.01E-02 | 6.76E-03 | 3 | 12 | MaleJornatus |

**Enriched in Female Tissue of *O. ventralis***

| GO-ID | Term | FDR | P-Value | #FTranscripts | #Mtranscripts | Enriched in |
|---|---|---|---|---|---|---|
| GO:0032237 | activation of store-operated calcium channel activity | 1.30E-06 | 3.57E-09 | 23 | 0 | FemaleOventralis |
| GO:0051835 | positive regulation of synapse structural plasticity | 5.06E-05 | 5.84E-07 | 17 | 0 | FemaleOventralis |
| GO:0046827 | positive regulation of protein export from nucleus | 5.06E-05 | 5.84E-07 | 17 | 0 | FemaleOventralis |
| GO:0060267 | positive regulation of respiratory burst | 9.54E-05 | 1.36E-06 | 16 | 0 | FemaleOventralis |
| GO:0008078 | mesodermal cell migration | 1.09E-04 | 1.74E-06 | 21 | 2 | FemaleOventralis |
| GO:0060999 | positive regulation of dendritic spine development | 2.89E-04 | 6.26E-06 | 17 | 1 | FemaleOventralis |
| GO:0090307 | spindle assembly involved in mitosis | 3.16E-04 | 7.42E-06 | 14 | 0 | FemaleOventralis |
| GO:0050732 | negative regulation of peptidyl-tyrosine phosphorylation | 9.04E-04 | 2.87E-05 | 18 | 2 | FemaleOventralis |
| GO:2001256 | regulation of store-operated calcium entry | 9.48E-04 | 3.05E-05 | 15 | 1 | FemaleOventralis |
| GO:0005513 | detection of calcium ion | 1.16E-03 | 3.84E-05 | 19 | 3 | FemaleOventralis |
| GO:0006562 | proline catabolic process | 1.16E-03 | 4.03E-05 | 12 | 0 | FemaleOventralis |
| GO:0006537 | glutamate biosynthetic process | 1.16E-03 | 4.03E-05 | 12 | 0 | FemaleOventralis |
| GO:0044335 | canonical Wnt receptor signaling pathway involved in neural crest cell differentiation | 1.16E-03 | 4.03E-05 | 12 | 0 | FemaleOventralis |
| GO:0006200 | ATP catabolic process | 1.58E-03 | 6.07E-05 | 39 | 17 | FemaleOventralis |
| GO:0060178 | regulation of exocyst localization | 2.08E-03 | 9.40E-05 | 11 | 0 | FemaleOventralis |
| GO:0032859 | activation of Ral GTPase activity | 2.08E-03 | 9.40E-05 | 11 | 0 | FemaleOventralis |
| GO:0033126 | positive regulation of GTP catabolic process | 2.08E-03 | 9.40E-05 | 11 | 0 | FemaleOventralis |
| GO:0045022 | early endosome to late endosome transport | 2.08E-03 | 9.40E-05 | 11 | 0 | FemaleOventralis |
| GO:1900220 | semaphorin-plexin signaling pathway involved in bone trabecula morphogenesis | 2.08E-03 | 9.40E-05 | 11 | 0 | FemaleOventralis |
| GO:0051298 | centrosome duplication | 2.08E-03 | 9.40E-05 | 11 | 0 | FemaleOventralis |
| GO:0010259 | multicellular organismal aging | 2.08E-03 | 9.40E-05 | 11 | 0 | FemaleOventralis |
| GO:0032793 | positive regulation of CREB transcription factor activity | 2.34E-03 | 1.11E-04 | 16 | 2 | FemaleOventralis |
| GO:0050766 | positive regulation of phagocytosis | 2.34E-03 | 1.11E-04 | 16 | 2 | FemaleOventralis |
| GO:0006734 | NADH metabolic process | 3.85E-03 | 2.19E-04 | 10 | 0 | FemaleOventralis |
| GO:0007062 | sister chromatid cohesion | 3.85E-03 | 2.19E-04 | 10 | 0 | FemaleOventralis |
| GO:0006103 | 2-oxoglutarate metabolic process | 3.85E-03 | 2.19E-04 | 10 | 0 | FemaleOventralis |
| GO:0006102 | isocitrate metabolic process | 3.85E-03 | 2.19E-04 | 10 | 0 | FemaleOventralis |
| GO:0006099 | tricarboxylic acid cycle | 3.85E-03 | 2.19E-04 | 10 | 0 | FemaleOventralis |
| GO:2000104 | negative regulation of DNA-dependent DNA replication | 3.85E-03 | 2.19E-04 | 10 | 0 | FemaleOventralis |
| GO:0008033 | tRNA processing | 3.85E-03 | 2.19E-04 | 10 | 0 | FemaleOventralis |
| GO:0043525 | positive regulation of neuron apoptotic process | 3.85E-03 | 2.19E-04 | 10 | 0 | FemaleOventralis |
| GO:0022011 | myelination in peripheral nervous system | 6.56E-03 | 4.37E-04 | 14 | 2 | FemaleOventralis |
| GO:0090305 | nucleic acid phosphodiester bond hydrolysis | 6.56E-03 | 4.37E-04 | 14 | 2 | FemaleOventralis |
| GO:0035725 | sodium ion transmembrane transport | 6.56E-03 | 4.56E-04 | 39 | 20 | FemaleOventralis |
| GO:0031397 | negative regulation of protein ubiquitination | 6.56E-03 | 5.09E-04 | 9 | 0 | FemaleOventralis |
| GO:0006659 | phosphatidylserine biosynthetic process | 6.56E-03 | 5.09E-04 | 9 | 0 | FemaleOventralis |
| GO:0000075 | cell cycle checkpoint | 6.56E-03 | 5.09E-04 | 9 | 0 | FemaleOventralis |
| GO:0033198 | response to ATP | 6.56E-03 | 5.09E-04 | 9 | 0 | FemaleOventralis |
| GO:0042267 | natural killer cell mediated cytotoxicity | 6.56E-03 | 5.09E-04 | 9 | 0 | FemaleOventralis |
| GO:0010693 | negative regulation of alkaline phosphatase activity | 6.56E-03 | 5.09E-04 | 9 | 0 | FemaleOventralis |
| GO:0046513 | ceramide biosynthetic process | 6.56E-03 | 5.09E-04 | 9 | 0 | FemaleOventralis |
| GO:0002924 | negative regulation of humoral immune response mediated by circulating immunoglobulin | 6.56E-03 | 5.09E-04 | 9 | 0 | FemaleOventralis |
| GO:0048672 | positive regulation of collateral sprouting | 6.56E-03 | 5.09E-04 | 9 | 0 | FemaleOventralis |
| GO:0030071 | regulation of mitotic metaphase/anaphase transition | 6.56E-03 | 5.09E-04 | 9 | 0 | FemaleOventralis |
| GO:0070486 | leukocyte aggregation | 6.56E-03 | 5.09E-04 | 9 | 0 | FemaleOventralis |
| GO:0050860 | negative regulation of T cell receptor signaling pathway | 6.56E-03 | 5.09E-04 | 9 | 0 | FemaleOventralis |
| GO:1902083 | negative regulation of peptidyl-cysteine S-nitrosylation | 6.56E-03 | 5.09E-04 | 9 | 0 | FemaleOventralis |
| GO:0003117 | regulation of vasoconstriction by circulating norepinephrine | 6.56E-03 | 5.09E-04 | 9 | 0 | FemaleOventralis |
| GO:0003056 | regulation of vascular smooth muscle contraction | 6.56E-03 | 5.09E-04 | 9 | 0 | FemaleOventralis |
| GO:0002554 | serotonin secretion by platelet | 6.56E-03 | 5.09E-04 | 9 | 0 | FemaleOventralis |
| GO:0007320 | insemination | 6.56E-03 | 5.09E-04 | 9 | 0 | FemaleOventralis |
| GO:0060307 | regulation of ventricular cardiac muscle cell membrane repolarization | 6.56E-03 | 5.09E-04 | 9 | 0 | FemaleOventralis |
| GO:0043931 | ossification involved in bone maturation | 8.33E-03 | 6.84E-04 | 11 | 1 | FemaleOventralis |
| GO:0014068 | positive regulation of phosphatidylinositol 3-kinase cascade | 8.72E-03 | 7.26E-04 | 23 | 8 | FemaleOventralis |
| GO:0051149 | positive regulation of muscle cell differentiation | 1.13E-02 | 9.93E-04 | 18 | 5 | FemaleOventralis |
| GO:0060037 | pharyngeal system development | 1.17E-02 | 1.04E-03 | 16 | 4 | FemaleOventralis |
| GO:0090023 | positive regulation of neutrophil chemotaxis | 1.17E-02 | 1.04E-03 | 16 | 4 | FemaleOventralis |
| GO:0006397 | mRNA processing | 1.17E-02 | 1.04E-03 | 37 | 20 | FemaleOventralis |
| GO:0051938 | L-glutamate import | 1.25E-02 | 1.18E-03 | 8 | 0 | FemaleOventralis |
| GO:0032543 | mitochondrial translation | 1.25E-02 | 1.18E-03 | 8 | 0 | FemaleOventralis |
| GO:0019985 | translesion synthesis | 1.25E-02 | 1.18E-03 | 8 | 0 | FemaleOventralis |
| GO:0070779 | D-aspartate import | 1.25E-02 | 1.18E-03 | 8 | 0 | FemaleOventralis |
| GO:0051570 | regulation of histone H3-K9 methylation | 1.25E-02 | 1.18E-03 | 8 | 0 | FemaleOventralis |
| GO:0008345 | larval locomotory behavior | 1.25E-02 | 1.18E-03 | 8 | 0 | FemaleOventralis |
| GO:0070681 | glutaminyl-tRNAGln biosynthesis via transamidation | 1.25E-02 | 1.18E-03 | 8 | 0 | FemaleOventralis |

| | | | | | | |
|---|---|---|---|---|---|---|
| GO:0008292 | acetylcholine biosynthetic process | 1.25E-02 | 1.18E-03 | 8 | 0 | FemaleOventralis |
| GO:0034418 | urate biosynthetic process | 1.25E-02 | 1.18E-03 | 8 | 0 | FemaleOventralis |
| GO:0072673 | lamellipodium morphogenesis | 1.25E-02 | 1.18E-03 | 8 | 0 | FemaleOventralis |
| GO:0030178 | negative regulation of Wnt receptor signaling pathway | 1.45E-02 | 1.41E-03 | 20 | 7 | FemaleOventralis |
| GO:0006094 | gluconeogenesis | 1.49E-02 | 1.47E-03 | 10 | 1 | FemaleOventralis |
| GO:0043407 | negative regulation of MAP kinase activity | 1.49E-02 | 1.47E-03 | 10 | 1 | FemaleOventralis |
| GO:0033590 | response to cobalamin | 2.49E-02 | 2.75E-03 | 7 | 0 | FemaleOventralis |
| GO:0032933 | SREBP signaling pathway | 2.49E-02 | 2.75E-03 | 7 | 0 | FemaleOventralis |
| GO:0006998 | nuclear envelope organization | 2.49E-02 | 2.75E-03 | 7 | 0 | FemaleOventralis |
| GO:0060041 | retina development in camera-type eye | 2.70E-02 | 3.02E-03 | 24 | 11 | FemaleOventralis |
| GO:0035590 | purinergic nucleotide receptor signaling pathway | 2.74E-02 | 3.12E-03 | 9 | 1 | FemaleOventralis |
| GO:0042130 | negative regulation of T cell proliferation | 2.74E-02 | 3.12E-03 | 9 | 1 | FemaleOventralis |
| GO:0043149 | stress fiber assembly | 2.90E-02 | 3.52E-03 | 14 | 4 | FemaleOventralis |
| GO:0071826 | ribonucleoprotein complex subunit organization | 3.00E-02 | 3.66E-03 | 13 | 3 | FemaleOventralis |
| GO:0006144 | purine nucleobase metabolic process | 3.00E-02 | 3.66E-03 | 13 | 3 | FemaleOventralis |
| GO:0022613 | ribonucleoprotein complex biogenesis | 4.54E-02 | 6.39E-03 | 13 | 4 | FemaleOventralis |
| GO:0021535 | cell migration in hindbrain | 4.54E-02 | 6.40E-03 | 6 | 0 | FemaleOventralis |
| GO:0016579 | protein deubiquitination | 4.54E-02 | 6.40E-03 | 6 | 0 | FemaleOventralis |
| GO:0006680 | glucosylceramide catabolic process | 4.54E-02 | 6.40E-03 | 6 | 0 | FemaleOventralis |
| GO:0060039 | pericardium development | 4.54E-02 | 6.40E-03 | 6 | 0 | FemaleOventralis |
| GO:0032776 | DNA methylation on cytosine | 4.54E-02 | 6.40E-03 | 6 | 0 | FemaleOventralis |
| GO:0035306 | positive regulation of dephosphorylation | 4.54E-02 | 6.40E-03 | 6 | 0 | FemaleOventralis |
| GO:0035304 | regulation of protein dephosphorylation | 4.54E-02 | 6.40E-03 | 6 | 0 | FemaleOventralis |
| GO:0051610 | serotonin uptake | 4.54E-02 | 6.40E-03 | 6 | 0 | FemaleOventralis |
| GO:0046621 | negative regulation of organ growth | 4.54E-02 | 6.40E-03 | 6 | 0 | FemaleOventralis |
| GO:0030207 | chondroitin sulfate catabolic process | 4.54E-02 | 6.40E-03 | 6 | 0 | FemaleOventralis |
| GO:0032365 | intracellular lipid transport | 4.54E-02 | 6.40E-03 | 6 | 0 | FemaleOventralis |
| GO:0045840 | positive regulation of mitosis | 4.54E-02 | 6.40E-03 | 6 | 0 | FemaleOventralis |
| GO:0072334 | UDP-galactose transmembrane transport | 4.54E-02 | 6.40E-03 | 6 | 0 | FemaleOventralis |
| GO:0060689 | cell differentiation involved in salivary gland development | 4.54E-02 | 6.51E-03 | 10 | 2 | FemaleOventralis |
| GO:0008645 | hexose transport | 4.54E-02 | 6.62E-03 | 12 | 3 | FemaleOventralis |

**Enriched in Male Tissue of *O. ventralis***

| GO-ID | Term | FDR | P-Value | #FTranscripts | #Mtranscripts | Enriched in |
|---|---|---|---|---|---|---|
| GO:0007411 | axon guidance | 1.77E-05 | 1.21E-07 | 24 | 95 | MaleOventralis |
| GO:0007179 | transforming growth factor beta receptor signaling pathway | 5.06E-05 | 6.15E-07 | 5 | 44 | MaleOventralis |
| GO:0006637 | acyl-CoA metabolic process | 8.01E-05 | 1.07E-06 | 2 | 33 | MaleOventralis |
| GO:0031122 | cytoplasmic microtubule organization | 2.00E-04 | 3.88E-06 | 0 | 23 | MaleOventralis |
| GO:0032436 | positive regulation of proteasomal ubiquitin-dependent protein catabolic process | 2.00E-04 | 3.88E-06 | 0 | 23 | MaleOventralis |
| GO:0050714 | positive regulation of protein secretion | 3.16E-04 | 7.28E-06 | 0 | 21 | MaleOventralis |
| GO:0001666 | response to hypoxia | 3.69E-04 | 9.10E-06 | 3 | 32 | MaleOventralis |
| GO:0009749 | response to glucose stimulus | 3.98E-04 | 1.01E-05 | 1 | 25 | MaleOventralis |
| GO:0007200 | phospholipase C-activating G-protein coupled receptor signaling pathway | 4.77E-04 | 1.31E-05 | 0 | 20 | MaleOventralis |
| GO:0060134 | prepulse inhibition | 4.77E-04 | 1.31E-05 | 0 | 20 | MaleOventralis |
| GO:0043122 | regulation of I-kappaB kinase/NF-kappaB cascade | 4.77E-04 | 1.31E-05 | 0 | 20 | MaleOventralis |
| GO:0034614 | cellular response to reactive oxygen species | 4.77E-04 | 1.31E-05 | 0 | 20 | MaleOventralis |
| GO:0035372 | protein localization to microtubule | 7.72E-04 | 2.38E-05 | 0 | 19 | MaleOventralis |
| GO:0001541 | ovarian follicle development | 7.72E-04 | 2.38E-05 | 0 | 19 | MaleOventralis |
| GO:0071340 | skeletal muscle acetylcholine-gated channel clustering | 7.72E-04 | 2.38E-05 | 0 | 19 | MaleOventralis |
| GO:0008037 | cell recognition | 9.75E-04 | 3.18E-05 | 1 | 23 | MaleOventralis |
| GO:0046135 | pyrimidine nucleoside catabolic process | 1.21E-03 | 4.33E-05 | 0 | 18 | MaleOventralis |
| GO:0035270 | endocrine system development | 1.49E-03 | 5.70E-05 | 1 | 22 | MaleOventralis |
| GO:0007218 | neuropeptide signaling pathway | 1.94E-03 | 7.93E-05 | 0 | 17 | MaleOventralis |
| GO:0030574 | collagen catabolic process | 1.94E-03 | 7.93E-05 | 0 | 17 | MaleOventralis |
| GO:0022617 | extracellular matrix disassembly | 1.94E-03 | 7.93E-05 | 0 | 17 | MaleOventralis |
| GO:2000021 | regulation of ion homeostasis | 1.94E-03 | 7.93E-05 | 0 | 17 | MaleOventralis |
| GO:0051607 | defense response to virus | 2.14E-03 | 9.81E-05 | 2 | 24 | MaleOventralis |
| GO:0032355 | response to estradiol stimulus | 2.20E-03 | 1.03E-04 | 1 | 21 | MaleOventralis |
| GO:0048041 | focal adhesion assembly | 3.22E-03 | 1.66E-04 | 2 | 23 | MaleOventralis |
| GO:0030336 | negative regulation of cell migration | 3.22E-03 | 1.66E-04 | 2 | 23 | MaleOventralis |
| GO:0051384 | response to glucocorticoid stimulus | 3.22E-03 | 1.66E-04 | 2 | 23 | MaleOventralis |
| GO:0007603 | phototransduction, visible light | 3.54E-03 | 1.85E-04 | 1 | 20 | MaleOventralis |
| GO:0032091 | negative regulation of protein binding | 3.54E-03 | 1.85E-04 | 1 | 20 | MaleOventralis |
| GO:0060562 | epithelial tube morphogenesis | 3.77E-03 | 1.99E-04 | 15 | 54 | MaleOventralis |
| GO:0051604 | protein maturation | 5.39E-03 | 3.35E-04 | 1 | 19 | MaleOventralis |
| GO:0021761 | limbic system development | 5.39E-03 | 3.35E-04 | 1 | 19 | MaleOventralis |
| GO:0016050 | vesicle organization | 6.56E-03 | 4.21E-04 | 5 | 30 | MaleOventralis |
| GO:0008543 | fibroblast growth factor receptor signaling pathway | 6.56E-03 | 4.26E-04 | 6 | 32 | MaleOventralis |
| GO:0045786 | negative regulation of cell cycle | 6.56E-03 | 4.74E-04 | 11 | 43 | MaleOventralis |
| GO:0021675 | nerve development | 6.56E-03 | 4.79E-04 | 2 | 21 | MaleOventralis |
| GO:0019233 | sensory perception of pain | 6.56E-03 | 5.06E-04 | 0 | 14 | MaleOventralis |
| GO:0007193 | adenylate cyclase-inhibiting G-protein coupled receptor signaling pathway | 6.56E-03 | 5.06E-04 | 0 | 14 | MaleOventralis |
| GO:0046627 | negative regulation of insulin receptor signaling pathway | 6.56E-03 | 5.06E-04 | 0 | 14 | MaleOventralis |
| GO:0018958 | phenol-containing compound metabolic process | 6.56E-03 | 5.06E-04 | 0 | 14 | MaleOventralis |
| GO:0051044 | positive regulation of membrane protein ectodomain proteolysis | 6.56E-03 | 5.06E-04 | 0 | 14 | MaleOventralis |
| GO:0060333 | interferon-gamma-mediated signaling pathway | 6.56E-03 | 5.06E-04 | 0 | 14 | MaleOventralis |
| GO:0071222 | cellular response to lipopolysaccharide | 7.67E-03 | 6.09E-04 | 1 | 18 | MaleOventralis |
| GO:0006206 | pyrimidine nucleobase metabolic process | 7.67E-03 | 6.09E-04 | 1 | 18 | MaleOventralis |
| GO:0050727 | regulation of inflammatory response | 7.67E-03 | 6.09E-04 | 1 | 18 | MaleOventralis |
| GO:0007605 | sensory perception of sound | 8.28E-03 | 6.69E-04 | 6 | 31 | MaleOventralis |
| GO:0030866 | cortical actin cytoskeleton organization | 1.10E-02 | 9.53E-04 | 0 | 13 | MaleOventralis |
| GO:0006688 | glycosphingolipid biosynthetic process | 1.10E-02 | 9.53E-04 | 0 | 13 | MaleOventralis |
| GO:0000956 | nuclear-transcribed mRNA catabolic process | 1.10E-02 | 9.53E-04 | 0 | 13 | MaleOventralis |
| GO:0030282 | bone mineralization | 1.10E-02 | 9.53E-04 | 0 | 13 | MaleOventralis |
| GO:0048483 | autonomic nervous system development | 1.10E-02 | 9.53E-04 | 0 | 13 | MaleOventralis |
| GO:0015014 | heparan sulfate proteoglycan biosynthetic process, polysaccharide chain biosynthetic process | 1.10E-02 | 9.53E-04 | 0 | 13 | MaleOventralis |
| GO:0033630 | positive regulation of cell adhesion mediated by integrin | 1.10E-02 | 9.53E-04 | 0 | 13 | MaleOventralis |
| GO:0060350 | endochondral bone morphogenesis | 1.10E-02 | 9.53E-04 | 0 | 13 | MaleOventralis |
| GO:0007565 | female pregnancy | 1.14E-02 | 1.00E-03 | 3 | 23 | MaleOventralis |
| GO:0001755 | neural crest cell migration | 1.20E-02 | 1.07E-03 | 1 | 16 | MaleOventralis |
| GO:0051899 | membrane depolarization | 1.23E-02 | 1.11E-03 | 1 | 17 | MaleOventralis |
| GO:0022007 | convergent extension involved in neural plate elongation | 1.43E-02 | 1.39E-03 | 2 | 19 | MaleOventralis |
| GO:0042455 | ribonucleoside biosynthetic process | 1.43E-02 | 1.39E-03 | 2 | 19 | MaleOventralis |
| GO:0045761 | regulation of adenylate cyclase activity | 1.43E-02 | 1.39E-03 | 2 | 19 | MaleOventralis |
| GO:0045088 | regulation of innate immune response | 1.46E-02 | 1.42E-03 | 8 | 34 | MaleOventralis |
| GO:0071214 | cellular response to abiotic stimulus | 1.46E-02 | 1.42E-03 | 8 | 34 | MaleOventralis |
| GO:0007611 | learning or memory | 1.73E-02 | 1.75E-03 | 5 | 26 | MaleOventralis |
| GO:0033555 | multicellular organismal response to stress | 1.75E-02 | 1.81E-03 | 0 | 12 | MaleOventralis |
| GO:0015804 | neutral amino acid transport | 1.75E-02 | 1.81E-03 | 0 | 12 | MaleOventralis |
| GO:0045010 | actin nucleation | 1.75E-02 | 1.81E-03 | 0 | 12 | MaleOventralis |

| | | | | | | |
|---|---|---|---|---|---|---|
| GO:0030210 | heparin biosynthetic process | 1.75E-02 | 1.81E-03 | 0 | 12 | MaleOventralis |
| GO:0022600 | digestive system process | 1.75E-02 | 1.81E-03 | 0 | 12 | MaleOventralis |
| GO:0048799 | organ maturation | 1.75E-02 | 1.81E-03 | 0 | 12 | MaleOventralis |
| GO:0050872 | white fat cell differentiation | 1.75E-02 | 1.81E-03 | 0 | 12 | MaleOventralis |
| GO:0043647 | inositol phosphate metabolic process | 1.75E-02 | 1.81E-03 | 0 | 12 | MaleOventralis |
| GO:0033333 | fin development | 1.78E-02 | 1.85E-03 | 1 | 15 | MaleOventralis |
| GO:0042542 | response to hydrogen peroxide | 1.78E-02 | 1.85E-03 | 1 | 15 | MaleOventralis |
| GO:0010839 | negative regulation of keratinocyte proliferation | 1.78E-02 | 1.85E-03 | 1 | 15 | MaleOventralis |
| GO:0090100 | positive regulation of transmembrane receptor protein serine/threonine kinase signaling pathway | 1.78E-02 | 1.85E-03 | 1 | 15 | MaleOventralis |
| GO:0006898 | receptor-mediated endocytosis | 2.06E-02 | 2.17E-03 | 8 | 33 | MaleOventralis |
| GO:0010675 | regulation of cellular carbohydrate metabolic process | 2.21E-02 | 2.37E-03 | 2 | 18 | MaleOventralis |
| GO:0030216 | keratinocyte differentiation | 2.21E-02 | 2.37E-03 | 2 | 18 | MaleOventralis |
| GO:0034394 | protein localization to cell surface | 2.21E-02 | 2.37E-03 | 2 | 18 | MaleOventralis |
| GO:0006486 | protein glycosylation | 2.59E-02 | 2.90E-03 | 9 | 34 | MaleOventralis |
| GO:0042593 | glucose homeostasis | 2.78E-02 | 3.21E-03 | 1 | 14 | MaleOventralis |
| GO:0032526 | response to retinoic acid | 2.78E-02 | 3.21E-03 | 1 | 14 | MaleOventralis |
| GO:0048701 | embryonic cranial skeleton morphogenesis | 2.78E-02 | 3.21E-03 | 1 | 14 | MaleOventralis |
| GO:0042157 | lipoprotein metabolic process | 2.78E-02 | 3.21E-03 | 1 | 14 | MaleOventralis |
| GO:0051154 | negative regulation of striated muscle cell differentiation | 2.78E-02 | 3.21E-03 | 1 | 14 | MaleOventralis |
| GO:0010042 | response to manganese ion | 2.84E-02 | 3.45E-03 | 0 | 11 | MaleOventralis |
| GO:0035710 | CD4-positive, alpha-beta T cell activation | 2.84E-02 | 3.45E-03 | 0 | 11 | MaleOventralis |
| GO:0019276 | UDP-N-acetylgalactosamine metabolic process | 2.84E-02 | 3.45E-03 | 0 | 11 | MaleOventralis |
| GO:0002062 | chondrocyte differentiation | 2.84E-02 | 3.45E-03 | 0 | 11 | MaleOventralis |
| GO:0042311 | vasodilation | 2.84E-02 | 3.45E-03 | 0 | 11 | MaleOventralis |
| GO:0019935 | cyclic-nucleotide-mediated signaling | 2.84E-02 | 3.45E-03 | 0 | 11 | MaleOventralis |
| GO:0046686 | response to cadmium ion | 2.84E-02 | 3.45E-03 | 0 | 11 | MaleOventralis |
| GO:0042117 | monocyte activation | 2.84E-02 | 3.45E-03 | 0 | 11 | MaleOventralis |
| GO:0046398 | UDP-glucuronate metabolic process | 2.84E-02 | 3.45E-03 | 0 | 11 | MaleOventralis |
| GO:0072593 | reactive oxygen species metabolic process | 2.84E-02 | 3.45E-03 | 0 | 11 | MaleOventralis |
| GO:0009744 | response to sucrose stimulus | 2.84E-02 | 3.45E-03 | 0 | 11 | MaleOventralis |
| GO:0034241 | positive regulation of macrophage fusion | 2.84E-02 | 3.45E-03 | 0 | 11 | MaleOventralis |
| GO:0050653 | chondroitin sulfate proteoglycan biosynthetic process, polysaccharide chain biosynthetic process | 2.84E-02 | 3.45E-03 | 0 | 11 | MaleOventralis |
| GO:0051088 | PMA-inducible membrane protein ectodomain proteolysis | 2.84E-02 | 3.45E-03 | 0 | 11 | MaleOventralis |
| GO:0035855 | megakaryocyte development | 2.84E-02 | 3.45E-03 | 0 | 11 | MaleOventralis |
| GO:0045600 | positive regulation of fat cell differentiation | 2.84E-02 | 3.45E-03 | 0 | 11 | MaleOventralis |
| GO:0017158 | regulation of calcium ion-dependent exocytosis | 2.84E-02 | 3.45E-03 | 0 | 11 | MaleOventralis |
| GO:0030522 | intracellular receptor signaling pathway | 3.26E-02 | 3.99E-03 | 6 | 27 | MaleOventralis |
| GO:0030819 | positive regulation of cAMP biosynthetic process | 3.27E-02 | 4.03E-03 | 2 | 17 | MaleOventralis |
| GO:0002262 | myeloid cell homeostasis | 3.52E-02 | 4.40E-03 | 3 | 19 | MaleOventralis |
| GO:0090103 | cochlea morphogenesis | 3.52E-02 | 4.40E-03 | 3 | 19 | MaleOventralis |
| GO:0001837 | epithelial to mesenchymal transition | 3.54E-02 | 4.43E-03 | 4 | 22 | MaleOventralis |
| GO:0008284 | positive regulation of cell proliferation | 4.06E-02 | 5.20E-03 | 35 | 80 | MaleOventralis |
| GO:0006081 | cellular aldehyde metabolic process | 4.31E-02 | 5.56E-03 | 1 | 13 | MaleOventralis |
| GO:0046131 | pyrimidine ribonucleoside metabolic process | 4.31E-02 | 5.56E-03 | 1 | 13 | MaleOventralis |
| GO:0007269 | neurotransmitter secretion | 4.54E-02 | 6.05E-03 | 14 | 42 | MaleOventralis |
| GO:0010634 | positive regulation of epithelial cell migration | 4.54E-02 | 6.18E-03 | 6 | 26 | MaleOventralis |
| GO:0002444 | myeloid leukocyte mediated immunity | 4.54E-02 | 6.61E-03 | 0 | 10 | MaleOventralis |
| GO:0050482 | arachidonic acid secretion | 4.54E-02 | 6.61E-03 | 0 | 10 | MaleOventralis |
| GO:0007189 | adenylate cyclase-activating G-protein coupled receptor signaling pathway | 4.54E-02 | 6.61E-03 | 0 | 10 | MaleOventralis |
| GO:0007141 | male meiosis I | 4.54E-02 | 6.61E-03 | 0 | 10 | MaleOventralis |
| GO:0006656 | phosphatidylcholine biosynthetic process | 4.54E-02 | 6.61E-03 | 0 | 10 | MaleOventralis |
| GO:0032851 | positive regulation of Rab GTPase activity | 4.54E-02 | 6.61E-03 | 0 | 10 | MaleOventralis |
| GO:0035458 | cellular response to interferon-beta | 4.54E-02 | 6.61E-03 | 0 | 10 | MaleOventralis |
| GO:0042572 | retinol metabolic process | 4.54E-02 | 6.61E-03 | 0 | 10 | MaleOventralis |
| GO:0032728 | positive regulation of interferon-beta production | 4.54E-02 | 6.61E-03 | 0 | 10 | MaleOventralis |
| GO:0030516 | regulation of axon extension | 4.54E-02 | 6.61E-03 | 0 | 10 | MaleOventralis |
| GO:0016236 | macroautophagy | 4.54E-02 | 6.61E-03 | 0 | 10 | MaleOventralis |
| GO:0070934 | CRD-mediated mRNA stabilization | 4.54E-02 | 6.61E-03 | 0 | 10 | MaleOventralis |
| GO:0046835 | carbohydrate phosphorylation | 4.54E-02 | 6.61E-03 | 0 | 10 | MaleOventralis |
| GO:0045055 | regulated secretory pathway | 4.54E-02 | 6.61E-03 | 0 | 10 | MaleOventralis |
| GO:0033002 | muscle cell proliferation | 4.54E-02 | 6.61E-03 | 0 | 10 | MaleOventralis |
| GO:0042307 | positive regulation of protein import into nucleus | 4.54E-02 | 6.61E-03 | 0 | 10 | MaleOventralis |
| GO:0051602 | response to electrical stimulus | 4.54E-02 | 6.61E-03 | 0 | 10 | MaleOventralis |
| GO:0034502 | protein localization to chromosome | 4.54E-02 | 6.61E-03 | 0 | 10 | MaleOventralis |
| GO:0050873 | brown fat cell differentiation | 4.54E-02 | 6.61E-03 | 0 | 10 | MaleOventralis |
| GO:0033762 | response to glucagon stimulus | 4.54E-02 | 6.61E-03 | 0 | 10 | MaleOventralis |
| GO:0060402 | calcium ion transport into cytosol | 4.54E-02 | 6.61E-03 | 0 | 10 | MaleOventralis |
| GO:0002573 | myeloid leukocyte differentiation | 4.54E-02 | 6.61E-03 | 0 | 10 | MaleOventralis |
| GO:0060337 | type I interferon-mediated signaling pathway | 4.54E-02 | 6.61E-03 | 0 | 10 | MaleOventralis |
| GO:0019079 | viral genome replication | 4.68E-02 | 6.85E-03 | 2 | 16 | MaleOventralis |
| GO:0002065 | columnar/cuboidal epithelial cell differentiation | 4.68E-02 | 6.85E-03 | 2 | 16 | MaleOventralis |
| GO:0043112 | receptor metabolic process | 4.84E-02 | 7.11E-03 | 3 | 18 | MaleOventralis |
| GO:0045765 | regulation of angiogenesis | 4.84E-02 | 7.11E-03 | 3 | 18 | MaleOventralis |
| GO:0048284 | organelle fusion | 4.84E-02 | 7.11E-03 | 3 | 18 | MaleOventralis |
| GO:0042246 | tissue regeneration | 4.84E-02 | 7.11E-03 | 4 | 21 | MaleOventralis |

| Color codes | |
|---|---|
| | involved in transcription |
| | involved in apoptosis |
| | wnt signaling pathway |
| | TGFbeta superfamily |
| | new candidates |

| **GO** | **GO term** |
|---|---|
| **FEMALE 4 species** | |
| GO:0001880 | Mullerian duct regression, , contains transcripts of the following genes |
| | mothers against decapentaplegic homolog 5 |
| | mothers against decapentaplegic homolog 9 |
| **MALE 4 species** | |
| GO:0043570 | maintenance of DNA repeat elements, contains transcripts of the following genes |
| | transcription factor 7-like 2-like |
| GO:0032350 | regulation of hormone metabolic process, contains transcripts of the following genes |
| | transcription factor 7-like 2-like |
| GO:0048545 | response to steroid hormone stimulus, contains transcripts of the following genes |
| | transcription factor 7-like 2-like |
| | membrane progestin receptor alpha |
| | aspartate cytoplasmic |
| | lysyl oxidase |
| | pyridoxal kinase |

desmoglein-2
transforming growth factor beta-3
nuclear receptor subfamily 2 group c member 2
sodium-dependent phosphate transport protein 2b
vitamin d3 receptor
cyclin-dependent kinase 2
plasma membrane calcium-transporting atpase 1-like
high mobility group protein b2
extracellular serine threonine protein kinase fam20c-like
protein kinase c alpha type
rotein kinase c alpha type
long-chain-fatty-acid-ligase acsbg2
neurogenic locus notch homolog protein 1
somatostatin receptor type 2
arginase-1
voltage-dependent n-type calcium channel subunit alpha-1b-like

## Supplementary Material Table 9

**Enriched in Ovary in all four species**

| GO-ID | Term | FDR | P-Value | #OTranscripts | #TTranscripts | Enriched in |
|---|---|---|---|---|---|---|
| GO:0031532 | actin cytoskeleton reorganization | 1.02E-13 | 9.72E-16 | 110 | 17 | Ovary4Species |
| GO:0016339 | calcium-dependent cell-cell adhesion | 2.33E-13 | 2.45E-15 | 53 | 0 | Ovary4Species |
| GO:0090136 | epithelial cell-cell adhesion | 4.24E-13 | 4.68E-15 | 52 | 0 | Ovary4Species |
| GO:0042632 | cholesterol homeostasis | 7.42E-13 | 8.85E-15 | 72 | 5 | Ovary4Species |
| GO:0034375 | high-density lipoprotein particle remodeling | 7.42E-13 | 8.96E-15 | 51 | 0 | Ovary4Species |
| GO:0006828 | manganese ion transport | 4.27E-12 | 6.32E-14 | 48 | 0 | Ovary4Species |
| GO:0032472 | Golgi calcium ion transport | 4.27E-12 | 6.32E-14 | 48 | 0 | Ovary4Species |
| GO:0032468 | Golgi calcium ion homeostasis | 4.27E-12 | 6.32E-14 | 48 | 0 | Ovary4Species |
| GO:0030026 | cellular manganese ion homeostasis | 4.27E-12 | 6.32E-14 | 48 | 0 | Ovary4Species |
| GO:0006516 | glycoprotein catabolic process | 1.44E-11 | 2.34E-13 | 46 | 0 | Ovary4Species |
| GO:0051764 | actin crosslink formation | 1.44E-11 | 2.34E-13 | 46 | 0 | Ovary4Species |
| GO:0045218 | zonula adherens maintenance | 4.61E-11 | 8.68E-13 | 44 | 0 | Ovary4Species |
| GO:0043691 | reverse cholesterol transport | 2.03E-10 | 4.49E-12 | 51 | 2 | Ovary4Species |
| GO:0010983 | positive regulation of high-density lipoprotein particle clearance | 2.72E-10 | 6.28E-12 | 41 | 0 | Ovary4Species |
| GO:0032148 | activation of protein kinase B activity | 2.92E-09 | 8.99E-11 | 37 | 0 | Ovary4Species |
| GO:0007077 | mitotic nuclear envelope disassembly | 1.02E-08 | 3.44E-10 | 35 | 0 | Ovary4Species |
| GO:0001829 | trophectodermal cell differentiation | 3.66E-08 | 1.32E-09 | 33 | 0 | Ovary4Species |
| GO:0019441 | tryptophan catabolic process to kynurenine | 6.73E-08 | 2.60E-09 | 32 | 0 | Ovary4Species |
| GO:0034354 | 'de novo' NAD biosynthetic process from tryptophan | 6.73E-08 | 2.60E-09 | 32 | 0 | Ovary4Species |
| GO:0032376 | positive regulation of cholesterol transport | 9.66E-08 | 3.82E-09 | 46 | 4 | Ovary4Species |
| GO:0033387 | putrescine biosynthetic process from ornithine | 4.30E-07 | 1.92E-08 | 28 | 0 | Ovary4Species |
| GO:0048149 | behavioral response to ethanol | 1.37E-06 | 6.93E-08 | 26 | 0 | Ovary4Species |
| GO:0016578 | histone deubiquitination | 2.30E-06 | 1.20E-07 | 30 | 1 | Ovary4Species |
| GO:0060261 | positive regulation of transcription initiation from RNA polymerase II promoter | 2.43E-06 | 1.32E-07 | 25 | 0 | Ovary4Species |
| GO:0055091 | phospholipid homeostasis | 3.68E-06 | 2.05E-07 | 44 | 6 | Ovary4Species |
| GO:0036336 | dendritic cell migration | 4.03E-06 | 2.27E-07 | 29 | 1 | Ovary4Species |
| GO:0050746 | regulation of lipoprotein metabolic process | 7.90E-06 | 4.70E-07 | 31 | 2 | Ovary4Species |
| GO:0051016 | barbed-end actin filament capping | 7.90E-06 | 4.70E-07 | 31 | 2 | Ovary4Species |
| GO:1900222 | negative regulation of beta-amyloid clearance | 7.93E-06 | 4.76E-07 | 23 | 0 | Ovary4Species |
| GO:0010735 | positive regulation of transcription via serum response element binding | 7.93E-06 | 4.76E-07 | 23 | 0 | Ovary4Species |
| GO:0003257 | positive regulation of transcription from RNA polymerase II promoter involved in myocardial precursor cell differentiation | 7.93E-06 | 4.76E-07 | 23 | 0 | Ovary4Species |
| GO:0000046 | autophagic vacuole fusion | 1.40E-05 | 9.07E-07 | 22 | 0 | Ovary4Species |
| GO:0035046 | pronuclear migration | 1.40E-05 | 9.07E-07 | 22 | 0 | Ovary4Species |
| GO:0006636 | unsaturated fatty acid biosynthetic process | 2.47E-05 | 1.73E-06 | 21 | 0 | Ovary4Species |
| GO:0006501 | C-terminal protein lipidation | 2.47E-05 | 1.73E-06 | 21 | 0 | Ovary4Species |
| GO:0048490 | anterograde synaptic vesicle transport | 2.47E-05 | 1.73E-06 | 21 | 0 | Ovary4Species |
| GO:0071466 | cellular response to xenobiotic stimulus | 2.99E-05 | 2.13E-06 | 55 | 13 | Ovary4Species |
| GO:0001522 | pseudouridine synthesis | 4.36E-05 | 3.30E-06 | 20 | 0 | Ovary4Species |
| GO:0016322 | neuron remodeling | 4.36E-05 | 3.30E-06 | 20 | 0 | Ovary4Species |
| GO:0072709 | cellular response to sorbitol | 4.36E-05 | 3.30E-06 | 20 | 0 | Ovary4Species |
| GO:0043123 | positive regulation of I-kappaB kinase/NF-kappaB cascade | 7.06E-05 | 5.69E-06 | 112 | 46 | Ovary4Species |
| GO:0051382 | kinetochore assembly | 7.65E-05 | 6.29E-06 | 19 | 0 | Ovary4Species |
| GO:0016254 | preassembly of GPI anchor in ER membrane | 7.65E-05 | 6.29E-06 | 19 | 0 | Ovary4Species |
| GO:0015701 | bicarbonate transport | 1.10E-04 | 9.40E-06 | 26 | 2 | Ovary4Species |
| GO:0006450 | regulation of translational fidelity | 1.36E-04 | 1.20E-05 | 18 | 0 | Ovary4Species |
| GO:0010827 | regulation of glucose transport | 1.43E-04 | 1.27E-05 | 51 | 13 | Ovary4Species |
| GO:0010862 | positive regulation of pathway-restricted SMAD protein phosphorylation | 1.44E-04 | 1.29E-05 | 33 | 5 | Ovary4Species |
| GO:0006626 | protein targeting to mitochondrion | 1.52E-04 | 1.37E-05 | 28 | 3 | Ovary4Species |
| GO:0030216 | keratinocyte differentiation | 1.57E-04 | 1.42E-05 | 36 | 6 | Ovary4Species |
| GO:0070613 | regulation of protein processing | 2.09E-04 | 1.95E-05 | 22 | 1 | Ovary4Species |
| GO:0000290 | deadenylation-dependent decapping of nuclear-transcribed mRNA | 2.43E-04 | 2.30E-05 | 17 | 0 | Ovary4Species |
| GO:0060391 | positive regulation of SMAD protein import into nucleus | 3.12E-04 | 3.06E-05 | 29 | 4 | Ovary4Species |
| GO:0009409 | response to cold | 3.12E-04 | 3.06E-05 | 29 | 4 | Ovary4Species |
| GO:0045746 | negative regulation of Notch signaling pathway | 3.49E-04 | 3.46E-05 | 20 | 1 | Ovary4Species |
| GO:0010458 | exit from mitosis | 3.97E-04 | 4.01E-05 | 36 | 7 | Ovary4Species |
| GO:0070358 | actin polymerization-dependent cell motility | 4.14E-04 | 4.22E-05 | 26 | 3 | Ovary4Species |
| GO:0055010 | ventricular cardiac muscle tissue morphogenesis | 4.14E-04 | 4.22E-05 | 26 | 3 | Ovary4Species |
| GO:0016266 | O-glycan processing | 4.14E-04 | 4.22E-05 | 26 | 3 | Ovary4Species |
| GO:0006975 | DNA damage induced protein phosphorylation | 4.30E-04 | 4.41E-05 | 16 | 0 | Ovary4Species |
| GO:0051660 | establishment of centrosome localization | 4.30E-04 | 4.41E-05 | 16 | 0 | Ovary4Species |
| GO:0008203 | cholesterol metabolic process | 7.02E-04 | 7.71E-05 | 58 | 19 | Ovary4Species |
| GO:0006851 | mitochondrial calcium ion transport | 7.61E-04 | 8.46E-05 | 15 | 0 | Ovary4Species |
| GO:0030220 | platelet formation | 7.61E-04 | 8.46E-05 | 15 | 0 | Ovary4Species |
| GO:2001243 | negative regulation of intrinsic apoptotic signaling pathway | 8.86E-04 | 1.01E-04 | 22 | 2 | Ovary4Species |
| GO:0045665 | negative regulation of neuron differentiation | 9.34E-04 | 1.07E-04 | 34 | 7 | Ovary4Species |
| GO:0045669 | positive regulation of osteoblast differentiation | 1.08E-03 | 1.29E-04 | 24 | 3 | Ovary4Species |
| GO:0051150 | regulation of smooth muscle cell differentiation | 1.08E-03 | 1.29E-04 | 24 | 3 | Ovary4Species |
| GO:0009311 | oligosaccharide metabolic process | 1.24E-03 | 1.51E-04 | 26 | 4 | Ovary4Species |
| GO:0021747 | cochlear nucleus development | 1.32E-03 | 1.63E-04 | 14 | 0 | Ovary4Species |
| GO:0072428 | signal transduction involved in intra-S DNA damage checkpoint | 1.32E-03 | 1.63E-04 | 14 | 0 | Ovary4Species |
| GO:0001880 | Mullerian duct regression | 1.32E-03 | 1.63E-04 | 14 | 0 | Ovary4Species |
| GO:0032981 | mitochondrial respiratory chain complex I assembly | 1.32E-03 | 1.63E-04 | 14 | 0 | Ovary4Species |
| GO:0007084 | mitotic nuclear envelope reassembly | 1.32E-03 | 1.63E-04 | 14 | 0 | Ovary4Species |
| GO:0006438 | valyl-tRNA aminoacylation | 1.32E-03 | 1.63E-04 | 14 | 0 | Ovary4Species |
| GO:0071257 | cellular response to electrical stimulus | 1.32E-03 | 1.63E-04 | 14 | 0 | Ovary4Species |
| GO:0006044 | N-acetylglucosamine metabolic process | 1.32E-03 | 1.63E-04 | 14 | 0 | Ovary4Species |
| GO:0043525 | positive regulation of neuron apoptotic process | 1.88E-03 | 2.45E-04 | 36 | 9 | Ovary4Species |
| GO:0045987 | positive regulation of smooth muscle contraction | 1.94E-03 | 2.55E-04 | 25 | 4 | Ovary4Species |
| GO:0033238 | regulation of cellular amine metabolic process | 2.15E-03 | 2.86E-04 | 29 | 6 | Ovary4Species |
| GO:0045454 | cell redox homeostasis | 2.19E-03 | 2.93E-04 | 42 | 12 | Ovary4Species |
| GO:0048382 | mesendoderm development | 2.30E-03 | 3.13E-04 | 13 | 0 | Ovary4Species |
| GO:0051044 | positive regulation of membrane protein ectodomain proteolysis | 2.30E-03 | 3.13E-04 | 13 | 0 | Ovary4Species |
| GO:0035357 | peroxisome proliferator activated receptor signaling pathway | 2.30E-03 | 3.13E-04 | 13 | 0 | Ovary4Species |
| GO:0010591 | regulation of lamellipodium assembly | 2.30E-03 | 3.13E-04 | 13 | 0 | Ovary4Species |
| GO:0010510 | regulation of acetyl-CoA biosynthetic process from pyruvate | 2.30E-03 | 3.13E-04 | 13 | 0 | Ovary4Species |
| GO:0060687 | regulation of branching involved in prostate gland morphogenesis | 2.30E-03 | 3.13E-04 | 13 | 0 | Ovary4Species |
| GO:0002175 | protein localization to paranode region of axon | 2.75E-03 | 3.82E-04 | 16 | 1 | Ovary4Species |
| GO:2000114 | regulation of establishment of cell polarity | 2.75E-03 | 3.82E-04 | 16 | 1 | Ovary4Species |
| GO:0006360 | transcription from RNA polymerase I promoter | 2.78E-03 | 3.87E-04 | 22 | 3 | Ovary4Species |
| GO:0015698 | inorganic anion transport | 2.97E-03 | 4.14E-04 | 52 | 18 | Ovary4Species |
| GO:0070423 | nucleotide-binding oligomerization domain containing signaling pathway | 3.07E-03 | 4.29E-04 | 24 | 4 | Ovary4Species |
| GO:0048333 | mesodermal cell differentiation | 3.21E-03 | 4.52E-04 | 31 | 7 | Ovary4Species |
| GO:0006919 | activation of cysteine-type endopeptidase activity involved in apoptotic process | 3.26E-03 | 4.60E-04 | 28 | 6 | Ovary4Species |
| GO:0043967 | histone H4 acetylation | 3.43E-03 | 4.86E-04 | 38 | 11 | Ovary4Species |
| GO:0060119 | inner ear receptor cell development | 3.88E-03 | 5.78E-04 | 42 | 13 | Ovary4Species |
| GO:0007016 | cytoskeletal anchoring at plasma membrane | 3.88E-03 | 5.80E-04 | 19 | 2 | Ovary4Species |
| GO:0021799 | cerebral cortex radially oriented cell migration | 3.97E-03 | 6.05E-04 | 12 | 0 | Ovary4Species |
| GO:0019373 | epoxygenase P450 pathway | 3.97E-03 | 6.05E-04 | 12 | 0 | Ovary4Species |
| GO:0006546 | glycine catabolic process | 3.97E-03 | 6.05E-04 | 12 | 0 | Ovary4Species |
| GO:0014036 | neural crest cell fate specification | 3.97E-03 | 6.05E-04 | 12 | 0 | Ovary4Species |

| | | | | | | |
|---|---|---|---|---|---|---|
| GO:2000096 | positive regulation of Wnt receptor signaling pathway, planar cell polarity pathway | 3.97E-03 | 6.05E-04 | 12 | 0 | Ovary4Species |
| GO:0016446 | somatic hypermutation of immunoglobulin genes | 3.97E-03 | 6.05E-04 | 12 | 0 | Ovary4Species |
| GO:0010507 | negative regulation of autophagy | 3.97E-03 | 6.05E-04 | 12 | 0 | Ovary4Species |
| GO:0046386 | deoxyribose phosphate catabolic process | 3.97E-03 | 6.05E-04 | 12 | 0 | Ovary4Species |
| GO:0045604 | regulation of epidermal cell differentiation | 4.35E-03 | 6.68E-04 | 21 | 3 | Ovary4Species |
| GO:0030071 | regulation of mitotic metaphase/anaphase transition | 4.35E-03 | 6.68E-04 | 21 | 3 | Ovary4Species |
| GO:0033540 | fatty acid beta-oxidation using acyl-CoA oxidase | 4.48E-03 | 6.93E-04 | 15 | 1 | Ovary4Species |
| GO:0006662 | glycerol ether metabolic process | 4.48E-03 | 6.93E-04 | 15 | 1 | Ovary4Species |
| GO:0071205 | protein localization to juxtaparanode region of axon | 4.48E-03 | 6.93E-04 | 15 | 1 | Ovary4Species |
| GO:2000059 | negative regulation of protein ubiquitination involved in ubiquitin-dependent protein catabolic process | 4.48E-03 | 6.93E-04 | 15 | 1 | Ovary4Species |
| GO:1901187 | regulation of ephrin receptor signaling pathway | 4.48E-03 | 6.93E-04 | 15 | 1 | Ovary4Species |
| GO:0071800 | podosome assembly | 4.63E-03 | 7.18E-04 | 23 | 4 | Ovary4Species |
| GO:0051289 | protein homotetramerization | 4.63E-03 | 7.20E-04 | 29 | 7 | Ovary4Species |
| GO:0032526 | response to retinoic acid | 6.16E-03 | 9.80E-04 | 38 | 12 | Ovary4Species |
| GO:0038032 | termination of G-protein coupled receptor signaling pathway | 6.35E-03 | 1.01E-03 | 17 | 2 | Ovary4Species |
| GO:0008585 | female gonad development | 6.35E-03 | 1.01E-03 | 17 | 2 | Ovary4Species |
| GO:0045909 | positive regulation of vasodilation | 6.35E-03 | 1.01E-03 | 17 | 2 | Ovary4Species |
| GO:0051560 | mitochondrial calcium ion homeostasis | 6.45E-03 | 1.03E-03 | 18 | 2 | Ovary4Species |
| GO:0035336 | long-chain fatty-acyl-CoA metabolic process | 6.45E-03 | 1.03E-03 | 18 | 2 | Ovary4Species |
| GO:0008344 | adult locomotory behavior | 6.47E-03 | 1.04E-03 | 50 | 19 | Ovary4Species |
| GO:0032456 | endocytic recycling | 6.79E-03 | 1.15E-03 | 20 | 3 | Ovary4Species |
| GO:2000650 | negative regulation of sodium ion transmembrane transporter activity | 6.79E-03 | 1.17E-03 | 11 | 0 | Ovary4Species |
| GO:0006691 | leukotriene metabolic process | 6.79E-03 | 1.17E-03 | 11 | 0 | Ovary4Species |
| GO:0006660 | phosphatidylserine catabolic process | 6.79E-03 | 1.17E-03 | 11 | 0 | Ovary4Species |
| GO:0051825 | adhesion to other organism involved in symbiotic interaction | 6.79E-03 | 1.17E-03 | 11 | 0 | Ovary4Species |
| GO:2000242 | negative regulation of reproductive process | 6.79E-03 | 1.17E-03 | 11 | 0 | Ovary4Species |
| GO:0006189 | 'de novo' IMP biosynthetic process | 6.79E-03 | 1.17E-03 | 11 | 0 | Ovary4Species |
| GO:0006070 | octanol metabolic process | 6.79E-03 | 1.17E-03 | 11 | 0 | Ovary4Species |
| GO:0006000 | fructose metabolic process | 6.79E-03 | 1.17E-03 | 11 | 0 | Ovary4Species |
| GO:0043654 | recognition of apoptotic cell | 6.79E-03 | 1.17E-03 | 11 | 0 | Ovary4Species |
| GO:0070127 | tRNA aminoacylation for mitochondrial protein translation | 6.79E-03 | 1.17E-03 | 11 | 0 | Ovary4Species |
| GO:0000083 | regulation of transcription involved in G1/S transition of mitotic cell cycle | 6.79E-03 | 1.17E-03 | 11 | 0 | Ovary4Species |
| GO:0043097 | pyrimidine nucleoside salvage | 6.79E-03 | 1.17E-03 | 11 | 0 | Ovary4Species |
| GO:0015811 | L-cystine transport | 6.79E-03 | 1.17E-03 | 11 | 0 | Ovary4Species |
| GO:0010996 | response to auditory stimulus | 6.79E-03 | 1.17E-03 | 11 | 0 | Ovary4Species |
| GO:0010825 | positive regulation of centrosome duplication | 6.79E-03 | 1.17E-03 | 11 | 0 | Ovary4Species |
| GO:0008215 | spermine metabolic process | 6.79E-03 | 1.17E-03 | 11 | 0 | Ovary4Species |
| GO:0060775 | planar cell polarity pathway involved in gastrula mediolateral intercalation | 6.79E-03 | 1.17E-03 | 11 | 0 | Ovary4Species |
| GO:0060766 | negative regulation of androgen receptor signaling pathway | 6.79E-03 | 1.17E-03 | 11 | 0 | Ovary4Species |
| GO:0019682 | glyceraldehyde-3-phosphate metabolic process | 6.79E-03 | 1.17E-03 | 11 | 0 | Ovary4Species |
| GO:0060601 | lateral sprouting from an epithelium | 6.79E-03 | 1.17E-03 | 11 | 0 | Ovary4Species |
| GO:0046292 | formaldehyde metabolic process | 6.79E-03 | 1.17E-03 | 11 | 0 | Ovary4Species |
| GO:0046272 | stilbene catabolic process | 6.79E-03 | 1.17E-03 | 11 | 0 | Ovary4Species |
| GO:0060216 | definitive hemopoiesis | 6.82E-03 | 1.18E-03 | 26 | 6 | Ovary4Species |
| GO:0050434 | positive regulation of viral transcription | 8.12E-03 | 1.42E-03 | 32 | 9 | Ovary4Species |
| GO:0043217 | myelin maintenance | 9.87E-03 | 1.76E-03 | 16 | 2 | Ovary4Species |
| GO:0070482 | response to oxygen levels | 9.87E-03 | 1.76E-03 | 114 | 62 | Ovary4Species |
| GO:0042789 | mRNA transcription from RNA polymerase II promoter | 1.09E-02 | 1.95E-03 | 23 | 5 | Ovary4Species |
| GO:0010332 | response to gamma radiation | 1.09E-02 | 1.95E-03 | 23 | 5 | Ovary4Species |
| GO:0060841 | venous blood vessel development | 1.09E-02 | 1.96E-03 | 19 | 3 | Ovary4Species |
| GO:0051220 | cytoplasmic sequestering of protein | 1.10E-02 | 1.99E-03 | 21 | 4 | Ovary4Species |
| GO:0021529 | spinal cord oligodendrocyte cell differentiation | 1.22E-02 | 2.27E-03 | 10 | 0 | Ovary4Species |
| GO:2000641 | regulation of early endosome to late endosome transport | 1.22E-02 | 2.27E-03 | 10 | 0 | Ovary4Species |
| GO:0006421 | asparaginyl-tRNA aminoacylation | 1.22E-02 | 2.27E-03 | 10 | 0 | Ovary4Species |
| GO:2000370 | positive regulation of clathrin-mediated endocytosis | 1.22E-02 | 2.27E-03 | 10 | 0 | Ovary4Species |
| GO:0032438 | melanosome organization | 1.22E-02 | 2.27E-03 | 10 | 0 | Ovary4Species |
| GO:0032415 | regulation of sodium:hydrogen antiporter activity | 1.22E-02 | 2.27E-03 | 10 | 0 | Ovary4Species |
| GO:2000015 | regulation of determination of dorsal identity | 1.22E-02 | 2.27E-03 | 10 | 0 | Ovary4Species |
| GO:0070265 | necrotic cell death | 1.22E-02 | 2.27E-03 | 10 | 0 | Ovary4Species |
| GO:0000154 | rRNA modification | 1.22E-02 | 2.27E-03 | 10 | 0 | Ovary4Species |
| GO:0030517 | negative regulation of axon extension | 1.22E-02 | 2.27E-03 | 10 | 0 | Ovary4Species |
| GO:0009052 | pentose-phosphate shunt, non-oxidative branch | 1.22E-02 | 2.27E-03 | 10 | 0 | Ovary4Species |
| GO:0042347 | negative regulation of NF-kappaB import into nucleus | 1.22E-02 | 2.27E-03 | 10 | 0 | Ovary4Species |
| GO:0016045 | detection of bacterium | 1.22E-02 | 2.27E-03 | 10 | 0 | Ovary4Species |
| GO:0008209 | androgen metabolic process | 1.22E-02 | 2.27E-03 | 10 | 0 | Ovary4Species |
| GO:0097111 | endoplasmic reticulum-Golgi intermediate compartment organization | 1.22E-02 | 2.27E-03 | 10 | 0 | Ovary4Species |
| GO:0061036 | positive regulation of cartilage development | 1.22E-02 | 2.27E-03 | 10 | 0 | Ovary4Species |
| GO:0003190 | atrioventricular valve formation | 1.22E-02 | 2.27E-03 | 10 | 0 | Ovary4Species |
| GO:0000959 | mitochondrial RNA metabolic process | 1.22E-02 | 2.27E-03 | 13 | 1 | Ovary4Species |
| GO:0035476 | angioblast cell migration | 1.54E-02 | 3.03E-03 | 15 | 2 | Ovary4Species |
| GO:0006749 | glutathione metabolic process | 1.65E-02 | 3.28E-03 | 20 | 4 | Ovary4Species |
| GO:0006099 | tricarboxylic acid cycle | 1.65E-02 | 3.28E-03 | 20 | 4 | Ovary4Species |
| GO:0060444 | branching involved in mammary gland duct morphogenesis | 1.67E-02 | 3.32E-03 | 18 | 3 | Ovary4Species |
| GO:0001836 | release of cytochrome c from mitochondria | 1.67E-02 | 3.32E-03 | 18 | 3 | Ovary4Species |
| GO:0003281 | ventricular septum development | 1.67E-02 | 3.32E-03 | 18 | 3 | Ovary4Species |
| GO:0008360 | regulation of cell shape | 1.85E-02 | 3.70E-03 | 77 | 39 | Ovary4Species |
| GO:0003140 | determination of left/right asymmetry in lateral mesoderm | 2.03E-02 | 4.10E-03 | 12 | 1 | Ovary4Species |
| GO:0045777 | positive regulation of blood pressure | 2.08E-02 | 4.21E-03 | 25 | 7 | Ovary4Species |
| GO:0060055 | angiogenesis involved in wound healing | 2.08E-02 | 4.21E-03 | 25 | 7 | Ovary4Species |
| GO:0033523 | histone H2B ubiquitination | 2.11E-02 | 4.41E-03 | 9 | 0 | Ovary4Species |
| GO:2001045 | negative regulation of integrin-mediated signaling pathway | 2.11E-02 | 4.41E-03 | 9 | 0 | Ovary4Species |
| GO:0002098 | tRNA wobble uridine modification | 2.11E-02 | 4.41E-03 | 9 | 0 | Ovary4Species |
| GO:0006435 | threonyl-tRNA aminoacylation | 2.11E-02 | 4.41E-03 | 9 | 0 | Ovary4Species |
| GO:0006069 | ethanol oxidation | 2.11E-02 | 4.41E-03 | 9 | 0 | Ovary4Species |
| GO:0050992 | dimethylallyl diphosphate biosynthetic process | 2.11E-02 | 4.41E-03 | 9 | 0 | Ovary4Species |
| GO:0048484 | enteric nervous system development | 2.11E-02 | 4.41E-03 | 9 | 0 | Ovary4Species |
| GO:0032049 | cardiolipin biosynthetic process | 2.11E-02 | 4.41E-03 | 9 | 0 | Ovary4Species |
| GO:0035912 | dorsal aorta morphogenesis | 2.11E-02 | 4.41E-03 | 9 | 0 | Ovary4Species |
| GO:0009396 | folic acid-containing compound biosynthetic process | 2.11E-02 | 4.41E-03 | 9 | 0 | Ovary4Species |
| GO:0035634 | response to stilbenoid | 2.11E-02 | 4.41E-03 | 9 | 0 | Ovary4Species |
| GO:0009264 | deoxyribonucleotide catabolic process | 2.11E-02 | 4.41E-03 | 9 | 0 | Ovary4Species |
| GO:0009113 | purine nucleobase biosynthetic process | 2.11E-02 | 4.41E-03 | 9 | 0 | Ovary4Species |
| GO:0015695 | organic cation transport | 2.11E-02 | 4.41E-03 | 9 | 0 | Ovary4Species |
| GO:0046655 | folic acid metabolic process | 2.11E-02 | 4.41E-03 | 9 | 0 | Ovary4Species |
| GO:0061309 | cardiac neural crest cell development involved in outflow tract morphogenesis | 2.11E-02 | 4.41E-03 | 9 | 0 | Ovary4Species |
| GO:0046456 | icosanoid biosynthetic process | 2.11E-02 | 4.41E-03 | 9 | 0 | Ovary4Species |
| GO:0080111 | DNA demethylation | 2.11E-02 | 4.41E-03 | 9 | 0 | Ovary4Species |
| GO:0034227 | tRNA thio-modification | 2.11E-02 | 4.41E-03 | 9 | 0 | Ovary4Species |
| GO:0016126 | sterol biosynthetic process | 2.19E-02 | 4.59E-03 | 34 | 12 | Ovary4Species |
| GO:0032495 | response to muramyl dipeptide | 2.20E-02 | 4.63E-03 | 23 | 6 | Ovary4Species |
| GO:0000768 | syncytium formation by plasma membrane fusion | 2.20E-02 | 4.63E-03 | 23 | 6 | Ovary4Species |
| GO:0008089 | anterograde axon cargo transport | 2.20E-02 | 4.63E-03 | 23 | 6 | Ovary4Species |
| GO:0051881 | regulation of mitochondrial membrane potential | 2.35E-02 | 5.20E-03 | 14 | 2 | Ovary4Species |
| GO:0016559 | peroxisome fission | 2.35E-02 | 5.20E-03 | 14 | 2 | Ovary4Species |
| GO:0061299 | retina vasculature morphogenesis in camera-type eye | 2.35E-02 | 5.20E-03 | 14 | 2 | Ovary4Species |

| | | | | | | |
|---|---|---|---|---|---|---|
| GO:0007588 | excretion | 2.35E-02 | 5.20E-03 | 14 | 2 | Ovary4Species |
| GO:0043149 | stress fiber assembly | 2.41E-02 | 5.37E-03 | 32 | 11 | Ovary4Species |
| GO:0006779 | porphyrin-containing compound biosynthetic process | 2.41E-02 | 5.37E-03 | 19 | 4 | Ovary4Species |
| GO:0033344 | cholesterol efflux | 2.41E-02 | 5.37E-03 | 19 | 4 | Ovary4Species |
| GO:0048384 | retinoic acid receptor signaling pathway | 2.41E-02 | 5.37E-03 | 19 | 4 | Ovary4Species |
| GO:0001702 | gastrulation with mouth forming second | 2.48E-02 | 5.54E-03 | 45 | 19 | Ovary4Species |
| GO:0032836 | glomerular basement membrane development | 2.50E-02 | 5.60E-03 | 17 | 3 | Ovary4Species |
| GO:0044743 | intracellular protein transmembrane import | 2.50E-02 | 5.60E-03 | 17 | 3 | Ovary4Species |
| GO:0060795 | cell fate commitment involved in formation of primary germ layer | 2.56E-02 | 5.76E-03 | 26 | 8 | Ovary4Species |
| GO:0031648 | protein destabilization | 3.21E-02 | 7.39E-03 | 11 | 1 | Ovary4Species |
| GO:0031577 | spindle checkpoint | 3.21E-02 | 7.39E-03 | 11 | 1 | Ovary4Species |
| GO:1900028 | negative regulation of ruffle assembly | 3.21E-02 | 7.39E-03 | 11 | 1 | Ovary4Species |
| GO:1900025 | negative regulation of substrate adhesion-dependent cell spreading | 3.21E-02 | 7.39E-03 | 11 | 1 | Ovary4Species |
| GO:0019985 | translesion synthesis | 3.21E-02 | 7.39E-03 | 11 | 1 | Ovary4Species |
| GO:0060528 | secretory columnal luminar epithelial cell differentiation involved in prostate glandular acinus development | 3.21E-02 | 7.39E-03 | 11 | 1 | Ovary4Species |
| GO:0060445 | branching involved in salivary gland morphogenesis | 3.57E-02 | 8.59E-03 | 8 | 0 | Ovary4Species |
| GO:0019368 | fatty acid elongation, unsaturated fatty acid | 3.57E-02 | 8.59E-03 | 8 | 0 | Ovary4Species |
| GO:0034063 | stress granule assembly | 3.57E-02 | 8.59E-03 | 8 | 0 | Ovary4Species |
| GO:0033561 | regulation of water loss via skin | 3.57E-02 | 8.59E-03 | 8 | 0 | Ovary4Species |
| GO:0032968 | positive regulation of transcription elongation from RNA polymerase II promoter | 3.57E-02 | 8.59E-03 | 8 | 0 | Ovary4Species |
| GO:0072104 | glomerular capillary formation | 3.57E-02 | 8.59E-03 | 8 | 0 | Ovary4Species |
| GO:0006625 | protein targeting to peroxisome | 3.57E-02 | 8.59E-03 | 8 | 0 | Ovary4Species |
| GO:1902306 | negative regulation of sodium ion transmembrane transport | 3.57E-02 | 8.59E-03 | 8 | 0 | Ovary4Species |
| GO:0032796 | uropod organization | 3.57E-02 | 8.59E-03 | 8 | 0 | Ovary4Species |
| GO:0071476 | cellular hypotonic response | 3.57E-02 | 8.59E-03 | 8 | 0 | Ovary4Species |
| GO:0006108 | malate metabolic process | 3.57E-02 | 8.59E-03 | 8 | 0 | Ovary4Species |
| GO:0090222 | centrosome-templated microtubule nucleation | 3.57E-02 | 8.59E-03 | 8 | 0 | Ovary4Species |
| GO:2000009 | negative regulation of protein localization to cell surface | 3.57E-02 | 8.59E-03 | 8 | 0 | Ovary4Species |
| GO:1901380 | negative regulation of potassium ion transmembrane transport | 3.57E-02 | 8.59E-03 | 8 | 0 | Ovary4Species |
| GO:1901017 | negative regulation of potassium ion transmembrane transporter activity | 3.57E-02 | 8.59E-03 | 8 | 0 | Ovary4Species |
| GO:0036302 | atrioventricular canal development | 3.57E-02 | 8.59E-03 | 8 | 0 | Ovary4Species |
| GO:0000212 | meiotic spindle organization | 3.57E-02 | 8.59E-03 | 8 | 0 | Ovary4Species |
| GO:0048147 | negative regulation of fibroblast proliferation | 3.57E-02 | 8.59E-03 | 8 | 0 | Ovary4Species |
| GO:0042753 | positive regulation of circadian rhythm | 3.57E-02 | 8.59E-03 | 8 | 0 | Ovary4Species |
| GO:0015911 | plasma membrane long-chain fatty acid transport | 3.57E-02 | 8.59E-03 | 8 | 0 | Ovary4Species |
| GO:0008611 | ether lipid biosynthetic process | 3.57E-02 | 8.59E-03 | 8 | 0 | Ovary4Species |
| GO:0015761 | mannose transport | 3.57E-02 | 8.59E-03 | 8 | 0 | Ovary4Species |
| GO:0010886 | positive regulation of cholesterol storage | 3.57E-02 | 8.59E-03 | 8 | 0 | Ovary4Species |
| GO:0061444 | endocardial cushion cell development | 3.57E-02 | 8.59E-03 | 8 | 0 | Ovary4Species |
| GO:0008356 | asymmetric cell division | 3.57E-02 | 8.59E-03 | 8 | 0 | Ovary4Species |
| GO:0010607 | negative regulation of cytoplasmic mRNA processing body assembly | 3.57E-02 | 8.59E-03 | 8 | 0 | Ovary4Species |
| GO:0008295 | spermidine biosynthetic process | 3.57E-02 | 8.59E-03 | 8 | 0 | Ovary4Species |
| GO:0002943 | tRNA dihydrouridine synthesis | 3.57E-02 | 8.59E-03 | 8 | 0 | Ovary4Species |
| GO:0002902 | regulation of B cell apoptotic process | 3.57E-02 | 8.59E-03 | 8 | 0 | Ovary4Species |
| GO:0061156 | pulmonary artery morphogenesis | 3.57E-02 | 8.59E-03 | 8 | 0 | Ovary4Species |
| GO:0060638 | mesenchymal-epithelial cell signaling | 3.57E-02 | 8.59E-03 | 8 | 0 | Ovary4Species |
| GO:0061029 | eyelid development in camera-type eye | 3.57E-02 | 8.59E-03 | 8 | 0 | Ovary4Species |
| GO:0072554 | blood vessel lumenization | 3.57E-02 | 8.59E-03 | 8 | 0 | Ovary4Species |
| GO:0032434 | regulation of proteasomal ubiquitin-dependent protein catabolic process | 3.62E-02 | 8.74E-03 | 18 | 4 | Ovary4Species |
| GO:0090263 | positive regulation of canonical Wnt receptor signaling pathway | 3.62E-02 | 8.74E-03 | 18 | 4 | Ovary4Species |
| GO:0045743 | positive regulation of fibroblast growth factor receptor signaling pathway | 3.66E-02 | 8.88E-03 | 13 | 2 | Ovary4Species |
| GO:0051295 | establishment of meiotic spindle localization | 3.66E-02 | 8.88E-03 | 13 | 2 | Ovary4Species |
| GO:0043462 | regulation of ATPase activity | 3.66E-02 | 8.88E-03 | 13 | 2 | Ovary4Species |
| GO:0035118 | embryonic pectoral fin morphogenesis | 3.66E-02 | 8.88E-03 | 13 | 2 | Ovary4Species |
| GO:0097345 | mitochondrial outer membrane permeabilization | 3.66E-02 | 8.88E-03 | 13 | 2 | Ovary4Species |
| GO:0003272 | endocardial cushion formation | 3.66E-02 | 8.88E-03 | 13 | 2 | Ovary4Species |
| GO:0007004 | telomere maintenance via telomerase | 4.00E-02 | 9.83E-03 | 23 | 7 | Ovary4Species |
| GO:0008299 | isoprenoid biosynthetic process | 4.00E-02 | 9.83E-03 | 23 | 7 | Ovary4Species |
| GO:0002011 | morphogenesis of an epithelial sheet | 4.06E-02 | 1.00E-02 | 47 | 21 | Ovary4Species |
| GO:0090307 | spindle assembly involved in mitosis | 4.34E-02 | 1.11E-02 | 27 | 9 | Ovary4Species |
| GO:0010977 | negative regulation of neuron projection development | 4.45E-02 | 1.14E-02 | 30 | 11 | Ovary4Species |
| GO:0046839 | phospholipid dephosphorylation | 4.48E-02 | 1.15E-02 | 36 | 15 | Ovary4Species |
| GO:0030913 | paranodal junction assembly | 4.88E-02 | 1.26E-02 | 19 | 5 | Ovary4Species |

**Enriched in Testis in all four species**

| | | | | | | |
|---|---|---|---|---|---|---|
| GO:0035083 | cilium axoneme assembly | 4.07E-13 | 4.43E-15 | 5 | 61 | Testis4Species |
| GO:0045494 | photoreceptor cell maintenance | 4.07E-13 | 4.46E-15 | 2 | 52 | Testis4Species |
| GO:0060729 | intestinal epithelial structure maintenance | 8.04E-12 | 1.24E-13 | 3 | 51 | Testis4Species |
| GO:0071879 | positive regulation of adrenergic receptor signaling pathway | 9.68E-12 | 1.52E-13 | 0 | 39 | Testis4Species |
| GO:0007286 | spermatid development | 1.13E-11 | 1.79E-13 | 24 | 95 | Testis4Species |
| GO:0044333 | Wnt receptor signaling pathway involved in digestive tract morphogenesis | 1.91E-11 | 3.24E-13 | 0 | 38 | Testis4Species |
| GO:0006977 | DNA damage response, signal transduction by p53 class mediator resulting in cell cycle arrest | 3.81E-11 | 6.92E-13 | 0 | 37 | Testis4Species |
| GO:0090327 | negative regulation of locomotion involved in locomotory behavior | 7.43E-11 | 1.48E-12 | 0 | 36 | Testis4Species |
| GO:0044252 | negative regulation of multicellular organismal metabolic process | 7.43E-11 | 1.48E-12 | 0 | 36 | Testis4Species |
| GO:0031651 | negative regulation of heat generation | 7.43E-11 | 1.48E-12 | 0 | 36 | Testis4Species |
| GO:0046827 | positive regulation of protein export from nucleus | 1.47E-10 | 3.15E-12 | 0 | 35 | Testis4Species |
| GO:0043570 | maintenance of DNA repeat elements | 2.90E-10 | 6.73E-12 | 0 | 34 | Testis4Species |
| GO:0048625 | myoblast fate commitment | 5.78E-10 | 1.44E-11 | 0 | 33 | Testis4Species |
| GO:0060613 | fat pad development | 1.12E-09 | 2.97E-11 | 1 | 36 | Testis4Species |
| GO:0040018 | positive regulation of multicellular organism growth | 1.15E-09 | 3.06E-11 | 0 | 32 | Testis4Species |
| GO:0033119 | negative regulation of RNA splicing | 2.12E-09 | 6.18E-11 | 1 | 35 | Testis4Species |
| GO:0000122 | negative regulation of transcription from RNA polymerase II promoter | 2.15E-09 | 6.45E-11 | 130 | 231 | Testis4Species |
| GO:0072369 | regulation of lipid transport by positive regulation of transcription from RNA polymerase II promoter | 2.15E-09 | 6.54E-11 | 0 | 31 | Testis4Species |
| GO:0044334 | canonical Wnt receptor signaling pathway involved in positive regulation of epithelial to mesenchymal transition | 2.15E-09 | 6.54E-11 | 0 | 31 | Testis4Species |
| GO:0010909 | positive regulation of heparan sulfate proteoglycan biosynthetic process | 2.15E-09 | 6.54E-11 | 0 | 31 | Testis4Species |
| GO:0006376 | mRNA splice site selection | 2.74E-09 | 8.36E-11 | 2 | 38 | Testis4Species |
| GO:0030538 | embryonic genitalia morphogenesis | 4.33E-09 | 1.39E-10 | 0 | 30 | Testis4Species |
| GO:0046621 | negative regulation of organ growth | 4.33E-09 | 1.39E-10 | 0 | 30 | Testis4Species |
| GO:0009791 | post-embryonic development | 5.26E-09 | 1.72E-10 | 31 | 94 | Testis4Species |
| GO:0000381 | regulation of alternative mRNA splicing, via spliceosome | 1.60E-08 | 5.52E-10 | 1 | 32 | Testis4Species |
| GO:0035721 | intraflagellar retrograde transport | 1.84E-08 | 6.35E-10 | 0 | 28 | Testis4Species |
| GO:1902165 | regulation of intrinsic apoptotic signaling pathway in response to DNA damage by p53 class mediator | 3.21E-08 | 1.14E-09 | 1 | 31 | Testis4Species |
| GO:0051877 | pigment granule aggregation in cell center | 3.69E-08 | 1.35E-09 | 0 | 27 | Testis4Species |
| GO:0032252 | secretory granule localization | 3.69E-08 | 1.35E-09 | 0 | 27 | Testis4Species |
| GO:0070121 | Kupffer's vesicle development | 6.21E-08 | 2.35E-09 | 13 | 58 | Testis4Species |
| GO:0007252 | I-kappaB phosphorylation | 6.21E-08 | 2.36E-09 | 1 | 30 | Testis4Species |
| GO:0051965 | positive regulation of synapse assembly | 1.51E-07 | 6.16E-09 | 0 | 25 | Testis4Species |
| GO:0021513 | spinal cord dorsal/ventral patterning | 2.39E-07 | 1.01E-08 | 1 | 28 | Testis4Species |
| GO:0045716 | positive regulation of low-density lipoprotein particle receptor biosynthetic process | 2.60E-07 | 1.11E-08 | 2 | 31 | Testis4Species |
| GO:0060296 | regulation of cilium beat frequency involved in ciliary motility | 3.02E-07 | 1.31E-08 | 0 | 24 | Testis4Species |
| GO:0032350 | regulation of hormone metabolic process | 3.40E-07 | 1.49E-08 | 5 | 39 | Testis4Species |
| GO:0048286 | lung alveolus development | 3.83E-07 | 1.70E-08 | 9 | 47 | Testis4Species |
| GO:0048619 | embryonic hindgut morphogenesis | 4.83E-07 | 2.21E-08 | 2 | 30 | Testis4Species |
| GO:0019886 | antigen processing and presentation of exogenous peptide antigen via MHC class II | 1.20E-06 | 5.94E-08 | 13 | 52 | Testis4Species |
| GO:0048557 | embryonic digestive tract morphogenesis | 1.26E-06 | 6.28E-08 | 4 | 34 | Testis4Species |

| | | | | | | |
|---|---|---|---|---|---|---|
| GO:2000675 | negative regulation of type B pancreatic cell apoptotic process | 1.50E-06 | 7.65E-08 | 3 | 31 | Testis4Species |
| GO:0007205 | protein kinase C-activating G-protein coupled receptor signaling pathway | 1.70E-06 | 8.80E-08 | 2 | 28 | Testis4Species |
| GO:0042759 | long-chain fatty acid biosynthetic process | 1.70E-06 | 8.80E-08 | 2 | 28 | Testis4Species |
| GO:0038108 | negative regulation of appetite by leptin-mediated signaling pathway | 2.37E-06 | 1.27E-07 | 0 | 21 | Testis4Species |
| GO:0048208 | COPII vesicle coating | 2.37E-06 | 1.27E-07 | 0 | 21 | Testis4Species |
| GO:0060831 | smoothened signaling pathway involved in dorsal/ventral neural tube patterning | 2.37E-06 | 1.27E-07 | 0 | 21 | Testis4Species |
| GO:0032024 | positive regulation of insulin secretion | 2.52E-06 | 1.37E-07 | 7 | 39 | Testis4Species |
| GO:0061178 | regulation of insulin secretion involved in cellular response to glucose stimulus | 4.35E-06 | 2.47E-07 | 7 | 38 | Testis4Species |
| GO:0048854 | brain morphogenesis | 8.54E-06 | 5.16E-07 | 11 | 45 | Testis4Species |
| GO:0030241 | skeletal muscle myosin thick filament assembly | 9.47E-06 | 5.78E-07 | 0 | 19 | Testis4Species |
| GO:0006114 | glycerol biosynthetic process | 1.83E-05 | 1.23E-06 | 0 | 18 | Testis4Species |
| GO:0060830 | ciliary receptor clustering involved in smoothened signaling pathway | 1.83E-05 | 1.23E-06 | 0 | 18 | Testis4Species |
| GO:0001938 | positive regulation of endothelial cell proliferation | 1.87E-05 | 1.26E-06 | 6 | 34 | Testis4Species |
| GO:0007601 | visual perception | 2.07E-05 | 1.42E-06 | 89 | 149 | Testis4Species |
| GO:0046330 | positive regulation of JNK cascade | 2.10E-05 | 1.44E-06 | 4 | 29 | Testis4Species |
| GO:0035058 | nonmotile primary cilium assembly | 2.83E-05 | 2.01E-06 | 3 | 26 | Testis4Species |
| GO:0048793 | pronephros development | 2.84E-05 | 2.01E-06 | 14 | 47 | Testis4Species |
| GO:0051154 | negative regulation of striated muscle cell differentiation | 3.61E-05 | 2.63E-06 | 0 | 17 | Testis4Species |
| GO:0048713 | regulation of oligodendrocyte differentiation | 3.63E-05 | 2.68E-06 | 4 | 28 | Testis4Species |
| GO:0021756 | striatum development | 3.63E-05 | 2.68E-06 | 2 | 23 | Testis4Species |
| GO:0001958 | endochondral ossification | 3.63E-05 | 2.68E-06 | 2 | 23 | Testis4Species |
| GO:0046627 | negative regulation of insulin receptor signaling pathway | 5.47E-05 | 4.19E-06 | 10 | 39 | Testis4Species |
| GO:0070098 | chemokine-mediated signaling pathway | 6.09E-05 | 4.75E-06 | 1 | 20 | Testis4Species |
| GO:0006378 | mRNA polyadenylation | 6.28E-05 | 4.95E-06 | 4 | 27 | Testis4Species |
| GO:0050798 | activated T cell proliferation | 6.61E-05 | 5.25E-06 | 2 | 22 | Testis4Species |
| GO:0021547 | midbrain-hindbrain boundary initiation | 6.95E-05 | 5.60E-06 | 0 | 16 | Testis4Species |
| GO:0010839 | negative regulation of keratinocyte proliferation | 6.95E-05 | 5.60E-06 | 0 | 16 | Testis4Species |
| GO:2000177 | regulation of neural precursor cell proliferation | 7.51E-05 | 6.12E-06 | 13 | 43 | Testis4Species |
| GO:0042147 | retrograde transport, endosome to Golgi | 7.51E-05 | 6.12E-06 | 13 | 43 | Testis4Species |
| GO:0032402 | melanosome transport | 8.38E-05 | 6.94E-06 | 6 | 31 | Testis4Species |
| GO:0017156 | calcium ion-dependent exocytosis | 8.38E-05 | 6.94E-06 | 6 | 31 | Testis4Species |
| GO:0040037 | negative regulation of fibroblast growth factor receptor signaling pathway | 8.84E-05 | 7.37E-06 | 9 | 36 | Testis4Species |
| GO:0000132 | establishment of mitotic spindle orientation | 1.08E-04 | 9.14E-06 | 4 | 26 | Testis4Species |
| GO:0000724 | double-strand break repair via homologous recombination | 1.24E-04 | 1.08E-05 | 5 | 28 | Testis4Species |
| GO:0080009 | mRNA methylation | 1.35E-04 | 1.19E-05 | 0 | 15 | Testis4Species |
| GO:0006002 | fructose 6-phosphate metabolic process | 1.35E-04 | 1.19E-05 | 0 | 15 | Testis4Species |
| GO:0036265 | RNA (guanine-N7)-methylation | 1.35E-04 | 1.19E-05 | 0 | 15 | Testis4Species |
| GO:0007202 | activation of phospholipase C activity | 1.37E-04 | 1.22E-05 | 6 | 30 | Testis4Species |
| GO:0048660 | regulation of smooth muscle cell proliferation | 1.45E-04 | 1.30E-05 | 18 | 50 | Testis4Species |
| GO:0007029 | endoplasmic reticulum organization | 1.53E-04 | 1.38E-05 | 3 | 23 | Testis4Species |
| GO:0007140 | male meiosis | 2.03E-04 | 1.87E-05 | 1 | 18 | Testis4Species |
| GO:0010881 | regulation of cardiac muscle contraction by regulation of the release of sequestered calcium ion | 2.03E-04 | 1.87E-05 | 1 | 18 | Testis4Species |
| GO:0021984 | adenohypophysis development | 2.03E-04 | 1.87E-05 | 1 | 18 | Testis4Species |
| GO:0048708 | astrocyte differentiation | 2.08E-04 | 1.93E-05 | 5 | 27 | Testis4Species |
| GO:0019233 | sensory perception of pain | 2.12E-04 | 1.98E-05 | 10 | 36 | Testis4Species |
| GO:0010988 | regulation of low-density lipoprotein particle clearance | 2.42E-04 | 2.28E-05 | 7 | 31 | Testis4Species |
| GO:0072019 | proximal convoluted tubule development | 2.63E-04 | 2.54E-05 | 0 | 14 | Testis4Species |
| GO:0006533 | aspartate catabolic process | 2.63E-04 | 2.54E-05 | 0 | 14 | Testis4Species |
| GO:0048739 | cardiac muscle fiber development | 2.63E-04 | 2.54E-05 | 0 | 14 | Testis4Species |
| GO:0018094 | protein polyglycylation | 2.63E-04 | 2.54E-05 | 0 | 14 | Testis4Species |
| GO:0031585 | regulation of inositol 1,4,5-trisphosphate-sensitive calcium-release channel activity | 2.63E-04 | 2.54E-05 | 0 | 14 | Testis4Species |
| GO:0031116 | positive regulation of microtubule polymerization | 2.63E-04 | 2.54E-05 | 0 | 14 | Testis4Species |
| GO:0042517 | positive regulation of tyrosine phosphorylation of Stat3 protein | 2.63E-04 | 2.54E-05 | 0 | 14 | Testis4Species |
| GO:0090090 | negative regulation of canonical Wnt receptor signaling pathway | 2.82E-04 | 2.73E-05 | 38 | 76 | Testis4Species |
| GO:0048015 | phosphatidylinositol-mediated signaling | 3.25E-04 | 3.20E-05 | 39 | 77 | Testis4Species |
| GO:0048488 | synaptic vesicle endocytosis | 3.48E-04 | 3.44E-05 | 5 | 26 | Testis4Species |
| GO:0045879 | negative regulation of smoothened signaling pathway | 3.48E-04 | 3.44E-05 | 5 | 26 | Testis4Species |
| GO:0006562 | proline catabolic process | 5.14E-04 | 5.41E-05 | 0 | 13 | Testis4Species |
| GO:0070842 | aggresome assembly | 5.14E-04 | 5.41E-05 | 0 | 13 | Testis4Species |
| GO:0032237 | activation of store-operated calcium channel activity | 5.14E-04 | 5.41E-05 | 0 | 13 | Testis4Species |
| GO:0050893 | sensory processing | 5.14E-04 | 5.41E-05 | 0 | 13 | Testis4Species |
| GO:0035845 | photoreceptor cell outer segment organization | 5.14E-04 | 5.41E-05 | 0 | 13 | Testis4Species |
| GO:0043388 | positive regulation of DNA binding | 5.14E-04 | 5.41E-05 | 0 | 13 | Testis4Species |
| GO:0035641 | locomotory exploration behavior | 5.14E-04 | 5.41E-05 | 0 | 13 | Testis4Species |
| GO:0032481 | positive regulation of type I interferon production | 5.15E-04 | 5.44E-05 | 10 | 34 | Testis4Species |
| GO:0030819 | positive regulation of cAMP biosynthetic process | 5.29E-04 | 5.61E-05 | 4 | 23 | Testis4Species |
| GO:0000160 | phosphorelay signal transduction system | 5.29E-04 | 5.61E-05 | 4 | 23 | Testis4Species |
| GO:0045727 | positive regulation of translation | 5.73E-04 | 6.11E-05 | 5 | 25 | Testis4Species |
| GO:0043001 | Golgi to plasma membrane protein transport | 5.98E-04 | 6.42E-05 | 6 | 27 | Testis4Species |
| GO:0050879 | multicellular organismal movement | 6.11E-04 | 6.59E-05 | 7 | 29 | Testis4Species |
| GO:0030514 | negative regulation of BMP signaling pathway | 6.62E-04 | 7.17E-05 | 12 | 37 | Testis4Species |
| GO:0002437 | inflammatory response to antigenic stimulus | 6.71E-04 | 7.35E-05 | 1 | 16 | Testis4Species |
| GO:0030213 | hyaluronan biosynthetic process | 6.71E-04 | 7.35E-05 | 1 | 16 | Testis4Species |
| GO:0006334 | nucleosome assembly | 8.01E-04 | 8.95E-05 | 10 | 33 | Testis4Species |
| GO:0031016 | pancreas development | 8.02E-04 | 8.97E-05 | 26 | 57 | Testis4Species |
| GO:0032092 | positive regulation of protein binding | 8.09E-04 | 9.06E-05 | 14 | 40 | Testis4Species |
| GO:0045930 | negative regulation of mitotic cell cycle | 8.13E-04 | 9.14E-05 | 3 | 20 | Testis4Species |
| GO:0032963 | collagen metabolic process | 8.92E-04 | 1.02E-04 | 4 | 22 | Testis4Species |
| GO:0035176 | social behavior | 8.92E-04 | 1.02E-04 | 4 | 22 | Testis4Species |
| GO:0032855 | positive regulation of Rac GTPase activity | 9.38E-04 | 1.08E-04 | 5 | 24 | Testis4Species |
| GO:0006369 | termination of RNA polymerase II transcription | 9.63E-04 | 1.11E-04 | 7 | 28 | Testis4Species |
| GO:0032006 | regulation of TOR signaling cascade | 9.75E-04 | 1.15E-04 | 12 | 36 | Testis4Species |
| GO:0060426 | lung vasculature development | 9.75E-04 | 1.15E-04 | 0 | 12 | Testis4Species |
| GO:0033235 | positive regulation of protein sumoylation | 9.75E-04 | 1.15E-04 | 0 | 12 | Testis4Species |
| GO:2000463 | positive regulation of excitatory postsynaptic membrane potential | 9.75E-04 | 1.15E-04 | 0 | 12 | Testis4Species |
| GO:2000310 | regulation of N-methyl-D-aspartate selective glutamate receptor activity | 9.75E-04 | 1.15E-04 | 0 | 12 | Testis4Species |
| GO:0050872 | white fat cell differentiation | 9.75E-04 | 1.15E-04 | 0 | 12 | Testis4Species |
| GO:0042416 | dopamine biosynthetic process | 9.75E-04 | 1.15E-04 | 0 | 12 | Testis4Species |
| GO:0042264 | peptidyl-aspartic acid hydroxylation | 9.75E-04 | 1.15E-04 | 0 | 12 | Testis4Species |
| GO:0060004 | reflex | 1.20E-03 | 1.45E-04 | 1 | 15 | Testis4Species |
| GO:0007159 | leukocyte cell-cell adhesion | 1.37E-03 | 1.70E-04 | 3 | 19 | Testis4Species |
| GO:0021707 | cerebellar granule cell differentiation | 1.47E-03 | 1.84E-04 | 4 | 21 | Testis4Species |
| GO:0060028 | convergent extension involved in axis elongation | 1.51E-03 | 1.90E-04 | 5 | 23 | Testis4Species |
| GO:0000722 | telomere maintenance via recombination | 1.88E-03 | 2.42E-04 | 2 | 17 | Testis4Species |
| GO:0000050 | urea cycle | 1.88E-03 | 2.42E-04 | 2 | 17 | Testis4Species |
| GO:0045722 | positive regulation of gluconeogenesis | 1.88E-03 | 2.46E-04 | 0 | 11 | Testis4Species |
| GO:2000821 | regulation of grooming behavior | 1.88E-03 | 2.46E-04 | 0 | 11 | Testis4Species |
| GO:0071625 | vocalization behavior | 1.88E-03 | 2.46E-04 | 0 | 11 | Testis4Species |
| GO:0045071 | negative regulation of viral genome replication | 1.88E-03 | 2.46E-04 | 0 | 11 | Testis4Species |
| GO:0090292 | nuclear matrix anchoring at nuclear membrane | 1.88E-03 | 2.46E-04 | 0 | 11 | Testis4Species |
| GO:0048791 | calcium ion-dependent exocytosis of neurotransmitter | 1.88E-03 | 2.46E-04 | 0 | 11 | Testis4Species |
| GO:0035695 | mitochondrion degradation by induced vacuole formation | 1.88E-03 | 2.46E-04 | 0 | 11 | Testis4Species |
| GO:0035694 | mitochondrial protein catabolic process | 1.88E-03 | 2.46E-04 | 0 | 11 | Testis4Species |
| GO:0036088 | D-serine catabolic process | 1.88E-03 | 2.46E-04 | 0 | 11 | Testis4Species |
| GO:0055130 | D-alanine catabolic process | 1.88E-03 | 2.46E-04 | 0 | 11 | Testis4Species |

| | | | | | | |
|---|---|---|---|---|---|---|
| GO:0035022 | positive regulation of Rac protein signal transduction | 1.88E-03 | 2.46E-04 | 0 | 11 | Testis4Species |
| GO:0042026 | protein refolding | 1.88E-03 | 2.46E-04 | 0 | 11 | Testis4Species |
| GO:0097113 | alpha-amino-3-hydroxy-5-methyl-4-isoxazole propionate receptor clustering | 1.88E-03 | 2.46E-04 | 0 | 11 | Testis4Species |
| GO:0072661 | protein targeting to plasma membrane | 1.88E-03 | 2.46E-04 | 0 | 11 | Testis4Species |
| GO:0085032 | modulation by symbiont of host I-kappaB kinase/NF-kappaB cascade | 1.88E-03 | 2.46E-04 | 0 | 11 | Testis4Species |
| GO:0021766 | hippocampus development | 1.92E-03 | 2.51E-04 | 13 | 36 | Testis4Species |
| GO:0031290 | retinal ganglion cell axon guidance | 1.92E-03 | 2.51E-04 | 13 | 36 | Testis4Species |
| GO:0003146 | heart jogging | 2.06E-03 | 2.71E-04 | 20 | 46 | Testis4Species |
| GO:0060021 | palate development | 2.08E-03 | 2.74E-04 | 26 | 54 | Testis4Species |
| GO:0060213 | positive regulation of nuclear-transcribed mRNA poly(A) tail shortening | 2.15E-03 | 2.87E-04 | 1 | 14 | Testis4Species |
| GO:0032516 | positive regulation of phosphoprotein phosphatase activity | 2.15E-03 | 2.87E-04 | 1 | 14 | Testis4Species |
| GO:0048026 | positive regulation of mRNA splicing, via spliceosome | 2.15E-03 | 2.87E-04 | 1 | 14 | Testis4Species |
| GO:0045843 | negative regulation of striated muscle tissue development | 2.15E-03 | 2.87E-04 | 1 | 14 | Testis4Species |
| GO:0014003 | oligodendrocyte development | 2.16E-03 | 2.88E-04 | 12 | 34 | Testis4Species |
| GO:0048011 | neurotrophin TRK receptor signaling pathway | 2.30E-03 | 3.09E-04 | 83 | 122 | Testis4Species |
| GO:0060291 | long-term synaptic potentiation | 2.30E-03 | 3.14E-04 | 3 | 18 | Testis4Species |
| GO:0042755 | eating behavior | 2.30E-03 | 3.14E-04 | 3 | 18 | Testis4Species |
| GO:0008105 | asymmetric protein localization | 2.40E-03 | 3.30E-04 | 4 | 20 | Testis4Species |
| GO:0045880 | positive regulation of smoothened signaling pathway | 2.40E-03 | 3.30E-04 | 4 | 20 | Testis4Species |
| GO:0035019 | somatic stem cell maintenance | 2.80E-03 | 3.90E-04 | 13 | 35 | Testis4Species |
| GO:0031641 | regulation of myelination | 3.21E-03 | 4.53E-04 | 12 | 33 | Testis4Species |
| GO:0007413 | axonal fasciculation | 3.58E-03 | 5.23E-04 | 0 | 10 | Testis4Species |
| GO:0060386 | synapse assembly involved in innervation | 3.58E-03 | 5.23E-04 | 0 | 10 | Testis4Species |
| GO:0002329 | pre-B cell differentiation | 3.58E-03 | 5.23E-04 | 0 | 10 | Testis4Species |
| GO:0006532 | aspartate biosynthetic process | 3.58E-03 | 5.23E-04 | 0 | 10 | Testis4Species |
| GO:0045299 | otolith mineralization | 3.58E-03 | 5.23E-04 | 0 | 10 | Testis4Species |
| GO:0033194 | response to hydroperoxide | 3.58E-03 | 5.23E-04 | 0 | 10 | Testis4Species |
| GO:0006388 | tRNA splicing, via endonucleolytic cleavage and ligation | 3.58E-03 | 5.23E-04 | 0 | 10 | Testis4Species |
| GO:0035995 | detection of muscle stretch | 3.58E-03 | 5.23E-04 | 0 | 10 | Testis4Species |
| GO:0031581 | hemidesmosome assembly | 3.58E-03 | 5.23E-04 | 0 | 10 | Testis4Species |
| GO:0043537 | negative regulation of blood vessel endothelial cell migration | 3.58E-03 | 5.23E-04 | 0 | 10 | Testis4Species |
| GO:0048265 | response to pain | 3.58E-03 | 5.23E-04 | 0 | 10 | Testis4Species |
| GO:0048251 | elastic fiber assembly | 3.58E-03 | 5.23E-04 | 0 | 10 | Testis4Species |
| GO:0070073 | clustering of voltage-gated calcium channels | 3.58E-03 | 5.23E-04 | 0 | 10 | Testis4Species |
| GO:0042518 | negative regulation of tyrosine phosphorylation of Stat3 protein | 3.58E-03 | 5.23E-04 | 0 | 10 | Testis4Species |
| GO:0035278 | negative regulation of translation involved in gene silencing by miRNA | 3.58E-03 | 5.23E-04 | 0 | 10 | Testis4Species |
| GO:0010760 | negative regulation of macrophage chemotaxis | 3.58E-03 | 5.23E-04 | 0 | 10 | Testis4Species |
| GO:0014824 | artery smooth muscle contraction | 3.58E-03 | 5.23E-04 | 0 | 10 | Testis4Species |
| GO:0019551 | glutamate catabolic process to 2-oxoglutarate | 3.58E-03 | 5.23E-04 | 0 | 10 | Testis4Species |
| GO:0019550 | glutamate catabolic process to aspartate | 3.58E-03 | 5.23E-04 | 0 | 10 | Testis4Species |
| GO:0051966 | regulation of synaptic transmission, glutamatergic | 3.83E-03 | 5.65E-04 | 1 | 13 | Testis4Species |
| GO:0043567 | regulation of insulin-like growth factor receptor signaling pathway | 3.83E-03 | 5.65E-04 | 1 | 13 | Testis4Species |
| GO:0002026 | regulation of the force of heart contraction | 3.88E-03 | 5.77E-04 | 5 | 21 | Testis4Species |
| GO:0014850 | response to muscle activity | 3.88E-03 | 5.77E-04 | 3 | 17 | Testis4Species |
| GO:0007129 | synapsis | 3.92E-03 | 5.88E-04 | 4 | 19 | Testis4Species |
| GO:0032570 | response to progesterone stimulus | 3.92E-03 | 5.88E-04 | 4 | 19 | Testis4Species |
| GO:0055089 | fatty acid homeostasis | 3.92E-03 | 5.88E-04 | 4 | 19 | Testis4Species |
| GO:0023014 | signal transduction by phosphorylation | 3.93E-03 | 5.91E-04 | 20 | 44 | Testis4Species |
| GO:0032355 | response to estradiol stimulus | 3.97E-03 | 6.04E-04 | 13 | 34 | Testis4Species |
| GO:0048169 | regulation of long-term neuronal synaptic plasticity | 4.06E-03 | 6.19E-04 | 10 | 29 | Testis4Species |
| GO:0006338 | chromatin remodeling | 4.16E-03 | 6.36E-04 | 29 | 56 | Testis4Species |
| GO:0043200 | response to amino acid stimulus | 5.32E-03 | 8.35E-04 | 11 | 30 | Testis4Species |
| GO:0051865 | protein autoubiquitination | 5.46E-03 | 8.63E-04 | 7 | 24 | Testis4Species |
| GO:0071482 | cellular response to light stimulus | 5.46E-03 | 8.63E-04 | 18 | 40 | Testis4Species |
| GO:0003351 | epithelial cilium movement | 5.87E-03 | 9.30E-04 | 13 | 33 | Testis4Species |
| GO:0045822 | negative regulation of heart contraction | 5.87E-03 | 9.32E-04 | 6 | 22 | Testis4Species |
| GO:0010649 | regulation of cell communication by electrical coupling | 6.48E-03 | 1.04E-03 | 4 | 18 | Testis4Species |
| GO:0000184 | nuclear-transcribed mRNA catabolic process, nonsense-mediated decay | 6.64E-03 | 1.10E-03 | 12 | 31 | Testis4Species |
| GO:0005513 | detection of calcium ion | 6.64E-03 | 1.11E-03 | 1 | 12 | Testis4Species |
| GO:0060785 | regulation of apoptosis involved in tissue homeostasis | 6.64E-03 | 1.11E-03 | 1 | 12 | Testis4Species |
| GO:0002639 | positive regulation of immunoglobulin production | 6.64E-03 | 1.11E-03 | 0 | 9 | Testis4Species |
| GO:0045724 | positive regulation of cilium assembly | 6.64E-03 | 1.11E-03 | 0 | 9 | Testis4Species |
| GO:0021744 | dorsal motor nucleus of vagus nerve development | 6.64E-03 | 1.11E-03 | 0 | 9 | Testis4Species |
| GO:0046154 | rhodopsin metabolic process | 6.64E-03 | 1.11E-03 | 0 | 9 | Testis4Species |
| GO:0060315 | negative regulation of ryanodine-sensitive calcium-release channel activity | 6.64E-03 | 1.11E-03 | 0 | 9 | Testis4Species |
| GO:0021592 | fourth ventricle development | 6.64E-03 | 1.11E-03 | 0 | 9 | Testis4Species |
| GO:2000727 | positive regulation of cardiac muscle cell differentiation | 6.64E-03 | 1.11E-03 | 0 | 9 | Testis4Species |
| GO:0006551 | leucine metabolic process | 6.64E-03 | 1.11E-03 | 0 | 9 | Testis4Species |
| GO:0002091 | negative regulation of receptor internalization | 6.64E-03 | 1.11E-03 | 0 | 9 | Testis4Species |
| GO:0070846 | Hsp90 deacetylation | 6.64E-03 | 1.11E-03 | 0 | 9 | Testis4Species |
| GO:1902083 | negative regulation of peptidyl-cysteine S-nitrosylation | 6.64E-03 | 1.11E-03 | 0 | 9 | Testis4Species |
| GO:0032515 | negative regulation of phosphoprotein phosphatase activity | 6.64E-03 | 1.11E-03 | 0 | 9 | Testis4Species |
| GO:0071218 | cellular response to misfolded protein | 6.64E-03 | 1.11E-03 | 0 | 9 | Testis4Species |
| GO:0090314 | positive regulation of protein targeting to membrane | 6.64E-03 | 1.11E-03 | 0 | 9 | Testis4Species |
| GO:0090245 | axis elongation involved in somitogenesis | 6.64E-03 | 1.11E-03 | 0 | 9 | Testis4Species |
| GO:0032314 | regulation of Rac GTPase activity | 6.64E-03 | 1.11E-03 | 0 | 9 | Testis4Species |
| GO:0090129 | positive regulation of synapse maturation | 6.64E-03 | 1.11E-03 | 0 | 9 | Testis4Species |
| GO:1901250 | negative regulation of lung goblet cell differentiation | 6.64E-03 | 1.11E-03 | 0 | 9 | Testis4Species |
| GO:0090074 | negative regulation of protein homodimerization activity | 6.64E-03 | 1.11E-03 | 0 | 9 | Testis4Species |
| GO:0090042 | tubulin deacetylation | 6.64E-03 | 1.11E-03 | 0 | 9 | Testis4Species |
| GO:1900748 | positive regulation of vascular endothelial growth factor signaling pathway | 6.64E-03 | 1.11E-03 | 0 | 9 | Testis4Species |
| GO:0043252 | sodium-independent organic anion transport | 6.64E-03 | 1.11E-03 | 0 | 9 | Testis4Species |
| GO:0031122 | cytoplasmic microtubule organization | 6.64E-03 | 1.11E-03 | 0 | 9 | Testis4Species |
| GO:0016332 | establishment or maintenance of polarity of embryonic epithelium | 6.64E-03 | 1.11E-03 | 0 | 9 | Testis4Species |
| GO:0031061 | negative regulation of histone methylation | 6.64E-03 | 1.11E-03 | 0 | 9 | Testis4Species |
| GO:0042527 | negative regulation of tyrosine phosphorylation of Stat6 protein | 6.64E-03 | 1.11E-03 | 0 | 9 | Testis4Species |
| GO:0042524 | negative regulation of tyrosine phosphorylation of Stat5 protein | 6.64E-03 | 1.11E-03 | 0 | 9 | Testis4Species |
| GO:0015732 | prostaglandin transport | 6.64E-03 | 1.11E-03 | 0 | 9 | Testis4Species |
| GO:0034587 | piRNA metabolic process | 6.64E-03 | 1.11E-03 | 0 | 9 | Testis4Species |
| GO:0061140 | lung secretory cell differentiation | 6.64E-03 | 1.11E-03 | 0 | 9 | Testis4Species |
| GO:0043407 | negative regulation of MAP kinase activity | 6.79E-03 | 1.15E-03 | 9 | 27 | Testis4Species |
| GO:0007257 | activation of JUN kinase activity | 8.14E-03 | 1.42E-03 | 30 | 55 | Testis4Species |
| GO:1900153 | positive regulation of nuclear-transcribed mRNA catabolic process, deadenylation-dependent decay | 8.80E-03 | 1.55E-03 | 2 | 14 | Testis4Species |
| GO:0035235 | ionotropic glutamate receptor signaling pathway | 8.80E-03 | 1.55E-03 | 2 | 14 | Testis4Species |
| GO:0061001 | regulation of dendritic spine morphogenesis | 8.80E-03 | 1.55E-03 | 2 | 14 | Testis4Species |
| GO:0035090 | maintenance of apical/basal cell polarity | 9.61E-03 | 1.70E-03 | 5 | 19 | Testis4Species |
| GO:0060019 | radial glial cell differentiation | 1.02E-02 | 1.82E-03 | 3 | 16 | Testis4Species |
| GO:1900006 | positive regulation of dendrite development | 1.02E-02 | 1.82E-03 | 3 | 16 | Testis4Species |
| GO:0006349 | regulation of gene expression by genetic imprinting | 1.02E-02 | 1.83E-03 | 4 | 17 | Testis4Species |
| GO:0019933 | cAMP-mediated signaling | 1.13E-02 | 2.05E-03 | 8 | 24 | Testis4Species |
| GO:0045634 | regulation of melanocyte differentiation | 1.19E-02 | 2.17E-03 | 1 | 11 | Testis4Species |
| GO:0090286 | cytoskeletal anchoring at nuclear membrane | 1.19E-02 | 2.17E-03 | 1 | 11 | Testis4Species |
| GO:0070509 | calcium ion import | 1.19E-02 | 2.17E-03 | 1 | 11 | Testis4Species |
| GO:0007131 | reciprocal meiotic recombination | 1.23E-02 | 2.31E-03 | 7 | 22 | Testis4Species |
| GO:0010811 | positive regulation of cell-substrate adhesion | 1.23E-02 | 2.31E-03 | 7 | 22 | Testis4Species |

| | | | | | | |
|---|---|---|---|---|---|---|
| GO:0046135 | pyrimidine nucleoside catabolic process | 1.24E-02 | 2.37E-03 | 0 | 8 | Testis4Species |
| GO:0045650 | negative regulation of macrophage differentiation | 1.24E-02 | 2.37E-03 | 0 | 8 | Testis4Species |
| GO:0022038 | corpus callosum development | 1.24E-02 | 2.37E-03 | 0 | 8 | Testis4Species |
| GO:0007157 | heterophilic cell-cell adhesion | 1.24E-02 | 2.37E-03 | 0 | 8 | Testis4Species |
| GO:0007063 | regulation of sister chromatid cohesion | 1.24E-02 | 2.37E-03 | 0 | 8 | Testis4Species |
| GO:0071549 | cellular response to dexamethasone stimulus | 1.24E-02 | 2.37E-03 | 0 | 8 | Testis4Species |
| GO:1902202 | regulation of hepatocyte growth factor receptor signaling pathway | 1.24E-02 | 2.37E-03 | 0 | 8 | Testis4Species |
| GO:0002087 | regulation of respiratory gaseous exchange by neurological system process | 1.24E-02 | 2.37E-03 | 0 | 8 | Testis4Species |
| GO:0051823 | regulation of synapse structural plasticity | 1.24E-02 | 2.37E-03 | 0 | 8 | Testis4Species |
| GO:0044458 | motile cilium assembly | 1.24E-02 | 2.37E-03 | 0 | 8 | Testis4Species |
| GO:0044380 | protein localization to cytoskeleton | 1.24E-02 | 2.37E-03 | 0 | 8 | Testis4Species |
| GO:1901350 | cell-cell signaling involved in cell-cell junction organization | 1.24E-02 | 2.37E-03 | 0 | 8 | Testis4Species |
| GO:0048669 | collateral sprouting in absence of injury | 1.24E-02 | 2.37E-03 | 0 | 8 | Testis4Species |
| GO:0090024 | negative regulation of neutrophil chemotaxis | 1.24E-02 | 2.37E-03 | 0 | 8 | Testis4Species |
| GO:0043619 | regulation of transcription from RNA polymerase II promoter in response to oxidative stress | 1.24E-02 | 2.37E-03 | 0 | 8 | Testis4Species |
| GO:0044027 | hypermethylation of CpG island | 1.24E-02 | 2.37E-03 | 0 | 8 | Testis4Species |
| GO:0031579 | membrane raft organization | 1.24E-02 | 2.37E-03 | 0 | 8 | Testis4Species |
| GO:0043517 | positive regulation of DNA damage response, signal transduction by p53 class mediator | 1.24E-02 | 2.37E-03 | 0 | 8 | Testis4Species |
| GO:0036372 | opsin transport | 1.24E-02 | 2.37E-03 | 0 | 8 | Testis4Species |
| GO:0051012 | microtubule sliding | 1.24E-02 | 2.37E-03 | 0 | 8 | Testis4Species |
| GO:0036159 | inner dynein arm assembly | 1.24E-02 | 2.37E-03 | 0 | 8 | Testis4Species |
| GO:0036158 | outer dynein arm assembly | 1.24E-02 | 2.37E-03 | 0 | 8 | Testis4Species |
| GO:0035621 | ER to Golgi ceramide transport | 1.24E-02 | 2.37E-03 | 0 | 8 | Testis4Species |
| GO:0015864 | pyrimidine nucleoside transport | 1.24E-02 | 2.37E-03 | 0 | 8 | Testis4Species |
| GO:0015855 | pyrimidine nucleobase transport | 1.24E-02 | 2.37E-03 | 0 | 8 | Testis4Species |
| GO:0086019 | cell-cell signaling involved in cardiac conduction | 1.24E-02 | 2.37E-03 | 0 | 8 | Testis4Species |
| GO:0008340 | determination of adult lifespan | 1.24E-02 | 2.37E-03 | 0 | 8 | Testis4Species |
| GO:0060718 | chorionic trophoblast cell differentiation | 1.24E-02 | 2.37E-03 | 0 | 8 | Testis4Species |
| GO:0043433 | negative regulation of sequence-specific DNA binding transcription factor activity | 1.29E-02 | 2.48E-03 | 35 | 59 | Testis4Species |
| GO:0045055 | regulated secretory pathway | 1.34E-02 | 2.60E-03 | 12 | 29 | Testis4Species |
| GO:0001890 | placenta development | 1.41E-02 | 2.73E-03 | 33 | 56 | Testis4Species |
| GO:2001240 | negative regulation of extrinsic apoptotic signaling pathway in absence of ligand | 1.48E-02 | 2.89E-03 | 5 | 18 | Testis4Species |
| GO:0030282 | bone mineralization | 1.50E-02 | 2.94E-03 | 23 | 44 | Testis4Species |
| GO:0051568 | histone H3-K4 methylation | 1.61E-02 | 3.18E-03 | 4 | 16 | Testis4Species |
| GO:0086064 | cell communication by electrical coupling involved in cardiac conduction | 1.61E-02 | 3.18E-03 | 4 | 16 | Testis4Species |
| GO:0006379 | mRNA cleavage | 1.62E-02 | 3.21E-03 | 3 | 15 | Testis4Species |
| GO:0042059 | negative regulation of epidermal growth factor receptor signaling pathway | 1.62E-02 | 3.21E-03 | 3 | 15 | Testis4Species |
| GO:0014047 | glutamate secretion | 1.67E-02 | 3.33E-03 | 15 | 33 | Testis4Species |
| GO:0006607 | NLS-bearing substrate import into nucleus | 1.87E-02 | 3.74E-03 | 7 | 21 | Testis4Species |
| GO:0035411 | catenin import into nucleus | 2.01E-02 | 4.05E-03 | 16 | 34 | Testis4Species |
| GO:0006107 | oxaloacetate metabolic process | 2.08E-02 | 4.22E-03 | 1 | 10 | Testis4Species |
| GO:0016480 | negative regulation of transcription from RNA polymerase III promoter | 2.08E-02 | 4.22E-03 | 1 | 10 | Testis4Species |
| GO:0030214 | hyaluronan catabolic process | 2.08E-02 | 4.22E-03 | 1 | 10 | Testis4Species |
| GO:0006165 | nucleoside diphosphate phosphorylation | 2.12E-02 | 4.43E-03 | 9 | 24 | Testis4Species |
| GO:0051302 | regulation of cell division | 2.13E-02 | 4.46E-03 | 35 | 57 | Testis4Species |
| GO:0050885 | neuromuscular process controlling balance | 2.29E-02 | 4.82E-03 | 11 | 26 | Testis4Species |
| GO:0038095 | Fc-epsilon receptor signaling pathway | 2.29E-02 | 4.83E-03 | 39 | 62 | Testis4Species |
| GO:0060043 | regulation of cardiac muscle cell proliferation | 2.30E-02 | 4.87E-03 | 5 | 17 | Testis4Species |
| GO:0046835 | carbohydrate phosphorylation | 2.30E-02 | 4.87E-03 | 5 | 17 | Testis4Species |
| GO:0045599 | negative regulation of fat cell differentiation | 2.30E-02 | 4.88E-03 | 13 | 29 | Testis4Species |
| GO:0019048 | modulation by virus of host morphology or physiology | 2.30E-02 | 5.01E-03 | 90 | 118 | Testis4Species |
| GO:0006978 | DNA damage response, signal transduction by p53 class mediator resulting in transcription of p21 class mediator | 2.30E-02 | 5.05E-03 | 0 | 7 | Testis4Species |
| GO:0060339 | negative regulation of type I interferon-mediated signaling pathway | 2.30E-02 | 5.05E-03 | 0 | 7 | Testis4Species |
| GO:0019100 | male germ-line sex determination | 2.30E-02 | 5.05E-03 | 0 | 7 | Testis4Species |
| GO:0060134 | prepulse inhibition | 2.30E-02 | 5.05E-03 | 0 | 7 | Testis4Species |
| GO:0033320 | UDP-D-xylose biosynthetic process | 2.30E-02 | 5.05E-03 | 0 | 7 | Testis4Species |
| GO:0001778 | plasma membrane repair | 2.30E-02 | 5.05E-03 | 0 | 7 | Testis4Species |
| GO:2000587 | negative regulation of platelet-derived growth factor receptor-beta signaling pathway | 2.30E-02 | 5.05E-03 | 0 | 7 | Testis4Species |
| GO:0032853 | positive regulation of Ran GTPase activity | 2.30E-02 | 5.05E-03 | 0 | 7 | Testis4Species |
| GO:0006537 | glutamate biosynthetic process | 2.30E-02 | 5.05E-03 | 0 | 7 | Testis4Species |
| GO:1902233 | negative regulation of positive thymic T cell selection | 2.30E-02 | 5.05E-03 | 0 | 7 | Testis4Species |
| GO:1902227 | negative regulation of macrophage colony-stimulating factor signaling pathway | 2.30E-02 | 5.05E-03 | 0 | 7 | Testis4Species |
| GO:1902215 | negative regulation of interleukin-4-mediated signaling pathway | 2.30E-02 | 5.05E-03 | 0 | 7 | Testis4Species |
| GO:1902212 | negative regulation of prolactin signaling pathway | 2.30E-02 | 5.05E-03 | 0 | 7 | Testis4Species |
| GO:1902206 | negative regulation of interleukin-2-mediated signaling pathway | 2.30E-02 | 5.05E-03 | 0 | 7 | Testis4Species |
| GO:0070932 | histone H3 deacetylation | 2.30E-02 | 5.05E-03 | 0 | 7 | Testis4Species |
| GO:0051645 | Golgi localization | 2.30E-02 | 5.05E-03 | 0 | 7 | Testis4Species |
| GO:0051598 | meiotic recombination checkpoint | 2.30E-02 | 5.05E-03 | 0 | 7 | Testis4Species |
| GO:0051570 | regulation of histone H3-K9 methylation | 2.30E-02 | 5.05E-03 | 0 | 7 | Testis4Species |
| GO:0048769 | sarcomerogenesis | 2.30E-02 | 5.05E-03 | 0 | 7 | Testis4Species |
| GO:0018095 | protein polyglutamylation | 2.30E-02 | 5.05E-03 | 0 | 7 | Testis4Species |
| GO:0090116 | C-5 methylation of cytosine | 2.30E-02 | 5.05E-03 | 0 | 7 | Testis4Species |
| GO:0050930 | induction of positive chemotaxis | 2.30E-02 | 5.05E-03 | 0 | 7 | Testis4Species |
| GO:0050911 | detection of chemical stimulus involved in sensory perception of smell | 2.30E-02 | 5.05E-03 | 0 | 7 | Testis4Species |
| GO:0016973 | poly(A)+ mRNA export from nucleus | 2.30E-02 | 5.05E-03 | 0 | 7 | Testis4Species |
| GO:0043584 | nose development | 2.30E-02 | 5.05E-03 | 0 | 7 | Testis4Species |
| GO:0051177 | meiotic sister chromatid cohesion | 2.30E-02 | 5.05E-03 | 0 | 7 | Testis4Species |
| GO:0048280 | vesicle fusion with Golgi apparatus | 2.30E-02 | 5.05E-03 | 0 | 7 | Testis4Species |
| GO:0070104 | negative regulation of interleukin-6-mediated signaling pathway | 2.30E-02 | 5.05E-03 | 0 | 7 | Testis4Species |
| GO:0030860 | regulation of polarized epithelial cell differentiation | 2.30E-02 | 5.05E-03 | 0 | 7 | Testis4Species |
| GO:0050435 | beta-amyloid metabolic process | 2.30E-02 | 5.05E-03 | 0 | 7 | Testis4Species |
| GO:0000019 | regulation of mitotic recombination | 2.30E-02 | 5.05E-03 | 0 | 7 | Testis4Species |
| GO:0015871 | choline transport | 2.30E-02 | 5.05E-03 | 0 | 7 | Testis4Species |
| GO:0035404 | histone-serine phosphorylation | 2.30E-02 | 5.05E-03 | 0 | 7 | Testis4Species |
| GO:0030578 | PML body organization | 2.30E-02 | 5.05E-03 | 0 | 7 | Testis4Species |
| GO:0086091 | regulation of heart rate by cardiac conduction | 2.30E-02 | 5.05E-03 | 0 | 7 | Testis4Species |
| GO:0010804 | negative regulation of tumor necrosis factor-mediated signaling pathway | 2.30E-02 | 5.05E-03 | 0 | 7 | Testis4Species |
| GO:0034721 | histone H3-K4 demethylation, trimethyl-H3-K4-specific | 2.30E-02 | 5.05E-03 | 0 | 7 | Testis4Species |
| GO:0035115 | embryonic forelimb morphogenesis | 2.30E-02 | 5.05E-03 | 0 | 7 | Testis4Species |
| GO:0042138 | meiotic DNA double-strand break formation | 2.30E-02 | 5.05E-03 | 0 | 7 | Testis4Species |
| GO:0060629 | regulation of homologous chromosome segregation | 2.30E-02 | 5.05E-03 | 0 | 7 | Testis4Species |
| GO:0007512 | adult heart development | 2.30E-02 | 5.05E-03 | 0 | 7 | Testis4Species |
| GO:0048925 | lateral line system development | 2.34E-02 | 5.15E-03 | 8 | 22 | Testis4Species |
| GO:0050728 | negative regulation of inflammatory response | 2.34E-02 | 5.15E-03 | 8 | 22 | Testis4Species |
| GO:1901863 | positive regulation of muscle tissue development | 2.36E-02 | 5.23E-03 | 2 | 12 | Testis4Species |
| GO:0070934 | CRD-mediated mRNA stabilization | 2.36E-02 | 5.23E-03 | 2 | 12 | Testis4Species |
| GO:0042487 | regulation of odontogenesis of dentin-containing tooth | 2.36E-02 | 5.23E-03 | 2 | 12 | Testis4Species |
| GO:0046487 | glyoxylate metabolic process | 2.36E-02 | 5.23E-03 | 2 | 12 | Testis4Species |
| GO:0045428 | regulation of nitric oxide biosynthetic process | 2.46E-02 | 5.49E-03 | 4 | 15 | Testis4Species |
| GO:0014037 | Schwann cell differentiation | 2.46E-02 | 5.49E-03 | 4 | 15 | Testis4Species |
| GO:0006342 | chromatin silencing | 2.46E-02 | 5.49E-03 | 4 | 15 | Testis4Species |
| GO:0032000 | positive regulation of fatty acid beta-oxidation | 2.46E-02 | 5.49E-03 | 4 | 15 | Testis4Species |
| GO:0030206 | chondroitin sulfate biosynthetic process | 2.46E-02 | 5.49E-03 | 4 | 15 | Testis4Species |
| GO:0008045 | motor neuron axon guidance | 2.50E-02 | 5.61E-03 | 3 | 14 | Testis4Species |
| GO:0046631 | alpha-beta T cell activation | 3.20E-02 | 7.31E-03 | 11 | 25 | Testis4Species |

| | | | | | | |
|---|---|---|---|---|---|---|
| GO:0006386 | termination of RNA polymerase III transcription | 3.48E-02 | 8.07E-03 | 8 | 21 | Testis4Species |
| GO:0006385 | transcription elongation from RNA polymerase III promoter | 3.48E-02 | 8.07E-03 | 8 | 21 | Testis4Species |
| GO:0000413 | protein peptidyl-prolyl isomerization | 3.48E-02 | 8.07E-03 | 8 | 21 | Testis4Species |
| GO:0033198 | response to ATP | 3.49E-02 | 8.12E-03 | 5 | 16 | Testis4Species |
| GO:0070527 | platelet aggregation | 3.49E-02 | 8.12E-03 | 5 | 16 | Testis4Species |
| GO:0046519 | sphingoid metabolic process | 3.49E-02 | 8.12E-03 | 5 | 16 | Testis4Species |
| GO:0072383 | plus-end-directed vesicle transport along microtubule | 3.49E-02 | 8.18E-03 | 1 | 9 | Testis4Species |
| GO:0006863 | purine nucleobase transport | 3.49E-02 | 8.18E-03 | 1 | 9 | Testis4Species |
| GO:0007194 | negative regulation of adenylate cyclase activity | 3.49E-02 | 8.18E-03 | 1 | 9 | Testis4Species |
| GO:0032957 | inositol trisphosphate metabolic process | 3.49E-02 | 8.18E-03 | 1 | 9 | Testis4Species |
| GO:0033152 | immunoglobulin V(D)J recombination | 3.49E-02 | 8.18E-03 | 1 | 9 | Testis4Species |
| GO:0006303 | double-strand break repair via nonhomologous end joining | 3.49E-02 | 8.18E-03 | 1 | 9 | Testis4Species |
| GO:0032330 | regulation of chondrocyte differentiation | 3.49E-02 | 8.18E-03 | 1 | 9 | Testis4Species |
| GO:0010611 | regulation of cardiac muscle hypertrophy | 3.49E-02 | 8.18E-03 | 1 | 9 | Testis4Species |
| GO:0097120 | receptor localization to synapse | 3.49E-02 | 8.18E-03 | 1 | 9 | Testis4Species |
| GO:0060501 | positive regulation of epithelial cell proliferation involved in lung morphogenesis | 3.49E-02 | 8.18E-03 | 1 | 9 | Testis4Species |
| GO:0070373 | negative regulation of ERK1 and ERK2 cascade | 3.57E-02 | 8.43E-03 | 16 | 32 | Testis4Species |
| GO:0030866 | cortical actin cytoskeleton organization | 3.60E-02 | 8.68E-03 | 14 | 29 | Testis4Species |
| GO:2001014 | regulation of skeletal muscle cell differentiation | 3.84E-02 | 9.37E-03 | 4 | 14 | Testis4Species |
| GO:0034616 | response to laminar fluid shear stress | 3.89E-02 | 9.50E-03 | 2 | 11 | Testis4Species |
| GO:0007509 | mesoderm migration involved in gastrulation | 3.89E-02 | 9.50E-03 | 2 | 11 | Testis4Species |
| GO:0006833 | water transport | 3.90E-02 | 9.54E-03 | 7 | 19 | Testis4Species |
| GO:0021702 | cerebellar Purkinje cell differentiation | 3.96E-02 | 9.73E-03 | 3 | 13 | Testis4Species |
| GO:0060316 | positive regulation of ryanodine-sensitive calcium-release channel activity | 3.96E-02 | 9.73E-03 | 3 | 13 | Testis4Species |
| GO:0007595 | lactation | 4.02E-02 | 9.89E-03 | 17 | 33 | Testis4Species |
| GO:0060325 | face morphogenesis | 4.23E-02 | 1.07E-02 | 32 | 51 | Testis4Species |
| GO:0045721 | negative regulation of gluconeogenesis | 4.23E-02 | 1.08E-02 | 0 | 6 | Testis4Species |
| GO:0046108 | uridine metabolic process | 4.23E-02 | 1.08E-02 | 0 | 6 | Testis4Species |
| GO:0045672 | positive regulation of osteoclast differentiation | 4.23E-02 | 1.08E-02 | 0 | 6 | Testis4Species |
| GO:0021678 | third ventricle development | 4.23E-02 | 1.08E-02 | 0 | 6 | Testis4Species |
| GO:0007352 | zygotic specification of dorsal/ventral axis | 4.23E-02 | 1.08E-02 | 0 | 6 | Testis4Species |
| GO:0010044 | response to aluminum ion | 4.23E-02 | 1.08E-02 | 0 | 6 | Testis4Species |
| GO:0019227 | neuronal action potential propagation | 4.23E-02 | 1.08E-02 | 0 | 6 | Testis4Species |
| GO:0072234 | metanephric nephron tubule development | 4.23E-02 | 1.08E-02 | 0 | 6 | Testis4Species |
| GO:2000507 | positive regulation of energy homeostasis | 4.23E-02 | 1.08E-02 | 0 | 6 | Testis4Species |
| GO:0051955 | regulation of amino acid transport | 4.23E-02 | 1.08E-02 | 0 | 6 | Testis4Species |
| GO:0033278 | cell proliferation in midbrain | 4.23E-02 | 1.08E-02 | 0 | 6 | Testis4Species |
| GO:0045123 | cellular extravasation | 4.23E-02 | 1.08E-02 | 0 | 6 | Testis4Species |
| GO:0071305 | cellular response to vitamin D | 4.23E-02 | 1.08E-02 | 0 | 6 | Testis4Species |
| GO:0090403 | oxidative stress-induced premature senescence | 4.23E-02 | 1.08E-02 | 0 | 6 | Testis4Species |
| GO:0006288 | base-excision repair, DNA ligation | 4.23E-02 | 1.08E-02 | 0 | 6 | Testis4Species |
| GO:0051532 | regulation of NFAT protein import into nucleus | 4.23E-02 | 1.08E-02 | 0 | 6 | Testis4Species |
| GO:0051453 | regulation of intracellular pH | 4.23E-02 | 1.08E-02 | 0 | 6 | Testis4Species |
| GO:0032298 | positive regulation of DNA-dependent DNA replication initiation | 4.23E-02 | 1.08E-02 | 0 | 6 | Testis4Species |
| GO:0031659 | positive regulation of cyclin-dependent protein serine/threonine kinase activity involved in G1/S transition of mitotic cell cycl | 4.23E-02 | 1.08E-02 | 0 | 6 | Testis4Species |
| GO:0031630 | regulation of synaptic vesicle fusion to presynaptic membrane | 4.23E-02 | 1.08E-02 | 0 | 6 | Testis4Species |
| GO:0043490 | malate-aspartate shuttle | 4.23E-02 | 1.08E-02 | 0 | 6 | Testis4Species |
| GO:0000301 | retrograde transport, vesicle recycling within Golgi | 4.23E-02 | 1.08E-02 | 0 | 6 | Testis4Species |
| GO:0051014 | actin filament severing | 4.23E-02 | 1.08E-02 | 0 | 6 | Testis4Species |
| GO:0042977 | activation of JAK2 kinase activity | 4.23E-02 | 1.08E-02 | 0 | 6 | Testis4Species |
| GO:0042730 | fibrinolysis | 4.23E-02 | 1.08E-02 | 0 | 6 | Testis4Species |
| GO:0000066 | mitochondrial ornithine transport | 4.23E-02 | 1.08E-02 | 0 | 6 | Testis4Species |
| GO:0042660 | positive regulation of cell fate specification | 4.23E-02 | 1.08E-02 | 0 | 6 | Testis4Species |
| GO:0015881 | creatine transport | 4.23E-02 | 1.08E-02 | 0 | 6 | Testis4Species |
| GO:0035428 | hexose transmembrane transport | 4.23E-02 | 1.08E-02 | 0 | 6 | Testis4Species |
| GO:0031065 | positive regulation of histone deacetylation | 4.23E-02 | 1.08E-02 | 0 | 6 | Testis4Species |
| GO:0042541 | hemoglobin biosynthetic process | 4.23E-02 | 1.08E-02 | 0 | 6 | Testis4Species |
| GO:0042512 | negative regulation of tyrosine phosphorylation of Stat1 protein | 4.23E-02 | 1.08E-02 | 0 | 6 | Testis4Species |
| GO:0086013 | membrane repolarization involved in regulation of cardiac muscle cell action potential | 4.23E-02 | 1.08E-02 | 0 | 6 | Testis4Species |
| GO:0016199 | axon midline choice point recognition | 4.23E-02 | 1.08E-02 | 0 | 6 | Testis4Species |
| GO:0046689 | response to mercury ion | 4.23E-02 | 1.08E-02 | 0 | 6 | Testis4Species |
| GO:0008211 | glucocorticoid metabolic process | 4.23E-02 | 1.08E-02 | 0 | 6 | Testis4Species |
| GO:0042036 | negative regulation of cytokine biosynthetic process | 4.23E-02 | 1.08E-02 | 0 | 6 | Testis4Species |
| GO:0021942 | radial glia guided migration of Purkinje cell | 4.23E-02 | 1.08E-02 | 0 | 6 | Testis4Species |
| GO:0045898 | regulation of RNA polymerase II transcriptional preinitiation complex assembly | 4.23E-02 | 1.08E-02 | 0 | 6 | Testis4Species |
| GO:0080163 | regulation of protein serine/threonine phosphatase activity | 4.23E-02 | 1.08E-02 | 0 | 6 | Testis4Species |
| GO:0060586 | multicellular organismal iron ion homeostasis | 4.23E-02 | 1.08E-02 | 0 | 6 | Testis4Species |
| GO:0001841 | neural tube formation | 4.80E-02 | 1.24E-02 | 45 | 65 | Testis4Species |

**Enriched in Ovary in *A. burtoni***

| GO-ID | Term | FDR | P-Value | #OTranscripts | #TTranscripts | Enriched in |
|---|---|---|---|---|---|---|
| GO:0006805 | xenobiotic metabolic process | 3.06E-06 | 2.36E-08 | 28 | 1 | OvaryAburtoni |
| GO:0006750 | glutathione biosynthetic process | 5.29E-06 | 4.87E-08 | 23 | 0 | OvaryAburtoni |
| GO:1901687 | glutathione derivative biosynthetic process | 5.29E-06 | 4.87E-08 | 23 | 0 | OvaryAburtoni |
| GO:0007269 | neurotransmitter secretion | 1.64E-05 | 1.73E-07 | 40 | 7 | OvaryAburtoni |
| GO:0051092 | positive regulation of NF-kappaB transcription factor activity | 1.72E-05 | 1.90E-07 | 25 | 1 | OvaryAburtoni |
| GO:0090179 | planar cell polarity pathway involved in neural tube closure | 3.40E-05 | 4.42E-07 | 20 | 0 | OvaryAburtoni |
| GO:0022007 | convergent extension involved in neural plate elongation | 5.88E-05 | 9.22E-07 | 19 | 0 | OvaryAburtoni |
| GO:0035372 | protein localization to microtubule | 5.88E-05 | 9.22E-07 | 19 | 0 | OvaryAburtoni |
| GO:0071340 | skeletal muscle acetylcholine-gated channel clustering | 5.88E-05 | 9.22E-07 | 19 | 0 | OvaryAburtoni |
| GO:0043123 | positive regulation of I-kappaB kinase/NF-kappaB cascade | 9.50E-05 | 1.60E-06 | 25 | 2 | OvaryAburtoni |
| GO:0048668 | collateral sprouting | 1.69E-04 | 3.01E-06 | 21 | 1 | OvaryAburtoni |
| GO:0043687 | post-translational protein modification | 1.72E-04 | 3.07E-06 | 24 | 2 | OvaryAburtoni |
| GO:0032312 | regulation of ARF GTPase activity | 2.03E-04 | 4.00E-06 | 17 | 0 | OvaryAburtoni |
| GO:0034138 | toll-like receptor 3 signaling pathway | 2.03E-04 | 4.00E-06 | 17 | 0 | OvaryAburtoni |
| GO:0034134 | toll-like receptor 2 signaling pathway | 2.03E-04 | 4.00E-06 | 17 | 0 | OvaryAburtoni |
| GO:0035666 | TRIF-dependent toll-like receptor signaling pathway | 3.64E-04 | 8.33E-06 | 16 | 0 | OvaryAburtoni |
| GO:0038124 | toll-like receptor TLR6:TLR2 signaling pathway | 3.64E-04 | 8.33E-06 | 16 | 0 | OvaryAburtoni |
| GO:0038123 | toll-like receptor TLR1:TLR2 signaling pathway | 3.64E-04 | 8.33E-06 | 16 | 0 | OvaryAburtoni |
| GO:0002755 | MyD88-dependent toll-like receptor signaling pathway | 3.64E-04 | 8.33E-06 | 16 | 0 | OvaryAburtoni |
| GO:0034166 | toll-like receptor 10 signaling pathway | 3.64E-04 | 8.33E-06 | 16 | 0 | OvaryAburtoni |
| GO:0034162 | toll-like receptor 9 signaling pathway | 3.64E-04 | 8.33E-06 | 16 | 0 | OvaryAburtoni |
| GO:0034146 | toll-like receptor 5 signaling pathway | 3.64E-04 | 8.33E-06 | 16 | 0 | OvaryAburtoni |
| GO:0031122 | cytoplasmic microtubule organization | 4.89E-04 | 1.18E-05 | 19 | 1 | OvaryAburtoni |
| GO:0090263 | positive regulation of canonical Wnt receptor signaling pathway | 6.61E-04 | 1.66E-05 | 24 | 3 | OvaryAburtoni |
| GO:0030148 | sphingolipid biosynthetic process | 6.72E-04 | 1.73E-05 | 15 | 0 | OvaryAburtoni |
| GO:0051260 | protein homooligomerization | 8.76E-04 | 2.29E-05 | 46 | 15 | OvaryAburtoni |
| GO:0090398 | cellular senescence | 8.87E-04 | 2.34E-05 | 18 | 1 | OvaryAburtoni |
| GO:0034142 | toll-like receptor 4 signaling pathway | 8.87E-04 | 2.34E-05 | 18 | 1 | OvaryAburtoni |
| GO:0016050 | vesicle organization | 1.44E-03 | 4.41E-05 | 27 | 5 | OvaryAburtoni |
| GO:0046649 | lymphocyte activation | 2.21E-03 | 7.45E-05 | 52 | 21 | OvaryAburtoni |
| GO:0033762 | response to glucagon stimulus | 2.21E-03 | 7.51E-05 | 13 | 0 | OvaryAburtoni |
| GO:0032926 | negative regulation of activin receptor signaling pathway | 3.91E-03 | 1.56E-04 | 12 | 0 | OvaryAburtoni |
| GO:0007097 | nuclear migration | 3.91E-03 | 1.56E-04 | 12 | 0 | OvaryAburtoni |
| GO:0042348 | NF-kappaB import into nucleus | 3.91E-03 | 1.56E-04 | 12 | 0 | OvaryAburtoni |

| | | | | | | |
|---|---|---|---|---|---|---|
| GO:0006271 | DNA strand elongation involved in DNA replication | 3.91E-03 | 1.56E-04 | 12 | 0 | OvaryAburtoni |
| GO:0042157 | lipoprotein metabolic process | 3.91E-03 | 1.56E-04 | 12 | 0 | OvaryAburtoni |
| GO:0042147 | retrograde transport, endosome to Golgi | 3.91E-03 | 1.56E-04 | 12 | 0 | OvaryAburtoni |
| GO:0035999 | tetrahydrofolate interconversion | 3.91E-03 | 1.56E-04 | 12 | 0 | OvaryAburtoni |
| GO:0043589 | skin morphogenesis | 4.34E-03 | 1.78E-04 | 15 | 1 | OvaryAburtoni |
| GO:0032874 | positive regulation of stress-activated MAPK cascade | 5.12E-03 | 2.15E-04 | 22 | 4 | OvaryAburtoni |
| GO:0031532 | actin cytoskeleton reorganization | 5.12E-03 | 2.15E-04 | 22 | 4 | OvaryAburtoni |
| GO:0060260 | regulation of transcription initiation from RNA polymerase II promoter | 7.12E-03 | 3.25E-04 | 11 | 0 | OvaryAburtoni |
| GO:0043094 | cellular metabolic compound salvage | 7.12E-03 | 3.25E-04 | 11 | 0 | OvaryAburtoni |
| GO:0038032 | termination of G-protein coupled receptor signaling pathway | 7.12E-03 | 3.25E-04 | 11 | 0 | OvaryAburtoni |
| GO:2001046 | positive regulation of integrin-mediated signaling pathway | 7.12E-03 | 3.25E-04 | 11 | 0 | OvaryAburtoni |
| GO:0045812 | negative regulation of Wnt receptor signaling pathway, calcium modulating pathway | 7.12E-03 | 3.25E-04 | 11 | 0 | OvaryAburtoni |
| GO:0065004 | protein-DNA complex assembly | 7.54E-03 | 3.48E-04 | 14 | 1 | OvaryAburtoni |
| GO:0043401 | steroid hormone mediated signaling pathway | 8.26E-03 | 3.95E-04 | 23 | 5 | OvaryAburtoni |
| GO:0006801 | superoxide metabolic process | 1.03E-02 | 5.05E-04 | 16 | 2 | OvaryAburtoni |
| GO:0007059 | chromosome segregation | 1.03E-02 | 5.05E-04 | 16 | 2 | OvaryAburtoni |
| GO:0032436 | positive regulation of proteasomal ubiquitin-dependent protein catabolic process | 1.20E-02 | 6.61E-04 | 20 | 4 | OvaryAburtoni |
| GO:0006729 | tetrahydrobiopterin biosynthetic process | 1.20E-02 | 6.75E-04 | 10 | 0 | OvaryAburtoni |
| GO:0009247 | glycolipid biosynthetic process | 1.20E-02 | 6.75E-04 | 10 | 0 | OvaryAburtoni |
| GO:0014059 | regulation of dopamine secretion | 1.20E-02 | 6.75E-04 | 10 | 0 | OvaryAburtoni |
| GO:0033133 | positive regulation of glucokinase activity | 1.20E-02 | 6.75E-04 | 10 | 0 | OvaryAburtoni |
| GO:0009060 | aerobic respiration | 1.20E-02 | 6.75E-04 | 10 | 0 | OvaryAburtoni |
| GO:0071379 | cellular response to prostaglandin stimulus | 1.20E-02 | 6.75E-04 | 10 | 0 | OvaryAburtoni |
| GO:0010499 | proteasomal ubiquitin-independent protein catabolic process | 1.20E-02 | 6.75E-04 | 10 | 0 | OvaryAburtoni |
| GO:0070423 | nucleotide-binding oligomerization domain containing signaling pathway | 1.20E-02 | 6.75E-04 | 10 | 0 | OvaryAburtoni |
| GO:0043524 | negative regulation of neuron apoptotic process | 1.20E-02 | 6.75E-04 | 10 | 0 | OvaryAburtoni |
| GO:0006919 | activation of cysteine-type endopeptidase activity involved in apoptotic process | 1.20E-02 | 6.79E-04 | 13 | 1 | OvaryAburtoni |
| GO:0046835 | carbohydrate phosphorylation | 1.85E-02 | 1.14E-03 | 21 | 5 | OvaryAburtoni |
| GO:0007067 | mitosis | 1.95E-02 | 1.22E-03 | 32 | 12 | OvaryAburtoni |
| GO:0006739 | NADP metabolic process | 2.06E-02 | 1.32E-03 | 12 | 1 | OvaryAburtoni |
| GO:0030318 | melanocyte differentiation | 2.06E-02 | 1.32E-03 | 12 | 1 | OvaryAburtoni |
| GO:0006012 | galactose metabolic process | 2.06E-02 | 1.32E-03 | 12 | 1 | OvaryAburtoni |
| GO:0060218 | hematopoietic stem cell differentiation | 2.07E-02 | 1.40E-03 | 9 | 0 | OvaryAburtoni |
| GO:0001833 | inner cell mass cell proliferation | 2.07E-02 | 1.40E-03 | 9 | 0 | OvaryAburtoni |
| GO:0036089 | cleavage furrow formation | 2.07E-02 | 1.40E-03 | 9 | 0 | OvaryAburtoni |
| GO:0015939 | pantothenate metabolic process | 2.07E-02 | 1.40E-03 | 9 | 0 | OvaryAburtoni |
| GO:0045176 | apical protein localization | 2.07E-02 | 1.40E-03 | 9 | 0 | OvaryAburtoni |
| GO:0090399 | replicative senescence | 2.07E-02 | 1.40E-03 | 9 | 0 | OvaryAburtoni |
| GO:0035194 | posttranscriptional gene silencing by RNA | 2.07E-02 | 1.40E-03 | 9 | 0 | OvaryAburtoni |
| GO:0060900 | embryonic camera-type eye formation | 2.07E-02 | 1.40E-03 | 9 | 0 | OvaryAburtoni |
| GO:0019853 | L-ascorbic acid biosynthetic process | 2.07E-02 | 1.40E-03 | 9 | 0 | OvaryAburtoni |
| GO:0006071 | glycerol metabolic process | 2.07E-02 | 1.40E-03 | 9 | 0 | OvaryAburtoni |
| GO:0032053 | microtubule basal body organization | 2.07E-02 | 1.40E-03 | 9 | 0 | OvaryAburtoni |
| GO:0046185 | aldehyde catabolic process | 2.07E-02 | 1.40E-03 | 9 | 0 | OvaryAburtoni |
| GO:0046173 | polyol biosynthetic process | 2.07E-02 | 1.40E-03 | 9 | 0 | OvaryAburtoni |
| GO:0046135 | pyrimidine nucleoside catabolic process | 2.07E-02 | 1.40E-03 | 9 | 0 | OvaryAburtoni |
| GO:0072384 | organelle transport along microtubule | 2.07E-02 | 1.40E-03 | 9 | 0 | OvaryAburtoni |
| GO:0031100 | organ regeneration | 2.73E-02 | 1.93E-03 | 16 | 3 | OvaryAburtoni |
| GO:0010639 | negative regulation of organelle organization | 3.12E-02 | 2.34E-03 | 24 | 8 | OvaryAburtoni |
| GO:0006487 | protein N-linked glycosylation | 3.26E-02 | 2.54E-03 | 11 | 1 | OvaryAburtoni |
| GO:0006090 | pyruvate metabolic process | 3.26E-02 | 2.54E-03 | 11 | 1 | OvaryAburtoni |
| GO:0019264 | glycine biosynthetic process from serine | 3.39E-02 | 2.92E-03 | 8 | 0 | OvaryAburtoni |
| GO:0007252 | I-kappaB phosphorylation | 3.39E-02 | 2.92E-03 | 8 | 0 | OvaryAburtoni |
| GO:0042840 | D-glucuronate catabolic process | 3.39E-02 | 2.92E-03 | 8 | 0 | OvaryAburtoni |
| GO:0000185 | activation of MAPKKK activity | 3.39E-02 | 2.92E-03 | 8 | 0 | OvaryAburtoni |
| GO:0046985 | positive regulation of hemoglobin biosynthetic process | 3.39E-02 | 2.92E-03 | 8 | 0 | OvaryAburtoni |
| GO:0009155 | purine deoxyribonucleotide catabolic process | 3.39E-02 | 2.92E-03 | 8 | 0 | OvaryAburtoni |
| GO:0042462 | eye photoreceptor cell development | 3.39E-02 | 2.92E-03 | 8 | 0 | OvaryAburtoni |
| GO:0042417 | dopamine metabolic process | 3.39E-02 | 2.92E-03 | 8 | 0 | OvaryAburtoni |
| GO:0010833 | telomere maintenance via telomere lengthening | 3.39E-02 | 2.92E-03 | 8 | 0 | OvaryAburtoni |
| GO:0016064 | immunoglobulin mediated immune response | 3.39E-02 | 2.92E-03 | 8 | 0 | OvaryAburtoni |
| GO:0000972 | transcription-dependent tethering of RNA polymerase II gene DNA at nuclear periphery | 3.39E-02 | 2.92E-03 | 8 | 0 | OvaryAburtoni |
| GO:0000956 | nuclear-transcribed mRNA catabolic process | 3.39E-02 | 2.92E-03 | 8 | 0 | OvaryAburtoni |
| GO:0051642 | centrosome localization | 3.39E-02 | 2.92E-03 | 8 | 0 | OvaryAburtoni |
| GO:0043973 | histone H3-K4 acetylation | 3.39E-02 | 2.92E-03 | 8 | 0 | OvaryAburtoni |
| GO:0051568 | histone H3-K4 methylation | 3.39E-02 | 2.92E-03 | 8 | 0 | OvaryAburtoni |
| GO:0006144 | purine nucleobase metabolic process | 3.39E-02 | 2.92E-03 | 8 | 0 | OvaryAburtoni |
| GO:1900107 | regulation of nodal signaling pathway | 3.39E-02 | 2.92E-03 | 8 | 0 | OvaryAburtoni |
| GO:0018022 | peptidyl-lysine methylation | 3.39E-02 | 2.92E-03 | 8 | 0 | OvaryAburtoni |
| GO:0045987 | positive regulation of smooth muscle contraction | 3.39E-02 | 2.92E-03 | 8 | 0 | OvaryAburtoni |
| GO:1901655 | cellular response to ketone | 3.39E-02 | 2.92E-03 | 8 | 0 | OvaryAburtoni |
| GO:0051225 | spindle assembly | 3.39E-02 | 2.92E-03 | 8 | 0 | OvaryAburtoni |
| GO:0021895 | cerebral cortex neuron differentiation | 3.39E-02 | 2.92E-03 | 8 | 0 | OvaryAburtoni |
| GO:0050772 | positive regulation of axonogenesis | 3.39E-02 | 2.92E-03 | 8 | 0 | OvaryAburtoni |
| GO:0019483 | beta-alanine biosynthetic process | 3.39E-02 | 2.92E-03 | 8 | 0 | OvaryAburtoni |
| GO:0021702 | cerebellar Purkinje cell differentiation | 3.39E-02 | 2.92E-03 | 8 | 0 | OvaryAburtoni |
| GO:0045647 | negative regulation of erythrocyte differentiation | 3.39E-02 | 2.92E-03 | 8 | 0 | OvaryAburtoni |
| GO:0045604 | regulation of epidermal cell differentiation | 3.39E-02 | 2.92E-03 | 8 | 0 | OvaryAburtoni |
| GO:0048262 | determination of dorsal/ventral asymmetry | 3.39E-02 | 2.92E-03 | 8 | 0 | OvaryAburtoni |
| GO:0060134 | prepulse inhibition | 3.42E-02 | 2.95E-03 | 21 | 6 | OvaryAburtoni |
| GO:0032388 | positive regulation of intracellular transport | 3.60E-02 | 3.14E-03 | 25 | 9 | OvaryAburtoni |
| GO:0006302 | double-strand break repair | 3.63E-02 | 3.17E-03 | 13 | 2 | OvaryAburtoni |
| GO:0071248 | cellular response to metal ion | 3.63E-02 | 3.17E-03 | 13 | 2 | OvaryAburtoni |
| GO:0031334 | positive regulation of protein complex assembly | 3.65E-02 | 3.21E-03 | 19 | 5 | OvaryAburtoni |
| GO:0030216 | keratinocyte differentiation | 3.85E-02 | 3.41E-03 | 15 | 3 | OvaryAburtoni |
| GO:0035264 | multicellular organism growth | 4.14E-02 | 3.72E-03 | 23 | 8 | OvaryAburtoni |
| GO:0009948 | anterior/posterior axis specification | 4.59E-02 | 4.25E-03 | 22 | 7 | OvaryAburtoni |
| GO:0051100 | negative regulation of binding | 4.59E-02 | 4.25E-03 | 22 | 7 | OvaryAburtoni |
| GO:0009123 | nucleoside monophosphate metabolic process | 4.96E-02 | 4.68E-03 | 41 | 21 | OvaryAburtoni |
| | | | | | | |
| **Enriched in Testis in *A. burtoni*** | | | | | | |
| GO:0051012 | microtubule sliding | 2.87E-07 | 1.32E-09 | 0 | 31 | TestisAburtoni |
| GO:0051294 | establishment of spindle orientation | 1.67E-05 | 1.79E-07 | 2 | 31 | TestisAburtoni |
| GO:0008543 | fibroblast growth factor receptor signaling pathway | 2.36E-05 | 2.74E-07 | 16 | 63 | TestisAburtoni |
| GO:0007173 | epidermal growth factor receptor signaling pathway | 5.66E-05 | 8.19E-07 | 19 | 66 | TestisAburtoni |
| GO:0006693 | prostaglandin metabolic process | 6.95E-05 | 1.14E-06 | 0 | 21 | TestisAburtoni |
| GO:0048011 | neurotrophin TRK receptor signaling pathway | 2.93E-04 | 6.19E-06 | 20 | 63 | TestisAburtoni |
| GO:0046777 | protein autophosphorylation | 3.36E-04 | 7.39E-06 | 15 | 54 | TestisAburtoni |
| GO:0009065 | glutamine family amino acid catabolic process | 6.71E-04 | 1.72E-05 | 0 | 17 | TestisAburtoni |
| GO:0050885 | neuromuscular process controlling balance | 6.71E-04 | 1.72E-05 | 0 | 17 | TestisAburtoni |
| GO:0042384 | cilium assembly | 1.50E-03 | 4.67E-05 | 9 | 38 | TestisAburtoni |
| GO:0050680 | negative regulation of epithelial cell proliferation | 2.21E-03 | 7.65E-05 | 2 | 21 | TestisAburtoni |
| GO:0051897 | positive regulation of protein kinase B signaling cascade | 2.44E-03 | 8.59E-05 | 1 | 18 | TestisAburtoni |
| GO:0007052 | mitotic spindle organization | 3.61E-03 | 1.35E-04 | 7 | 32 | TestisAburtoni |
| GO:0045787 | positive regulation of cell cycle | 3.66E-03 | 1.38E-04 | 2 | 20 | TestisAburtoni |

| | | | | | | |
|---|---|---|---|---|---|---|
| GO:0008286 | insulin receptor signaling pathway | 3.76E-03 | 1.43E-04 | 18 | 52 | TestisAburtoni |
| GO:0048015 | phosphatidylinositol-mediated signaling | 4.01E-03 | 1.64E-04 | 28 | 68 | TestisAburtoni |
| GO:0038095 | Fc-epsilon receptor signaling pathway | 5.57E-03 | 2.36E-04 | 24 | 60 | TestisAburtoni |
| GO:0033540 | fatty acid beta-oxidation using acyl-CoA oxidase | 6.08E-03 | 2.62E-04 | 0 | 13 | TestisAburtoni |
| GO:0055012 | ventricular cardiac muscle cell differentiation | 6.08E-03 | 2.62E-04 | 0 | 13 | TestisAburtoni |
| GO:0033262 | regulation of nuclear cell cycle DNA replication | 6.08E-03 | 2.62E-04 | 0 | 13 | TestisAburtoni |
| GO:0016559 | peroxisome fission | 6.87E-03 | 3.01E-04 | 1 | 16 | TestisAburtoni |
| GO:0036109 | alpha-linolenic acid metabolic process | 1.03E-02 | 5.18E-04 | 0 | 12 | TestisAburtoni |
| GO:0007033 | vacuole organization | 1.03E-02 | 5.18E-04 | 0 | 12 | TestisAburtoni |
| GO:0032715 | negative regulation of interleukin-6 production | 1.03E-02 | 5.18E-04 | 0 | 12 | TestisAburtoni |
| GO:0016236 | macroautophagy | 1.03E-02 | 5.18E-04 | 0 | 12 | TestisAburtoni |
| GO:0042445 | hormone metabolic process | 1.09E-02 | 5.50E-04 | 3 | 20 | TestisAburtoni |
| GO:0055123 | digestive system development | 1.15E-02 | 5.82E-04 | 7 | 29 | TestisAburtoni |
| GO:0006363 | termination of RNA polymerase I transcription | 1.40E-02 | 8.00E-04 | 2 | 17 | TestisAburtoni |
| GO:0006361 | transcription initiation from RNA polymerase I promoter | 1.40E-02 | 8.00E-04 | 2 | 17 | TestisAburtoni |
| GO:0007032 | endosome organization | 1.72E-02 | 1.03E-03 | 0 | 11 | TestisAburtoni |
| GO:0007016 | cytoskeletal anchoring at plasma membrane | 1.72E-02 | 1.03E-03 | 0 | 11 | TestisAburtoni |
| GO:0048857 | neural nucleus development | 1.72E-02 | 1.03E-03 | 0 | 11 | TestisAburtoni |
| GO:0035025 | positive regulation of Rho protein signal transduction | 1.72E-02 | 1.03E-03 | 0 | 11 | TestisAburtoni |
| GO:0014808 | release of sequestered calcium ion into cytosol by sarcoplasmic reticulum | 1.72E-02 | 1.03E-03 | 0 | 11 | TestisAburtoni |
| GO:0014721 | twitch skeletal muscle contraction | 1.72E-02 | 1.03E-03 | 0 | 11 | TestisAburtoni |
| GO:0007602 | phototransduction | 1.72E-02 | 1.03E-03 | 4 | 21 | TestisAburtoni |
| GO:0072331 | signal transduction by p53 class mediator | 1.72E-02 | 1.04E-03 | 5 | 23 | TestisAburtoni |
| GO:0031397 | negative regulation of protein ubiquitination | 1.72E-02 | 1.05E-03 | 1 | 14 | TestisAburtoni |
| GO:0000038 | very long-chain fatty acid metabolic process | 2.09E-02 | 1.42E-03 | 2 | 16 | TestisAburtoni |
| GO:2000189 | positive regulation of cholesterol homeostasis | 2.09E-02 | 1.42E-03 | 2 | 16 | TestisAburtoni |
| GO:0007605 | sensory perception of sound | 2.19E-02 | 1.49E-03 | 7 | 27 | TestisAburtoni |
| GO:0033002 | muscle cell proliferation | 2.37E-02 | 1.64E-03 | 3 | 18 | TestisAburtoni |
| GO:0035774 | positive regulation of insulin secretion involved in cellular response to glucose stimulus | 2.74E-02 | 2.03E-03 | 0 | 10 | TestisAburtoni |
| GO:0001946 | lymphangiogenesis | 2.74E-02 | 2.03E-03 | 0 | 10 | TestisAburtoni |
| GO:0031000 | response to caffeine | 2.74E-02 | 2.03E-03 | 0 | 10 | TestisAburtoni |
| GO:0032717 | negative regulation of interleukin-8 production | 2.74E-02 | 2.03E-03 | 0 | 10 | TestisAburtoni |
| GO:0045162 | clustering of voltage-gated sodium channels | 2.74E-02 | 2.03E-03 | 0 | 10 | TestisAburtoni |
| GO:0090240 | positive regulation of histone H4 acetylation | 2.74E-02 | 2.03E-03 | 0 | 10 | TestisAburtoni |
| GO:0010508 | positive regulation of autophagy | 2.74E-02 | 2.03E-03 | 0 | 10 | TestisAburtoni |
| GO:0060688 | regulation of morphogenesis of a branching structure | 2.74E-02 | 2.03E-03 | 0 | 10 | TestisAburtoni |
| GO:2000134 | negative regulation of G1/S transition of mitotic cell cycle | 2.74E-02 | 2.03E-03 | 0 | 10 | TestisAburtoni |
| GO:0060420 | regulation of heart growth | 2.74E-02 | 2.03E-03 | 0 | 10 | TestisAburtoni |
| GO:0007156 | homophilic cell adhesion | 3.14E-02 | 2.37E-03 | 7 | 26 | TestisAburtoni |
| GO:0035050 | embryonic heart tube development | 3.14E-02 | 2.37E-03 | 7 | 26 | TestisAburtoni |
| GO:0070588 | calcium ion transmembrane transport | 3.14E-02 | 2.37E-03 | 7 | 26 | TestisAburtoni |
| GO:0001755 | neural crest cell migration | 3.26E-02 | 2.52E-03 | 2 | 15 | TestisAburtoni |
| GO:2001239 | regulation of extrinsic apoptotic signaling pathway in absence of ligand | 3.26E-02 | 2.52E-03 | 2 | 15 | TestisAburtoni |
| GO:0032107 | regulation of response to nutrient levels | 3.26E-02 | 2.52E-03 | 2 | 15 | TestisAburtoni |
| GO:0042698 | ovulation cycle | 3.39E-02 | 2.80E-03 | 3 | 17 | TestisAburtoni |
| GO:0048608 | reproductive structure development | 3.39E-02 | 2.81E-03 | 14 | 37 | TestisAburtoni |
| GO:0044708 | single-organism behavior | 3.39E-02 | 2.87E-03 | 30 | 62 | TestisAburtoni |
| GO:0005978 | glycogen biosynthetic process | 4.01E-02 | 3.59E-03 | 1 | 12 | TestisAburtoni |
| GO:0050806 | positive regulation of synaptic transmission | 4.01E-02 | 3.59E-03 | 1 | 12 | TestisAburtoni |
| GO:0006537 | glutamate biosynthetic process | 4.37E-02 | 4.04E-03 | 0 | 9 | TestisAburtoni |
| GO:0042537 | benzene-containing compound metabolic process | 4.37E-02 | 4.04E-03 | 0 | 9 | TestisAburtoni |
| GO:0035284 | brain segmentation | 4.37E-02 | 4.04E-03 | 0 | 9 | TestisAburtoni |
| GO:0035138 | pectoral fin morphogenesis | 4.37E-02 | 4.04E-03 | 0 | 9 | TestisAburtoni |
| GO:0042219 | cellular modified amino acid catabolic process | 4.37E-02 | 4.04E-03 | 0 | 9 | TestisAburtoni |
| GO:0010456 | cell proliferation in dorsal spinal cord | 4.37E-02 | 4.04E-03 | 0 | 9 | TestisAburtoni |
| GO:0061099 | negative regulation of protein tyrosine kinase activity | 4.37E-02 | 4.04E-03 | 0 | 9 | TestisAburtoni |
| GO:0021778 | oligodendrocyte cell fate specification | 4.37E-02 | 4.04E-03 | 0 | 9 | TestisAburtoni |
| GO:0060445 | branching involved in salivary gland morphogenesis | 4.37E-02 | 4.04E-03 | 0 | 9 | TestisAburtoni |
| GO:0031398 | positive regulation of protein ubiquitination | 4.75E-02 | 4.44E-03 | 2 | 14 | TestisAburtoni |
| GO:0006633 | fatty acid biosynthetic process | 4.75E-02 | 4.44E-03 | 2 | 14 | TestisAburtoni |
| GO:0001508 | regulation of action potential | 4.94E-02 | 4.66E-03 | 11 | 31 | TestisAburtoni |
| GO:0021799 | cerebral cortex radially oriented cell migration | 4.99E-02 | 4.72E-03 | 4 | 18 | TestisAburtoni |

**Enriched in Ovary in *O. ventralis***

| GO-ID | Term | FDR | P-Value | #OTranscripts | #TTranscripts | Enriched in |
|---|---|---|---|---|---|---|
| GO:0008078 | mesodermal cell migration | 1.15E-19 | 4.11E-23 | 75 | 1 | OvaryOventralis |
| GO:0070966 | nuclear-transcribed mRNA catabolic process, no-go decay | 1.19E-07 | 6.42E-10 | 29 | 0 | OvaryOventralis |
| GO:0048268 | clathrin coat assembly | 4.55E-06 | 5.23E-08 | 23 | 0 | OvaryOventralis |
| GO:0043393 | regulation of protein binding | 4.03E-05 | 7.95E-07 | 23 | 1 | OvaryOventralis |
| GO:0008156 | negative regulation of DNA replication | 4.21E-05 | 8.61E-07 | 26 | 2 | OvaryOventralis |
| GO:0090263 | positive regulation of canonical Wnt receptor signaling pathway | 4.50E-05 | 9.76E-07 | 19 | 0 | OvaryOventralis |
| GO:0051835 | positive regulation of synapse structural plasticity | 1.57E-04 | 4.21E-06 | 17 | 0 | OvaryOventralis |
| GO:0046827 | positive regulation of protein export from nucleus | 1.57E-04 | 4.21E-06 | 17 | 0 | OvaryOventralis |
| GO:0060999 | positive regulation of dendritic spine development | 1.57E-04 | 4.21E-06 | 17 | 0 | OvaryOventralis |
| GO:0035023 | regulation of Rho protein signal transduction | 1.90E-04 | 5.30E-06 | 40 | 10 | OvaryOventralis |
| GO:0060690 | epithelial cell differentiation involved in salivary gland development | 2.11E-04 | 6.07E-06 | 23 | 2 | OvaryOventralis |
| GO:0060267 | positive regulation of respiratory burst | 2.81E-04 | 8.74E-06 | 16 | 0 | OvaryOventralis |
| GO:0032793 | positive regulation of CREB transcription factor activity | 2.81E-04 | 8.74E-06 | 16 | 0 | OvaryOventralis |
| GO:0042059 | negative regulation of epidermal growth factor receptor signaling pathway | 2.81E-04 | 8.74E-06 | 16 | 0 | OvaryOventralis |
| GO:0047496 | vesicle transport along microtubule | 3.56E-04 | 1.16E-05 | 22 | 2 | OvaryOventralis |
| GO:0070588 | calcium ion transmembrane transport | 4.50E-04 | 1.56E-05 | 36 | 9 | OvaryOventralis |
| GO:0007435 | salivary gland morphogenesis | 4.76E-04 | 1.70E-05 | 24 | 3 | OvaryOventralis |
| GO:0010259 | multicellular organismal aging | 4.96E-04 | 1.81E-05 | 15 | 0 | OvaryOventralis |
| GO:0043931 | ossification involved in bone maturation | 8.96E-04 | 3.76E-05 | 14 | 0 | OvaryOventralis |
| GO:0003407 | neural retina development | 8.96E-04 | 3.76E-05 | 14 | 0 | OvaryOventralis |
| GO:0090090 | negative regulation of canonical Wnt receptor signaling pathway | 9.21E-04 | 3.90E-05 | 25 | 4 | OvaryOventralis |
| GO:0000076 | DNA replication checkpoint | 1.52E-03 | 7.81E-05 | 13 | 0 | OvaryOventralis |
| GO:0045214 | sarcomere organization | 1.52E-03 | 7.81E-05 | 13 | 0 | OvaryOventralis |
| GO:0032481 | positive regulation of type I interferon production | 1.52E-03 | 7.81E-05 | 13 | 0 | OvaryOventralis |
| GO:0008345 | larval locomotory behavior | 1.52E-03 | 7.81E-05 | 13 | 0 | OvaryOventralis |
| GO:0036342 | post-anal tail morphogenesis | 1.52E-03 | 7.81E-05 | 13 | 0 | OvaryOventralis |
| GO:0090023 | positive regulation of neutrophil chemotaxis | 1.75E-03 | 9.38E-05 | 16 | 1 | OvaryOventralis |
| GO:0050766 | positive regulation of phagocytosis | 1.75E-03 | 9.38E-05 | 16 | 1 | OvaryOventralis |
| GO:0016579 | protein deubiquitination | 1.93E-03 | 1.05E-04 | 21 | 3 | OvaryOventralis |
| GO:0043200 | response to amino acid stimulus | 2.22E-03 | 1.24E-04 | 23 | 4 | OvaryOventralis |
| GO:0016340 | calcium-dependent cell-matrix adhesion | 2.64E-03 | 1.62E-04 | 12 | 0 | OvaryOventralis |
| GO:0045176 | apical protein localization | 2.64E-03 | 1.62E-04 | 12 | 0 | OvaryOventralis |
| GO:0006400 | tRNA modification | 2.64E-03 | 1.62E-04 | 12 | 0 | OvaryOventralis |
| GO:0060832 | oocyte animal/vegetal axis specification | 2.64E-03 | 1.62E-04 | 12 | 0 | OvaryOventralis |
| GO:0050732 | negative regulation of peptidyl-tyrosine phosphorylation | 2.93E-03 | 1.84E-04 | 15 | 1 | OvaryOventralis |
| GO:0022011 | myelination in peripheral nervous system | 4.70E-03 | 3.35E-04 | 11 | 0 | OvaryOventralis |
| GO:0051938 | L-glutamate import | 4.70E-03 | 3.35E-04 | 11 | 0 | OvaryOventralis |
| GO:0033126 | positive regulation of GTP catabolic process | 4.70E-03 | 3.35E-04 | 11 | 0 | OvaryOventralis |
| GO:0070779 | D-aspartate import | 4.70E-03 | 3.35E-04 | 11 | 0 | OvaryOventralis |
| GO:0090307 | spindle assembly involved in mitosis | 4.70E-03 | 3.35E-04 | 11 | 0 | OvaryOventralis |

| | | | | | | |
|---|---|---|---|---|---|---|
| GO:0000724 | double-strand break repair via homologous recombination | 4.70E-03 | 3.35E-04 | 11 | 0 | OvaryOventralis |
| GO:0045736 | negative regulation of cyclin-dependent protein serine/threonine kinase activity | 4.70E-03 | 3.35E-04 | 11 | 0 | OvaryOventralis |
| GO:0000381 | regulation of alternative mRNA splicing, via spliceosome | 4.70E-03 | 3.35E-04 | 11 | 0 | OvaryOventralis |
| GO:0022027 | interkinetic nuclear migration | 4.94E-03 | 3.58E-04 | 14 | 1 | OvaryOventralis |
| GO:0006323 | DNA packaging | 4.94E-03 | 3.58E-04 | 14 | 1 | OvaryOventralis |
| GO:0010976 | positive regulation of neuron projection development | 5.45E-03 | 4.00E-04 | 23 | 5 | OvaryOventralis |
| GO:0045944 | positive regulation of transcription from RNA polymerase II promoter | 7.69E-03 | 6.01E-04 | 78 | 44 | OvaryOventralis |
| GO:0060119 | inner ear receptor cell development | 7.86E-03 | 6.20E-04 | 18 | 3 | OvaryOventralis |
| GO:0048814 | regulation of dendrite morphogenesis | 7.86E-03 | 6.20E-04 | 18 | 3 | OvaryOventralis |
| GO:0001843 | neural tube closure | 8.23E-03 | 6.71E-04 | 20 | 4 | OvaryOventralis |
| GO:0006888 | ER to Golgi vesicle-mediated transport | 8.23E-03 | 6.95E-04 | 10 | 0 | OvaryOventralis |
| GO:0043297 | apical junction assembly | 8.23E-03 | 6.95E-04 | 10 | 0 | OvaryOventralis |
| GO:0007199 | G-protein coupled receptor signaling pathway coupled to cGMP nucleotide second messenger | 8.23E-03 | 6.95E-04 | 10 | 0 | OvaryOventralis |
| GO:0071679 | commissural neuron axon guidance | 8.23E-03 | 6.95E-04 | 10 | 0 | OvaryOventralis |
| GO:0060026 | convergent extension | 8.23E-03 | 6.95E-04 | 10 | 0 | OvaryOventralis |
| GO:0033278 | cell proliferation in midbrain | 8.23E-03 | 6.95E-04 | 10 | 0 | OvaryOventralis |
| GO:0014036 | neural crest cell fate specification | 8.23E-03 | 6.95E-04 | 10 | 0 | OvaryOventralis |
| GO:0030241 | skeletal muscle myosin thick filament assembly | 8.23E-03 | 6.95E-04 | 10 | 0 | OvaryOventralis |
| GO:0051491 | positive regulation of filopodium assembly | 8.23E-03 | 6.95E-04 | 10 | 0 | OvaryOventralis |
| GO:0032237 | activation of store-operated calcium channel activity | 8.23E-03 | 6.95E-04 | 10 | 0 | OvaryOventralis |
| GO:0070486 | leukocyte aggregation | 8.23E-03 | 6.95E-04 | 10 | 0 | OvaryOventralis |
| GO:0021754 | facial nucleus development | 8.23E-03 | 6.95E-04 | 10 | 0 | OvaryOventralis |
| GO:0060307 | regulation of ventricular cardiac muscle cell membrane repolarization | 8.23E-03 | 6.95E-04 | 10 | 0 | OvaryOventralis |
| GO:0030953 | astral microtubule organization | 8.23E-03 | 6.96E-04 | 13 | 1 | OvaryOventralis |
| GO:0006103 | 2-oxoglutarate metabolic process | 8.23E-03 | 6.96E-04 | 13 | 1 | OvaryOventralis |
| GO:0006102 | isocitrate metabolic process | 8.23E-03 | 6.96E-04 | 13 | 1 | OvaryOventralis |
| GO:0010948 | negative regulation of cell cycle process | 9.20E-03 | 7.85E-04 | 41 | 17 | OvaryOventralis |
| GO:0006099 | tricarboxylic acid cycle | 1.10E-02 | 9.59E-04 | 15 | 2 | OvaryOventralis |
| GO:0019886 | antigen processing and presentation of exogenous peptide antigen via MHC class II | 1.12E-02 | 1.03E-03 | 25 | 7 | OvaryOventralis |
| GO:0090068 | positive regulation of cell cycle process | 1.24E-02 | 1.16E-03 | 21 | 5 | OvaryOventralis |
| GO:0071496 | cellular response to external stimulus | 1.39E-02 | 1.30E-03 | 27 | 9 | OvaryOventralis |
| GO:0038096 | Fc-gamma receptor signaling pathway involved in phagocytosis | 1.40E-02 | 1.35E-03 | 12 | 1 | OvaryOventralis |
| GO:0043409 | negative regulation of MAPK cascade | 1.40E-02 | 1.35E-03 | 12 | 1 | OvaryOventralis |
| GO:0019370 | leukotriene biosynthetic process | 1.40E-02 | 1.35E-03 | 12 | 1 | OvaryOventralis |
| GO:0048261 | negative regulation of receptor-mediated endocytosis | 1.40E-02 | 1.35E-03 | 12 | 1 | OvaryOventralis |
| GO:0030917 | midbrain-hindbrain boundary development | 1.40E-02 | 1.44E-03 | 9 | 0 | OvaryOventralis |
| GO:0007274 | neuromuscular synaptic transmission | 1.40E-02 | 1.44E-03 | 9 | 0 | OvaryOventralis |
| GO:0002115 | store-operated calcium entry | 1.40E-02 | 1.44E-03 | 9 | 0 | OvaryOventralis |
| GO:0042267 | natural killer cell mediated cytotoxicity | 1.40E-02 | 1.44E-03 | 9 | 0 | OvaryOventralis |
| GO:0034644 | cellular response to UV | 1.40E-02 | 1.44E-03 | 9 | 0 | OvaryOventralis |
| GO:0010693 | negative regulation of alkaline phosphatase activity | 1.40E-02 | 1.44E-03 | 9 | 0 | OvaryOventralis |
| GO:0002924 | negative regulation of humoral immune response mediated by circulating immunoglobulin | 1.40E-02 | 1.44E-03 | 9 | 0 | OvaryOventralis |
| GO:0048672 | positive regulation of collateral sprouting | 1.40E-02 | 1.44E-03 | 9 | 0 | OvaryOventralis |
| GO:0034453 | microtubule anchoring | 1.40E-02 | 1.44E-03 | 9 | 0 | OvaryOventralis |
| GO:0050860 | negative regulation of T cell receptor signaling pathway | 1.40E-02 | 1.44E-03 | 9 | 0 | OvaryOventralis |
| GO:1902083 | negative regulation of peptidyl-cysteine S-nitrosylation | 1.40E-02 | 1.44E-03 | 9 | 0 | OvaryOventralis |
| GO:0003117 | regulation of vasoconstriction by circulating norepinephrine | 1.40E-02 | 1.44E-03 | 9 | 0 | OvaryOventralis |
| GO:0006998 | nuclear envelope organization | 1.40E-02 | 1.44E-03 | 9 | 0 | OvaryOventralis |
| GO:0030032 | lamellipodium assembly | 1.81E-02 | 1.94E-03 | 20 | 5 | OvaryOventralis |
| GO:0043065 | positive regulation of apoptotic process | 1.81E-02 | 1.98E-03 | 52 | 27 | OvaryOventralis |
| GO:0060042 | retina morphogenesis in camera-type eye | 1.81E-02 | 2.01E-03 | 18 | 4 | OvaryOventralis |
| GO:0007605 | sensory perception of sound | 1.84E-02 | 2.06E-03 | 26 | 9 | OvaryOventralis |
| GO:0060541 | respiratory system development | 2.11E-02 | 2.38E-03 | 25 | 8 | OvaryOventralis |
| GO:0000122 | negative regulation of transcription from RNA polymerase II promoter | 2.11E-02 | 2.39E-03 | 64 | 37 | OvaryOventralis |
| GO:0042130 | negative regulation of T cell proliferation | 2.27E-02 | 2.60E-03 | 11 | 1 | OvaryOventralis |
| GO:0042472 | inner ear morphogenesis | 2.40E-02 | 2.98E-03 | 21 | 6 | OvaryOventralis |
| GO:0043388 | positive regulation of DNA binding | 2.40E-02 | 2.98E-03 | 8 | 0 | OvaryOventralis |
| GO:0042759 | long-chain fatty acid biosynthetic process | 2.40E-02 | 2.98E-03 | 8 | 0 | OvaryOventralis |
| GO:0048026 | positive regulation of mRNA splicing, via spliceosome | 2.40E-02 | 2.98E-03 | 8 | 0 | OvaryOventralis |
| GO:0048007 | antigen processing and presentation, exogenous lipid antigen via MHC class Ib | 2.40E-02 | 2.98E-03 | 8 | 0 | OvaryOventralis |
| GO:0043154 | negative regulation of cysteine-type endopeptidase activity involved in apoptotic process | 2.40E-02 | 2.98E-03 | 8 | 0 | OvaryOventralis |
| GO:0015937 | coenzyme A biosynthetic process | 2.40E-02 | 2.98E-03 | 8 | 0 | OvaryOventralis |
| GO:0060071 | Wnt receptor signaling pathway, planar cell polarity pathway | 2.40E-02 | 2.98E-03 | 8 | 0 | OvaryOventralis |
| GO:0007063 | regulation of sister chromatid cohesion | 2.40E-02 | 2.98E-03 | 8 | 0 | OvaryOventralis |
| GO:0047484 | regulation of response to osmotic stress | 2.40E-02 | 2.98E-03 | 8 | 0 | OvaryOventralis |
| GO:0015871 | choline transport | 2.40E-02 | 2.98E-03 | 8 | 0 | OvaryOventralis |
| GO:0071386 | cellular response to corticosterone stimulus | 2.40E-02 | 2.98E-03 | 8 | 0 | OvaryOventralis |
| GO:0048840 | otolith development | 2.40E-02 | 2.98E-03 | 8 | 0 | OvaryOventralis |
| GO:0032456 | endocytic recycling | 2.40E-02 | 2.98E-03 | 8 | 0 | OvaryOventralis |
| GO:0043983 | histone H4-K12 acetylation | 2.40E-02 | 2.98E-03 | 8 | 0 | OvaryOventralis |
| GO:0060855 | venous endothelial cell migration involved in lymph vessel development | 2.40E-02 | 2.98E-03 | 8 | 0 | OvaryOventralis |
| GO:0060854 | patterning of lymph vessels | 2.40E-02 | 2.98E-03 | 8 | 0 | OvaryOventralis |
| GO:0008292 | acetylcholine biosynthetic process | 2.40E-02 | 2.98E-03 | 8 | 0 | OvaryOventralis |
| GO:0042066 | perineurial glial growth | 2.40E-02 | 2.98E-03 | 8 | 0 | OvaryOventralis |
| GO:0051351 | positive regulation of ligase activity | 2.40E-02 | 2.98E-03 | 8 | 0 | OvaryOventralis |
| GO:1901660 | calcium ion export | 2.40E-02 | 2.98E-03 | 8 | 0 | OvaryOventralis |
| GO:0044154 | histone H3-K14 acetylation | 2.40E-02 | 2.98E-03 | 8 | 0 | OvaryOventralis |
| GO:0061088 | regulation of sequestering of zinc ion | 2.40E-02 | 2.98E-03 | 8 | 0 | OvaryOventralis |
| GO:0010389 | regulation of G2/M transition of mitotic cell cycle | 2.40E-02 | 2.98E-03 | 8 | 0 | OvaryOventralis |
| GO:0045814 | negative regulation of gene expression, epigenetic | 2.40E-02 | 2.98E-03 | 8 | 0 | OvaryOventralis |
| GO:0048499 | synaptic vesicle membrane organization | 2.40E-02 | 2.98E-03 | 8 | 0 | OvaryOventralis |
| GO:0051138 | positive regulation of NK T cell differentiation | 2.40E-02 | 2.98E-03 | 8 | 0 | OvaryOventralis |
| GO:0031443 | fast-twitch skeletal muscle fiber contraction | 2.40E-02 | 2.98E-03 | 8 | 0 | OvaryOventralis |
| GO:0030072 | peptide hormone secretion | 3.16E-02 | 4.19E-03 | 26 | 10 | OvaryOventralis |
| GO:0030048 | actin filament-based movement | 3.16E-02 | 4.19E-03 | 26 | 10 | OvaryOventralis |
| GO:0043414 | macromolecule methylation | 3.21E-02 | 4.28E-03 | 22 | 7 | OvaryOventralis |
| GO:0006511 | ubiquitin-dependent protein catabolic process | 3.23E-02 | 4.31E-03 | 59 | 34 | OvaryOventralis |
| GO:0034504 | protein localization to nucleus | 3.55E-02 | 4.83E-03 | 20 | 6 | OvaryOventralis |
| GO:0065004 | protein-DNA complex assembly | 3.58E-02 | 4.98E-03 | 10 | 1 | OvaryOventralis |
| GO:0046326 | positive regulation of glucose import | 3.58E-02 | 4.98E-03 | 10 | 1 | OvaryOventralis |
| GO:0031577 | spindle checkpoint | 3.58E-02 | 4.98E-03 | 10 | 1 | OvaryOventralis |
| GO:0051149 | positive regulation of muscle cell differentiation | 3.81E-02 | 5.37E-03 | 18 | 5 | OvaryOventralis |
| GO:0048919 | posterior lateral line neuromast development | 4.10E-02 | 5.87E-03 | 12 | 2 | OvaryOventralis |
| GO:0007088 | regulation of mitosis | 4.10E-02 | 6.05E-03 | 14 | 3 | OvaryOventralis |
| GO:0033590 | response to cobalamin | 4.10E-02 | 6.18E-03 | 7 | 0 | OvaryOventralis |
| GO:0045494 | photoreceptor cell maintenance | 4.10E-02 | 6.18E-03 | 7 | 0 | OvaryOventralis |
| GO:0032968 | positive regulation of transcription elongation from RNA polymerase II promoter | 4.10E-02 | 6.18E-03 | 7 | 0 | OvaryOventralis |
| GO:0006582 | melanin metabolic process | 4.10E-02 | 6.18E-03 | 7 | 0 | OvaryOventralis |
| GO:0010961 | cellular magnesium ion homeostasis | 4.10E-02 | 6.18E-03 | 7 | 0 | OvaryOventralis |
| GO:0010894 | negative regulation of steroid biosynthetic process | 4.10E-02 | 6.18E-03 | 7 | 0 | OvaryOventralis |
| GO:0075713 | establishment of integrated proviral latency | 4.10E-02 | 6.18E-03 | 7 | 0 | OvaryOventralis |
| GO:0006398 | histone mRNA 3'-end processing | 4.10E-02 | 6.18E-03 | 7 | 0 | OvaryOventralis |
| GO:0018345 | protein palmitoylation | 4.10E-02 | 6.18E-03 | 7 | 0 | OvaryOventralis |
| GO:0006383 | transcription from RNA polymerase III promoter | 4.10E-02 | 6.18E-03 | 7 | 0 | OvaryOventralis |
| GO:0006379 | mRNA cleavage | 4.10E-02 | 6.18E-03 | 7 | 0 | OvaryOventralis |

| | | | | | | |
|---|---|---|---|---|---|---|
| GO:0006378 | mRNA polyadenylation | 4.10E-02 | 6.18E-03 | 7 | 0 | OvaryOventralis |
| GO:0035278 | negative regulation of translation involved in gene silencing by miRNA | 4.10E-02 | 6.18E-03 | 7 | 0 | OvaryOventralis |
| GO:0051694 | pointed-end actin filament capping | 4.10E-02 | 6.18E-03 | 7 | 0 | OvaryOventralis |
| GO:0070782 | phosphatidylserine exposure on apoptotic cell surface | 4.10E-02 | 6.18E-03 | 7 | 0 | OvaryOventralis |
| GO:0090329 | regulation of DNA-dependent DNA replication | 4.10E-02 | 6.18E-03 | 7 | 0 | OvaryOventralis |
| GO:0048845 | venous blood vessel morphogenesis | 4.10E-02 | 6.18E-03 | 7 | 0 | OvaryOventralis |
| GO:0044335 | canonical Wnt receptor signaling pathway involved in neural crest cell differentiation | 4.10E-02 | 6.18E-03 | 7 | 0 | OvaryOventralis |
| GO:0050996 | positive regulation of lipid catabolic process | 4.10E-02 | 6.18E-03 | 7 | 0 | OvaryOventralis |
| GO:0031572 | G2 DNA damage checkpoint | 4.10E-02 | 6.18E-03 | 7 | 0 | OvaryOventralis |
| GO:1901407 | regulation of phosphorylation of RNA polymerase II C-terminal domain | 4.10E-02 | 6.18E-03 | 7 | 0 | OvaryOventralis |
| GO:0000395 | mRNA 5'-splice site recognition | 4.10E-02 | 6.18E-03 | 7 | 0 | OvaryOventralis |
| GO:0045622 | regulation of T-helper cell differentiation | 4.10E-02 | 6.18E-03 | 7 | 0 | OvaryOventralis |
| | | | | | | |
| **Enriched in Testis in *O. ventralis*** | | | | | | |
| GO:0070301 | cellular response to hydrogen peroxide | 7.06E-07 | 5.57E-09 | 1 | 33 | TestisOventralis |
| GO:0072661 | protein targeting to plasma membrane | 1.92E-06 | 1.88E-08 | 0 | 27 | TestisOventralis |
| GO:0045838 | positive regulation of membrane potential | 1.92E-06 | 1.88E-08 | 0 | 27 | TestisOventralis |
| GO:0010765 | positive regulation of sodium ion transport | 3.40E-06 | 3.71E-08 | 0 | 26 | TestisOventralis |
| GO:0045760 | positive regulation of action potential | 5.84E-06 | 7.30E-08 | 0 | 25 | TestisOventralis |
| GO:0043266 | regulation of potassium ion transport | 9.40E-06 | 1.41E-07 | 1 | 28 | TestisOventralis |
| GO:1900827 | positive regulation of membrane depolarization involved in regulation of cardiac muscle cell action potential | 9.40E-06 | 1.44E-07 | 0 | 24 | TestisOventralis |
| GO:0000281 | mitotic cytokinesis | 9.40E-06 | 1.44E-07 | 0 | 24 | TestisOventralis |
| GO:0019233 | sensory perception of pain | 9.40E-06 | 1.44E-07 | 0 | 24 | TestisOventralis |
| GO:2000651 | positive regulation of sodium ion transmembrane transporter activity | 9.40E-06 | 1.44E-07 | 0 | 24 | TestisOventralis |
| GO:0090314 | positive regulation of protein targeting to membrane | 9.40E-06 | 1.44E-07 | 0 | 24 | TestisOventralis |
| GO:0010650 | positive regulation of cell communication by electrical coupling | 9.40E-06 | 1.44E-07 | 0 | 24 | TestisOventralis |
| GO:0072660 | maintenance of protein location in plasma membrane | 9.40E-06 | 1.44E-07 | 0 | 24 | TestisOventralis |
| GO:0034112 | positive regulation of homotypic cell-cell adhesion | 9.40E-06 | 1.44E-07 | 0 | 24 | TestisOventralis |
| GO:0008209 | androgen metabolic process | 1.57E-05 | 2.69E-07 | 1 | 27 | TestisOventralis |
| GO:0031529 | ruffle organization | 1.57E-05 | 2.69E-07 | 1 | 27 | TestisOventralis |
| GO:0008210 | estrogen metabolic process | 2.70E-05 | 5.11E-07 | 1 | 26 | TestisOventralis |
| GO:0045662 | negative regulation of myoblast differentiation | 2.91E-05 | 5.60E-07 | 0 | 22 | TestisOventralis |
| GO:0033327 | Leydig cell differentiation | 4.50E-05 | 9.72E-07 | 1 | 25 | TestisOventralis |
| GO:0001553 | luteinization | 4.50E-05 | 9.72E-07 | 1 | 25 | TestisOventralis |
| GO:0060291 | long-term synaptic potentiation | 1.59E-04 | 4.31E-06 | 0 | 19 | TestisOventralis |
| GO:0051044 | positive regulation of membrane protein ectodomain proteolysis | 1.59E-04 | 4.31E-06 | 0 | 19 | TestisOventralis |
| GO:0009791 | post-embryonic development | 2.17E-04 | 6.30E-06 | 7 | 38 | TestisOventralis |
| GO:0051898 | negative regulation of protein kinase B signaling cascade | 2.28E-04 | 6.73E-06 | 2 | 25 | TestisOventralis |
| GO:0048008 | platelet-derived growth factor receptor signaling pathway | 2.58E-04 | 7.68E-06 | 4 | 30 | TestisOventralis |
| GO:0043627 | response to estrogen stimulus | 3.17E-04 | 1.02E-05 | 3 | 27 | TestisOventralis |
| GO:0045766 | positive regulation of angiogenesis | 3.17E-04 | 1.02E-05 | 3 | 27 | TestisOventralis |
| GO:0032851 | positive regulation of Rab GTPase activity | 3.71E-04 | 1.26E-05 | 1 | 21 | TestisOventralis |
| GO:0031100 | organ regeneration | 4.76E-04 | 1.68E-05 | 0 | 17 | TestisOventralis |
| GO:0032486 | Rap protein signal transduction | 4.76E-04 | 1.68E-05 | 0 | 17 | TestisOventralis |
| GO:0060325 | face morphogenesis | 6.06E-04 | 2.34E-05 | 4 | 28 | TestisOventralis |
| GO:0051549 | positive regulation of keratinocyte migration | 8.21E-04 | 3.33E-05 | 0 | 16 | TestisOventralis |
| GO:0035270 | endocrine system development | 1.20E-03 | 5.71E-05 | 3 | 24 | TestisOventralis |
| GO:0007193 | adenylate cyclase-inhibiting G-protein coupled receptor signaling pathway | 1.61E-03 | 8.42E-05 | 1 | 18 | TestisOventralis |
| GO:0048661 | positive regulation of smooth muscle cell proliferation | 1.61E-03 | 8.42E-05 | 1 | 18 | TestisOventralis |
| GO:0060292 | long term synaptic depression | 2.28E-03 | 1.31E-04 | 0 | 14 | TestisOventralis |
| GO:0021516 | dorsal spinal cord development | 2.28E-03 | 1.31E-04 | 0 | 14 | TestisOventralis |
| GO:0048146 | positive regulation of fibroblast proliferation | 2.28E-03 | 1.31E-04 | 0 | 14 | TestisOventralis |
| GO:0000186 | activation of MAPKK activity | 2.28E-03 | 1.31E-04 | 0 | 14 | TestisOventralis |
| GO:0070654 | sensory epithelium regeneration | 2.28E-03 | 1.31E-04 | 0 | 14 | TestisOventralis |
| GO:0046599 | regulation of centriole replication | 2.28E-03 | 1.31E-04 | 0 | 14 | TestisOventralis |
| GO:0051897 | positive regulation of protein kinase B signaling cascade | 2.34E-03 | 1.35E-04 | 2 | 20 | TestisOventralis |
| GO:0030574 | collagen catabolic process | 2.64E-03 | 1.58E-04 | 1 | 17 | TestisOventralis |
| GO:0045907 | positive regulation of vasoconstriction | 2.64E-03 | 1.58E-04 | 1 | 17 | TestisOventralis |
| GO:0008037 | cell recognition | 2.64E-03 | 1.58E-04 | 1 | 17 | TestisOventralis |
| GO:0050714 | positive regulation of protein secretion | 2.64E-03 | 1.58E-04 | 1 | 17 | TestisOventralis |
| GO:0007229 | integrin-mediated signaling pathway | 3.47E-03 | 2.21E-04 | 7 | 31 | TestisOventralis |
| GO:0070098 | chemokine-mediated signaling pathway | 3.92E-03 | 2.59E-04 | 0 | 13 | TestisOventralis |
| GO:0009435 | NAD biosynthetic process | 3.92E-03 | 2.59E-04 | 0 | 13 | TestisOventralis |
| GO:0006206 | pyrimidine nucleobase metabolic process | 3.92E-03 | 2.59E-04 | 0 | 13 | TestisOventralis |
| GO:0006067 | ethanol metabolic process | 3.92E-03 | 2.59E-04 | 0 | 13 | TestisOventralis |
| GO:0050667 | homocysteine metabolic process | 3.92E-03 | 2.59E-04 | 0 | 13 | TestisOventralis |
| GO:0090303 | positive regulation of wound healing | 4.40E-03 | 2.97E-04 | 1 | 16 | TestisOventralis |
| GO:0045665 | negative regulation of neuron differentiation | 4.40E-03 | 2.97E-04 | 1 | 16 | TestisOventralis |
| GO:0050906 | detection of stimulus involved in sensory perception | 4.91E-03 | 3.52E-04 | 4 | 23 | TestisOventralis |
| GO:0010595 | positive regulation of endothelial cell migration | 6.74E-03 | 5.13E-04 | 0 | 12 | TestisOventralis |
| GO:0006070 | octanol metabolic process | 6.74E-03 | 5.13E-04 | 0 | 12 | TestisOventralis |
| GO:0046292 | formaldehyde metabolic process | 6.74E-03 | 5.13E-04 | 0 | 12 | TestisOventralis |
| GO:0046135 | pyrimidine nucleoside catabolic process | 6.74E-03 | 5.13E-04 | 0 | 12 | TestisOventralis |
| GO:0001890 | placenta development | 7.17E-03 | 5.55E-04 | 1 | 15 | TestisOventralis |
| GO:0048663 | neuron fate commitment | 7.17E-03 | 5.55E-04 | 1 | 15 | TestisOventralis |
| GO:0071222 | cellular response to lipopolysaccharide | 7.86E-03 | 6.19E-04 | 5 | 24 | TestisOventralis |
| GO:0030522 | intracellular receptor signaling pathway | 8.23E-03 | 6.76E-04 | 13 | 39 | TestisOventralis |
| GO:0014910 | regulation of smooth muscle cell migration | 9.20E-03 | 7.88E-04 | 2 | 17 | TestisOventralis |
| GO:0006767 | water-soluble vitamin metabolic process | 1.11E-02 | 1.01E-03 | 4 | 21 | TestisOventralis |
| GO:0010042 | response to manganese ion | 1.11E-02 | 1.02E-03 | 0 | 11 | TestisOventralis |
| GO:0007176 | regulation of epidermal growth factor-activated receptor activity | 1.11E-02 | 1.02E-03 | 0 | 11 | TestisOventralis |
| GO:0007129 | synapsis | 1.11E-02 | 1.02E-03 | 0 | 11 | TestisOventralis |
| GO:0043094 | cellular metabolic compound salvage | 1.11E-02 | 1.02E-03 | 0 | 11 | TestisOventralis |
| GO:0042312 | regulation of vasodilation | 1.11E-02 | 1.02E-03 | 0 | 11 | TestisOventralis |
| GO:0030199 | collagen fibril organization | 1.11E-02 | 1.02E-03 | 0 | 11 | TestisOventralis |
| GO:2000377 | regulation of reactive oxygen species metabolic process | 1.11E-02 | 1.02E-03 | 0 | 11 | TestisOventralis |
| GO:0034241 | positive regulation of macrophage fusion | 1.11E-02 | 1.02E-03 | 0 | 11 | TestisOventralis |
| GO:0035999 | tetrahydrofolate interconversion | 1.11E-02 | 1.02E-03 | 0 | 11 | TestisOventralis |
| GO:0035965 | cardiolipin acyl-chain remodeling | 1.11E-02 | 1.02E-03 | 0 | 11 | TestisOventralis |
| GO:0021879 | forebrain neuron differentiation | 1.12E-02 | 1.04E-03 | 1 | 14 | TestisOventralis |
| GO:0022617 | extracellular matrix disassembly | 1.45E-02 | 1.50E-03 | 7 | 27 | TestisOventralis |
| GO:0008344 | adult locomotory behavior | 1.55E-02 | 1.61E-03 | 3 | 18 | TestisOventralis |
| GO:0021536 | diencephalon development | 1.60E-02 | 1.67E-03 | 5 | 22 | TestisOventralis |
| GO:0048771 | tissue remodeling | 1.61E-02 | 1.69E-03 | 4 | 20 | TestisOventralis |
| GO:0021954 | central nervous system neuron development | 1.61E-02 | 1.69E-03 | 4 | 20 | TestisOventralis |
| GO:0006801 | superoxide metabolic process | 1.81E-02 | 1.92E-03 | 1 | 13 | TestisOventralis |
| GO:0007141 | male meiosis I | 1.81E-02 | 2.02E-03 | 0 | 10 | TestisOventralis |
| GO:0007131 | reciprocal meiotic recombination | 1.81E-02 | 2.02E-03 | 0 | 10 | TestisOventralis |
| GO:0030517 | negative regulation of axon extension | 1.81E-02 | 2.02E-03 | 0 | 10 | TestisOventralis |
| GO:0009086 | methionine biosynthetic process | 1.81E-02 | 2.02E-03 | 0 | 10 | TestisOventralis |
| GO:0010761 | fibroblast migration | 1.81E-02 | 2.02E-03 | 0 | 10 | TestisOventralis |
| GO:0048841 | regulation of axon extension involved in axon guidance | 1.81E-02 | 2.02E-03 | 0 | 10 | TestisOventralis |
| GO:0006040 | amino sugar metabolic process | 1.81E-02 | 2.02E-03 | 0 | 10 | TestisOventralis |
| GO:0050919 | negative chemotaxis | 1.81E-02 | 2.02E-03 | 0 | 10 | TestisOventralis |
| GO:0033692 | cellular polysaccharide biosynthetic process | 1.81E-02 | 2.02E-03 | 0 | 10 | TestisOventralis |

| | | | | | | |
|---|---|---|---|---|---|---|
| GO:0007178 | transmembrane receptor protein serine/threonine kinase signaling pathway | 1.85E-02 | 2.07E-03 | 11 | 33 | TestisOventralis |
| GO:0055074 | calcium ion homeostasis | 2.11E-02 | 2.39E-03 | 17 | 42 | TestisOventralis |
| GO:0006661 | phosphatidylinositol biosynthetic process | 2.25E-02 | 2.56E-03 | 6 | 23 | TestisOventralis |
| GO:0031532 | actin cytoskeleton reorganization | 2.39E-02 | 2.76E-03 | 3 | 17 | TestisOventralis |
| GO:0007569 | cell aging | 2.40E-02 | 2.81E-03 | 4 | 19 | TestisOventralis |
| GO:0009168 | purine ribonucleoside monophosphate biosynthetic process | 2.78E-02 | 3.56E-03 | 1 | 12 | TestisOventralis |
| GO:0033630 | positive regulation of cell adhesion mediated by integrin | 2.78E-02 | 3.56E-03 | 1 | 12 | TestisOventralis |
| GO:0009201 | ribonucleoside triphosphate biosynthetic process | 3.05E-02 | 4.01E-03 | 0 | 9 | TestisOventralis |
| GO:0038032 | termination of G-protein coupled receptor signaling pathway | 3.05E-02 | 4.01E-03 | 0 | 9 | TestisOventralis |
| GO:0002087 | regulation of respiratory gaseous exchange by neurological system process | 3.05E-02 | 4.01E-03 | 0 | 9 | TestisOventralis |
| GO:0071322 | cellular response to carbohydrate stimulus | 3.05E-02 | 4.01E-03 | 0 | 9 | TestisOventralis |
| GO:2000352 | negative regulation of endothelial cell apoptotic process | 3.05E-02 | 4.01E-03 | 0 | 9 | TestisOventralis |
| GO:0008298 | intracellular mRNA localization | 3.05E-02 | 4.01E-03 | 0 | 9 | TestisOventralis |
| GO:0050951 | sensory perception of temperature stimulus | 3.05E-02 | 4.01E-03 | 0 | 9 | TestisOventralis |
| GO:0050922 | negative regulation of chemotaxis | 3.05E-02 | 4.01E-03 | 0 | 9 | TestisOventralis |
| GO:0034446 | substrate adhesion-dependent cell spreading | 3.05E-02 | 4.01E-03 | 0 | 9 | TestisOventralis |
| GO:0050849 | negative regulation of calcium-mediated signaling | 3.05E-02 | 4.01E-03 | 0 | 9 | TestisOventralis |
| GO:0032148 | activation of protein kinase B activity | 3.05E-02 | 4.01E-03 | 0 | 9 | TestisOventralis |
| GO:0007520 | myoblast fusion | 3.05E-02 | 4.01E-03 | 0 | 9 | TestisOventralis |
| GO:0045761 | regulation of adenylate cyclase activity | 3.05E-02 | 4.01E-03 | 0 | 9 | TestisOventralis |
| GO:0045685 | regulation of glial cell differentiation | 3.05E-02 | 4.01E-03 | 0 | 9 | TestisOventralis |
| GO:0006940 | regulation of smooth muscle contraction | 3.05E-02 | 4.01E-03 | 0 | 9 | TestisOventralis |
| GO:0001935 | endothelial cell proliferation | 3.28E-02 | 4.40E-03 | 2 | 14 | TestisOventralis |
| GO:0035094 | response to nicotine | 3.28E-02 | 4.40E-03 | 2 | 14 | TestisOventralis |
| GO:0009755 | hormone-mediated signaling pathway | 3.48E-02 | 4.70E-03 | 3 | 16 | TestisOventralis |
| GO:0051054 | positive regulation of DNA metabolic process | 3.48E-02 | 4.70E-03 | 3 | 16 | TestisOventralis |
| GO:0048705 | skeletal system morphogenesis | 3.55E-02 | 4.85E-03 | 17 | 40 | TestisOventralis |
| GO:0071466 | cellular response to xenobiotic stimulus | 3.60E-02 | 5.02E-03 | 8 | 26 | TestisOventralis |
| GO:0043406 | positive regulation of MAP kinase activity | 3.60E-02 | 5.02E-03 | 8 | 26 | TestisOventralis |
| GO:0071709 | membrane assembly | 3.89E-02 | 5.50E-03 | 10 | 29 | TestisOventralis |
| GO:0043001 | Golgi to plasma membrane protein transport | 3.89E-02 | 5.50E-03 | 10 | 29 | TestisOventralis |
| GO:0007528 | neuromuscular junction development | 3.89E-02 | 5.50E-03 | 10 | 29 | TestisOventralis |
| GO:0036293 | response to decreased oxygen levels | 4.02E-02 | 5.68E-03 | 18 | 41 | TestisOventralis |
| GO:0009615 | response to virus | 4.26E-02 | 6.42E-03 | 6 | 21 | TestisOventralis |
| GO:0048169 | regulation of long-term neuronal synaptic plasticity | 4.33E-02 | 6.57E-03 | 1 | 11 | TestisOventralis |
| GO:0015701 | bicarbonate transport | 4.33E-02 | 6.57E-03 | 1 | 11 | TestisOventralis |
| GO:0042398 | cellular modified amino acid biosynthetic process | 4.33E-02 | 6.57E-03 | 1 | 11 | TestisOventralis |
| GO:0060872 | semicircular canal development | 4.33E-02 | 6.57E-03 | 1 | 11 | TestisOventralis |
| GO:0070613 | regulation of protein processing | 4.33E-02 | 6.57E-03 | 1 | 11 | TestisOventralis |
| GO:0042117 | monocyte activation | 4.33E-02 | 6.57E-03 | 1 | 11 | TestisOventralis |
| GO:0051088 | PMA-inducible membrane protein ectodomain proteolysis | 4.33E-02 | 6.57E-03 | 1 | 11 | TestisOventralis |
| GO:0006906 | vesicle fusion | 4.33E-02 | 6.57E-03 | 1 | 11 | TestisOventralis |
| GO:0005996 | monosaccharide metabolic process | 4.44E-02 | 6.75E-03 | 27 | 54 | TestisOventralis |
| GO:0007612 | learning | 4.99E-02 | 7.64E-03 | 4 | 17 | TestisOventralis |

**Enriched in Ovary in *E. cyanostictus***

| GO-ID | Term | FDR | P-Value | #Otranscripts | #TTranscripts | Enriched in |
|---|---|---|---|---|---|---|
| GO:0032854 | positive regulation of Rap GTPase activity | 5.80E-10 | 1.86E-12 | 32 | 0 | OvaryEcyanostictus |
| GO:0000186 | activation of MAPKK activity | 3.24E-07 | 1.73E-09 | 30 | 2 | OvaryEcyanostictus |
| GO:0038180 | nerve growth factor signaling pathway | 6.52E-07 | 3.80E-09 | 29 | 2 | OvaryEcyanostictus |
| GO:0071320 | cellular response to cAMP | 2.35E-06 | 1.81E-08 | 27 | 2 | OvaryEcyanostictus |
| GO:0000086 | G2/M transition of mitotic cell cycle | 5.23E-06 | 4.85E-08 | 20 | 0 | OvaryEcyanostictus |
| GO:0008053 | mitochondrial fusion | 5.23E-06 | 4.85E-08 | 20 | 0 | OvaryEcyanostictus |
| GO:0006744 | ubiquinone biosynthetic process | 1.02E-05 | 1.13E-07 | 19 | 0 | OvaryEcyanostictus |
| GO:0007007 | inner mitochondrial membrane organization | 1.02E-05 | 1.13E-07 | 19 | 0 | OvaryEcyanostictus |
| GO:0090303 | positive regulation of wound healing | 1.06E-05 | 1.21E-07 | 22 | 1 | OvaryEcyanostictus |
| GO:0010976 | positive regulation of neuron projection development | 2.94E-05 | 4.39E-07 | 41 | 12 | OvaryEcyanostictus |
| GO:0021545 | cranial nerve development | 2.43E-04 | 4.90E-06 | 22 | 3 | OvaryEcyanostictus |
| GO:0006554 | lysine catabolic process | 3.58E-04 | 7.69E-06 | 14 | 0 | OvaryEcyanostictus |
| GO:0010763 | positive regulation of fibroblast migration | 6.02E-04 | 1.44E-05 | 16 | 1 | OvaryEcyanostictus |
| GO:0006734 | NADH metabolic process | 7.06E-04 | 1.79E-05 | 13 | 0 | OvaryEcyanostictus |
| GO:0006338 | chromatin remodeling | 7.06E-04 | 1.79E-05 | 13 | 0 | OvaryEcyanostictus |
| GO:0030382 | sperm mitochondrion organization | 7.06E-04 | 1.79E-05 | 13 | 0 | OvaryEcyanostictus |
| GO:0048240 | sperm capacitation | 7.06E-04 | 1.79E-05 | 13 | 0 | OvaryEcyanostictus |
| GO:0021591 | ventricular system development | 1.28E-03 | 4.15E-05 | 12 | 0 | OvaryEcyanostictus |
| GO:0048022 | negative regulation of melanin biosynthetic process | 1.28E-03 | 4.15E-05 | 12 | 0 | OvaryEcyanostictus |
| GO:2001224 | positive regulation of neuron migration | 1.28E-03 | 4.15E-05 | 12 | 0 | OvaryEcyanostictus |
| GO:2001214 | positive regulation of vasculogenesis | 1.28E-03 | 4.15E-05 | 12 | 0 | OvaryEcyanostictus |
| GO:2000670 | positive regulation of dendritic cell apoptotic process | 1.28E-03 | 4.15E-05 | 12 | 0 | OvaryEcyanostictus |
| GO:0071321 | cellular response to cGMP | 1.28E-03 | 4.15E-05 | 12 | 0 | OvaryEcyanostictus |
| GO:2000481 | positive regulation of cAMP-dependent protein kinase activity | 1.28E-03 | 4.15E-05 | 12 | 0 | OvaryEcyanostictus |
| GO:1900026 | positive regulation of substrate adhesion-dependent cell spreading | 1.28E-03 | 4.15E-05 | 12 | 0 | OvaryEcyanostictus |
| GO:0070560 | protein secretion by platelet | 1.28E-03 | 4.15E-05 | 12 | 0 | OvaryEcyanostictus |
| GO:0050774 | negative regulation of dendrite morphogenesis | 1.28E-03 | 4.15E-05 | 12 | 0 | OvaryEcyanostictus |
| GO:0031547 | brain-derived neurotrophic factor receptor signaling pathway | 1.28E-03 | 4.15E-05 | 12 | 0 | OvaryEcyanostictus |
| GO:2001244 | positive regulation of intrinsic apoptotic signaling pathway | 1.84E-03 | 6.90E-05 | 14 | 1 | OvaryEcyanostictus |
| GO:0006103 | 2-oxoglutarate metabolic process | 1.84E-03 | 6.90E-05 | 14 | 1 | OvaryEcyanostictus |
| GO:0010042 | response to manganese ion | 2.22E-03 | 9.64E-05 | 11 | 0 | OvaryEcyanostictus |
| GO:0035585 | calcium-mediated signaling using extracellular calcium source | 2.22E-03 | 9.64E-05 | 11 | 0 | OvaryEcyanostictus |
| GO:0090527 | actin filament reorganization | 2.22E-03 | 9.64E-05 | 11 | 0 | OvaryEcyanostictus |
| GO:0090315 | negative regulation of protein targeting to membrane | 2.22E-03 | 9.64E-05 | 11 | 0 | OvaryEcyanostictus |
| GO:0051549 | positive regulation of keratinocyte migration | 2.22E-03 | 9.64E-05 | 11 | 0 | OvaryEcyanostictus |
| GO:1900025 | negative regulation of substrate adhesion-dependent cell spreading | 2.22E-03 | 9.64E-05 | 11 | 0 | OvaryEcyanostictus |
| GO:1901731 | positive regulation of platelet aggregation | 2.22E-03 | 9.64E-05 | 11 | 0 | OvaryEcyanostictus |
| GO:0034241 | positive regulation of macrophage fusion | 2.22E-03 | 9.64E-05 | 11 | 0 | OvaryEcyanostictus |
| GO:0033630 | positive regulation of cell adhesion mediated by integrin | 2.22E-03 | 9.64E-05 | 11 | 0 | OvaryEcyanostictus |
| GO:0006200 | ATP catabolic process | 3.08E-03 | 1.48E-04 | 31 | 12 | OvaryEcyanostictus |
| GO:0010212 | response to ionizing radiation | 3.21E-03 | 1.59E-04 | 24 | 7 | OvaryEcyanostictus |
| GO:0022028 | tangential migration from the subventricular zone to the olfactory bulb | 4.10E-03 | 2.24E-04 | 10 | 0 | OvaryEcyanostictus |
| GO:0045070 | positive regulation of viral genome replication | 4.10E-03 | 2.24E-04 | 10 | 0 | OvaryEcyanostictus |
| GO:0090398 | cellular senescence | 4.10E-03 | 2.24E-04 | 10 | 0 | OvaryEcyanostictus |
| GO:0035092 | sperm chromatin condensation | 4.10E-03 | 2.24E-04 | 10 | 0 | OvaryEcyanostictus |
| GO:0006104 | succinyl-CoA metabolic process | 4.10E-03 | 2.24E-04 | 10 | 0 | OvaryEcyanostictus |
| GO:0070584 | mitochondrion morphogenesis | 4.10E-03 | 2.24E-04 | 10 | 0 | OvaryEcyanostictus |
| GO:0050849 | negative regulation of calcium-mediated signaling | 4.10E-03 | 2.24E-04 | 10 | 0 | OvaryEcyanostictus |
| GO:0061034 | olfactory bulb mitral cell layer development | 4.10E-03 | 2.24E-04 | 10 | 0 | OvaryEcyanostictus |
| GO:0021860 | pyramidal neuron development | 4.10E-03 | 2.24E-04 | 10 | 0 | OvaryEcyanostictus |
| GO:0007093 | mitotic cell cycle checkpoint | 5.49E-03 | 3.22E-04 | 20 | 5 | OvaryEcyanostictus |
| GO:0071880 | adenylate cyclase-activating adrenergic receptor signaling pathway | 5.49E-03 | 3.25E-04 | 12 | 1 | OvaryEcyanostictus |
| GO:0043950 | positive regulation of cAMP-mediated signaling | 5.49E-03 | 3.25E-04 | 12 | 1 | OvaryEcyanostictus |
| GO:0050714 | positive regulation of protein secretion | 5.49E-03 | 3.25E-04 | 12 | 1 | OvaryEcyanostictus |
| GO:0048008 | platelet-derived growth factor receptor signaling pathway | 7.87E-03 | 4.85E-04 | 22 | 7 | OvaryEcyanostictus |
| GO:0031122 | cytoplasmic microtubule organization | 7.88E-03 | 5.19E-04 | 9 | 0 | OvaryEcyanostictus |
| GO:0045218 | zonula adherens maintenance | 7.88E-03 | 5.19E-04 | 9 | 0 | OvaryEcyanostictus |
| GO:0006284 | base-excision repair | 7.88E-03 | 5.19E-04 | 9 | 0 | OvaryEcyanostictus |

| | | | | | | |
|---|---|---|---|---|---|---|
| GO:0090136 | epithelial cell-cell adhesion | 7.88E-03 | 5.19E-04 | 9 | 0 | OvaryEcyanostictus |
| GO:0070536 | protein K63-linked deubiquitination | 7.88E-03 | 5.19E-04 | 9 | 0 | OvaryEcyanostictus |
| GO:0045930 | negative regulation of mitotic cell cycle | 7.88E-03 | 5.19E-04 | 9 | 0 | OvaryEcyanostictus |
| GO:0090043 | regulation of tubulin deacetylation | 7.88E-03 | 5.19E-04 | 9 | 0 | OvaryEcyanostictus |
| GO:0001843 | neural tube closure | 8.41E-03 | 5.73E-04 | 17 | 4 | OvaryEcyanostictus |
| GO:0031114 | regulation of microtubule depolymerization | 8.85E-03 | 6.13E-04 | 15 | 3 | OvaryEcyanostictus |
| GO:0034612 | response to tumor necrosis factor | 8.85E-03 | 6.13E-04 | 15 | 3 | OvaryEcyanostictus |
| GO:0000724 | double-strand break repair via homologous recombination | 8.85E-03 | 6.13E-04 | 15 | 3 | OvaryEcyanostictus |
| GO:0042117 | monocyte activation | 9.82E-03 | 7.00E-04 | 11 | 1 | OvaryEcyanostictus |
| GO:0051088 | PMA-inducible membrane protein ectodomain proteolysis | 9.82E-03 | 7.00E-04 | 11 | 1 | OvaryEcyanostictus |
| GO:0042542 | response to hydrogen peroxide | 1.17E-02 | 8.41E-04 | 21 | 7 | OvaryEcyanostictus |
| GO:0006457 | protein folding | 1.35E-02 | 1.01E-03 | 19 | 6 | OvaryEcyanostictus |
| GO:0033137 | negative regulation of peptidyl-serine phosphorylation | 1.40E-02 | 1.07E-03 | 16 | 4 | OvaryEcyanostictus |
| GO:0002026 | regulation of the force of heart contraction | 1.53E-02 | 1.21E-03 | 8 | 0 | OvaryEcyanostictus |
| GO:0070375 | ERK5 cascade | 1.53E-02 | 1.21E-03 | 8 | 0 | OvaryEcyanostictus |
| GO:0032057 | negative regulation of translational initiation in response to stress | 1.53E-02 | 1.21E-03 | 8 | 0 | OvaryEcyanostictus |
| GO:0035970 | peptidyl-threonine dephosphorylation | 1.53E-02 | 1.21E-03 | 8 | 0 | OvaryEcyanostictus |
| GO:0006888 | ER to Golgi vesicle-mediated transport | 1.83E-02 | 1.50E-03 | 10 | 1 | OvaryEcyanostictus |
| GO:0007131 | reciprocal meiotic recombination | 1.83E-02 | 1.50E-03 | 10 | 1 | OvaryEcyanostictus |
| GO:0051646 | mitochondrion localization | 1.83E-02 | 1.50E-03 | 10 | 1 | OvaryEcyanostictus |
| GO:0090201 | negative regulation of release of cytochrome c from mitochondria | 1.83E-02 | 1.50E-03 | 10 | 1 | OvaryEcyanostictus |
| GO:0021756 | striatum development | 2.06E-02 | 1.73E-03 | 12 | 2 | OvaryEcyanostictus |
| GO:0070654 | sensory epithelium regeneration | 2.27E-02 | 2.04E-03 | 14 | 3 | OvaryEcyanostictus |
| GO:0061387 | regulation of extent of cell growth | 2.69E-02 | 2.47E-03 | 19 | 7 | OvaryEcyanostictus |
| GO:0002444 | myeloid leukocyte mediated immunity | 2.83E-02 | 2.80E-03 | 7 | 0 | OvaryEcyanostictus |
| GO:0031365 | N-terminal protein amino acid modification | 2.83E-02 | 2.80E-03 | 7 | 0 | OvaryEcyanostictus |
| GO:0043299 | leukocyte degranulation | 2.83E-02 | 2.80E-03 | 7 | 0 | OvaryEcyanostictus |
| GO:0042797 | tRNA transcription from RNA polymerase III promoter | 2.83E-02 | 2.80E-03 | 7 | 0 | OvaryEcyanostictus |
| GO:0042791 | 5S class rRNA transcription from RNA polymerase III type 1 promoter | 2.83E-02 | 2.80E-03 | 7 | 0 | OvaryEcyanostictus |
| GO:0002275 | myeloid cell activation involved in immune response | 2.83E-02 | 2.80E-03 | 7 | 0 | OvaryEcyanostictus |
| GO:0032781 | positive regulation of ATPase activity | 2.83E-02 | 2.80E-03 | 7 | 0 | OvaryEcyanostictus |
| GO:0045090 | retroviral genome replication | 2.83E-02 | 2.80E-03 | 7 | 0 | OvaryEcyanostictus |
| GO:0006303 | double-strand break repair via nonhomologous end joining | 2.83E-02 | 2.80E-03 | 7 | 0 | OvaryEcyanostictus |
| GO:1901881 | positive regulation of protein depolymerization | 2.83E-02 | 2.80E-03 | 7 | 0 | OvaryEcyanostictus |
| GO:0042118 | endothelial cell activation | 2.83E-02 | 2.80E-03 | 7 | 0 | OvaryEcyanostictus |
| GO:0006081 | cellular aldehyde metabolic process | 2.83E-02 | 2.80E-03 | 7 | 0 | OvaryEcyanostictus |
| GO:0045910 | negative regulation of DNA recombination | 2.83E-02 | 2.80E-03 | 7 | 0 | OvaryEcyanostictus |
| GO:0032108 | negative regulation of response to nutrient levels | 2.83E-02 | 2.80E-03 | 7 | 0 | OvaryEcyanostictus |
| GO:0051103 | DNA ligation involved in DNA repair | 2.83E-02 | 2.80E-03 | 7 | 0 | OvaryEcyanostictus |
| GO:0043504 | mitochondrial DNA repair | 2.83E-02 | 2.80E-03 | 7 | 0 | OvaryEcyanostictus |
| GO:0006354 | DNA-dependent transcription, elongation | 2.91E-02 | 2.89E-03 | 18 | 6 | OvaryEcyanostictus |
| GO:0021766 | hippocampus development | 2.91E-02 | 2.89E-03 | 18 | 6 | OvaryEcyanostictus |
| GO:0051489 | regulation of filopodium assembly | 3.12E-02 | 3.13E-03 | 16 | 5 | OvaryEcyanostictus |
| GO:0008156 | negative regulation of DNA replication | 3.12E-02 | 3.18E-03 | 9 | 1 | OvaryEcyanostictus |
| GO:0097094 | craniofacial suture morphogenesis | 3.12E-02 | 3.18E-03 | 9 | 1 | OvaryEcyanostictus |
| GO:0006099 | tricarboxylic acid cycle | 3.26E-02 | 3.39E-03 | 11 | 2 | OvaryEcyanostictus |
| GO:0050869 | negative regulation of B cell activation | 3.26E-02 | 3.39E-03 | 11 | 2 | OvaryEcyanostictus |
| GO:0071478 | cellular response to radiation | 3.37E-02 | 3.60E-03 | 14 | 4 | OvaryEcyanostictus |
| GO:0010950 | positive regulation of endopeptidase activity | 3.37E-02 | 3.60E-03 | 14 | 4 | OvaryEcyanostictus |
| GO:0000070 | mitotic sister chromatid segregation | 3.43E-02 | 3.70E-03 | 13 | 3 | OvaryEcyanostictus |
| GO:0048821 | erythrocyte development | 3.43E-02 | 3.70E-03 | 13 | 3 | OvaryEcyanostictus |
| GO:0043966 | histone H3 acetylation | 3.43E-02 | 3.70E-03 | 13 | 3 | OvaryEcyanostictus |
| GO:0016236 | macroautophagy | 4.80E-02 | 5.46E-03 | 15 | 5 | OvaryEcyanostictus |
| GO:0043484 | regulation of RNA splicing | 4.80E-02 | 5.46E-03 | 15 | 5 | OvaryEcyanostictus |
| | | | | | | |
| **Enriched in Testis in *E. cyanostictus*** | | | | | | |
| GO:0031952 | regulation of protein autophosphorylation | 5.91E-10 | 1.97E-12 | 1 | 53 | TestisEcyanostictus |
| GO:0001525 | angiogenesis | 3.69E-09 | 1.45E-11 | 14 | 93 | TestisEcyanostictus |
| GO:0006414 | translational elongation | 9.22E-08 | 4.27E-10 | 7 | 66 | TestisEcyanostictus |
| GO:0035335 | peptidyl-tyrosine dephosphorylation | 1.36E-07 | 6.47E-10 | 0 | 38 | TestisEcyanostictus |
| GO:0030324 | lung development | 3.14E-06 | 2.73E-08 | 1 | 36 | TestisEcyanostictus |
| GO:0032355 | response to estradiol stimulus | 6.64E-06 | 6.39E-08 | 2 | 39 | TestisEcyanostictus |
| GO:0030336 | negative regulation of cell migration | 1.03E-05 | 1.16E-07 | 2 | 38 | TestisEcyanostictus |
| GO:0043552 | positive regulation of phosphatidylinositol 3-kinase activity | 2.12E-05 | 3.02E-07 | 0 | 27 | TestisEcyanostictus |
| GO:0006406 | mRNA export from nucleus | 6.43E-05 | 1.08E-06 | 0 | 25 | TestisEcyanostictus |
| GO:2000811 | negative regulation of anoikis | 1.15E-04 | 2.06E-06 | 0 | 24 | TestisEcyanostictus |
| GO:0006369 | termination of RNA polymerase II transcription | 1.15E-04 | 2.06E-06 | 0 | 24 | TestisEcyanostictus |
| GO:0035909 | aorta morphogenesis | 2.03E-04 | 3.96E-06 | 0 | 23 | TestisEcyanostictus |
| GO:0032570 | response to progesterone stimulus | 3.58E-04 | 7.67E-06 | 0 | 22 | TestisEcyanostictus |
| GO:0046621 | negative regulation of organ growth | 3.58E-04 | 7.67E-06 | 0 | 22 | TestisEcyanostictus |
| GO:0050806 | positive regulation of synaptic transmission | 4.54E-04 | 1.01E-05 | 1 | 25 | TestisEcyanostictus |
| GO:0022408 | negative regulation of cell-cell adhesion | 5.61E-04 | 1.31E-05 | 0 | 20 | TestisEcyanostictus |
| GO:0050853 | B cell receptor signaling pathway | 5.61E-04 | 1.31E-05 | 0 | 20 | TestisEcyanostictus |
| GO:0055088 | lipid homeostasis | 8.99E-04 | 2.32E-05 | 5 | 36 | TestisEcyanostictus |
| GO:0050680 | negative regulation of epithelial cell proliferation | 9.08E-04 | 2.37E-05 | 0 | 19 | TestisEcyanostictus |
| GO:0009617 | response to bacterium | 9.08E-04 | 2.37E-05 | 20 | 71 | TestisEcyanostictus |
| GO:0019217 | regulation of fatty acid metabolic process | 1.29E-03 | 4.32E-05 | 0 | 18 | TestisEcyanostictus |
| GO:0090263 | positive regulation of canonical Wnt receptor signaling pathway | 1.29E-03 | 4.32E-05 | 0 | 18 | TestisEcyanostictus |
| GO:0042130 | negative regulation of T cell proliferation | 1.29E-03 | 4.32E-05 | 0 | 18 | TestisEcyanostictus |
| GO:0032846 | positive regulation of homeostatic process | 1.60E-03 | 5.74E-05 | 1 | 22 | TestisEcyanostictus |
| GO:0014068 | positive regulation of phosphatidylinositol 3-kinase cascade | 1.91E-03 | 7.36E-05 | 3 | 28 | TestisEcyanostictus |
| GO:0045017 | glycerolipid biosynthetic process | 1.91E-03 | 7.36E-05 | 3 | 28 | TestisEcyanostictus |
| GO:0060396 | growth hormone receptor signaling pathway | 1.96E-03 | 7.91E-05 | 0 | 17 | TestisEcyanostictus |
| GO:0050796 | regulation of insulin secretion | 2.23E-03 | 9.80E-05 | 2 | 24 | TestisEcyanostictus |
| GO:0007254 | JNK cascade | 2.23E-03 | 9.97E-05 | 5 | 33 | TestisEcyanostictus |
| GO:0003281 | ventricular septum development | 2.23E-03 | 1.02E-04 | 2 | 25 | TestisEcyanostictus |
| GO:0046488 | phosphatidylinositol metabolic process | 2.26E-03 | 1.03E-04 | 1 | 21 | TestisEcyanostictus |
| GO:0007172 | signal complex assembly | 3.07E-03 | 1.46E-04 | 0 | 16 | TestisEcyanostictus |
| GO:0051964 | negative regulation of synapse assembly | 3.07E-03 | 1.46E-04 | 0 | 16 | TestisEcyanostictus |
| GO:0038007 | netrin-activated signaling pathway | 3.07E-03 | 1.46E-04 | 0 | 16 | TestisEcyanostictus |
| GO:0046685 | response to arsenic-containing substance | 3.07E-03 | 1.46E-04 | 0 | 16 | TestisEcyanostictus |
| GO:2000060 | positive regulation of protein ubiquitination involved in ubiquitin-dependent protein catabolic process | 3.07E-03 | 1.46E-04 | 0 | 16 | TestisEcyanostictus |
| GO:0045444 | fat cell differentiation | 3.14E-03 | 1.53E-04 | 4 | 29 | TestisEcyanostictus |
| GO:0045944 | positive regulation of transcription from RNA polymerase II promoter | 3.35E-03 | 1.69E-04 | 26 | 77 | TestisEcyanostictus |
| GO:0007270 | neuron-neuron synaptic transmission | 4.74E-03 | 2.71E-04 | 0 | 15 | TestisEcyanostictus |
| GO:0006703 | estrogen biosynthetic process | 4.74E-03 | 2.71E-04 | 0 | 15 | TestisEcyanostictus |
| GO:0015909 | long-chain fatty acid transport | 4.74E-03 | 2.71E-04 | 0 | 15 | TestisEcyanostictus |
| GO:0019369 | arachidonic acid metabolic process | 4.74E-03 | 2.71E-04 | 0 | 15 | TestisEcyanostictus |
| GO:0000187 | activation of MAPK activity | 4.89E-03 | 2.82E-04 | 2 | 22 | TestisEcyanostictus |
| GO:0048013 | ephrin receptor signaling pathway | 4.89E-03 | 2.82E-04 | 2 | 22 | TestisEcyanostictus |
| GO:0040023 | establishment of nucleus localization | 5.68E-03 | 3.39E-04 | 1 | 19 | TestisEcyanostictus |
| GO:0045637 | regulation of myeloid cell differentiation | 5.68E-03 | 3.39E-04 | 1 | 19 | TestisEcyanostictus |
| GO:0007163 | establishment or maintenance of cell polarity | 7.03E-03 | 4.27E-04 | 12 | 46 | TestisEcyanostictus |
| GO:0010594 | regulation of endothelial cell migration | 7.03E-03 | 4.28E-04 | 5 | 30 | TestisEcyanostictus |
| GO:0060252 | positive regulation of glial cell proliferation | 7.88E-03 | 5.07E-04 | 0 | 14 | TestisEcyanostictus |

| | | | | | | |
|---|---|---|---|---|---|---|
| GO:0030837 | negative regulation of actin filament polymerization | 7.88E-03 | 5.07E-04 | 0 | 14 | TestisEcyanostictus |
| GO:0030644 | cellular chloride ion homeostasis | 7.88E-03 | 5.07E-04 | 0 | 14 | TestisEcyanostictus |
| GO:0015879 | carnitine transport | 7.88E-03 | 5.07E-04 | 0 | 14 | TestisEcyanostictus |
| GO:0045909 | positive regulation of vasodilation | 7.88E-03 | 5.07E-04 | 0 | 14 | TestisEcyanostictus |
| GO:0003333 | amino acid transmembrane transport | 8.85E-03 | 6.15E-04 | 1 | 18 | TestisEcyanostictus |
| GO:0043647 | inositol phosphate metabolic process | 8.85E-03 | 6.15E-04 | 1 | 18 | TestisEcyanostictus |
| GO:0030879 | mammary gland development | 9.31E-03 | 6.54E-04 | 4 | 26 | TestisEcyanostictus |
| GO:0001938 | positive regulation of endothelial cell proliferation | 1.28E-02 | 9.55E-04 | 0 | 13 | TestisEcyanostictus |
| GO:0030890 | positive regulation of B cell proliferation | 1.28E-02 | 9.55E-04 | 0 | 13 | TestisEcyanostictus |
| GO:0001708 | cell fate specification | 1.28E-02 | 9.55E-04 | 0 | 13 | TestisEcyanostictus |
| GO:0015813 | L-glutamate transport | 1.28E-02 | 9.55E-04 | 0 | 13 | TestisEcyanostictus |
| GO:0016064 | immunoglobulin mediated immune response | 1.28E-02 | 9.55E-04 | 0 | 13 | TestisEcyanostictus |
| GO:0006278 | RNA-dependent DNA replication | 1.28E-02 | 9.55E-04 | 0 | 13 | TestisEcyanostictus |
| GO:0048565 | digestive tract development | 1.28E-02 | 9.55E-04 | 0 | 13 | TestisEcyanostictus |
| GO:0043401 | steroid hormone mediated signaling pathway | 1.35E-02 | 1.02E-03 | 3 | 23 | TestisEcyanostictus |
| GO:0042417 | dopamine metabolic process | 1.40E-02 | 1.07E-03 | 1 | 16 | TestisEcyanostictus |
| GO:0010927 | cellular component assembly involved in morphogenesis | 1.53E-02 | 1.19E-03 | 10 | 39 | TestisEcyanostictus |
| GO:0001945 | lymph vessel development | 1.74E-02 | 1.39E-03 | 2 | 19 | TestisEcyanostictus |
| GO:0031016 | pancreas development | 1.74E-02 | 1.39E-03 | 2 | 19 | TestisEcyanostictus |
| GO:0031124 | mRNA 3'-end processing | 2.06E-02 | 1.72E-03 | 4 | 24 | TestisEcyanostictus |
| GO:0006576 | cellular biogenic amine metabolic process | 2.06E-02 | 1.72E-03 | 3 | 22 | TestisEcyanostictus |
| GO:0035264 | multicellular organism growth | 2.06E-02 | 1.72E-03 | 3 | 22 | TestisEcyanostictus |
| GO:0007584 | response to nutrient | 2.07E-02 | 1.75E-03 | 5 | 26 | TestisEcyanostictus |
| GO:0045667 | regulation of osteoblast differentiation | 2.07E-02 | 1.75E-03 | 5 | 26 | TestisEcyanostictus |
| GO:0006754 | ATP biosynthetic process | 2.07E-02 | 1.81E-03 | 0 | 12 | TestisEcyanostictus |
| GO:0006635 | fatty acid beta-oxidation | 2.07E-02 | 1.81E-03 | 0 | 12 | TestisEcyanostictus |
| GO:2001251 | negative regulation of chromosome organization | 2.07E-02 | 1.81E-03 | 0 | 12 | TestisEcyanostictus |
| GO:0001516 | prostaglandin biosynthetic process | 2.07E-02 | 1.81E-03 | 0 | 12 | TestisEcyanostictus |
| GO:0035176 | social behavior | 2.07E-02 | 1.81E-03 | 0 | 12 | TestisEcyanostictus |
| GO:0070555 | response to interleukin-1 | 2.07E-02 | 1.81E-03 | 0 | 12 | TestisEcyanostictus |
| GO:0007586 | digestion | 2.07E-02 | 1.81E-03 | 0 | 12 | TestisEcyanostictus |
| GO:0045740 | positive regulation of DNA replication | 2.07E-02 | 1.81E-03 | 0 | 12 | TestisEcyanostictus |
| GO:2001252 | positive regulation of chromosome organization | 2.09E-02 | 1.85E-03 | 1 | 15 | TestisEcyanostictus |
| GO:0046849 | bone remodeling | 2.09E-02 | 1.85E-03 | 1 | 15 | TestisEcyanostictus |
| GO:0030316 | osteoclast differentiation | 2.09E-02 | 1.85E-03 | 1 | 15 | TestisEcyanostictus |
| GO:0001890 | placenta development | 2.20E-02 | 1.97E-03 | 9 | 35 | TestisEcyanostictus |
| GO:1901616 | organic hydroxy compound catabolic process | 2.61E-02 | 2.38E-03 | 2 | 18 | TestisEcyanostictus |
| GO:0034637 | cellular carbohydrate biosynthetic process | 2.83E-02 | 2.71E-03 | 3 | 20 | TestisEcyanostictus |
| GO:0043433 | negative regulation of sequence-specific DNA binding transcription factor activity | 2.83E-02 | 2.71E-03 | 3 | 20 | TestisEcyanostictus |
| GO:0007269 | neurotransmitter secretion | 2.83E-02 | 2.74E-03 | 5 | 25 | TestisEcyanostictus |
| GO:0001578 | microtubule bundle formation | 2.83E-02 | 2.74E-03 | 5 | 25 | TestisEcyanostictus |
| GO:0006641 | triglyceride metabolic process | 2.83E-02 | 2.77E-03 | 4 | 23 | TestisEcyanostictus |
| GO:0007589 | body fluid secretion | 2.83E-02 | 2.77E-03 | 4 | 23 | TestisEcyanostictus |
| GO:0009612 | response to mechanical stimulus | 2.83E-02 | 2.77E-03 | 4 | 23 | TestisEcyanostictus |
| GO:0046165 | alcohol biosynthetic process | 2.83E-02 | 2.77E-03 | 4 | 23 | TestisEcyanostictus |
| GO:0007389 | pattern specification process | 3.05E-02 | 3.03E-03 | 29 | 72 | TestisEcyanostictus |
| GO:0007202 | activation of phospholipase C activity | 3.12E-02 | 3.20E-03 | 1 | 14 | TestisEcyanostictus |
| GO:0006766 | vitamin metabolic process | 3.12E-02 | 3.20E-03 | 1 | 14 | TestisEcyanostictus |
| GO:0055008 | cardiac muscle tissue morphogenesis | 3.12E-02 | 3.20E-03 | 1 | 14 | TestisEcyanostictus |
| GO:0051865 | protein autoubiquitination | 3.12E-02 | 3.20E-03 | 1 | 14 | TestisEcyanostictus |
| GO:0035270 | endocrine system development | 3.12E-02 | 3.20E-03 | 1 | 14 | TestisEcyanostictus |
| GO:0050766 | positive regulation of phagocytosis | 3.12E-02 | 3.20E-03 | 1 | 14 | TestisEcyanostictus |
| GO:0044070 | regulation of anion transport | 3.26E-02 | 3.35E-03 | 8 | 32 | TestisEcyanostictus |
| GO:0045577 | regulation of B cell differentiation | 3.26E-02 | 3.46E-03 | 0 | 11 | TestisEcyanostictus |
| GO:0001941 | postsynaptic membrane organization | 3.26E-02 | 3.46E-03 | 0 | 11 | TestisEcyanostictus |
| GO:0007185 | transmembrane receptor protein tyrosine phosphatase signaling pathway | 3.26E-02 | 3.46E-03 | 0 | 11 | TestisEcyanostictus |
| GO:0006691 | leukotriene metabolic process | 3.26E-02 | 3.46E-03 | 0 | 11 | TestisEcyanostictus |
| GO:0032863 | activation of Rac GTPase activity | 3.26E-02 | 3.46E-03 | 0 | 11 | TestisEcyanostictus |
| GO:0030595 | leukocyte chemotaxis | 3.26E-02 | 3.46E-03 | 0 | 11 | TestisEcyanostictus |
| GO:0001522 | pseudouridine synthesis | 3.26E-02 | 3.46E-03 | 0 | 11 | TestisEcyanostictus |
| GO:0006376 | mRNA splice site selection | 3.26E-02 | 3.46E-03 | 0 | 11 | TestisEcyanostictus |
| GO:0010833 | telomere maintenance via telomere lengthening | 3.26E-02 | 3.46E-03 | 0 | 11 | TestisEcyanostictus |
| GO:0034767 | positive regulation of ion transmembrane transport | 3.26E-02 | 3.46E-03 | 0 | 11 | TestisEcyanostictus |
| GO:0048845 | venous blood vessel morphogenesis | 3.26E-02 | 3.46E-03 | 0 | 11 | TestisEcyanostictus |
| GO:2000273 | positive regulation of receptor activity | 3.26E-02 | 3.46E-03 | 0 | 11 | TestisEcyanostictus |
| GO:0032204 | regulation of telomere maintenance | 3.26E-02 | 3.46E-03 | 0 | 11 | TestisEcyanostictus |
| GO:0050819 | negative regulation of coagulation | 3.26E-02 | 3.46E-03 | 0 | 11 | TestisEcyanostictus |
| GO:0050810 | regulation of steroid biosynthetic process | 3.26E-02 | 3.46E-03 | 0 | 11 | TestisEcyanostictus |
| GO:0002685 | regulation of leukocyte migration | 3.26E-02 | 3.46E-03 | 0 | 11 | TestisEcyanostictus |
| GO:0042551 | neuron maturation | 3.98E-02 | 4.40E-03 | 3 | 19 | TestisEcyanostictus |
| GO:0002758 | innate immune response-activating signal transduction | 3.98E-02 | 4.40E-03 | 3 | 19 | TestisEcyanostictus |
| GO:0043407 | negative regulation of MAP kinase activity | 3.98E-02 | 4.40E-03 | 3 | 19 | TestisEcyanostictus |
| GO:0001894 | tissue homeostasis | 4.01E-02 | 4.47E-03 | 4 | 22 | TestisEcyanostictus |
| GO:0032941 | secretion by tissue | 4.01E-02 | 4.47E-03 | 4 | 22 | TestisEcyanostictus |
| GO:0051896 | regulation of protein kinase B signaling cascade | 4.32E-02 | 4.86E-03 | 11 | 37 | TestisEcyanostictus |
| GO:0051259 | protein oligomerization | 4.56E-02 | 5.14E-03 | 19 | 52 | TestisEcyanostictus |
| GO:0097194 | execution phase of apoptosis | 4.83E-02 | 5.51E-03 | 7 | 28 | TestisEcyanostictus |
| GO:0006400 | tRNA modification | 4.83E-02 | 5.55E-03 | 1 | 13 | TestisEcyanostictus |
| GO:0006198 | cAMP catabolic process | 4.83E-02 | 5.55E-03 | 1 | 13 | TestisEcyanostictus |
| GO:0043576 | regulation of respiratory gaseous exchange | 4.83E-02 | 5.55E-03 | 1 | 13 | TestisEcyanostictus |

**Enriched in Ovary in *J. ornatus***

| GO-ID | Term | FDR | P-Value | #OTranscripts | #TTranscripts | Enriched in |
|---|---|---|---|---|---|---|
| GO:0090263 | positive regulation of canonical Wnt receptor signaling pathway | 3.05E-06 | 4.69E-08 | 24 | 0 | OvaryJornatus |
| GO:0021799 | cerebral cortex radially oriented cell migration | 1.01E-05 | 1.91E-07 | 29 | 2 | OvaryJornatus |
| GO:0006749 | glutathione metabolic process | 2.84E-05 | 6.50E-07 | 24 | 1 | OvaryJornatus |
| GO:0044272 | sulfur compound biosynthetic process | 3.29E-05 | 7.88E-07 | 20 | 0 | OvaryJornatus |
| GO:0001525 | angiogenesis | 2.12E-04 | 6.77E-06 | 84 | 36 | OvaryJornatus |
| GO:0051571 | positive regulation of histone H3-K4 methylation | 3.60E-04 | 1.32E-05 | 16 | 0 | OvaryJornatus |
| GO:0080182 | histone H3-K4 trimethylation | 3.60E-04 | 1.32E-05 | 16 | 0 | OvaryJornatus |
| GO:0021695 | cerebellar cortex development | 5.91E-04 | 2.32E-05 | 24 | 3 | OvaryJornatus |
| GO:0046686 | response to cadmium ion | 6.51E-04 | 2.67E-05 | 15 | 0 | OvaryJornatus |
| GO:0006110 | regulation of glycolysis | 6.51E-04 | 2.67E-05 | 15 | 0 | OvaryJornatus |
| GO:0030854 | positive regulation of granulocyte differentiation | 1.18E-03 | 5.39E-05 | 14 | 0 | OvaryJornatus |
| GO:0021869 | forebrain ventricular zone progenitor cell division | 1.39E-03 | 6.68E-05 | 17 | 1 | OvaryJornatus |
| GO:0050482 | arachidonic acid secretion | 2.10E-03 | 1.09E-04 | 13 | 0 | OvaryJornatus |
| GO:0006754 | ATP biosynthetic process | 2.10E-03 | 1.09E-04 | 13 | 0 | OvaryJornatus |
| GO:0043982 | histone H4-K8 acetylation | 2.10E-03 | 1.09E-04 | 13 | 0 | OvaryJornatus |
| GO:0043981 | histone H4-K5 acetylation | 2.10E-03 | 1.09E-04 | 13 | 0 | OvaryJornatus |
| GO:2000171 | negative regulation of dendrite development | 2.10E-03 | 1.09E-04 | 13 | 0 | OvaryJornatus |
| GO:0051893 | regulation of focal adhesion assembly | 2.35E-03 | 1.28E-04 | 16 | 1 | OvaryJornatus |
| GO:0008637 | apoptotic mitochondrial changes | 2.96E-03 | 1.67E-04 | 25 | 5 | OvaryJornatus |
| GO:0001570 | vasculogenesis | 3.41E-03 | 1.96E-04 | 18 | 2 | OvaryJornatus |
| GO:0019369 | arachidonic acid metabolic process | 3.41E-03 | 1.96E-04 | 18 | 2 | OvaryJornatus |
| GO:0046939 | nucleotide phosphorylation | 3.73E-03 | 2.20E-04 | 12 | 0 | OvaryJornatus |

| | | | | | | |
|---|---|---|---|---|---|---|
| GO:0006418 | tRNA aminoacylation for protein translation | 3.73E-03 | 2.20E-04 | 12 | 0 | OvaryJornatus |
| GO:0045136 | development of secondary sexual characteristics | 3.73E-03 | 2.20E-04 | 12 | 0 | OvaryJornatus |
| GO:0060444 | branching involved in mammary gland duct morphogenesis | 3.73E-03 | 2.20E-04 | 12 | 0 | OvaryJornatus |
| GO:0070207 | protein homotrimerization | 3.73E-03 | 2.20E-04 | 12 | 0 | OvaryJornatus |
| GO:0019370 | leukotriene biosynthetic process | 3.73E-03 | 2.20E-04 | 12 | 0 | OvaryJornatus |
| GO:0051597 | response to methylmercury | 4.05E-03 | 2.46E-04 | 15 | 1 | OvaryJornatus |
| GO:0006024 | glycosaminoglycan biosynthetic process | 4.05E-03 | 2.46E-04 | 15 | 1 | OvaryJornatus |
| GO:0006493 | protein O-linked glycosylation | 4.42E-03 | 2.74E-04 | 22 | 4 | OvaryJornatus |
| GO:0043001 | Golgi to plasma membrane protein transport | 6.68E-03 | 4.45E-04 | 11 | 0 | OvaryJornatus |
| GO:0051782 | negative regulation of cell division | 6.68E-03 | 4.45E-04 | 11 | 0 | OvaryJornatus |
| GO:0061041 | regulation of wound healing | 6.68E-03 | 4.45E-04 | 11 | 0 | OvaryJornatus |
| GO:0045662 | negative regulation of myoblast differentiation | 6.68E-03 | 4.45E-04 | 11 | 0 | OvaryJornatus |
| GO:0019262 | N-acetylneuraminate catabolic process | 6.96E-03 | 4.69E-04 | 14 | 1 | OvaryJornatus |
| GO:0008652 | cellular amino acid biosynthetic process | 7.00E-03 | 4.72E-04 | 25 | 6 | OvaryJornatus |
| GO:0035249 | synaptic transmission, glutamatergic | 7.00E-03 | 4.74E-04 | 21 | 4 | OvaryJornatus |
| GO:0035020 | regulation of Rac protein signal transduction | 7.05E-03 | 4.84E-04 | 23 | 5 | OvaryJornatus |
| GO:0010821 | regulation of mitochondrion organization | 1.13E-02 | 8.16E-04 | 22 | 5 | OvaryJornatus |
| GO:0022904 | respiratory electron transport chain | 1.21E-02 | 8.91E-04 | 13 | 1 | OvaryJornatus |
| GO:0006119 | oxidative phosphorylation | 1.21E-02 | 8.91E-04 | 13 | 1 | OvaryJornatus |
| GO:0036152 | phosphatidylethanolamine acyl-chain remodeling | 1.21E-02 | 8.98E-04 | 10 | 0 | OvaryJornatus |
| GO:0060065 | uterus development | 1.21E-02 | 8.98E-04 | 10 | 0 | OvaryJornatus |
| GO:0035338 | long-chain fatty-acyl-CoA biosynthetic process | 1.21E-02 | 8.98E-04 | 10 | 0 | OvaryJornatus |
| GO:0050729 | positive regulation of inflammatory response | 1.21E-02 | 8.98E-04 | 10 | 0 | OvaryJornatus |
| GO:0042246 | tissue regeneration | 1.68E-02 | 1.37E-03 | 21 | 5 | OvaryJornatus |
| GO:0033146 | regulation of intracellular estrogen receptor signaling pathway | 1.97E-02 | 1.69E-03 | 12 | 1 | OvaryJornatus |
| GO:0009065 | glutamine family amino acid catabolic process | 1.97E-02 | 1.69E-03 | 12 | 1 | OvaryJornatus |
| GO:0030837 | negative regulation of actin filament polymerization | 1.97E-02 | 1.69E-03 | 26 | 8 | OvaryJornatus |
| GO:0045862 | positive regulation of proteolysis | 1.97E-02 | 1.69E-03 | 26 | 8 | OvaryJornatus |
| GO:0045665 | negative regulation of neuron differentiation | 1.97E-02 | 1.69E-03 | 26 | 8 | OvaryJornatus |
| GO:0006884 | cell volume homeostasis | 2.02E-02 | 1.81E-03 | 9 | 0 | OvaryJornatus |
| GO:0042761 | very long-chain fatty acid biosynthetic process | 2.02E-02 | 1.81E-03 | 9 | 0 | OvaryJornatus |
| GO:0006569 | tryptophan catabolic process | 2.02E-02 | 1.81E-03 | 9 | 0 | OvaryJornatus |
| GO:0031065 | positive regulation of histone deacetylation | 2.02E-02 | 1.81E-03 | 9 | 0 | OvaryJornatus |
| GO:0030497 | fatty acid elongation | 2.02E-02 | 1.81E-03 | 9 | 0 | OvaryJornatus |
| GO:0071371 | cellular response to gonadotropin stimulus | 2.02E-02 | 1.81E-03 | 9 | 0 | OvaryJornatus |
| GO:0042339 | keratan sulfate metabolic process | 2.02E-02 | 1.81E-03 | 9 | 0 | OvaryJornatus |
| GO:0051639 | actin filament network formation | 2.02E-02 | 1.81E-03 | 9 | 0 | OvaryJornatus |
| GO:0034354 | 'de novo' NAD biosynthetic process from tryptophan | 2.02E-02 | 1.81E-03 | 9 | 0 | OvaryJornatus |
| GO:0043420 | anthranilate metabolic process | 2.02E-02 | 1.81E-03 | 9 | 0 | OvaryJornatus |
| GO:0000187 | activation of MAPK activity | 2.28E-02 | 2.10E-03 | 22 | 6 | OvaryJornatus |
| GO:0051963 | regulation of synapse assembly | 2.49E-02 | 2.37E-03 | 16 | 3 | OvaryJornatus |
| GO:0071300 | cellular response to retinoic acid | 2.49E-02 | 2.37E-03 | 16 | 3 | OvaryJornatus |
| GO:0043650 | dicarboxylic acid biosynthetic process | 2.49E-02 | 2.37E-03 | 16 | 3 | OvaryJornatus |
| GO:1901342 | regulation of vasculature development | 2.65E-02 | 2.60E-03 | 30 | 11 | OvaryJornatus |
| GO:0050772 | positive regulation of axonogenesis | 3.05E-02 | 3.03E-03 | 23 | 7 | OvaryJornatus |
| GO:0035272 | exocrine system development | 3.17E-02 | 3.18E-03 | 11 | 1 | OvaryJornatus |
| GO:0019432 | triglyceride biosynthetic process | 3.17E-02 | 3.18E-03 | 11 | 1 | OvaryJornatus |
| GO:0008654 | phospholipid biosynthetic process | 3.27E-02 | 3.30E-03 | 31 | 12 | OvaryJornatus |
| GO:0071383 | cellular response to steroid hormone stimulus | 3.40E-02 | 3.58E-03 | 26 | 9 | OvaryJornatus |
| GO:0046033 | AMP metabolic process | 3.40E-02 | 3.66E-03 | 8 | 0 | OvaryJornatus |
| GO:0021535 | cell migration in hindbrain | 3.40E-02 | 3.66E-03 | 8 | 0 | OvaryJornatus |
| GO:0001946 | lymphangiogenesis | 3.40E-02 | 3.66E-03 | 8 | 0 | OvaryJornatus |
| GO:0006658 | phosphatidylserine metabolic process | 3.40E-02 | 3.66E-03 | 8 | 0 | OvaryJornatus |
| GO:2001224 | positive regulation of neuron migration | 3.40E-02 | 3.66E-03 | 8 | 0 | OvaryJornatus |
| GO:0006400 | tRNA modification | 3.40E-02 | 3.66E-03 | 8 | 0 | OvaryJornatus |
| GO:0046889 | positive regulation of lipid biosynthetic process | 3.40E-02 | 3.66E-03 | 8 | 0 | OvaryJornatus |
| GO:0071260 | cellular response to mechanical stimulus | 3.40E-02 | 3.66E-03 | 8 | 0 | OvaryJornatus |
| GO:0042219 | cellular modified amino acid catabolic process | 3.40E-02 | 3.66E-03 | 8 | 0 | OvaryJornatus |
| GO:0010664 | negative regulation of striated muscle cell apoptotic process | 3.40E-02 | 3.66E-03 | 8 | 0 | OvaryJornatus |
| GO:0030148 | sphingolipid biosynthetic process | 3.40E-02 | 3.66E-03 | 8 | 0 | OvaryJornatus |
| GO:0006069 | ethanol oxidation | 3.40E-02 | 3.66E-03 | 8 | 0 | OvaryJornatus |
| GO:0008210 | estrogen metabolic process | 3.40E-02 | 3.66E-03 | 8 | 0 | OvaryJornatus |
| GO:0022400 | regulation of rhodopsin mediated signaling pathway | 3.40E-02 | 3.66E-03 | 8 | 0 | OvaryJornatus |
| GO:0045907 | positive regulation of vasoconstriction | 3.40E-02 | 3.66E-03 | 8 | 0 | OvaryJornatus |
| GO:0050850 | positive regulation of calcium-mediated signaling | 3.40E-02 | 3.66E-03 | 8 | 0 | OvaryJornatus |
| GO:0045822 | negative regulation of heart contraction | 3.40E-02 | 3.66E-03 | 8 | 0 | OvaryJornatus |
| GO:0043552 | positive regulation of phosphatidylinositol 3-kinase activity | 3.40E-02 | 3.66E-03 | 8 | 0 | OvaryJornatus |
| GO:0043497 | regulation of protein heterodimerization activity | 3.40E-02 | 3.66E-03 | 8 | 0 | OvaryJornatus |
| GO:0048262 | determination of dorsal/ventral asymmetry | 3.40E-02 | 3.66E-03 | 8 | 0 | OvaryJornatus |
| GO:0006805 | xenobiotic metabolic process | 3.53E-02 | 4.01E-03 | 17 | 4 | OvaryJornatus |
| GO:0031290 | retinal ganglion cell axon guidance | 3.59E-02 | 4.10E-03 | 15 | 3 | OvaryJornatus |
| GO:0051897 | positive regulation of protein kinase B signaling cascade | 3.59E-02 | 4.10E-03 | 15 | 3 | OvaryJornatus |
| GO:0050954 | sensory perception of mechanical stimulus | 4.02E-02 | 4.65E-03 | 27 | 10 | OvaryJornatus |
| GO:0051251 | positive regulation of lymphocyte activation | 4.68E-02 | 5.50E-03 | 25 | 9 | OvaryJornatus |
| **Enriched in Testis in *J. ornatus*** | | | | | | |
| GO:0007050 | cell cycle arrest | 9.41E-12 | 1.35E-14 | 7 | 71 | TestisJornatus |
| GO:0045944 | positive regulation of transcription from RNA polymerase II promoter | 5.62E-09 | 3.03E-11 | 68 | 168 | TestisJornatus |
| GO:0001776 | leukocyte homeostasis | 6.76E-08 | 4.98E-10 | 1 | 36 | TestisJornatus |
| GO:0007067 | mitosis | 1.69E-07 | 1.43E-09 | 18 | 75 | TestisJornatus |
| GO:0030511 | positive regulation of transforming growth factor beta receptor signaling pathway | 1.34E-06 | 1.78E-08 | 2 | 34 | TestisJornatus |
| GO:0019886 | antigen processing and presentation of exogenous peptide antigen via MHC class II | 1.34E-06 | 1.78E-08 | 2 | 34 | TestisJornatus |
| GO:0046329 | negative regulation of JNK cascade | 1.34E-06 | 1.78E-08 | 2 | 34 | TestisJornatus |
| GO:0051974 | negative regulation of telomerase activity | 1.92E-06 | 2.81E-08 | 0 | 26 | TestisJornatus |
| GO:0047496 | vesicle transport along microtubule | 1.92E-06 | 2.81E-08 | 0 | 26 | TestisJornatus |
| GO:0002051 | osteoblast fate commitment | 1.92E-06 | 2.81E-08 | 0 | 26 | TestisJornatus |
| GO:0045736 | negative regulation of cyclin-dependent protein serine/threonine kinase activity | 1.92E-06 | 2.81E-08 | 0 | 26 | TestisJornatus |
| GO:0046621 | negative regulation of organ growth | 3.46E-06 | 5.40E-08 | 1 | 29 | TestisJornatus |
| GO:0045668 | negative regulation of osteoblast differentiation | 3.46E-06 | 5.40E-08 | 1 | 29 | TestisJornatus |
| GO:0051781 | positive regulation of cell division | 4.00E-06 | 6.42E-08 | 2 | 32 | TestisJornatus |
| GO:0000122 | negative regulation of transcription from RNA polymerase II promoter | 4.38E-06 | 7.08E-08 | 48 | 117 | TestisJornatus |
| GO:0046580 | negative regulation of Ras protein signal transduction | 6.83E-06 | 1.22E-07 | 2 | 31 | TestisJornatus |
| GO:0030183 | B cell differentiation | 1.09E-05 | 2.10E-07 | 3 | 33 | TestisJornatus |
| GO:0000086 | G2/M transition of mitotic cell cycle | 1.16E-05 | 2.27E-07 | 9 | 47 | TestisJornatus |
| GO:0032092 | positive regulation of protein binding | 3.40E-05 | 8.19E-07 | 2 | 28 | TestisJornatus |
| GO:0032925 | regulation of activin receptor signaling pathway | 5.11E-05 | 1.32E-06 | 3 | 30 | TestisJornatus |
| GO:0043433 | negative regulation of sequence-specific DNA binding transcription factor activity | 6.73E-05 | 1.85E-06 | 10 | 45 | TestisJornatus |
| GO:0019048 | modulation by virus of host morphology or physiology | 9.16E-05 | 2.62E-06 | 6 | 36 | TestisJornatus |
| GO:0045176 | apical protein localization | 1.22E-04 | 3.70E-06 | 0 | 19 | TestisJornatus |
| GO:0006308 | DNA catabolic process | 1.22E-04 | 3.70E-06 | 0 | 19 | TestisJornatus |
| GO:0002076 | osteoblast development | 3.92E-04 | 1.46E-05 | 3 | 26 | TestisJornatus |
| GO:0008285 | negative regulation of cell proliferation | 5.17E-04 | 1.99E-05 | 44 | 94 | TestisJornatus |
| GO:0006338 | chromatin remodeling | 5.87E-04 | 2.30E-05 | 6 | 32 | TestisJornatus |
| GO:0006511 | ubiquitin-dependent protein catabolic process | 6.48E-04 | 2.57E-05 | 25 | 65 | TestisJornatus |
| GO:0016579 | protein deubiquitination | 6.51E-04 | 2.63E-05 | 3 | 25 | TestisJornatus |

| | | | | | | |
|---|---|---|---|---|---|---|
| GO:0002690 | positive regulation of leukocyte chemotaxis | 7.08E-04 | 2.99E-05 | 0 | 16 | TestisJornatus |
| GO:0010332 | response to gamma radiation | 1.24E-03 | 5.76E-05 | 10 | 38 | TestisJornatus |
| GO:2001234 | negative regulation of apoptotic signaling pathway | 1.31E-03 | 6.12E-05 | 17 | 50 | TestisJornatus |
| GO:0035507 | regulation of myosin-light-chain-phosphatase activity | 1.35E-03 | 6.41E-05 | 2 | 21 | TestisJornatus |
| GO:0045604 | regulation of epidermal cell differentiation | 1.35E-03 | 6.41E-05 | 2 | 21 | TestisJornatus |
| GO:0006302 | double-strand break repair | 2.10E-03 | 1.06E-04 | 8 | 33 | TestisJornatus |
| GO:0007126 | meiosis | 2.10E-03 | 1.08E-04 | 5 | 27 | TestisJornatus |
| GO:0042059 | negative regulation of epidermal growth factor receptor signaling pathway | 2.10E-03 | 1.08E-04 | 5 | 27 | TestisJornatus |
| GO:0032793 | positive regulation of CREB transcription factor activity | 2.24E-03 | 1.20E-04 | 0 | 14 | TestisJornatus |
| GO:0008156 | negative regulation of DNA replication | 2.24E-03 | 1.20E-04 | 0 | 14 | TestisJornatus |
| GO:0010259 | multicellular organismal aging | 2.24E-03 | 1.20E-04 | 0 | 14 | TestisJornatus |
| GO:0016567 | protein ubiquitination | 3.10E-03 | 1.76E-04 | 55 | 103 | TestisJornatus |
| GO:0006611 | protein export from nucleus | 4.02E-03 | 2.41E-04 | 0 | 13 | TestisJornatus |
| GO:0070988 | demethylation | 4.02E-03 | 2.41E-04 | 0 | 13 | TestisJornatus |
| GO:0032438 | melanosome organization | 4.02E-03 | 2.41E-04 | 0 | 13 | TestisJornatus |
| GO:0032091 | negative regulation of protein binding | 4.02E-03 | 2.41E-04 | 0 | 13 | TestisJornatus |
| GO:0072488 | ammonium transmembrane transport | 4.02E-03 | 2.41E-04 | 0 | 13 | TestisJornatus |
| GO:0000279 | M phase | 4.37E-03 | 2.70E-04 | 1 | 16 | TestisJornatus |
| GO:0071353 | cellular response to interleukin-4 | 4.37E-03 | 2.70E-04 | 1 | 16 | TestisJornatus |
| GO:0021952 | central nervous system projection neuron axonogenesis | 4.79E-03 | 3.01E-04 | 4 | 23 | TestisJornatus |
| GO:0030216 | keratinocyte differentiation | 4.94E-03 | 3.12E-04 | 6 | 27 | TestisJornatus |
| GO:0006310 | DNA recombination | 5.73E-03 | 3.68E-04 | 16 | 44 | TestisJornatus |
| GO:0030952 | establishment or maintenance of cytoskeleton polarity | 7.05E-03 | 4.83E-04 | 0 | 12 | TestisJornatus |
| GO:0010172 | embryonic body morphogenesis | 7.05E-03 | 4.83E-04 | 0 | 12 | TestisJornatus |
| GO:0007051 | spindle organization | 7.39E-03 | 5.11E-04 | 1 | 15 | TestisJornatus |
| GO:0009411 | response to UV | 8.24E-03 | 5.76E-04 | 18 | 46 | TestisJornatus |
| GO:0030042 | actin filament depolymerization | 8.39E-03 | 5.88E-04 | 13 | 38 | TestisJornatus |
| GO:0001825 | blastocyst formation | 1.25E-02 | 9.63E-04 | 1 | 14 | TestisJornatus |
| GO:0038124 | toll-like receptor TLR6:TLR2 signaling pathway | 1.25E-02 | 9.63E-04 | 1 | 14 | TestisJornatus |
| GO:0038123 | toll-like receptor TLR1:TLR2 signaling pathway | 1.25E-02 | 9.63E-04 | 1 | 14 | TestisJornatus |
| GO:0002027 | regulation of heart rate | 1.25E-02 | 9.63E-04 | 1 | 14 | TestisJornatus |
| GO:0034166 | toll-like receptor 10 signaling pathway | 1.25E-02 | 9.63E-04 | 1 | 14 | TestisJornatus |
| GO:1901186 | positive regulation of ERBB signaling pathway | 1.25E-02 | 9.67E-04 | 0 | 11 | TestisJornatus |
| GO:0072112 | glomerular visceral epithelial cell differentiation | 1.25E-02 | 9.67E-04 | 0 | 11 | TestisJornatus |
| GO:0009219 | pyrimidine deoxyribonucleotide metabolic process | 1.25E-02 | 9.67E-04 | 0 | 11 | TestisJornatus |
| GO:0009130 | pyrimidine nucleoside monophosphate biosynthetic process | 1.25E-02 | 9.67E-04 | 0 | 11 | TestisJornatus |
| GO:0033119 | negative regulation of RNA splicing | 1.25E-02 | 9.67E-04 | 0 | 11 | TestisJornatus |
| GO:0051304 | chromosome separation | 1.25E-02 | 9.67E-04 | 0 | 11 | TestisJornatus |
| GO:0070423 | nucleotide-binding oligomerization domain containing signaling pathway | 1.25E-02 | 9.67E-04 | 0 | 11 | TestisJornatus |
| GO:0034244 | negative regulation of transcription elongation from RNA polymerase II promoter | 1.25E-02 | 9.67E-04 | 0 | 11 | TestisJornatus |
| GO:0002064 | epithelial cell development | 1.46E-02 | 1.15E-03 | 8 | 28 | TestisJornatus |
| GO:0000910 | cytokinesis | 1.60E-02 | 1.28E-03 | 7 | 26 | TestisJornatus |
| GO:0006470 | protein dephosphorylation | 1.68E-02 | 1.36E-03 | 25 | 54 | TestisJornatus |
| GO:0032479 | regulation of type I interferon production | 1.77E-02 | 1.46E-03 | 3 | 18 | TestisJornatus |
| GO:0048011 | neurotrophin TRK receptor signaling pathway | 1.77E-02 | 1.48E-03 | 28 | 58 | TestisJornatus |
| GO:0045807 | positive regulation of endocytosis | 1.80E-02 | 1.51E-03 | 4 | 20 | TestisJornatus |
| GO:0050658 | RNA transport | 2.02E-02 | 1.82E-03 | 8 | 27 | TestisJornatus |
| GO:0033133 | positive regulation of glucokinase activity | 2.12E-02 | 1.94E-03 | 0 | 10 | TestisJornatus |
| GO:0010824 | regulation of centrosome duplication | 2.12E-02 | 1.94E-03 | 0 | 10 | TestisJornatus |
| GO:0008333 | endosome to lysosome transport | 2.12E-02 | 1.94E-03 | 0 | 10 | TestisJornatus |
| GO:0000082 | G1/S transition of mitotic cell cycle | 2.27E-02 | 2.09E-03 | 15 | 38 | TestisJornatus |
| GO:0045669 | positive regulation of osteoblast differentiation | 2.32E-02 | 2.15E-03 | 10 | 30 | TestisJornatus |
| GO:0035666 | TRIF-dependent toll-like receptor signaling pathway | 2.46E-02 | 2.32E-03 | 2 | 15 | TestisJornatus |
| GO:0008608 | attachment of spindle microtubules to kinetochore | 2.46E-02 | 2.32E-03 | 2 | 15 | TestisJornatus |
| GO:0014850 | response to muscle activity | 2.46E-02 | 2.32E-03 | 2 | 15 | TestisJornatus |
| GO:0030049 | muscle filament sliding | 2.46E-02 | 2.32E-03 | 2 | 15 | TestisJornatus |
| GO:0018105 | peptidyl-serine phosphorylation | 2.49E-02 | 2.37E-03 | 12 | 33 | TestisJornatus |
| GO:0032508 | DNA duplex unwinding | 2.55E-02 | 2.44E-03 | 5 | 21 | TestisJornatus |
| GO:0016458 | gene silencing | 2.61E-02 | 2.53E-03 | 3 | 17 | TestisJornatus |
| GO:0030512 | negative regulation of transforming growth factor beta receptor signaling pathway | 2.61E-02 | 2.53E-03 | 3 | 17 | TestisJornatus |
| GO:0046835 | carbohydrate phosphorylation | 2.61E-02 | 2.53E-03 | 3 | 17 | TestisJornatus |
| GO:0018279 | protein N-linked glycosylation via asparagine | 2.61E-02 | 2.53E-03 | 3 | 17 | TestisJornatus |
| GO:0034142 | toll-like receptor 4 signaling pathway | 2.61E-02 | 2.55E-03 | 4 | 19 | TestisJornatus |
| GO:0021700 | developmental maturation | 3.07E-02 | 3.05E-03 | 17 | 40 | TestisJornatus |
| GO:0005979 | regulation of glycogen biosynthetic process | 3.32E-02 | 3.38E-03 | 1 | 12 | TestisJornatus |
| GO:0071356 | cellular response to tumor necrosis factor | 3.32E-02 | 3.38E-03 | 1 | 12 | TestisJornatus |
| GO:0006003 | fructose 2,6-bisphosphate metabolic process | 3.32E-02 | 3.38E-03 | 1 | 12 | TestisJornatus |
| GO:0042992 | negative regulation of transcription factor import into nucleus | 3.32E-02 | 3.38E-03 | 1 | 12 | TestisJornatus |
| GO:0007569 | cell aging | 3.40E-02 | 3.53E-03 | 12 | 32 | TestisJornatus |
| GO:0060021 | palate development | 3.40E-02 | 3.66E-03 | 14 | 35 | TestisJornatus |
| GO:2001056 | positive regulation of cysteine-type endopeptidase activity | 3.42E-02 | 3.68E-03 | 16 | 38 | TestisJornatus |
| GO:0060135 | maternal process involved in female pregnancy | 3.43E-02 | 3.82E-03 | 9 | 27 | TestisJornatus |
| GO:0002467 | germinal center formation | 3.43E-02 | 3.88E-03 | 0 | 9 | TestisJornatus |
| GO:0031293 | membrane protein intracellular domain proteolysis | 3.43E-02 | 3.88E-03 | 0 | 9 | TestisJornatus |
| GO:0009264 | deoxyribonucleotide catabolic process | 3.43E-02 | 3.88E-03 | 0 | 9 | TestisJornatus |
| GO:0032859 | activation of Ral GTPase activity | 3.43E-02 | 3.88E-03 | 0 | 9 | TestisJornatus |
| GO:2001275 | positive regulation of glucose import in response to insulin stimulus | 3.43E-02 | 3.88E-03 | 0 | 9 | TestisJornatus |
| GO:0035313 | wound healing, spreading of epidermal cells | 3.43E-02 | 3.88E-03 | 0 | 9 | TestisJornatus |
| GO:0015701 | bicarbonate transport | 3.43E-02 | 3.88E-03 | 0 | 9 | TestisJornatus |
| GO:0048915 | posterior lateral line system development | 3.43E-02 | 3.88E-03 | 0 | 9 | TestisJornatus |
| GO:0010748 | negative regulation of plasma membrane long-chain fatty acid transport | 3.43E-02 | 3.88E-03 | 0 | 9 | TestisJornatus |
| GO:0090314 | positive regulation of protein targeting to membrane | 3.43E-02 | 3.88E-03 | 0 | 9 | TestisJornatus |
| GO:0090299 | regulation of neural crest formation | 3.43E-02 | 3.88E-03 | 0 | 9 | TestisJornatus |
| GO:0090162 | establishment of epithelial cell polarity | 3.43E-02 | 3.88E-03 | 0 | 9 | TestisJornatus |
| GO:0021979 | hypothalamus cell differentiation | 3.43E-02 | 3.88E-03 | 0 | 9 | TestisJornatus |
| GO:0046386 | deoxyribose phosphate catabolic process | 3.43E-02 | 3.88E-03 | 0 | 9 | TestisJornatus |
| GO:0051250 | negative regulation of lymphocyte activation | 3.43E-02 | 3.88E-03 | 0 | 9 | TestisJornatus |
| GO:0032000 | positive regulation of fatty acid beta-oxidation | 3.43E-02 | 3.88E-03 | 0 | 9 | TestisJornatus |
| GO:0048385 | regulation of retinoic acid receptor signaling pathway | 3.43E-02 | 3.88E-03 | 0 | 9 | TestisJornatus |
| GO:0045648 | positive regulation of erythrocyte differentiation | 3.43E-02 | 3.88E-03 | 0 | 9 | TestisJornatus |
| GO:0006855 | drug transmembrane transport | 3.59E-02 | 4.13E-03 | 2 | 14 | TestisJornatus |
| GO:0001678 | cellular glucose homeostasis | 3.59E-02 | 4.13E-03 | 2 | 14 | TestisJornatus |
| GO:0003407 | neural retina development | 3.59E-02 | 4.13E-03 | 2 | 14 | TestisJornatus |
| GO:0043154 | negative regulation of cysteine-type endopeptidase activity involved in apoptotic process | 3.68E-02 | 4.24E-03 | 19 | 42 | TestisJornatus |
| GO:0072511 | divalent inorganic cation transport | 3.78E-02 | 4.37E-03 | 24 | 49 | TestisJornatus |
| GO:0048706 | embryonic skeletal system development | 4.26E-02 | 4.94E-03 | 25 | 50 | TestisJornatus |
| GO:0030001 | metal ion transport | 4.55E-02 | 5.33E-03 | 64 | 101 | TestisJornatus |

| Color codes | |
|---|---|
| (grey) | involved in transcription |
| (red) | involved in apoptosis |
| (pink) | wnt signaling pathway |
| (light green) | TGFbeta superfamily |
| (yellow) | new candidates |

| | |
|---|---|
| **OVARY** | |
| GO:0008585 | female gonad development, contains transcripts of the following genes |
| | apoptosis regulator bax |
| | forkhead box protein o3 |
| | beta- -n-acetylglucosaminyltransferase lunatic fringe |
| | bone morphogenetic protein 15 |
| | tcdd-inducible poly |
| | forkhead box l2 |
| | prohibitin |
| GO:2000242 | negative regulation of reproductive process, contains transcripts of the following genes |
| | apoptosis regulator bax |
| | extracellular sulfatase sulf-1 |
| | wee1-like protein kinase 2 |
| | bone morphogenetic protein 15 |
| | bone morphogenetic protein 7 |
| | protein wnt-5a isoform x1 |
| GO:0060766 | negative regulation of androgen receptor signaling pathway, contains transcripts of the following genes |
| | forkhead box protein h1 |
| | prohibitin |
| | disabled homolog 2 isoform x3 |
| GO:0008209 | androgen metabolic process, contains transcripts of the following genes |
| | estradiol 17-beta-dehydrogenase 1 |
| | peroxisomal multifunctional enzyme type 2 |
| | tcdd-inducible poly |
| | scavenger receptor class b member 1 |
| **TESTIS** | |
| GO:0019100 | male germ-line sex determination, contains transcripts of the following genes |
| | mitogen-activated protein kinase kinase kinase 4 |
| GO:0032570 | response to progesterone stimulus, contains transcripts of the following genes |
| | transforming growth factor beta-3 |
| | transforming growth factor beta-2 |
| | suppressor of cytokine signaling 1 |
| | claudin-4 |
| | desmoglein-2 |
| | transforming protein |
| | caveolin 1 |
| | pyridoxal kinase |
| | transforming protein |
| GO:0032355 | response to estradiol stimulus, contains transcripts of the following genes |
| | protein c-ets-1 |
| | vitamin d3 receptor |
| | signal transducer and activator of transcription 3 |
| | sodium-dependent phosphate transport protein 2b |
| | protein kinase c alpha type |
| | signal transducer and activator of transcription 3 |
| | protein patched homolog 1 |
| | protein kinase c alpha type |
| | epidermal growth factor receptor |
| | cyclin-dependent kinase 2 |
| | suppressor of cytokine signaling 1 |
| | insulin-like growth factor binding protein 2 |
| | protein kinase c alpha type |
| | epidermal growth factor receptor |
| | 5-hydroxytryptamine receptor 6 |

**Supplementary Material Table 10**

Species-specific genes in all comparisons falling in the categories sex determination and differentiation

A) Genes specifically overexpressed in *A. burtoni*

| **Genes overexpressed in ovary/female tissue of *A. burtoni*** | **OE in** | **logFC Ovary** | **logFC Female tissue** |
|---|---|---|---|
| dachshund homolog 2-like (transcription factor involved in regulation of organogenesis) | ovary/females | 3.02 | 1.39 |
| follistatin (activin antagonist, specific inhibitor of biosynthesis/secretion of pituitary follicle stimulating hormone, binds and bioneutralizes TGF-β superfamily members)* | ovary/females | 3.4 ± 0.07 | 1.77 ± 0.06 |
| follistatin-related protein 3 (binding and antagonizing protein for TGF-β superfamily members) | ovary/females | 2.23 | 1.37 ± 0.02 |
| frizzled-8 (receptor for Wnt proteins) | ovary | 2.52 | |
| gdnf family receptor alpha-4-like (neurotrophic factor) | ovary | 2.34 | |
| hepatocyte nuclear factor 4-alpha (transcription factor) | ovary/females | 3.82 | 1.97 |
| inhibin beta b chain (two gene copies, inhibits the secretion of follitropin by the pituitary gland, member of TGF-β superfamily) | ovary/females | 2.21 | 2.13 |
| secreted frizzled-related protein 2 (soluble frizzled-related proteins are modulators of Wnt-signaling, regulates cell growth/differentiation in specific cell types) | ovary | 3.25 | |
| synaptophysin (possibly involved in structural functions) | ovary/females | 3.93 | |
| wnt7a (ligand for the frizzled-receptors, probable developmental protein, in female mammals involved in the anterior-posterior axis development in the reproductive tract) | ovary | 1.29 | |
| **Genes overexpressed in testis/male tissue of *A. burtoni*** | | **logFC Testis** | **logFC Male tissue** |
| activin receptor type-1b-like (transmembrane serine/threonine kinase receptor, TGF-β superfamily member) | testis | 1.25 | |
| anti-mullerian hormone (glycoprotein, produced by Sertoli cells of the testis, causes regression of the Muellerian duct in mammals) | males | | 1.55 |
| at-rich interactive domain-containing protein 5a (DNA-binding protein, may regulate transcription) | testis/males | 4.63 | 4.53 |
| basic helix-loop-helix transcription factor scleraxis | testis/males | 4.87 | 2.29 |
| beta-arrestin-1 (regulates agonist-mediated G-protein coupled receptor signaling) | testis/males | 3.18 ± 0.10 | 1.42 ± 0.04 |
| choline-phosphate cytidylyltransferase b (controls phosphatidylcholine synthesis) | testis | 4.68 ± 0.01 | |
| fanconi anemia group a protein (DNA repair protein) | males | | 1.02 |
| fibroblast growth factor 10 (regulation of embryonic development, cell proliferation and differentiation) | testis | 3.18 | |
| frizzled-3, different isoforms in male tissue and testis, (receptor for Wnt proteins) | testis/males | 1.70± 0.12 | 1.40 ± 0.05 |
| insulin-like growth factor 1 receptor-like (receptor tyrosine kinase), two gene copies | testis/males | 1.27 ± 0.30<br>1.71 | <br>1.3 |
| insulin receptor (receptor tyrosine kinase) | testis | 1.29 | |
| matrix metalloproteinase-15 (endopeptidase, degrades components of the extracellular matrix) | males | | 1.01 |
| matrix metalloproteinase-19 (see above) | males | | 1.76 |
| nuclear protein 1 (chromatin-binding protein, implicated in stress signaling) | testis | 2.70 | |
| nuclear receptor coactivator 3 (binds nuclear receptors, stimulates transcriptional activities in a hormone-dependent fashion) | males | | 1.08 |
| retinoic acid receptor alpha (binds to target response elements, regulates gene expression, implicated in germ cell development during spermatogenesis, survival of | testis | 1.19 | |

| | | | |
|---|---|---|---|
| spermatocytes ) | | | |
| roundabout homolog 2 (receptor for SLIT2, and probably SLIT1, which are possible molecular guidance cues in cellular migration) | males | | 1.25 ± 0.00 |
| schwannomin-interacting protein 1 (found in axon initial segments and nodes of Ranvier) | testis | 1.61 | |
| sox9a (transcription factor, sox9 in tetrapods regulates transcription of anti-Muellerian hormone) | testis | 1.55 | |
| splicing factor 1 (spliceosome assembly, possibly transcriptional repressor) | testis | 1.03 ± 0.03 | |
| steroidogenic factor 1 (transcriptional activator, essential for sexual differentiation and formation of the primary steroidogenic tissues) | males | | 2.07 |
| testis-specific y-encoded-like protein 5 (modulation of cell growth and cellular response to gamma radiation, regulation of p53/TP53) | males | | 1.26 |
| transcription factor 7-like 1-a-like (participates in the Wnt-signaling pathway) | testis | 1.16 ± 0.01 | |
| transcription factor 7-like 2-like (participates in the Wnt-signaling pathway) | males | | 1.57 |
| vascular endothelial growth factor receptor 1 (tyrosine-protein kinase, cell-surface receptor for VEGFA, VEGFB and PGF) | testis | 2.35 | |
| 3-oxo-5-alpha-steroid 4-dehydrogenase 2 (converts testosterone into 5-alpha-dihydrotestosterone and progesterone/corticosterone into 5-alpha-3-oxosteroids, central role in sexual differentiation and androgen physiology) | testis/males | 5.44 ± 0.02 | 2.78 ± 0.04 |

B) Genes specifically overexpressed in *O. ventralis*

| **Genes overexpressed in ovary/female tissue of *O. ventralis*** | **OE in** | **logFC Ovary** | **logFC Female tissue** |
|---|---|---|---|
| activin receptor type-1c (may be involved for left-right pattern formation during embryogenesis, TGF-β superfamily member) | ovary | 2.61 | |
| activin receptor type-1b-like (transmembrane serine/threonine kinase receptor, TGF-β superfamily member) | females | | 1.02 |
| activin receptor type-2a (serine/threonine kinase receptor, TGF-β superfamily member) | ovary | 2.03 | |
| ankyrin repeat and socs box protein 1 (may play a role in testis development, probable substrate-recognition component of a SCF-like ECS E3 ligase complex) | females | | 1.02 |
| bone morphogenetic protein 8a (induces cartilage/bone formation, TGF-β superfamily member) | ovary | 2.20 | |
| chromobox protein homolog 2 (part of a multiprotein complex maintaining the transcriptionally repressive state of many genes, involved in sexual development, activator of SF1 expression) | ovary | 1.25 ± 0.22 | |
| chromodomain-helicase-dna-binding protein 7 (transcription regulator) | ovary/females | 1.57 ± 0.26 | 1.09 |
| frizzled-3 (receptor for Wnt-proteins) | ovary | 1.13 ± 0.1 | |
| histone (chromatin structure protein) | ovary/females | 2.14 ± 0.01 | 1.09 ± 0.008 |
| low-density lipoprotein receptor-related protein 6 (cell-surface coreceptor of Wnt/beta-catenin signaling) | ovary/females | 2.04 ± 0.003 | 1.05 ± 0.002 |
| nuclear receptor coactivator 3 (directly binds nuclear receptors, stimulates the transcriptional activities in a hormone-dependent fashion) | ovary | 1.17 ± 0.06 | |
| pleckstrin homology domain-containing family a member 1-like (binds specifically to phosphatidylinositol 3,4-diphosphate) | ovary | 1.21 | |
| retinoic acid receptor alpha (binds to target response | ovary | 1.39 ± 0.51 | |

| | | | |
|---|---|---|---|
| elements regulates gene expression, implicated in germ cell development during spermatogenesis, survival of spermatocytes ) | | | |
| ubiquitin-protein ligase e3a-like (accepts ubiquitin and transfers it to its substrates) | ovary | 1.19 | |
| **Genes overexpressed in testis/male tissue of *O. ventralis*** | | **logFC Testis** | **logFC Male tissue** |
| adp-ribosylation factor 1 (GTP-binding protein, involved in protein trafficking) | testis | 1.75 ± 0.01 | |
| ciliary neurotrophic factor receptor (binds CNTF) | testis | 1.17 | |
| cytosolic phospholipase a2 (selectively hydrolyzes arachidonyl phospholipids in the sn-2 position releasing arachidonic acid, inflammatory response) | testis/males | 2.88 ± 0.17 | 1.48 ± 0.07 |
| cbp p300-interacting transactivator 3-like (stimulates estrogen-dependent transactivation activity mediated by estrogen receptors signaling, positively regulates TGF-β signaling) | males | | 1.14 ± 0.00 |
| dachshund homolog 2 (transcription factor involved in regulation of organogenesis | males | | 1.13 |
| delta-sterol reductase | males | | 1.93 |
| doublesex- and mab-3-related transcription factor 3 (see main text) | testis/males | 7.78 | 3.41 |
| gdnf family receptor alpha-4-like (neurotrophic factor) | testis | 2.57 | |
| hepatocyte nuclear factor 4-alpha (transcription factor) | testis | 2.07 | |
| long-chain-fatty-acid-ligase acsbg2 (may play a role in spermatogenesis) | testis | 5.21 | |
| luteinizing hormone receptor (G-protein coupled hormone receptor) | males | | 3.68 ± 1.09 |
| meiotic recombination protein spo11 (mediates DNA cleavage initiating meiotic recombination) | testis/males | 1.28 ± 0.12 | 1.32 ± 0.13 |
| nuclear receptor coactivator 3, second gene copy, (see above) | testis | 1.98 ± 0.01 | |
| odd-skipped-related 1 (transcription factor regulating embryonic heart and urogenital development) | males | | 1.27 ± 0.05 |
| pituitary adenylate cyclase-activating polypeptide type i receptor (possible role in release of hormones and spermatogenesis/sperm motility) | males | | 1.17 |
| plasma retinol-binding protein 1 | testis/males | 3.92 | 1.72 |
| pleckstrin homology domain-containing family a member 1, other gene copy as in ovary (binds specifically to phosphatidylinositol 3,4-diphosphate) | testis | 1.37 ± 0.29 | |
| retinol dehydrogenase 10 (converts all-trans-retinol to all-trans-retinal) | males | | 1.01 |
| signal transducer and activator of transcription 5b (mediates cellular responses to cytokine KITLG/SCF and other growth factors) | males | | 1.35 |
| slit homolog 3 (possible molecular guidance cue in cellular migration) | testis | 1.80 | |
| tyrosine-protein kinase receptor ufo (transduces signals from the extracellular matrix into the cytoplasm by binding growth factor GAS6) | testis | 2.51 ± 0.00 | |
| vascular endothelial growth factor receptor 1-like (cell-surface tyrosine-protein kinase) | males | | 1.77 |

C) Genes specifically overexpressed in *E. cyanostictus*

| **Genes overexpressed in ovary/female tissue of *E. cyanostictus*** | **OE in** | **logFC Ovary** | **logFC Female tissue** |
|---|---|---|---|
| bone morphogenetic protein receptor type-1a (receptor for BMP-2 and BMP-4, activates transcription, TGF-β superfamily member) | females | | 1.07 ± 0.002 |
| dachshund homolog 2 (transcription factor, involved in regulation of organogenesis), different gene copy than in *A. burtoni* | ovary/females | 1.50 | 1.13 |
| odd-skipped-related 1 (transcription factor, regulation of embryonic heart and urogenital development) | ovary/females | 3.20 ± 0.03 | 1.59 ± 0.03 |
| pre-b-cell leukemia transcription factor 1 (transcriptional activator, may have a role in steroidogenesis and, subsequently, sexual development and differentiation) | females | | 1.26 |
| retinoic acid receptor alpha (regulates gene expression, implicated in germ cell development during spermatogenesis, survival of spermatocytes ), different gene copy than in *O. ventralis* | females | | 1.02 |
| ribonucleoside-diphosphate reductase large subunit (DNA synthesis) | ovary | 1.15 | |
| translation initiation factor eif-2b subunit delta (catalyzes the exchange of eukaryotic initiation factor 2-bound GDP for GTP) | ovary | 1.18 ± 0.01 | |
| translation initiation factor eif-2b subunit epsilon (see above) | ovary | 1.20 ± 0.003 | |
| **Genes overexpressed in testis/male tissue of *E. cyanostictus*** | | **logFC Testis** | **logFC Male tissue** |
| a disintegrin and metalloproteinase with thrombospondin motifs 1 (cleaves aggrecan, a cartilage proteoglycan, may play a critical role in follicular ruptur) | males | | 1.44 ± 0.00 |
| anti-mullerian hormone, different isoforms as in *A. burtoni*, (glycoprotein, produced by the Sertoli cells of the testis, causes regression of the Muellerian duct in mammals) | males | | 1.57 ± 0.03 |
| at-rich interactive domain-containing protein 5b (transcription coactivator) | testis/males | 2.18 | 1.08 ± 0.00 |
| bone morphogenetic protein 4 (induces cartilage/bone formation, TGF-β superfamily member) | males | | 1.27 |
| bone morphogenetic protein 7 (induces cartilage/bone formation, TGF-β superfamily member) | testis | 1.21 | |
| bone morphogenetic protein 8a (induces cartilage/bone formation, TGF-β superfamily member) | testis | 2.14 ± 0.17 | |
| calpain-5, two gene copies are expressed, (calcium-dependent cysteine protease) | males | 1.89 | 1.11, 1.88 |
| dna excision repair protein ercc-1 (non-catalytic component of a structure-specific DNA repair endonuclease) | testis/males | 2.36 ± 0.01 | 1.21 ± 0.01 |
| fibroblast growth factor 8 (embryonic development, cell proliferation, differentiation, migration) | males | | 1.53 ± 0.12 |
| forkhead box protein o3, two gene copies, (transcriptional activator, triggers apoptosis in the absence of survival factors) | testis/males | 2.95 | 1.12 |
| frizzled-2 (receptor for Wnt proteins) | testis/males | 2.04 | 1.03 |
| frizzled-4 (receptor for Wnt-proteins) | testis | 1.05 | |
| frizzled-8 (receptor for Wnt-proteins) | males | | 2.72 |
| frizzled-7a (receptor for Wnt-proteins) | males | | 1.08 |
| histone-lysine n-methyltransferase mll4-like 9 (oocyte growth) | males | | 1.08 ± 0.04 |
| inhibin beta a chain a (inhibits the secretion of follitropin by the pituitary gland, TGF-β superfamily member) | males | | 1.64 ± 0.00 |
| inhibin beta a chain b (see above) | males | | 1.11 |

| | | | |
|---|---|---|---|
| matrix metalloproteinase-14 (seems to specifically activate progelatinase A) | testis | 2.18 | |
| secreted frizzled-related protein 1 (inhibits Wnt1/Wnt4-mediated TCF-dependent transcription) | testis | 2.27 | |
| secreted frizzled-related protein 5 (modulator of Wnt-signaling) | testis | 2.46 | |
| sonic hedgehog (binds to the patched receptor, activates transcription) | testis/males | 8.60 | 2.80 |
| sox9a (transcription factor, sox9 in tetrapods regulates transcription of anti-Muellerian hormone) | males | | 1.21 ± 0.20 |
| sox9b (transcription factor, see above) | testis | 1.62 | |
| sphingosine-1-phosphate lyase 1 (elevates stress-induced ceramide production and apoptosis) | males | | 1.67 ± 0.01 |
| superoxide dismutase (destroys radicals) | testis | 1.02 | |
| transcription initiation factor tfiid subunit 4 (part of multimeric protein complex that mediates promoter responses to various activators and repressors) | testis | 1.25 | |
| transcription factor gata-4 (transcriptional activator, Sertoli cell marker, regulates *amh* expression) | males | | 1.52 |
| transcription factor 4 (initiation of neuronal differentiation) | males | | 1.19 ± 0.02 |
| transcription factor 7-like 2-like (Wnt-signaling pathway, modulates MYC expression) | males | | 1.20 ± 0.00 |
| transcription factor 21 (involved in epithelial-mesenchymal interactions, basic helix-loop-helix family) | testis/males | 4.16 | 3.24 |
| tyrosine-protein kinase blk (non-receptor tyrosine kinase involved in B-lymphocyte development, differentiation and signaling) | testis | 1.88 | |
| tyrosine-protein phosphatase non-receptor type 11 (signal transduction from cell surface to nucleus) | testis | 1.18 ± 0.01 | |

D) Genes specifically overexpressed in *J. ornatus*

| **Genes overexpressed in ovary/female tissue of *J. ornatus*** | **OE in** | **logFC Ovary** | **logFC Female tissue** |
|---|---|---|---|
| anti-mullerian hormone (glycoprotein, produced by Sertoli cells of the testis, causes regression of the Muellerian duct in mammals) | ovary | 1.35 | |
| at-rich interactive domain-containing protein 5a (may act as repressor and down-regulate enhancer-dependent gene expression) | ovary/females | 8.71 | 4.49 |
| bone morphogenetic protein 7 (induces cartilage/bone formation, TGF-β superfamily member) | ovary | 1.46 | |
| bone morphogenetic protein receptor type-1b (receptor for BMP7/OP-1 and GDF5, TGF-β superfamily member) | ovary | 1.84 | |
| cytosolic phospholipase a2 (inflammatory response) | ovary/females | 3.85 ± 0.23 | 1.99 ± 0.12 |
| dachshund homolog 1 (transcription factor involved in regulation of organogenesis) | females | | 1.42 |
| frizzled-8 (receptor for Wnt proteins) | females | | 1.28 |
| insulin receptor-like (receptor tyrosine kinase) | ovary | 1.08 | |
| iroquois-class homeodomain protein irx-5 (involved in embryonic developmental processes) | females | | 2.47 |
| isocitrate dehydrogenase (catalyzes the decarboxylation of isocitrate to 2-oxoglutarate) | ovary | 1.35 ± 0.02 | |
| lim homeobox protein lhx8 (transcription factor, differentiation of neurons/mesenchymal cells) | ovary/females | 2.46 ± 0.005 | 1.37 ± 0.03 |
| luteinizing hormone receptor (G-protein coupled hormone receptor) | ovary/females | 2.23 ± 0.17 | 1.41 ± 0.02 |
| non-specific lipid-transfer (may play a role in regulating steroidogenesis) | ovary | 1.37 | |
| nuclear protein 1 (chromatin-binding protein, converts stress signals into a program of gene expression | ovary | 2.86 | |

| | | | |
|---|---|---|---|
| empowering cells with resistance to the stress) | | | |
| nuclear receptor coactivator 3, (binds nuclear receptors, stimulates the transcriptional activities in a hormone-dependent fashion) | ovary | 1.31 ± 0.02 | |
| nuclear receptor subfamily 0 group b member 1 (Dax1, orphan nuclear receptor, development of the hypothalamic-pituitary-adrenal-gonadal axis) | ovary | 1.51 | |
| prohibitin (inhibits DNA synthesis, regulates proliferation) | females | | 1.27 ± 0.003 |
| prohibitin 2, two different gene copies, (estrogen receptor-selective coregulator that potentiates the inhibitory activities of antiestrogens, represses activity of estrogens) | ovary/females | 1.24 ± 0.05 | 1.7 |
| sphingosine-1-phosphate lyase 1 (elevates stress-induced ceramide production and apoptosis) | ovary/females | 2.94 ± 0.02 | 1.45 ± 0.01 |
| steroidogenic factor 1 (transcriptional activator, seems to be essential for sexual differentiation and formation of the primary steroidogenic tissues) | ovary/females | 2.41 | 1.22 |
| transcription factor 21 (epithelial-mesenchymal interactions, basic helix-loop-helix family) | ovary | 2.77 | |
| translation initiation factor eif-2b subunit beta (catalyzes exchange of eukaryotic initiation factor 2-bound GDP for GTP) | ovary | 1.33 ± 0.02 | |
| tyrosine-protein kinase transmembrane receptor ror2 (may be involved in early formation of chondrocytes and may be required for cartilage/growth plate development) | ovary/females | 3.04 | 1.6 |
| wnt2b, copy one (ligand for frizzled receptors, probable developmental protein) | ovary | 1.97 ± 0.05 | |
| wnt4 | females | | 1.25 |
| **Genes overexpressed in testis/male tissue of *J. ornatus*** | | **logFC Testis** | **logFC Male tissue** |
| activin receptor type-2a (transmembrane serine/threonine kinase, TGF-β superfamily member) | testis | 1.03 | |
| ankyrin repeat and socs box protein 1 (may play a role in testis development, component of a SCF-liske ECS E3 ligase complex) | testis | 1.56 | |
| apoptosis regulator bax (accelerates programmed cell death) | testis | 1.88 | |
| calpain-5 (endopeptidase) | testis | 1.89 | |
| chromobox protein homolog 2 (component of multiprotein complex required to maintain the transcriptionally repressive state of many genes, involved in sexual development, activator of SF1 expression) | testis | 1.13 ± 0.13 | |
| endoplasmic reticulum metallopeptidase 1 (required for organization of somatic cells and oocytes into discrete follicular structures) | testis | 1.64 ± 0.05 | |
| factor in the germline alpha (transcription factor, postnatal oocyte-specific gene expression) | testis/males | 1.27 ± 0.11 | 1.17 ± 0.04 |
| follistatin-related protein 3 (binding and antagonizing protein for TGF-β superfamily members) | testis/males | 2.62 ± 0.13 | 1.26 ± 0.1 |
| iroquois-class homeodomain protein irx-5, other gene copy as in females, (involved in embryonic developmental processes) | testis | 1.74 | |
| lim homeobox protein lhx9 (involved in gonad development) | males | | 1.0 |
| nad-dependent protein deacetylase sirtuin-1 (links transcriptional regulation directly to intracellular energetics) | testis | 1.14 | |
| peptidyl-prolyl cis-trans isomerase fkbp4 (component of steroid receptor heterocomplexes) | testis | 1.67 | |
| tumor protein 63 (DNA binding transcriptional activator or repressor) | testis/males | 2.95 ± 0.11 | 2.01 ± 0.1 |

| | | | |
|---|---|---|---|
| wnt2b, copy two (ligand for frizzled receptors, probable developmental protein) | males | | 2.19 ± 0.06 |
| wnt7a (ligand for frizzled receptors, allows sexually dimorphic development of the mullerian ducts) | males | | 1.42 |
| zinc finger x-chromosomal protein (probable transcriptional activator) | males | | 1.2 ± 0.01 |

*color codes: green – TGF-β superfamily members, pink – wnt-pathway members, grey – transcription factors

Supplementary Material Table 11 Comparison of DE genes in the transcriptome data with previous published Q-PCR data from Böhne *et al*. 2013

| | Expression in adult gonad tissue | | | | | | |
|---|---|---|---|---|---|---|---|
| Gene | *A. burtoni* | *A. burtoni* Böhne *et al*. 2013 | *O. ventralis* | *O. ventralis* Böhne *et al*. 2013 | *J. ornatus* | *N. pulcher* (lamprologine as *J. ornatus*) Böhne *et al*. 2013 | *E. cyanostictus* |
| *ctnnb1A* | not DE* | not DE | not DE | OE** in ovary | not DE | OE in ovary | not DE |
| *cyp11b2* | OE in testis (6.41 ± 1.38) *** | OE in testis | OE in testis (9.94 ± 1.38) | OE in testis | OE in testis (5.82 ± 1.31) | OE in testis | OE in testis (6.62 ± 2.37) |
| *cyp19a1A* | OE in ovary (6.05) | OE in ovary | OE in testis (2.66) | not DE (higher in males, not significant) | OE in ovary (8.03) | OE in ovary | OE in ovary (7.86) |
| *cyp19a1B* | OE in testis (8.35 ± 0.01) | OE in testis | not DE | not DE | OE in ovary (2.3 ± 0.31) | OE in ovary | not expressed |
| *dax1A* | not DE | OE in ovary | OE in testis (1.45) | not DE | OE in ovary (1.51) | OE in ovary | OE in testis (1.2) |
| *dmrt1* | OE in testis (5.19 ± 0.01) | OE in testis | OE in testis (2.87 ± 0.03) | OE in testis | OE in testis (3.01 ± 0.01) | OE in testis | OE in testis (7.06 ± 0.004) |
| *figla* | OE in ovary (6.81 ± 0.07) | OE in ovary | OE in ovary (60.1 ± 0.03) | OE in ovary | OE in testis (1.27 ± 0.11) | OE in ovary | OE in ovary (3.13 ± 0.15) |
| *foxl2A* | OE in ovary (4.74) | OE in ovary | OE in ovary (2.7) | OE in ovary | OE in ovary (5.73) | OE in ovary | OE in ovary (4.61) |
| *foxl2B* | OE in testis (3.8) | not DE(huge variance in ovary) | not expressed | not tested | not expressed | not tested | OE in testis (3.17) |
| *gata4* | not DE | OE in ovary | OE in testis (2.08) | not DE | not expressed | not DE | OE in testis (1.86) |
| *nanos1A* | not expressed | OE in ovary | not expressed | OE in ovary | not expressed | OE in ovary | not DE |
| *nanos1B* | OE in ovary (2.41) | OE in ovary | not DE | OE in ovary | not expressed | not DE | not DE |
| *rspondin* | not expressed | not DE | not expressed | OE in testis | not expressed | not DE | not expressed |
| *nr5a2* | not expressed | not DE | not expressed | not DE | not expressed | OE in ovary | not expressed |
| *sf1A* | OE in testis (3.48 ± 0.1) | not DE (slightly higher in males but not significant) | OE in testis (4.69 ± 0.79) | not tested | OE in ovary (2.22 ± 0.23) | not tested | OE in testis (1.78 ± 0.33) |
| *nr5a5* | not expressed | not expressed | not expressed | OE in ovary | OE in ovary (7.2 ± 0.01) | OE in ovary | not expressed |
| *sox9A* | OE in testis (1.55) | not DE | not expressed | OE in ovary | not expressed | not DE | not expressed |
| *sox9B* | not DE | OE in ovary | not DE | OE in ovary | not DE | OE in ovary | OE in testis (1.62) |
| *wnt4A* | OE in ovary (3.07) | OE in ovary | not expressed | OE in ovary | not expressed | not tested | OE in ovary (1.48) |
| *wnt4B* | not expressed | OE in testis | OE in testis (8.38 ± 0.15) | OE in testis | OE in testis (4.4 ± 0.03) | OE in testis | OE in testis (6.07 ± 0.23) |
| *wt1A* | not DE | not DE | OE in testis (2.01 ± 0.03) | not DE | not DE | OE in ovary | OE in testis (2.32 ± 0.02) |
| *wt1B* | OE in ovary (1.27 ± 0.02) | not tested | not DE | not DE | OE in testis (2.17 ± 0.01) | OE in ovary | not DE |

*DE, differentially expressed, **OE, over expressed, ***if several transcripts of the same gene are expressed, the mean logFC is given, for details on single values see Supplementary Material Table 3

Supplementary Material Table 12 Expression of steroid hormone receptors in testis and ovary of the four species

| | Expression in adult gonad tissue | | | | Accession number ensembl |
|---|---|---|---|---|---|
| Gene | *A. burtoni* | *O. ventralis* | *J. ornatus* | *E. cyanostictus* | |
| *esr1* | OE in testis (2.91) | OE in ovary (3.3) | OE in ovary (1.12) | not DE | ENSONIG00000013354 |
| *esr2a* | not DE | not DE | not DE | not DE | ENSONIG00000005633 |
| *esr2b* | 1 of 4 transcripts OE in ovary (1.02) | OE in testis (1.99 ± 0.44) | not DE | 2 of 4 transcripts OE in ovary (1.45 ± 0.07) | ENSONIG00000001710 |
| *gperA* | not expressed | not expressed | not expressed | not expressed | ENSONIG00000020935 |
| *gperB* | not in reference sequences | not in reference sequences | not in reference sequences | not in reference sequences | ENSONIG00000021208 |
| *arA* | OE in testis (2.26 ± 0.04) | OE in testis (3.67 ± 0.53) | not DE | OE in testis (2.22 ± 0.15) | ENSONIG00000012854 |
| *arB* | not DE | OE in ovary (2.29 ± 0.25) | OE in ovary (2.09 ± 0.33) | not DE | ENSONIG00000017538 |

**Supplementary Material Table 13**

Assignment of genes over-expressed in ovary or testis in all four species and belonging to the GO categories sex determination and sex differentiation to expression modules

| OE in | genename | module | gene annotation |
|---|---|---|---|
| ovary | on.mrna.LG11.655 | black | cbp p300-interacting transactivator 3-like |
| ovary | on.mrna.LG12.202 | black | frizzled-10 |
| ovary | on.mrna.LG14.199 | black | tcdd-inducible poly |
| ovary | on.mrna.LG18.391 | black | bcl-2-related ovarian killer protein |
| ovary | on.mrna.LG19.337 | black | forkhead box protein o3 |
| ovary | on.mrna.LG2.254 | black | transcription factor sox-3-like |
| ovary | on.mrna.UNK211.2 | black | forkhead box l2 |
| ovary | on.mrna.LG12.912 | brown | doublesex- and mab-3-related transcription factor 2 |
| ovary | on.mrna.LG14.147 | brown | secreted frizzled-related protein 2 |
| ovary | on.mrna.LG14.453 | brown | ribonucleoside-diphosphate reductase large subunit |
| ovary | on.mrna.LG16-21.10[illegible] | brown | secreted frizzled-related protein 3 |
| ovary | on.mrna.LG20.619 | brown | tyrosine-protein phosphatase non-receptor type 11 |
| ovary | on.mrna.LG4.681 | brown | prohibitin |
| ovary | on.mrna.LG5.872 | brown | transcription factor ap-2 gamma |
| ovary | on.mrna.LG6.5 | brown | apoptosis regulator bax |
| ovary | on.mrna.UNK31.47 | brown | bone morphogenetic protein receptor type-1a |
| ovary | on.mrna.UNK5.27 | brown | hepatocyte growth factor receptor |
| ovary | on.mrna.UNK5.79 | brown | g1 s-specific cyclin-d1 |
| ovary | on.mrna.UNK70.13 | brown | bone morphogenetic protein 15 |
| ovary | on.mrna.UNK75.11 | brown | beta- -n-acetylglucosaminyltransferase lunatic fringe-lik |
| ovary | on.mrna.UNK9.28 | brown | mothers against decapentaplegic homolog 5 |
| ovary | on.mrna.LG1.561 | magenta | sal-like protein 1 |
| ovary | on.mrna.LG12.645 | midnightblue | peroxisomal multifunctional enzyme type 2 |
| ovary | on.mrna.LG15.531 | midnightblue | ensconsin |
| ovary | on.mrna.LG20.650 | midnightblue | protein wnt-5a isoform x1 |
| ovary | on.mrna.UNK168.10 | midnightblue | g1 s-specific cyclin-d1 |
| ovary | on.mrna.UNK248.2 | midnightblue | inhibitor of growth protein 2 |
| ovary | on.mrna.UNK89.5 | midnightblue | mothers against decapentaplegic homolog 9 |
| testis | on.mrna.LG22.257 | black | kelch-like protein 10 isoform 2 |
| testis | on.mrna.UNK11.32 | black | protein tilb homolog |
| testis | on.mrna.UNK3026.1 | black | kelch-like protein 10 |
| testis | on.mrna.LG10.30 | brown | histone |
| testis | on.mrna.LG19.753 | brown | protein jagged-2 |
| testis | on.mrna.LG2.292 | brown | transforming growth factor beta-2 |
| testis | on.mrna.LG23.318 | brown | mast stem cell growth factor receptor kit |
| testis | on.mrna.LG7.1096 | brown | long-chain-fatty-acid-- ligase acsbg2 |
| testis | on.mrna.LG8-24.496 | brown | transcription factor 7-like 2-lik |
| testis | on.mrna.LG9.184 | brown | retinol dehydrogenase 10 |
| testis | on.mrna.LG11.251 | cyan | frizzled-6 |
| testis | on.mrna.UNK8.15. | cyan | transcription factor 7-like 2-like |
| testis | on.mrna.LG16-21.57[illegible] | magenta | frizzled-7 |
| testis | on.mrna.LG6.1003 | magenta | high mobility group protein b2 |
| testis | on.mrna.LG8-24.198 | magenta | meiotic recombination protein dmc1 lim15 homolog |
| testis | on.mrna.LG8-24.580 | magenta | follicle stimulating hormone receptor |
| testis | on.mrna.LG18.751 | midnightblue | nuclear receptor coactivator 2 |
| testis | on.mrna.LG7.740 | midnightblue | nipped-b-like protein |
| testis | on.mrna.UNK164.3 | midnightblue | high mobility group protein b2 |
| testis | on.mrna.UNK206.10 | midnightblue | polyadenylate-binding protein 2 |

# Supplementary Material Table 14

Annotation and expression of gene modules with significant species association

| | Annotation | Expression in Gonads | | | |
|---|---|---|---|---|---|
| Module darkorange | | *J. ornatus* | *A. burtoni* | *O. ventralis* | *E. cyanostictus* |
| on.mrna.LG1.158.1 | - | not expressed | not expressed | OE in testis | OE in testis |
| on.mrna.LG1.702.1 | - | not expressed | OE in testis | OE in testis | OE in testis |
| on.mrna.LG1.733.1 | - | not expressed | not DE | OE in testis | not expressed |
| on.mrna.LG10.103.5 | LOC100708751, tripartite motif-containing protein 16-like | not DE | OE in ovary | not DE | OE in testis |
| on.mrna.LG10.2.1 | FV3gorf5R, viral protein | not expressed | not DE | not expressed | OE in testis |
| on.mrna.LG11.106.1 | PEG10, paternally expressed 10,retrotransposon-like protein | not expressed | not expressed | not expressed | not expressed |
| on.mrna.LG11.867.1 | pik3r4, phosphoinositide-3-kinase, regulatory subunit 4 | not DE | not DE | not DE | OE in ovary |
| on.mrna.LG12.260.2 | cyp1d1, cytochrome P450, family 1, subfamily D, polypeptide 1 | not expressed | not expressed | OE in ovary | OE in testis |
| on.mrna.LG12.872.1 | interleukin-8-like | not expressed | not expressed | not expressed | OE in testis |
| on.mrna.LG13.176.1 | - | not expressed | OE in testis | not expressed | OE in testis |
| on.mrna.LG13.349.1 | - | not expressed | not expressed | not expressed | not expressed |
| on.mrna.LG13.734.1 | fin bud initiation factor b | not expressed | not expressed | not expressed | OE in testis |
| on.mrna.LG15.84.2 | acyl-CoA thioesterase 1 | OE in ovary | OE in ovary | OE in ovary | OE in ovary |
| on.mrna.LG16-21.597.1 | SAMD9, sterile alpha motif domain containing 9 | not expressed | OE in testis | OE in testis | OE in testis |
| on.mrna.LG17.166.1 | - | not expressed | not expressed | not expressed | not expressed |
| on.mrna.LG17.690.1 | TBC1 domain family, member 22a | not DE | not DE | not DE | not DE |
| on.mrna.LG19.808.1 | - | not expressed | OE in testis | OE in testis | not expressed |
| on.mrna.LG3.71.2 | novel immune-type receptor | not expressed | OE in testis | not expressed | not expressed |
| on.mrna.LG4.19.1 | interferon-induced protein 44-like | not expressed | OE in testis | not expressed | OE in testis |
| on.mrna.LG4.905.1 | perforin 1 (pore forming protein) | not expressed | not expressed | OE in testis | not expressed |
| on.mrna.LG4.951.1 | gbgt1, Globoside alpha-1,3-N-acetylgalactosaminyltransferase 1 | OE in ovary | not DE | not DE | OE in testis |
| on.mrna.LG5.243.6 | Itih3, inter-alpha-trypsin inhibitor heavy chain3 | not DE | not DE | not DE | OE in testis |
| on.mrna.LG6.1018.1 | E3 ubiquitin-protein ligase TRIM21-like | not expressed | OE in testis | not expressed | not expressed |
| on.mrna.LG7.275.1 | fam206a, family with sequence similarity 206, member A | not expressed | OE in testis | OE in testis | OE in testis |
| on.mrna.LG7.808.1 | - | not expressed | OE in testis | not expressed | OE in testis |
| on.mrna.LG7.814.1 | protein phosphatase 2A1 B alpha subunit | not DE | not DE | not DE | not DE |
| on.mrna.LG7.815.2 | protein phosphatase 2, regulatory subunit B, delta isoform | not DE | not DE | not DE | not DE |
| on.mrna.LG8-24.678.1 | neoverrucotoxin subunit alpha-like | not expressed | not expressed | not expressed | OE in testis |
| on.mrna.UNK1.143.1 | interferon-induced protein 44 | not expressed | not expressed | not expressed | not expressed |
| on.mrna.UNK1.144.2 | interferon-induced protein 44 like | not expressed | OE in testis | OE in testis | OE in testis |
| on.mrna.UNK139.5.1 | neoverrucotoxin subunit alpha-like | not expressed | not expressed | not expressed | not expressed |
| on.mrna.UNK144.3.3 | prolyl-tRNA synthetase associated domain-containing protein 1-like | OE in ovary | not DE | not DE | not DE |
| on.mrna.UNK160.7.1 | sialic acid binding Ig-like lectin 1, sialoadhesin | not expressed | OE in testis | OE in testis | OE in testis |
| on.mrna.UNK169.5.2 | tumor necrosis factor alpha | not expressed | not expressed | not expressed | not expressed |
| on.mrna.UNK183.4.1 | harbinger transposase derived 1 | not expressed | OE in testis | OE in testis | OE in testis |
| on.mrna.UNK2042.1.1 | - | not expressed | not expressed | not expressed | OE in testis |
| on.mrna.UNK2154.1.1 | - | not expressed | OE in testis | OE in testis | not expressed |
| on.mrna.UNK22.35.1 | - | not expressed | not expressed | not expressed | not expressed |
| on.mrna.UNK237.2.4 | focadhesin | not DE | not DE | not DE | not DE |
| on.mrna.UNK24.29.1 | butyrophilin-like 2 | OE in ovary | not DE | not DE | not DE |
| on.mrna.UNK287.4.1 | ADAMTS-like 1 | not expressed | not DE | not expressed | OE in testis |
| on.mrna.UNK293.2.1 | interferon-induced protein 44 | not expressed | not expressed | not expressed | not expressed |
| on.mrna.UNK314.5.1 | butyrophilin-like 10 | not expressed | OE in testis | OE in ovary | OE in testis |
| on.mrna.UNK318.3.1 | - | not expressed | not expressed | not expressed | OE in testis |
| on.mrna.UNK339.3.1 | focadhesin | not DE | not DE | not DE | not DE |
| on.mrna.UNK34.13.1 | Fc receptor-like B | not expressed | not DE | not expressed | not expressed |
| on.mrna.UNK35.8.1 | interferon-induced protein 44 | not expressed | not expressed | not expressed | not expressed |
| on.mrna.UNK36.10.1 | - | not expressed | OE in testis | OE in testis | not expressed |
| on.mrna.UNK4370.1.2 | - | not expressed | not expressed | not expressed | OE in testis |
| on.mrna.UNK46.17.1 | - | not expressed | not expressed | not expressed | OE in testis |
| on.mrna.UNK479.1.1 | focadhesin | not DE | not DE | not DE | not DE |
| on.mrna.UNK4816.1.1 | - | not expressed | not DE | not expressed | not expressed |
| on.mrna.UNK531.2.1 | - | not expressed | not expressed | OE in testis | OE in testis |
| on.mrna.UNK76.4.1 | - | not expressed | OE in testis | not expressed | OE in testis |

| | Annotation | Expression in Gonads | | | |
|---|---|---|---|---|---|
| Module darkturquoise | | *J. ornatus* | *A. burtoni* | *O. ventralis* | *E. cyanostictus* |
| on.mrna.LG1.231.1 | Myb-related transcription factor, partner of profilin | not expressed | OE in testis | not expressed | OE in testis |
| on.mrna.LG1.651.1 | - | not expressed | not expressed | OE in testis | not expressed |
| on.mrna.LG10.218.1 | - | not DE | not DE | OE in Ovary | not DE |
| on.mrna.LG11.816.1 | - | not expressed | OE in testis | not expressed | OE in testis |
| on.mrna.LG12.755.1 | N-acetylated-alpha-linked acidic dipeptidase-like protein-like | not expressed | OE in testis | not expressed | not expressed |
| on.mrna.LG12.851.1 | - | not expressed | not expressed | not expressed | not expressed |
| on.mrna.LG12.937.3 | leucine rich repeat containing 2 | not DE | not DE | not DE | not DE |
| on.mrna.LG13.447.2 | fucose mutarotase-like | OE in Ovary | not expressed | not DE | not DE |
| on.mrna.LG14.13.11 | protein-tyrosine sulfotransferase 1-like | not DE | OE in Ovary | OE in Ovary | OE in Ovary |
| on.mrna.LG14.7.1 | gastric intrinsic factor-like | not expressed | not expressed | not expressed | OE in testis |
| on.mrna.LG15.352.1 | transcription cofactor vestigial-like protein 2-like | not expressed | OE in testis | OE in testis | not expressed |
| on.mrna.LG15.515.1 | deleted in malignant brain tumors 1 | not expressed | OE in testis | not DE | not DE |
| on.mrna.LG15.516.1 | deleted in malignant brain tumors 1 | not expressed | OE in testis | not expressed | not expressed |
| on.mrna.LG16-21.372.1 | poliovirus receptor-related 2 | not expressed | OE in testis | not expressed | OE in testis |

| | | | | | |
|---|---|---|---|---|---|
| on.mrna.LG16-21.374.1 | poliovirus receptor-related 2 | not expressed | not DE | not expressed | OE in testis |
| on.mrna.LG16-21.376.1 | poliovirus receptor-related 2 | not DE | OE in testis | OE in testis | OE in testis |
| on.mrna.LG16-21.50.1 | MOB-like protein phocein-like | not DE | OE in Ovary | not DE | not DE |
| on.mrna.LG16-21.744.1 | pogo transposable element with KRAB domain | not expressed | OE in testis | OE in testis | OE in testis |
| on.mrna.LG18.351.3 | zonadhesin-like | not expressed | OE in testis | not expressed | not DE |
| on.mrna.LG18.352.1 | zonadhesin-like | not expressed | not expressed | not expressed | not expressed |
| on.mrna.LG18.726.1 | acyl-coenzyme A thioesterase 2, mitochondrial-like | not expressed | not expressed | not expressed | not expressed |
| on.mrna.LG20.156.1 | ERCC6-PGBD3 readthrough | not expressed | OE in testis | not expressed | not expressed |
| on.mrna.LG20.54.2 | neural cell adhesion molecule L1-like | not DE | not DE | not DE | not DE |
| on.mrna.LG20.7.1 | - | not expressed | not expressed | not expressed | not expressed |
| on.mrna.LG22.593.1 | glutathione S-transferase A-like | not expressed | OE in testis | not expressed | OE in testis |
| on.mrna.LG3.321.1 | - | not expressed | OE in testis | not expressed | not expressed |
| on.mrna.LG4.374.1 | - | not expressed | not DE | not expressed | not DE |
| on.mrna.LG4.775.4 | Apolipoprotein-L3 | not expressed | OE in testis | not expressed | not DE |
| on.mrna.LG5.1012.1 | - | not expressed | OE in testis | OE in testis | not expressed |
| on.mrna.LG5.1018.1 | butyrophilin-like 1 | not expressed | OE in testis | not expressed | not expressed |
| on.mrna.LG5.800.2 | transglutaminase 5 | not expressed | not expressed | not expressed | not expressed |
| on.mrna.LG5.98.1 | - | not expressed | not expressed | OE in testis | not expressed |
| on.mrna.LG5.994.1 | granzyme B-like | not expressed | OE in testis | not expressed | not expressed |
| on.mrna.LG6.549.1 | sialidase 3, tandem duplicate 5 | not expressed | OE in testis | OE in testis | not expressed |
| on.mrna.LG7.401.1 | - | not DE | OE in Ovary | OE in Ovary | not DE |
| on.mrna.LG7.402.1 | 1-acylglycerol-3-phosphate O-acyltransferase 2 | not DE | OE in Ovary | OE in Ovary | not DE |
| on.mrna.LG7.952.1 | - | not expressed | OE in Ovary | not expressed | not expressed |
| on.mrna.LG8-24.166.1 | zinc finger CCCH-type containing 7B | not DE | not DE | OE in Ovary | not DE |
| on.mrna.LG8-24.647.1 | neoverrucotoxin subunit alpha-like | not expressed | not DE | not DE | not expressed |
| on.mrna.LG9.320.1 | repressor of RNA polymerase III transcription MAF1 homolog | not DE | not DE | not DE | not DE |
| on.mrna.LG9.336.1 | myomesin 1, 185kDa | OE in testis | OE in testis | not expressed | OE in testis |
| on.mrna.UNK10.64.1 | zinc finger, MYM-type 1 | not expressed | OE in testis | not expressed | not expressed |
| on.mrna.UNK100.13.1 | uncharacterized protein K02A2.6-like | not expressed | OE in testis | not expressed | not expressed |
| on.mrna.UNK108.18.1 | retrotransposable element | not expressed | OE in testis | OE in testis | not expressed |
| on.mrna.UNK1394.1.1 | poliovirus receptor-related 3 like | not expressed | OE in testis | not expressed | OE in testis |
| on.mrna.UNK1398.1.1 | - | not expressed | not expressed | not expressed | not expressed |
| on.mrna.UNK1458.1.1 | - | not expressed | not expressed | not expressed | not expressed |
| on.mrna.UNK149.6.1 | uncharacterized LOC754259 | not expressed | OE in testis | not expressed | OE in testis |
| on.mrna.UNK1519.1.1 | - | not expressed | OE in testis | not expressed | not expressed |
| on.mrna.UNK1610.1.1 | - | not expressed | OE in testis | OE in testis | not expressed |
| on.mrna.UNK162.11.1 | pogo transposable element with KRAB domain | not expressed | OE in testis | not expressed | OE in testis |
| on.mrna.UNK175.4.1 | - | not expressed | not expressed | OE in testis | not expressed |
| on.mrna.UNK197.3.1 | class I histocompatibility antigen, F10 alpha chain-like | not DE | OE in testis | not DE | OE in testis |
| on.mrna.UNK200.3.1 | pogo transposable element with KRAB domain | not expressed | OE in testis | not expressed | not expressed |
| on.mrna.UNK22.4.1 | - | not expressed | not expressed | not expressed | not expressed |
| on.mrna.UNK227.2.1 | uncharacterized LOC754259 | not expressed | OE in testis | not expressed | not expressed |
| on.mrna.UNK23.1.3 | gamma-glutamyl cyclotransferase b | not expressed | not DE | not expressed | not expressed |
| on.mrna.UNK234.8.1 | GTPase, IMAP family member 2 | not expressed | OE in testis | not expressed | OE in testis |
| on.mrna.UNK236.2.1 | putative nuclease HARBI1-like | not expressed | OE in testis | not expressed | OE in testis |
| on.mrna.UNK256.4.1 | zinc finger, BED-type containing 4 | not expressed | OE in testis | not expressed | not expressed |
| on.mrna.UNK264.3.1 | paternally expressed 10 | not expressed | not DE | not expressed | not expressed |
| on.mrna.UNK2793.1.1 | - | not expressed | not expressed | OE in testis | not expressed |
| on.mrna.UNK313.8.1 | - | not expressed | not expressed | not expressed | not expressed |
| on.mrna.UNK3223.1.1 | - | not expressed | not expressed | OE in testis | not expressed |
| on.mrna.UNK342.6.1 | paternally expressed 10 | not expressed | not expressed | not expressed | not expressed |
| on.mrna.UNK3588.1.1 | - | not expressed | not expressed | not expressed | not expressed |
| on.mrna.UNK38.8.1 | - | not expressed | OE in testis | not expressed | not expressed |
| on.mrna.UNK386.4.1 | - | not expressed | OE in testis | not expressed | not expressed |
| on.mrna.UNK3973.1.1 | ERCC6-PGBD3 readthrough | not expressed | OE in testis | not expressed | not expressed |
| on.mrna.UNK4606.1.1 | - | not expressed | OE in testis | not expressed | not expressed |
| on.mrna.UNK4687.1.1 | gag-pol fusion protein | not expressed | not expressed | not expressed | not DE |
| on.mrna.UNK48.9.2 | zgc:174680, uncharacterized protein | not expressed | not DE | not expressed | OE in testis |
| on.mrna.UNK519.4.1 | poliovirus receptor-related 4 | not expressed | OE in testis | not expressed | not expressed |
| on.mrna.UNK520.1.1 | - | not expressed | OE in testis | OE in testis | OE in testis |
| on.mrna.UNK550.2.1 | butyrophilin, subfamily 2, member A1 | not expressed | OE in testis | not expressed | not expressed |
| on.mrna.UNK599.1.1 | - | not expressed | not expressed | OE in testis | not expressed |
| on.mrna.UNK6.2.1 | nuclear GTPase, germinal center associated | not expressed | OE in testis | not expressed | not expressed |
| on.mrna.UNK618.1.1 | Retrovirus-related Pol polyprotein from transposon 17.6 | not expressed | OE in testis | not expressed | not expressed |
| on.mrna.UNK62.10.1 | myelin oligodendrocyte glycoprotein | OE in testis | OE in testis | on.mrna.UNK62 | not DE |
| on.mrna.UNK62.11.2 | cytosolic sulfotransferase 3-like | not expressed | not expressed | not expressed | not expressed |
| on.mrna.UNK63.7.1 | - | not expressed | OE in testis | not expressed | not expressed |
| on.mrna.UNK766.1.1 | glutathione S-transferase A-like | OE in Ovary | OE in testis | on.mrna.UNK76 | OE in testis |
| on.mrna.UNK766.2.1 | glutathione S-transferase A-like | OE in Ovary | OE in testis | on.mrna.UNK76 | OE in testis |
| on.mrna.UNK77.19.1 | endogenous retrovirus group FRD, member 1 | not expressed | OE in testis | not expressed | not expressed |
| on.mrna.UNK83.22.1 | - | not expressed | not expressed | not expressed | not expressed |
| | Annotation | Expression in Gonads | | | |
| Module darkgrey | | *J. ornatus* | *A. burtoni* | *O. ventralis* | *E. cyanostictus* |
| on.mrna.LG1.472.1 | - | not expressed | not expressed | not expressed | not DE |
| on.mrna.LG1.473.1 | - | not expressed | not expressed | not expressed | not DE |
| on.mrna.LG1.474.1 | - | not expressed | OE in testis | OE in testis | OE in testis |

| | | | | | |
|---|---|---|---|---|---|
| on.mrna.LG1.610.1 | gag-pol fusion protein | not expressed | not expressed | not expressed | OE in testis |
| on.mrna.LG11.76.1 | ZBED1, zinc finger, BED-type containing 1 | not expressed | not expressed | not expressed | OE in testis |
| on.mrna.LG11.196.2 | solute carrier family 40 (iron-regulated transporter), member 1 | not expressed | not expressed | not expressed | OE in testis |
| on.mrna.LG13.884.1 | cyclin-dependent kinase inhibitor 1B-like | not expressed | OE in ovary | OE in ovary | not expressed |
| on.mrna.LG14.105.1 | zgc:162193 | not expressed | not expressed | not expressed | not expressed |
| on.mrna.LG15.55.1 | cytosolic 5'-nucleotidase 1B-like | not expressed | not DE | not DE | not DE |
| on.mrna.LG15.702.1 | calmin | not expressed | not expressed | not expressed | OE in testis |
| on.mrna.LG16-21.129.1 | viral protein | not expressed | not expressed | not expressed | OE in testis |
| on.mrna.LG16-21.626.1 | - | not expressed | OE in testis | OE in testis | not DE |
| on.mrna.LG2.508.1 | immune-responsive gene 1 protein-like | not expressed | not expressed | not expressed | not expressed |
| on.mrna.LG2.509.1 | tRNA threonylcarbamoyladenosine biosynthesis protein YwlC | not expressed | not expressed | not expressed | not expressed |
| on.mrna.LG2.510.1 | putative transmembrane protein TA-2 | not expressed | OE in testis | OE in testis | OE in testis |
| on.mrna.LG2.512.1 | probable alpha-ketoglutarate-dependent hypophosphite dioxygenase-like | not expressed | not expressed | not expressed | not expressed |
| on.mrna.LG2.622.2 | moesin b | not expressed | OE in testis | not expressed | OE in testis |
| on.mrna.LG20.569.1 | PTB domain-containing engulfment adapter protein 1-like | not expressed | not expressed | not expressed | OE in testis |
| on.mrna.LG20.722.1 | activin receptor type-1B-like | not DE | OE in ovary | OE in ovary | not expressed |
| on.mrna.LG22.9.1 | proteasome subunit beta type-6-B like protein-like | not expressed | not expressed | not expressed | OE in testis |
| on.mrna.LG22.607.5 | KIAA1737 | OE in testis | not expressed | not expressed | OE in testis |
| on.mrna.LG22.673.1 | - | not expressed | OE in testis | OE in testis | not DE |
| on.mrna.LG23.93.1 | meprin A, beta | not expressed | not expressed | not expressed | OE in testis |
| on.mrna.LG3.283.1 | solute carrier family 16, member 12 | OE in ovary | OE in ovary | not DE | OE in testis |
| on.mrna.LG3.306.1 | reverse transcriptase family protein | not expressed | not expressed | not expressed | OE in testis |
| on.mrna.LG5.839.1 | eEF1A2 binding protein-like | not DE | not expressed | OE in testis | OE in testis |
| on.mrna.LG6.28.1 | - | not expressed | not DE | not expressed | not expressed |
| on.mrna.LG6.688.1 | - | not expressed | not expressed | not expressed | OE in testis |
| on.mrna.LG6.839.1 | lysyl oxidase homolog 4-like | not expressed | not expressed | not expressed | OE in testis |
| on.mrna.LG7.294.4 | G protein-coupled receptor kinase 5-like | not expressed | not expressed | not expressed | OE in testis |
| on.mrna.UNK10.63.1 | zinc finger, BED domain containing 4 | not expressed | not expressed | not expressed | OE in testis |
| on.mrna.UNK101.3.1 | orf250-like protein | not expressed | not expressed | not expressed | OE in testis |
| on.mrna.UNK104.6.1 | mannose receptor, C type 2 | not expressed | not expressed | not expressed | OE in testis |
| on.mrna.UNK12.46.1 | c3a anaphylatoxin chemotactic receptor-like | not expressed | not expressed | not expressed | OE in testis |
| on.mrna.UNK120.5.1 | Pr gag-pro-pol | not expressed | not expressed | OE in testis | OE in testis |
| on.mrna.UNK136.6.1 | gypsy retrotransposon integrase 1 | not expressed | not expressed | OE in testis | OE in testis |
| on.mrna.UNK1401.1.1 | zgc:153932 | not expressed | not expressed | not expressed | OE in testis |
| on.mrna.UNK173.1.1 | gag-pol fusion protein | not expressed | not expressed | not expressed | OE in testis |
| on.mrna.UNK2091.1.1 | Fc fragment of IgG binding protein | not expressed | not expressed | not expressed | OE in testis |
| on.mrna.UNK2186.1.2 | - | not expressed | not expressed | OE in testis | OE in testis |
| on.mrna.UNK2312.1.1 | tectorin alpha | not expressed | not expressed | not expressed | OE in testis |
| on.mrna.UNK25.8.1 | - | not expressed | not expressed | not expressed | OE in testis |
| on.mrna.UNK251.3.1 | retrotransposable element | not expressed | not expressed | not expressed | not expressed |
| on.mrna.UNK264.9.1 | | not expressed | not expressed | not expressed | OE in testis |
| on.mrna.UNK2641.1.1 | | not expressed | not expressed | not expressed | not expressed |
| on.mrna.UNK3.49.1 | G protein-coupled receptor 126 | not expressed | not expressed | not expressed | OE in testis |
| on.mrna.UNK309.1.1 | retrotransposable element | not expressed | OE in testis | OE in testis | OE in testis |
| on.mrna.UNK34.14.1 | uncharacterized LOC100888072 | not expressed | OE in testis | not expressed | OE in testis |
| on.mrna.UNK364.1.1 | - | not expressed | not expressed | not expressed | OE in testis |
| on.mrna.UNK3809.1.1 | zinc knuckle protein | not expressed | not expressed | not expressed | OE in testis |
| on.mrna.UNK394.1.1 | putative integrase core domain protein | not expressed | not expressed | not expressed | OE in testis |
| on.mrna.UNK3957.1.1 | retrotransposable element | not expressed | not expressed | not expressed | OE in testis |
| on.mrna.UNK40.5.1 | alkaline phosphatase, tissue-nonspecific isozyme-like | not expressed | not expressed | not expressed | OE in testis |
| on.mrna.UNK4111.1.1 | zinc finger, BED-type containing 4 | not expressed | not expressed | not expressed | OE in testis |
| on.mrna.UNK505.3.1 | - | not expressed | not expressed | not expressed | OE in testis |
| on.mrna.UNK523.3.1 | asteroid homolog 1 | not expressed | not expressed | not expressed | not DE |
| on.mrna.UNK528.1.1 | GTPase IMAP family member 7-like | not expressed | not expressed | not expressed | OE in testis |
| on.mrna.UNK528.2.1 | GTPase, IMAP family member 2 | not expressed | not expressed | not expressed | OE in testis |
| on.mrna.UNK55.5.3 | - | not expressed | not expressed | not expressed | OE in ovary |
| on.mrna.UNK555.2.1 | - | OE in testis | not expressed | not expressed | OE in testis |
| on.mrna.UNK570.1.1 | - | not expressed | not expressed | not expressed | not DE |
| on.mrna.UNK602.1.1 | - | not expressed | not expressed | not expressed | OE in testis |
| on.mrna.UNK62.3.1 | - | not expressed | not expressed | not expressed | not expressed |
| on.mrna.UNK636.1.1 | retrovirus-related Pol polyprotein from transposon 412 | not expressed | not expressed | not expressed | OE in testis |
| on.mrna.UNK782.1.1 | G2/M-phase specific E3 ubiquitin ligase | not expressed | not expressed | OE in testis | not DE |
| on.mrna.UNK86.3.1 | putative nuclease HARBI1-like | not expressed | not expressed | not expressed | OE in ovary |
| on.mrna.UNK894.1.1 | - | not expressed | not expressed | not expressed | OE in testis |

| | Annotation | Expression in Gonads | | | |
|---|---|---|---|---|---|
| Module darkgreen | | *J. ornatus* | *A. burtoni* | *O. ventralis* | *E. cyanostictus* |
| on.mrna.LG1.435.1 | ATP-binding cassette, sub-family B (MDR/TAP), member 10 | OE in testis | not DE | not DE | not DE |
| on.mrna.LG1.436.1 | ATP-binding cassette, sub-family B (MDR/TAP), member 10 | OE in testis | not DE | OE in ovary | OE in ovary |
| on.mrna.LG1.553.1 | - | not expressed | not expressed | not expressed | OE in ovary |
| on.mrna.LG10.1.1 | - | not expressed | not expressed | not expressed | OE in testis |
| on.mrna.LG13.330.2 | - | not expressed | not expressed | OE in testis | OE in ovary |
| on.mrna.LG13.358.1 | oncoprotein induced transcript 3 | not expressed | not expressed | not expressed | not DE |
| on.mrna.LG13.569.1 | | OE in testis | OE in testis | OE in testis | not DE |
| on.mrna.LG14.573.1 | sel-1 suppressor of lin-12-like 3 | not expressed | not expressed | not expressed | OE in testis |
| on.mrna.LG15.157.1 | forkhead box A2 | not DE | not expressed | not expressed | OE in testis |
| on.mrna.LG15.648.1 | glycerophosphocholine phosphodiesterase GDE1 homolog | OE in ovary | not DE | OE in ovary | not DE |

| | | | | | |
|---|---|---|---|---|---|
| on.mrna.LG17.147.1 | mitochondrial 10-formyltetrahydrofolate dehydrogenase-like | OE in ovary | not DE | OE in ovary | not expressed |
| on.mrna.LG17.550.2 | - | not expressed | OE in testis | OE in testis | OE in testis |
| on.mrna.LG17.671.1 | gamma-secretase activating protein | not expressed | OE in testis | not expressed | not DE |
| on.mrna.LG18.29.1 | - | not expressed | not expressed | not expressed | OE in testis |
| on.mrna.LG18.732.1 | zgc:174946 | not expressed | not expressed | not expressed | OE in testis |
| on.mrna.LG19.142.1 | mitochondrial fission regulator 1-like | OE in testis | OE in ovary | not DE | not DE |
| on.mrna.LG19.209.5 | TRAF3 interacting protein 2 | not expressed | not expressed | not expressed | OE in ovary |
| on.mrna.LG2.466.1 | heterogeneous nuclear ribonucleoprotein A/B-like | OE in ovary | OE in ovary | OE in ovary | not DE |
| on.mrna.LG20.462.2 | SAM domain and HD domain-containing protein 1-like | not expressed | not expressed | not expressed | OE in testis |
| on.mrna.LG6.997.1 | - | not expressed | not expressed | not expressed | OE in ovary |
| on.mrna.LG7.367.1 | phosphatidylinositol 3-kinase regulatory subunit alpha-like | not DE | not DE | OE in ovary | not DE |
| on.mrna.LG9.66.2 | cyclic nucleotide gated channel beta 3 | OE in ovary | OE in ovary | OE in ovary | not DE |
| on.mrna.LG9.196.2 | sulfatase 1 | not expressed | OE in testis | not expressed | OE in testis |
| on.mrna.UNK104.1.1 | butyrophilin-like 2 | OE in testis | not DE | not DE | OE in ovary |
| on.mrna.UNK120.4.1 | gag-pol fusion protein | OE in testis | not DE | not DE | OE in ovary |
| on.mrna.UNK1218.1.1 | myelin oligodendrocyte glycoprotein | not expressed | not expressed | not expressed | OE in ovary |
| on.mrna.UNK128.12.1 | calcium-sensing receptor | not expressed | not expressed | not expressed | OE in testis |
| on.mrna.UNK128.13.1 | - | not expressed | OE in testis | not expressed | not DE |
| on.mrna.UNK137.3.1 | retrotransposable element | OE in testis | not DE | not DE | not DE |
| on.mrna.UNK137.8.1 | V-set domain containing T cell activation inhibitor 1 | OE in testis | not DE | not DE | not DE |
| on.mrna.UNK1384.1.1 | myelin oligodendrocyte glycoprotein | not expressed | not expressed | not expressed | OE in ovary |
| on.mrna.UNK1399.1.1 | - | not expressed | not expressed | OE in ovary | OE in ovary |
| on.mrna.UNK142.3.1 | - | OE in testis | OE in testis | not expressed | OE in ovary |
| on.mrna.UNK146.1.1 | - | not expressed | not expressed | OE in testis | not DE |
| on.mrna.UNK149.1.1 | - | not expressed | not expressed | not expressed | OE in ovary |
| on.mrna.UNK1558.1.1 | retrotransposon-like 1 | not expressed | not expressed | not expressed | OE in testis |
| on.mrna.UNK161.2.1 | - | not expressed | not DE | not expressed | not DE |
| on.mrna.UNK165.1.1 | V-set domain containing T cell activation inhibitor 1 | OE in testis | not DE | not expressed | not DE |
| on.mrna.UNK17.31.1 | v-crk sarcoma virus CT10 oncogene homolog (avian) | OE in testis | not DE | not DE | OE in ovary |
| on.mrna.UNK17.33.2 | - | not DE | not DE | not DE | not DE |
| on.mrna.UNK183.1.1 | V-set domain containing T cell activation inhibitor 1 | OE in testis | OE in ovary | not DE | not DE |
| on.mrna.UNK183.3.1 | harbinger transposase derived 1 | OE in testis | not DE | not DE | not DE |
| on.mrna.UNK183.5.1 | butyrophilin-like 2 (MHC class II associated) | OE in testis | not DE | not DE | not DE |
| on.mrna.UNK198.1.1 | myelin oligodendrocyte glycoprotein | OE in testis | not DE | not DE | not DE |
| on.mrna.UNK198.3.1 | HERV-H LTR-associating 2 | OE in testis | not DE | not DE | not DE |
| on.mrna.UNK198.4.1 | V-set domain containing T cell activation inhibitor 1 | OE in testis | not DE | not DE | not DE |
| on.mrna.UNK210.1.1 | CD276 molecule | not expressed | not expressed | not expressed | not DE |
| on.mrna.UNK210.3.1 | butyrophilin-like 2 | OE in testis | not DE | not DE | not DE |
| on.mrna.UNK2294.1.1 | butyrophilin-like 10 | OE in testis | not expressed | not DE | OE in ovary |
| on.mrna.UNK25.15.1 | expressed sequence BC139653 | not expressed | not expressed | not expressed | OE in ovary |
| on.mrna.UNK26.39.1 | adseverin-like | not DE | not DE | OE in ovary | not DE |
| on.mrna.UNK272.1.1 | butyrophilin-like 2 | OE in testis | not DE | not DE | not DE |
| on.mrna.UNK295.1.1 | HERV-H LTR-associating 2 | OE in testis | not DE | not DE | not DE |
| on.mrna.UNK3.66.1 | arrestin-C-like | not expressed | not expressed | not expressed | not DE |
| on.mrna.UNK30.5.2 | loss of heterozygosity 12 chromosomal region 1 protein homolog | not DE | not DE | not DE | not DE |
| on.mrna.UNK311.4.1 | zinc finger and SCAN domain containing 30 | not expressed | OE in testis | not expressed | OE in ovary |
| on.mrna.UNK32.9.1 | podocin-like | not DE | not DE | not DE | not DE |
| on.mrna.UNK32.20.1 | Rho guanine nucleotide exchange factor (GEF) 39 | OE in testis | not expressed | not expressed | OE in ovary |
| on.mrna.UNK333.4.1 | uncharacterized LOC100702698 | OE in testis | not DE | not DE | not DE |
| on.mrna.UNK340.5.1 | - | OE in testis | not DE | not DE | not DE |
| on.mrna.UNK353.1.1 | butyrophilin-like 8 | OE in testis | OE in ovary | OE in ovary | OE in ovary |
| on.mrna.UNK386.3.1 | - | OE in testis | not expressed | not expressed | OE in testis |
| on.mrna.UNK396.5.1 | - | not expressed | not expressed | not expressed | OE in ovary |
| on.mrna.UNK398.1.1 | myelin oligodendrocyte glycoprotein | OE in testis | not DE | not DE | not DE |
| on.mrna.UNK4.2.1 | 5-hydroxytryptamine (serotonin) receptor 3D, ionotropic | not expressed | not expressed | not expressed | not DE |
| on.mrna.UNK4151.1.1 | myelin oligodendrocyte glycoprotein | OE in testis | OE in ovary | not DE | not DE |
| on.mrna.UNK4181.1.1 | zgc:113210 | OE in testis | not expressed | not expressed | OE in testis |
| on.mrna.UNK42.1.1 | butyrophilin-like 2 | not DE | OE in testis | not expressed | not DE |
| on.mrna.UNK42.13.1 | butyrophilin-like 2 | OE in testis | not DE | not DE | not DE |
| on.mrna.UNK42.17.1 | V-set domain containing T cell activation inhibitor 1 | OE in testis | not DE | not DE | not DE |
| on.mrna.UNK42.20.1 | HERV-H LTR-associating 2 | not DE | not DE | not DE | not DE |
| on.mrna.UNK421.2.1 | uncharacterized LOC100892091 | OE in testis | not expressed | not expressed | OE in testis |
| on.mrna.UNK479.2.1 | - | not expressed | not expressed | not expressed | not DE |
| on.mrna.UNK504.1.1 | gypsy retrotransposon integrase 1 | OE in testis | not DE | not DE | not DE |
| on.mrna.UNK516.2.1 | HERV-H LTR-associating 2 | not expressed | not expressed | not expressed | OE in ovary |
| on.mrna.UNK529.2.1 | - | not expressed | not expressed | not expressed | OE in testis |
| on.mrna.UNK529.3.1 | - | not expressed | not expressed | not expressed | OE in testis |
| on.mrna.UNK53.28.2 | fam151b | OE in testis | OE in testis | not DE | OE in ovary |
| on.mrna.UNK59.1.1 | butyrophilin-like 2 | OE in testis | not DE | not DE | not DE |
| on.mrna.UNK59.2.1 | V-set domain containing T cell activation inhibitor 1 | OE in testis | OE in testis | OE in testis | OE in ovary |
| on.mrna.UNK59.3.1 | - | OE in testis | not DE | not DE | OE in ovary |
| on.mrna.UNK59.5.1 | - | not DE | not DE | not DE | not DE |
| on.mrna.UNK59.8.1 | V-set domain containing T cell activation inhibitor 1 | not DE | OE in testis | not expressed | not DE |
| on.mrna.UNK59.10.1 | V-set domain containing T cell activation inhibitor 1 | OE in testis | not DE | not DE | not DE |
| on.mrna.UNK59.14.1 | CD276 molecule | not DE | OE in testis | not DE | not DE |
| on.mrna.UNK59.16.1 | - | OE in testis | not expressed | not DE | not DE |
| on.mrna.UNK591.2.1 | matrix-remodelling associated 8 | not DE | not DE | OE in ovary | not DE |

| | | | | | |
|---|---|---|---|---|---|
| on.mrna.UNK62.12.1 | V-set domain containing T cell activation inhibitor 1 | OE in testis | not expressed | not expressed | not DE |
| on.mrna.UNK624.1.1 | - | OE in testis | OE in ovary | not DE | not DE |
| on.mrna.UNK628.1.1 | butyrophilin-like 10 | OE in testis | not DE | not DE | not DE |
| on.mrna.UNK65.25.1 | zinc finger, BED-type containing 1 | not DE | OE in testis | not expressed | OE in ovary |
| on.mrna.UNK662.1.1 | matrix-remodelling associated 8 | not expressed | not DE | not DE | OE in ovary |
| on.mrna.UNK664.1.1 | butyrophilin-like 2 | OE in testis | not DE | not DE | not DE |
| on.mrna.UNK71.21.2 | - | not expressed | not expressed | not expressed | OE in ovary |
| on.mrna.UNK717.1.1 | V-set domain containing T cell activation inhibitor 1 | OE in testis | OE in ovary | not DE | OE in ovary |
| on.mrna.UNK75.15.1 | zgc:152698 | not expressed | not expressed | not DE | not expressed |
| on.mrna.UNK77.4.1 | - | OE in testis | not DE | not DE | not DE |
| on.mrna.UNK83.23.1 | - | not expressed | not expressed | not expressed | not DE |
| on.mrna.UNK848.1.1 | - | not expressed | OE in testis | not expressed | OE in testis |

# Supplementary Material Table 15

Assignment of gene candidates to modules

| module | gene |
| --- | --- |
| black | ara |
| black | cyp11b2 |
| black | foxl2a |
| black | foxl2b |
| blue | nanos1b |
| blue | amh |
| blue | ctnnb1a |
| blue | esr2a |
| blue | esr2b |
| blue | gata4 |
| blue | nanos1A |
| brown | arb |
| brown | cyp19a1b |
| brown | esr1 |
| brown | figla |
| brown | nr5a2 |
| brown | sox9A |
| brown | sox9B |
| greenyellow | cyp19a1a |
| grey | nr5a5 |
| magenta | amh |
| magenta | dmrt1 |
| magenta | sf1A(nr5a1) |
| magenta | tdrd1 |
| magenta | wnt4a |
| magenta | wt1a |
| midnightblue | wnt4b |
| orange | dax1a |
| | |
| not in network | rspondin |
| not in network | gperA |

**Supplementary Material Table 16 Pathways enriched on Nile tilapia LG1 and LG23**

**Female transcripts on LG1**

##Databases: BioCarta, Gene Ontology, GAD, FunDO, KEGG PATHWAY, OMIM, PID, PANTHER, Reactome, BioCyc,

## NHGRI GWAS Catalog, KEGG DISEASE

##Statistical test method: binomial test

##FDR correction method: Benjamini and Hochberg

| #Term | Database | ID | Input number | Background number | P-Value | Corrected P-Value |
|---|---|---|---|---|---|---|
| Stabilization and expansion of the E-cadherin adherens junction | PID | ecadherin_stabilization_pathway | 44 | 6 | 5.66E-92 | 1.51E-89 |
| Protein folding | Reactome | | 44 | 6 | 1.73E-82 | 2.79E-80 |
| Chaperonin-mediated protein folding | Reactome | | 44 | 6 | 1.73E-82 | 2.79E-80 |
| Adherens junction | KEGG PATHWAY | hsa04520 | 55 | 35 | 4.09E-67 | 3.65E-65 |
| Metabolism of proteins | Reactome | REACT_17015 | 44 | 55 | 7.12E-41 | 2.29E-39 |
| tRNA charging | BioCyc | TRNA-CHARGING-PWY | 9 | 8 | 5.08E-14 | 5.23E-13 |
| tRNA Aminoacylation | Reactome | | 9 | 8 | 1.77E-10 | 1.76E-09 |
| Aminoacyl-tRNA biosynthesis | KEGG PATHWAY | hsa00970 | 9 | 8 | 3.87E-10 | 3.74E-09 |
| Pyruvate metabolism and Citric Acid (TCA) cycle | Reactome | | 6 | 6 | 4.08E-07 | 3.38E-06 |
| The citric acid (TCA) cycle and respiratory electron transport | Reactome | | 6 | 6 | 4.08E-07 | 3.38E-06 |
| Gene Expression | Reactome | REACT_71 | 10 | 48 | 7.81E-05 | 0.0005752 |
| Cysteine and methionine metabolism | KEGG PATHWAY | hsa00270 | 4 | 5 | 0.0001281 | 0.0009024 |
| Dorso-ventral axis formation | KEGG PATHWAY | hsa04320 | 4 | 6 | 0.0002557 | 0.0017551 |
| Progesterone-mediated oocyte maturation | KEGG PATHWAY | hsa04914 | 4 | 8 | 0.0007488 | 0.0044871 |
| Hypertrophic cardiomyopathy (HCM) | KEGG PATHWAY | hsa05410 | 3 | 7 | 0.0054206 | 0.0262213 |
| Dilated cardiomyopathy | KEGG PATHWAY | hsa05414 | 3 | 7 | 0.0054206 | 0.0262213 |
| Cardiac muscle contraction | KEGG PATHWAY | hsa04260 | 3 | 9 | 0.0107233 | 0.0478378 |
| il-2 receptor beta chain in t cell activation | BioCarta | 100129 | 1 | 5 | 0.0109650 | 0.0485751 |

**Supplementary Material Table 16 Pathways enriched on Nile tilapia LG1 and LG23**

| **Male transcripts on LG23** | | | Input | Background | | |
|---|---|---|---|---|---|---|
| #Term | Database | ID | number | number | P-Value | Corrected P-Value |
| Notch signaling pathway | PID | notch_pathway | 23 | 5 | 4.35E-29 | 4.39E-26 |
| @@Notch signaling pathway | PANTHER | P00045 | 13 | 5 | 1.59E-19 | 5.36E-18 |
| Endocrine and other factor-regulated calcium reabsorption | KEGG PATHWAY | hsa04961 | 11 | 14 | 1.27E-13 | 2.30E-12 |
| Bacterial invasion of epithelial cells | KEGG PATHWAY | hsa05100 | 11 | 15 | 2.67E-13 | 4.64E-12 |
| Synaptic vesicle cycle | KEGG PATHWAY | hsa04721 | 13 | 27 | 3.15E-13 | 5.39E-12 |
| Endocytosis | KEGG PATHWAY | hsa04144 | 11 | 23 | 2.49E-11 | 3.81E-10 |
| Ion channel transport | Reactome | | 9 | 9 | 7.42E-10 | 9.61E-09 |
| Neurotrophic factor-mediated Trk receptor signaling | PID | trkrpathway | 10 | 5 | 1.59E-09 | 2.03E-08 |
| NGF signalling via TRKA from the plasma membrane | Reactome | | 11 | 21 | 7.55E-09 | 8.29E-08 |
| Signalling by NGF | Reactome | | 11 | 21 | 7.55E-09 | 8.29E-08 |
| Thromboxane A2 receptor signaling | PID | txa2pathway | 10 | 6 | 8.86E-09 | 9.52E-08 |
| Ephrin B reverse signaling | PID | ephrinbrevpathway | 10 | 6 | 8.86E-09 | 9.52E-08 |
| Transmembrane transport of small molecules | Reactome | REACT_15518 | 12 | 38 | 3.62E-07 | 2.66E-06 |
| MHC class II antigen presentation | Reactome | | 11 | 33 | 6.67E-07 | 4.55E-06 |
| Lysosome | KEGG PATHWAY | hsa04142 | 5 | 8 | 2.31E-06 | 1.49E-05 |
| Membrane Trafficking | Reactome | REACT_11123 | 10 | 31 | 2.88E-06 | 1.83E-05 |
| L1CAM interactions | Reactome | | 11 | 39 | 3.28E-06 | 2.07E-05 |
| CXCR4-mediated signaling events | PID | cxcr4_pathway | 10 | 12 | 4.82E-06 | 2.97E-05 |
| Adaptive Immune System | Reactome | | 13 | 69 | 3.07E-05 | 0.0001669 |
| Nucleotide excision repair | KEGG PATHWAY | hsa03420 | 4 | 8 | 5.91E-05 | 0.0003144 |
| Signal Transduction | Reactome | REACT_111102 | 15 | 100 | 9.89E-05 | 0.0005151 |
| Axon guidance | Reactome | | 11 | 65 | 0.0003132 | 0.0014851 |
| Validated transcriptional targets of TAp63 isoforms | PID | tap63pathway | 5 | 5 | 0.0005962 | 0.0027249 |
| Developmental Biology | Reactome | REACT_111045 | 11 | 71 | 0.0006483 | 0.0029232 |
| Immune System | Reactome | REACT_6900 | 13 | 130 | 0.0095953 | 0.0328518 |
| Tight junction | KEGG PATHWAY | hsa04530 | 3 | 18 | 0.0113905 | 0.0379685 |
| WNT ligand biogenesis and trafficking | Reactome | | 4 | 22 | 0.0213470 | 0.0678001 |
| Signaling by Wnt | Reactome | | 4 | 25 | 0.0319734 | 0.0952601 |
